# Supplementary figures and images for: Sclerostin promotes human dental pulp cells senescence (part 1 of 2)
Source: PeerJ. 2018 Oct 17;6:e5808. doi: 10.7717/peerj.5808 (PMC6195797; doi:10.7717/peerj.5808)

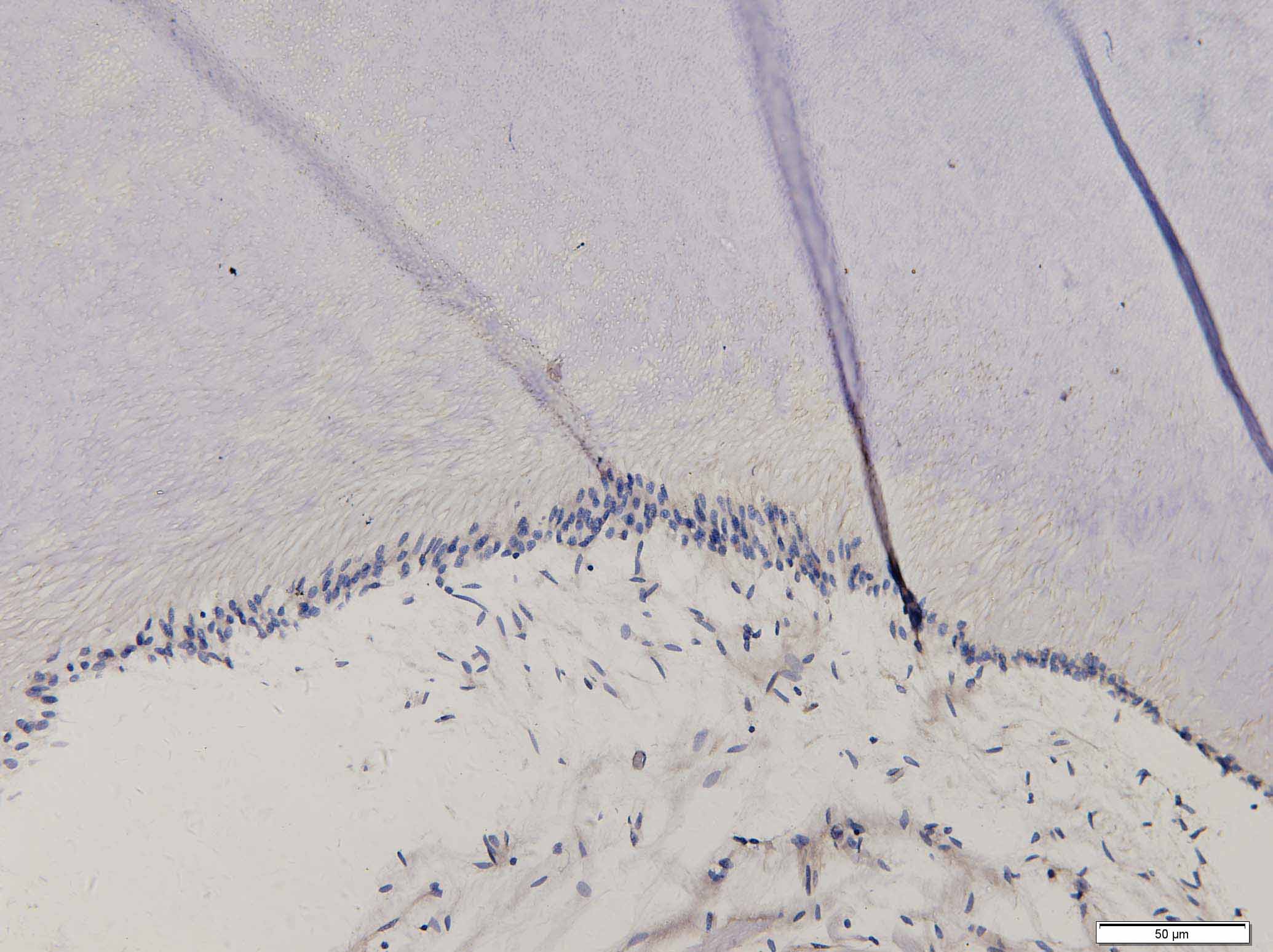

Supplement: Supplemental Information 1 — Immunohistochemical staining for sclerostin in young and senescent dental pulps. [file peerj-06-5808-s001.zip › Senescent/Image_9223.jpg]

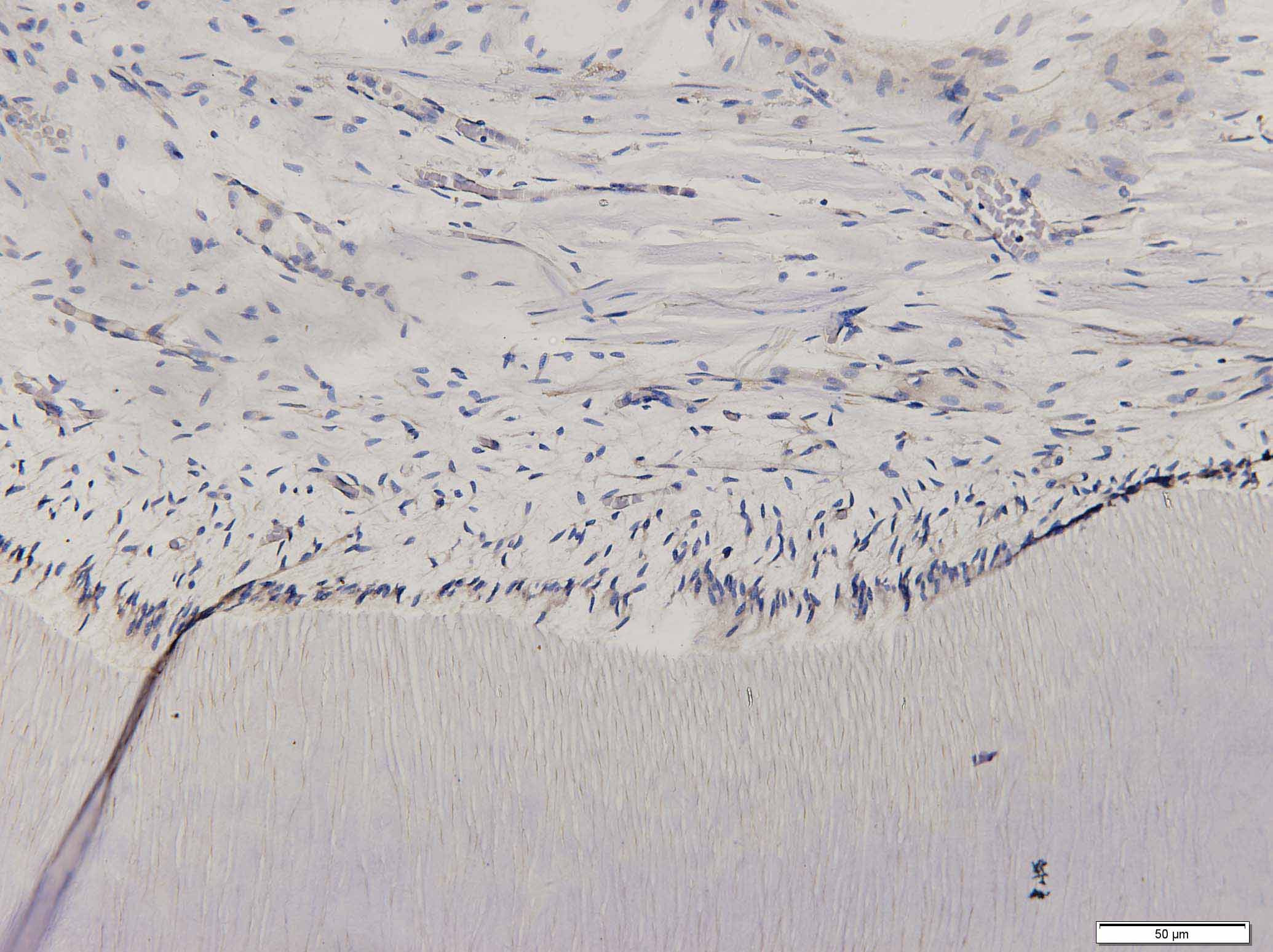

Supplement: Supplemental Information 1 — Immunohistochemical staining for sclerostin in young and senescent dental pulps. [file peerj-06-5808-s001.zip › Senescent/Image_9224.jpg]

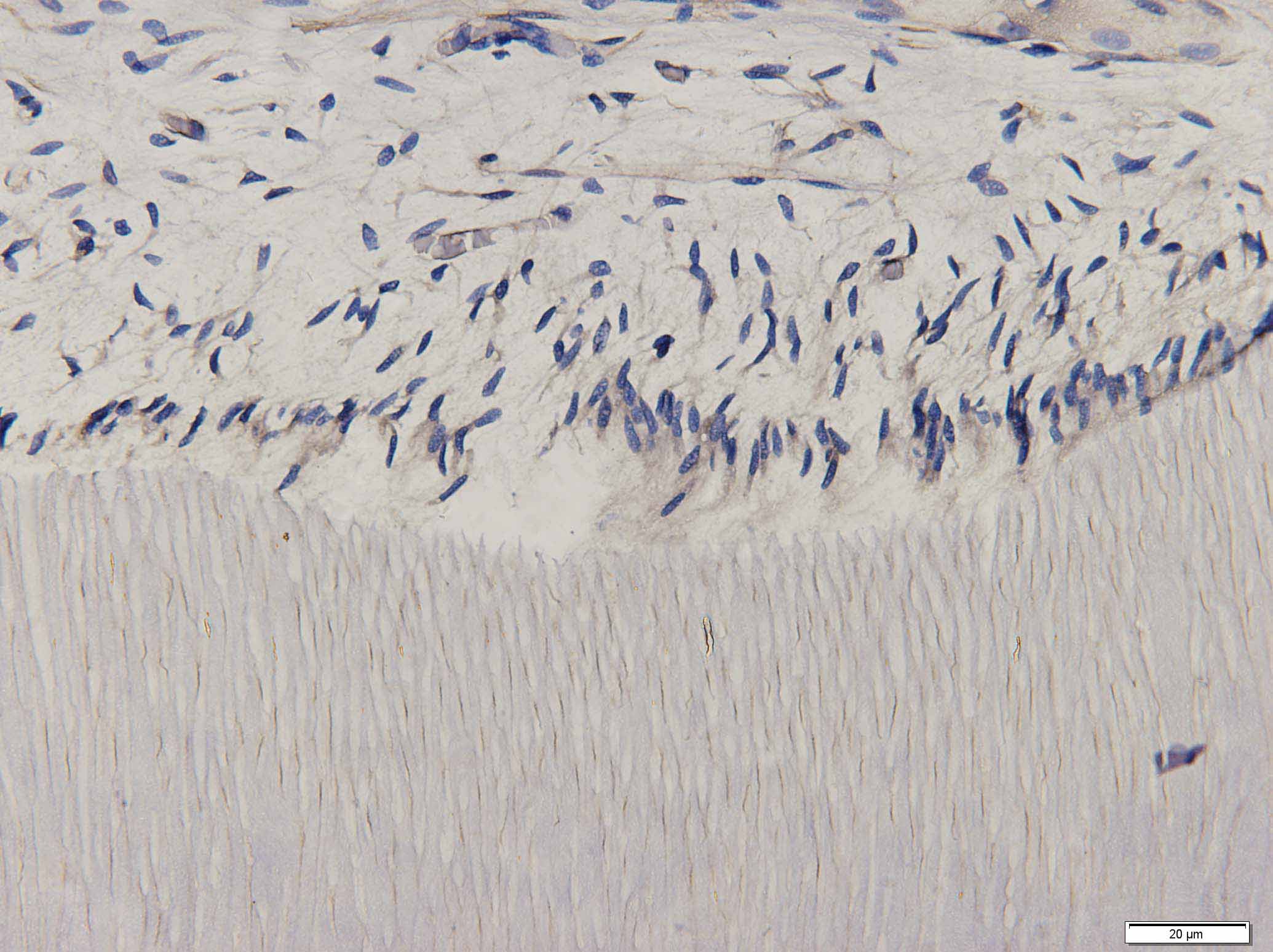

Supplement: Supplemental Information 1 — Immunohistochemical staining for sclerostin in young and senescent dental pulps. [file peerj-06-5808-s001.zip › Senescent/Image_9225.jpg]

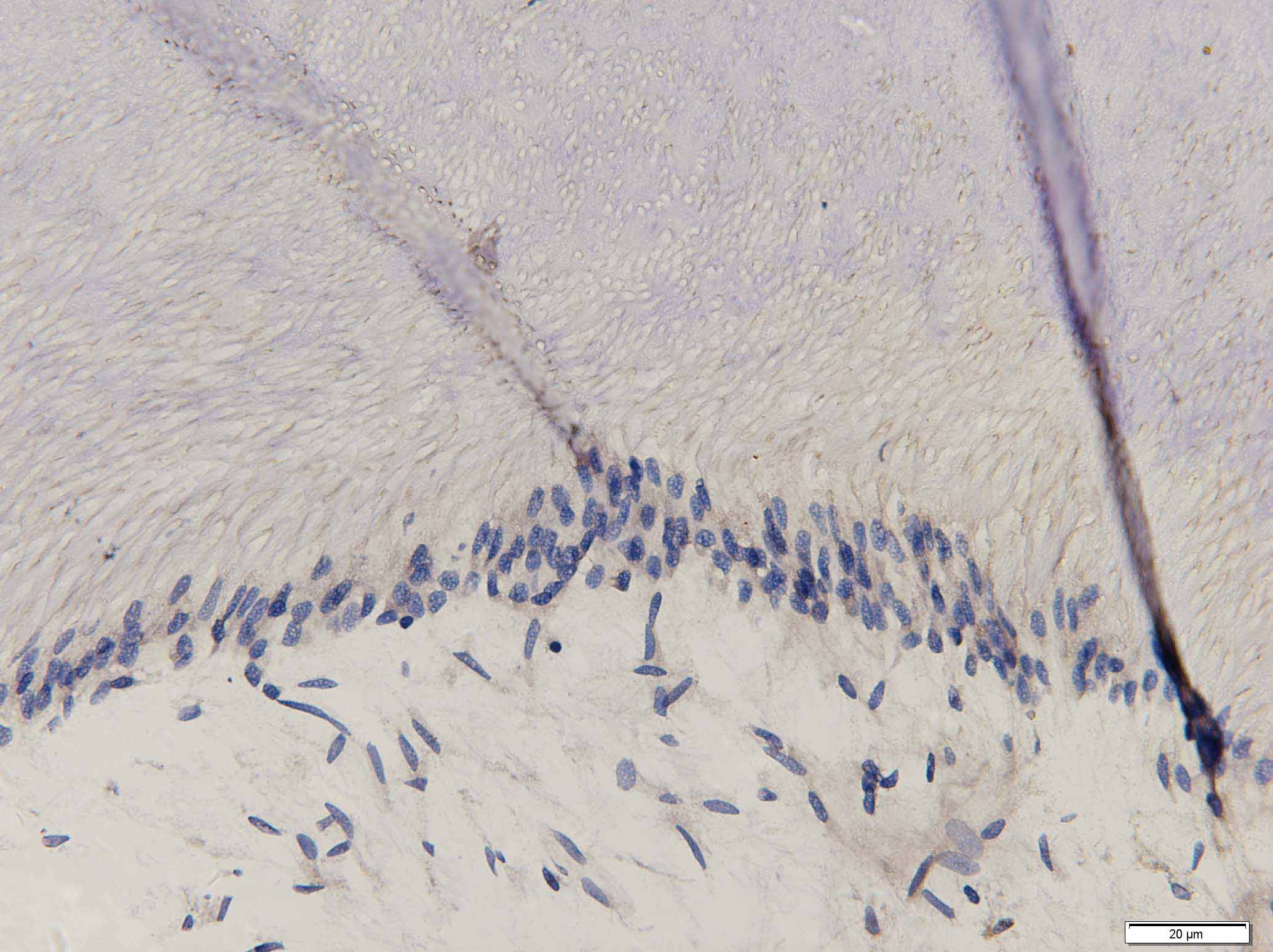

Supplement: Supplemental Information 1 — Immunohistochemical staining for sclerostin in young and senescent dental pulps. [file peerj-06-5808-s001.zip › Senescent/Image_9226.jpg]

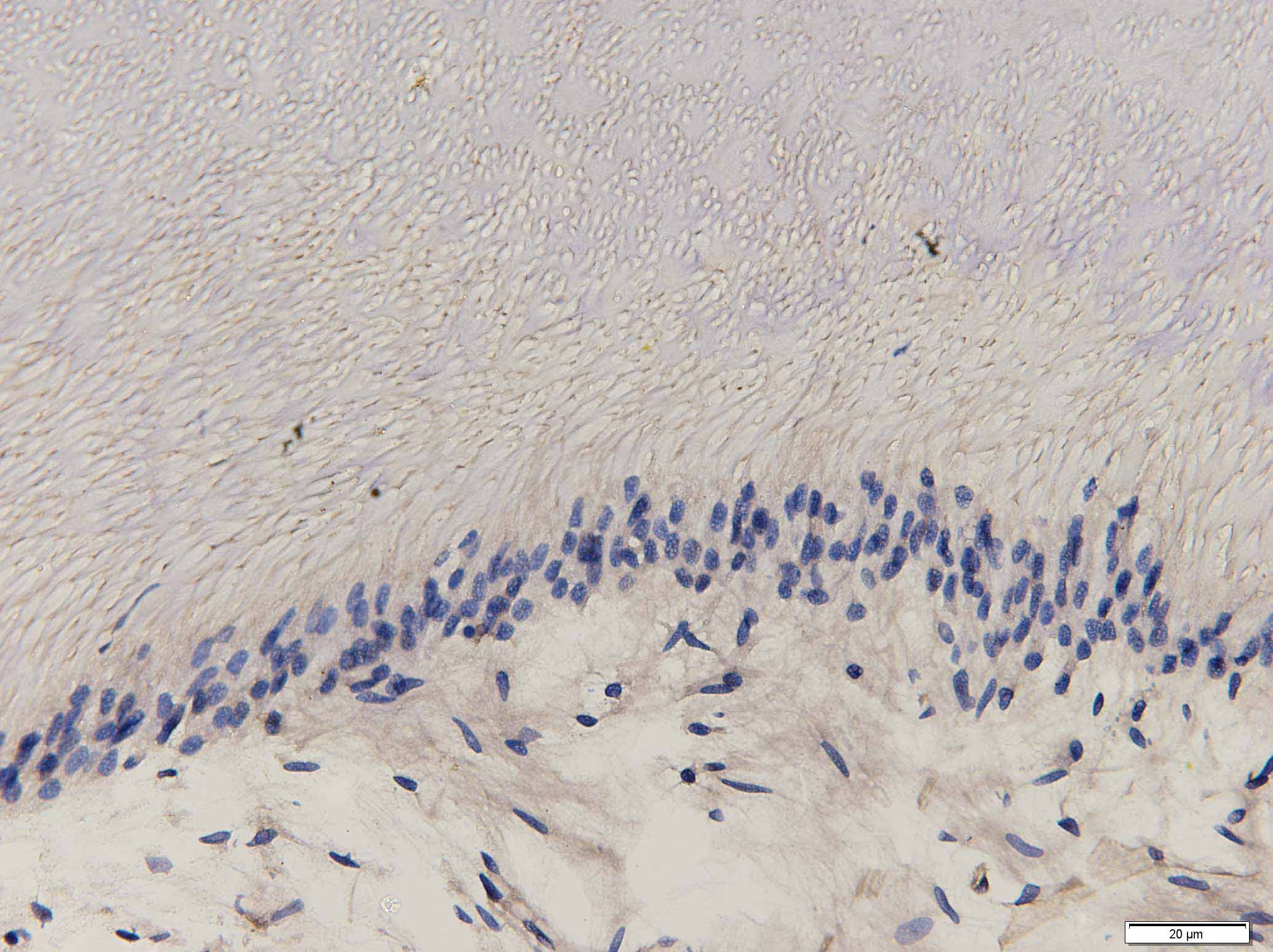

Supplement: Supplemental Information 1 — Immunohistochemical staining for sclerostin in young and senescent dental pulps. [file peerj-06-5808-s001.zip › Senescent/Image_9227.jpg]

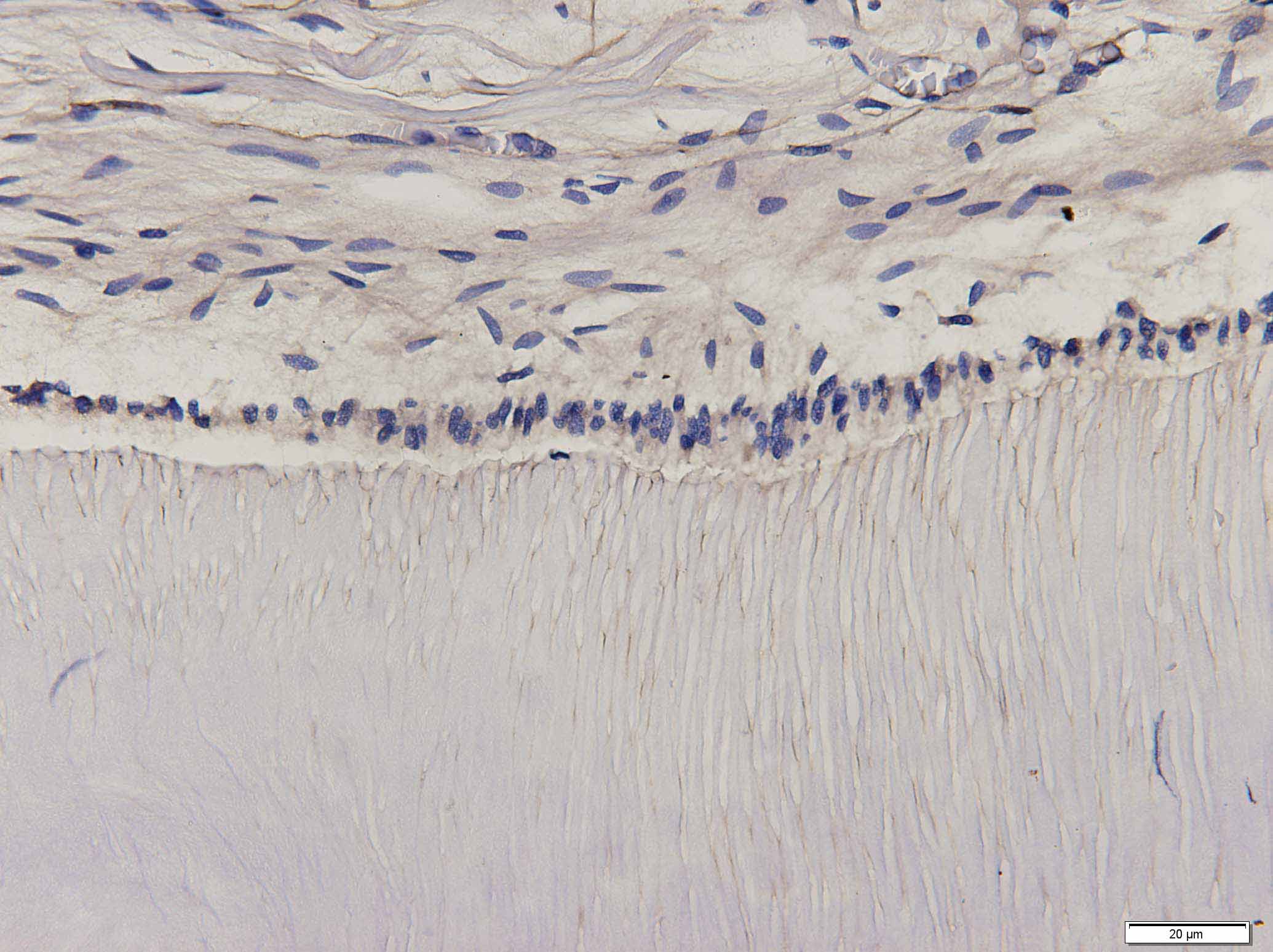

Supplement: Supplemental Information 1 — Immunohistochemical staining for sclerostin in young and senescent dental pulps. [file peerj-06-5808-s001.zip › Senescent/Image_9228.jpg]

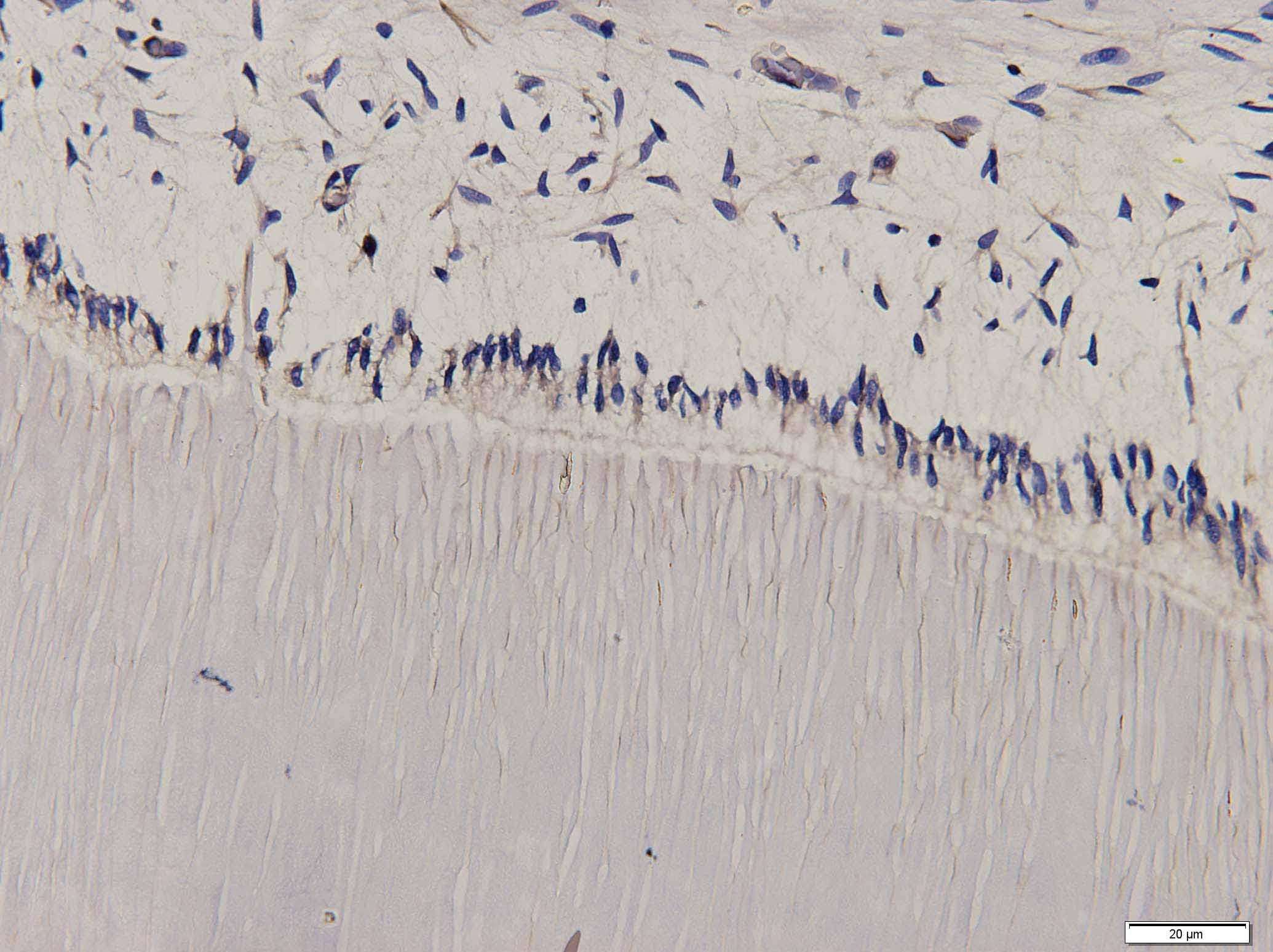

Supplement: Supplemental Information 1 — Immunohistochemical staining for sclerostin in young and senescent dental pulps. [file peerj-06-5808-s001.zip › Senescent/Image_9229.jpg]

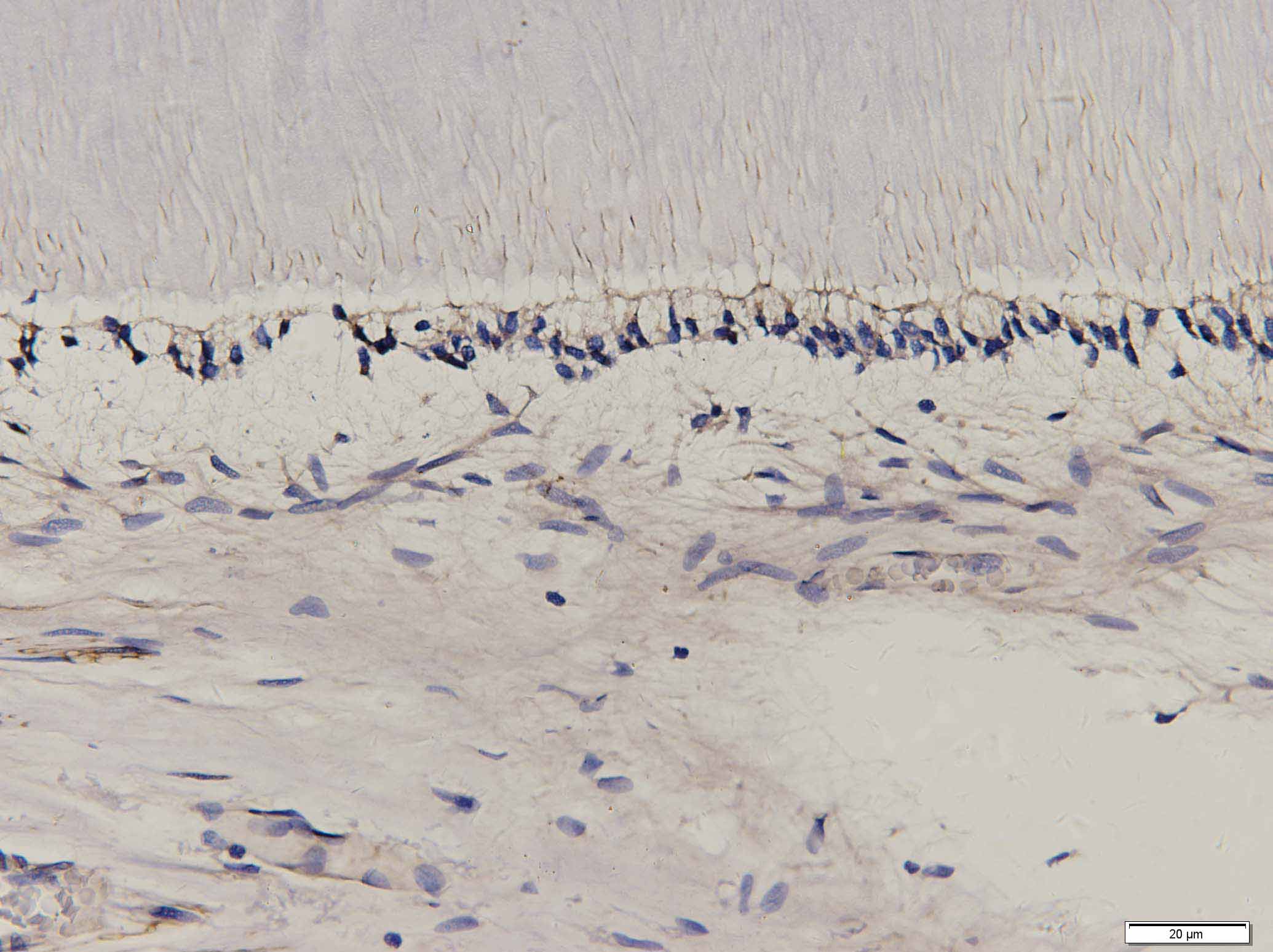

Supplement: Supplemental Information 1 — Immunohistochemical staining for sclerostin in young and senescent dental pulps. [file peerj-06-5808-s001.zip › Senescent/Image_9230.jpg]

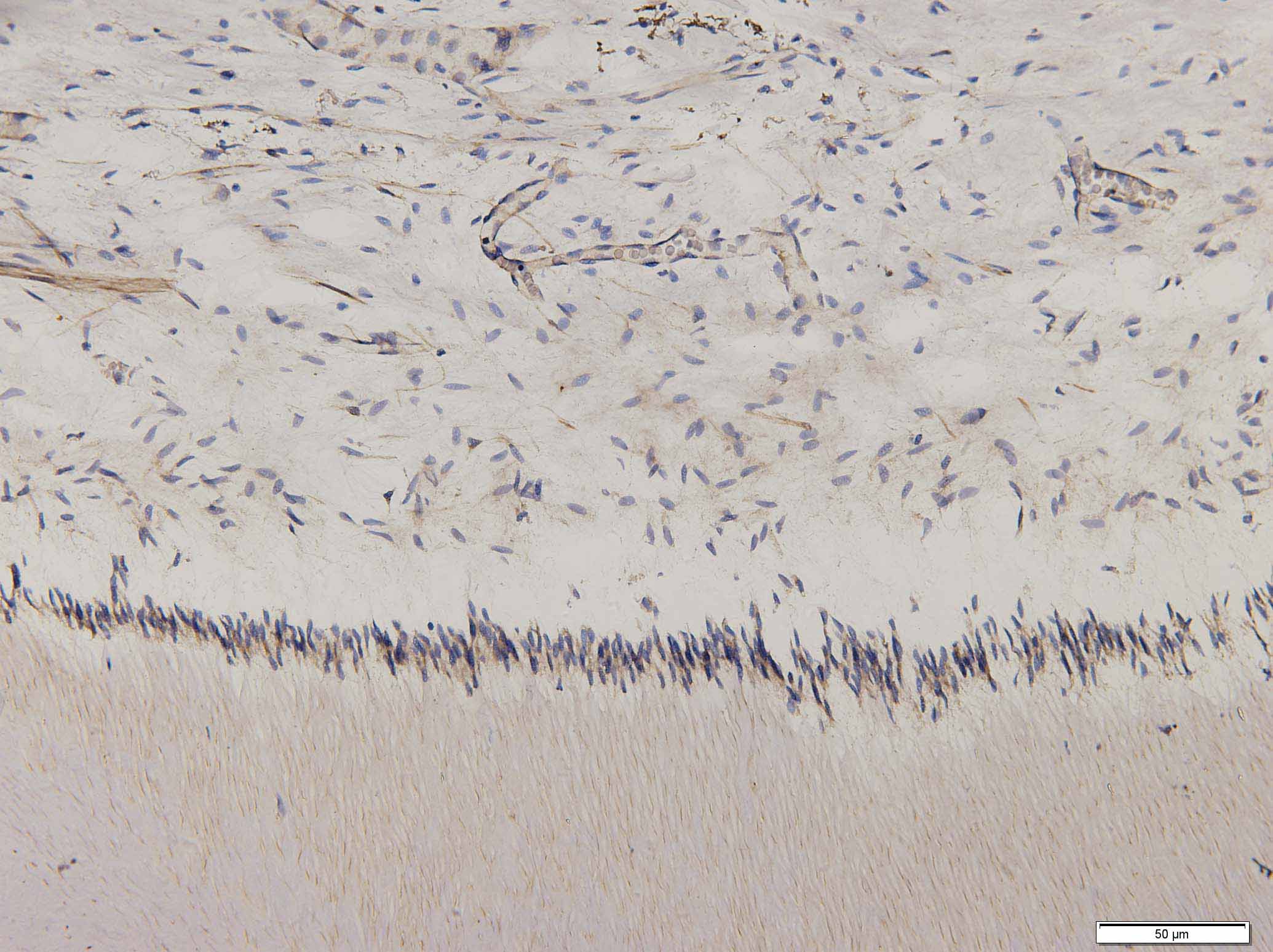

Supplement: Supplemental Information 1 — Immunohistochemical staining for sclerostin in young and senescent dental pulps. [file peerj-06-5808-s001.zip › Senescent/Image_9233.jpg]

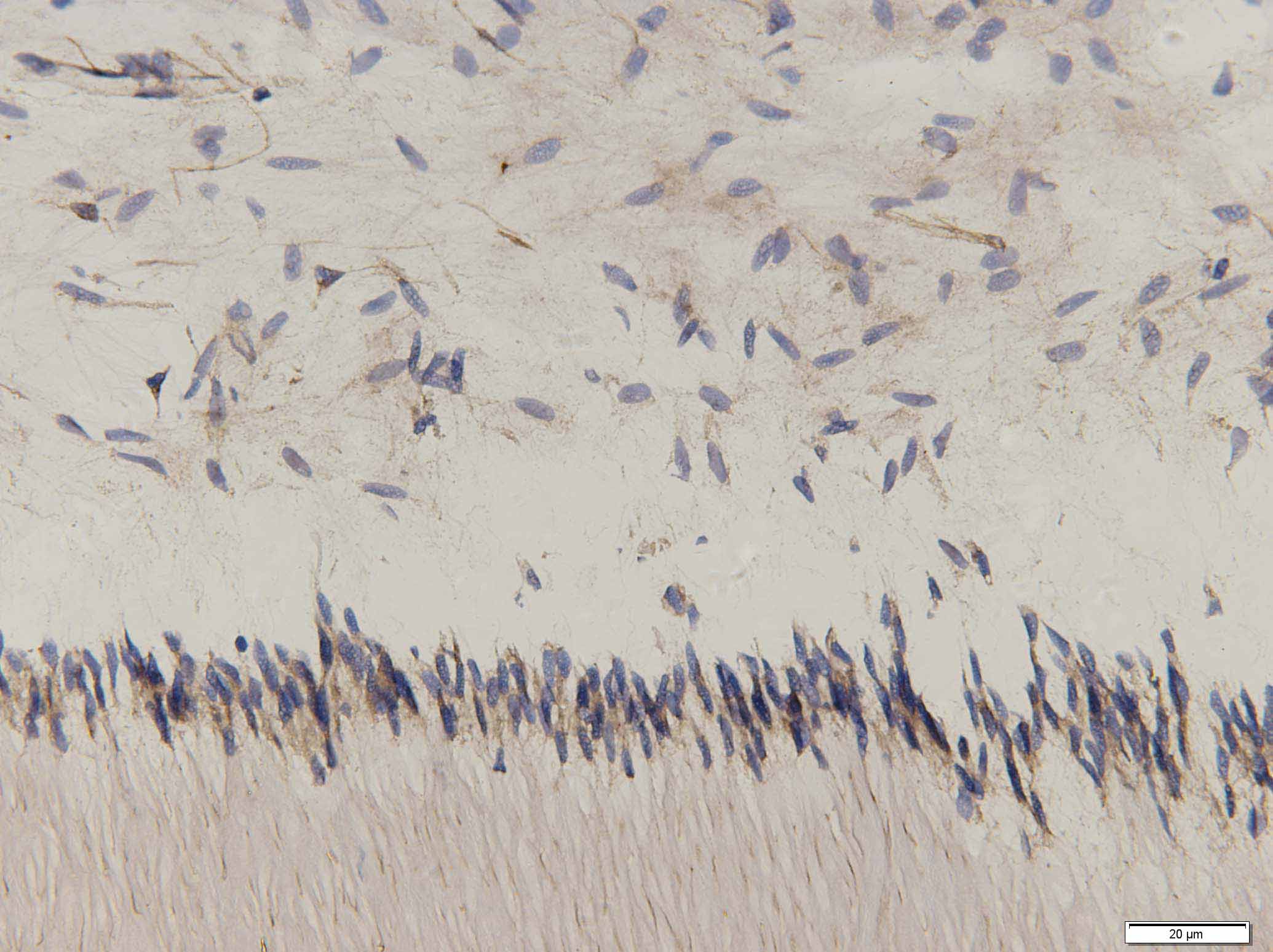

Supplement: Supplemental Information 1 — Immunohistochemical staining for sclerostin in young and senescent dental pulps. [file peerj-06-5808-s001.zip › Senescent/Image_9234.jpg]

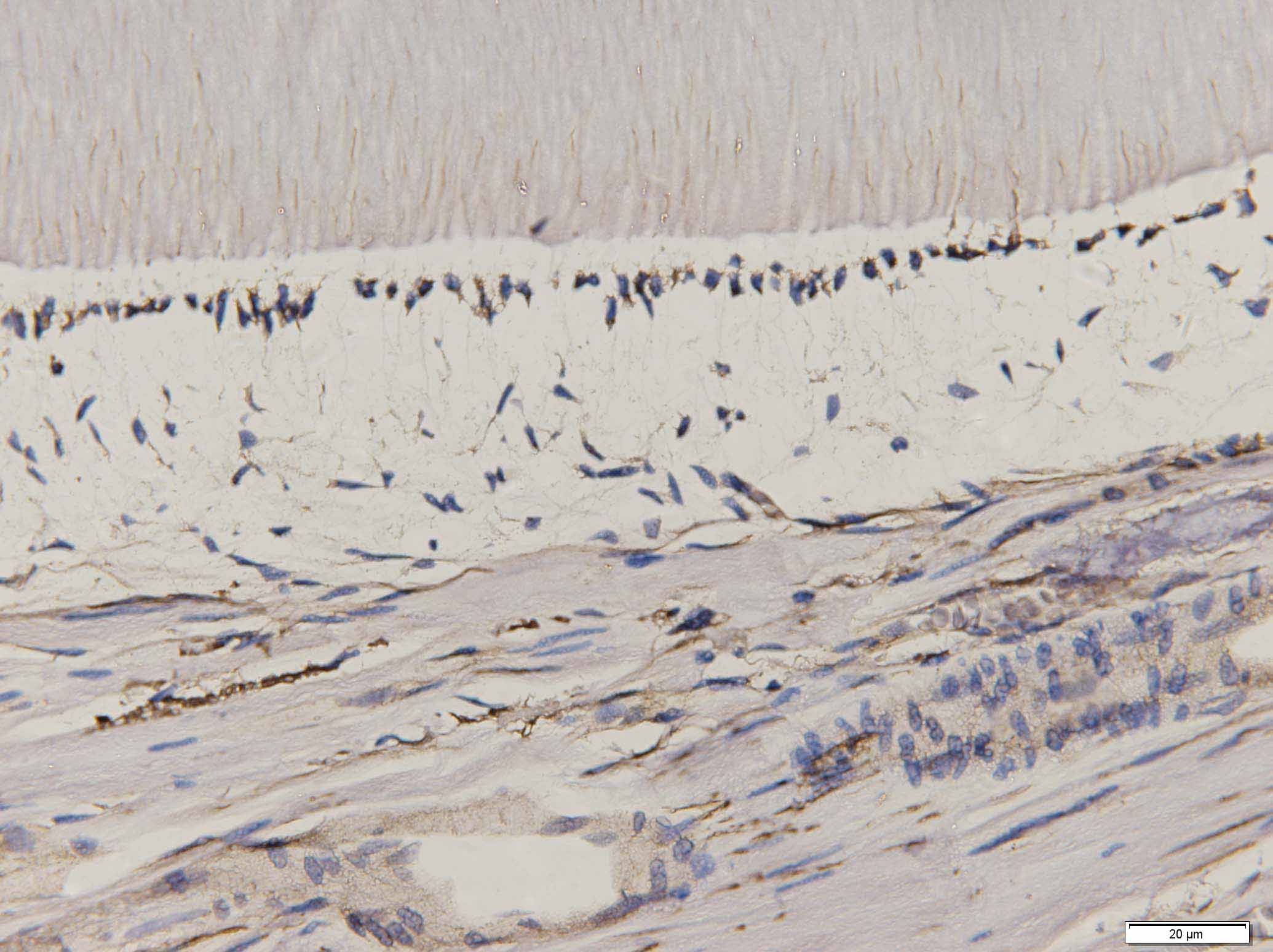

Supplement: Supplemental Information 1 — Immunohistochemical staining for sclerostin in young and senescent dental pulps. [file peerj-06-5808-s001.zip › Senescent/Image_9235.jpg]

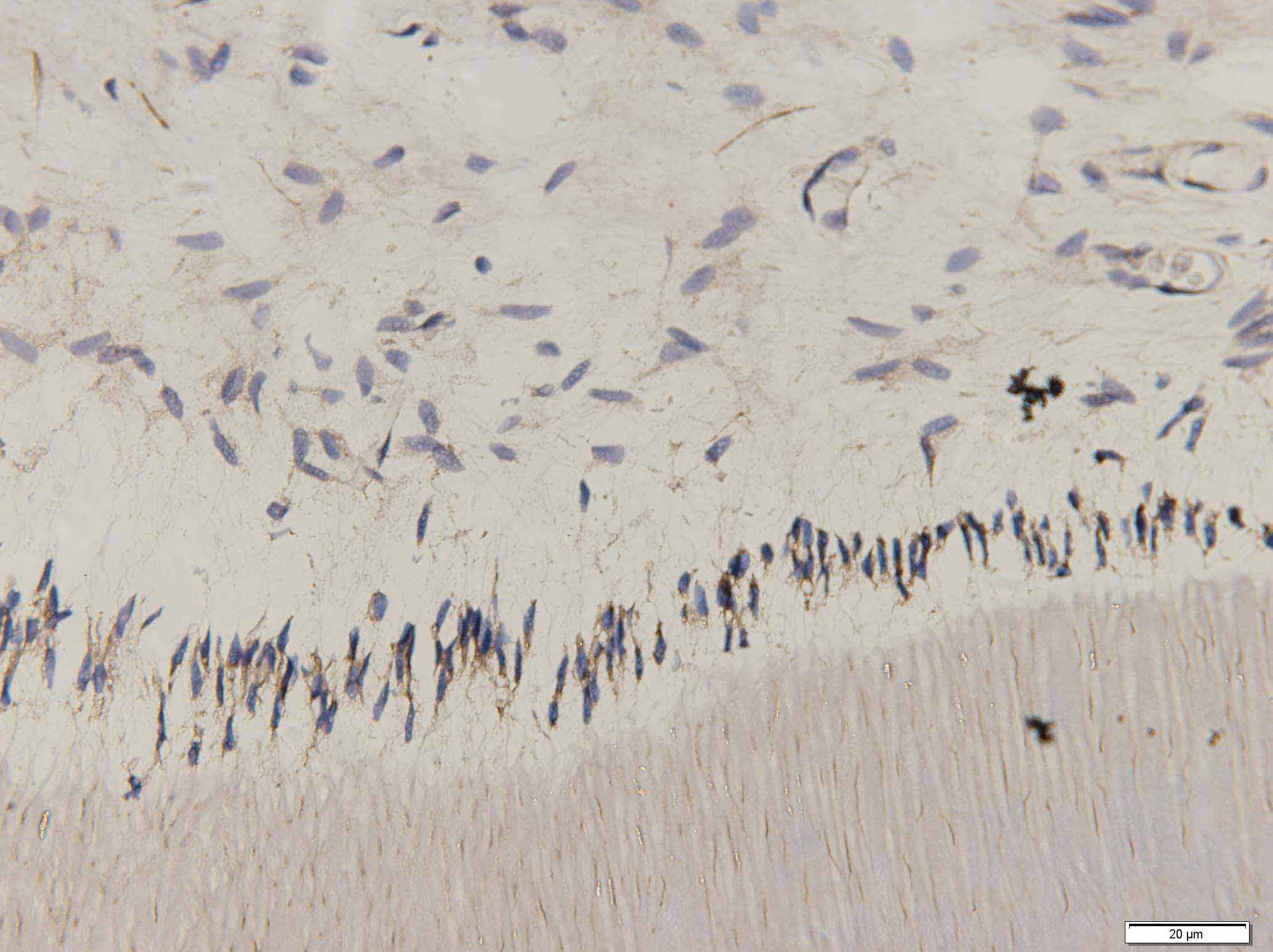

Supplement: Supplemental Information 1 — Immunohistochemical staining for sclerostin in young and senescent dental pulps. [file peerj-06-5808-s001.zip › Senescent/Image_9236.jpg]

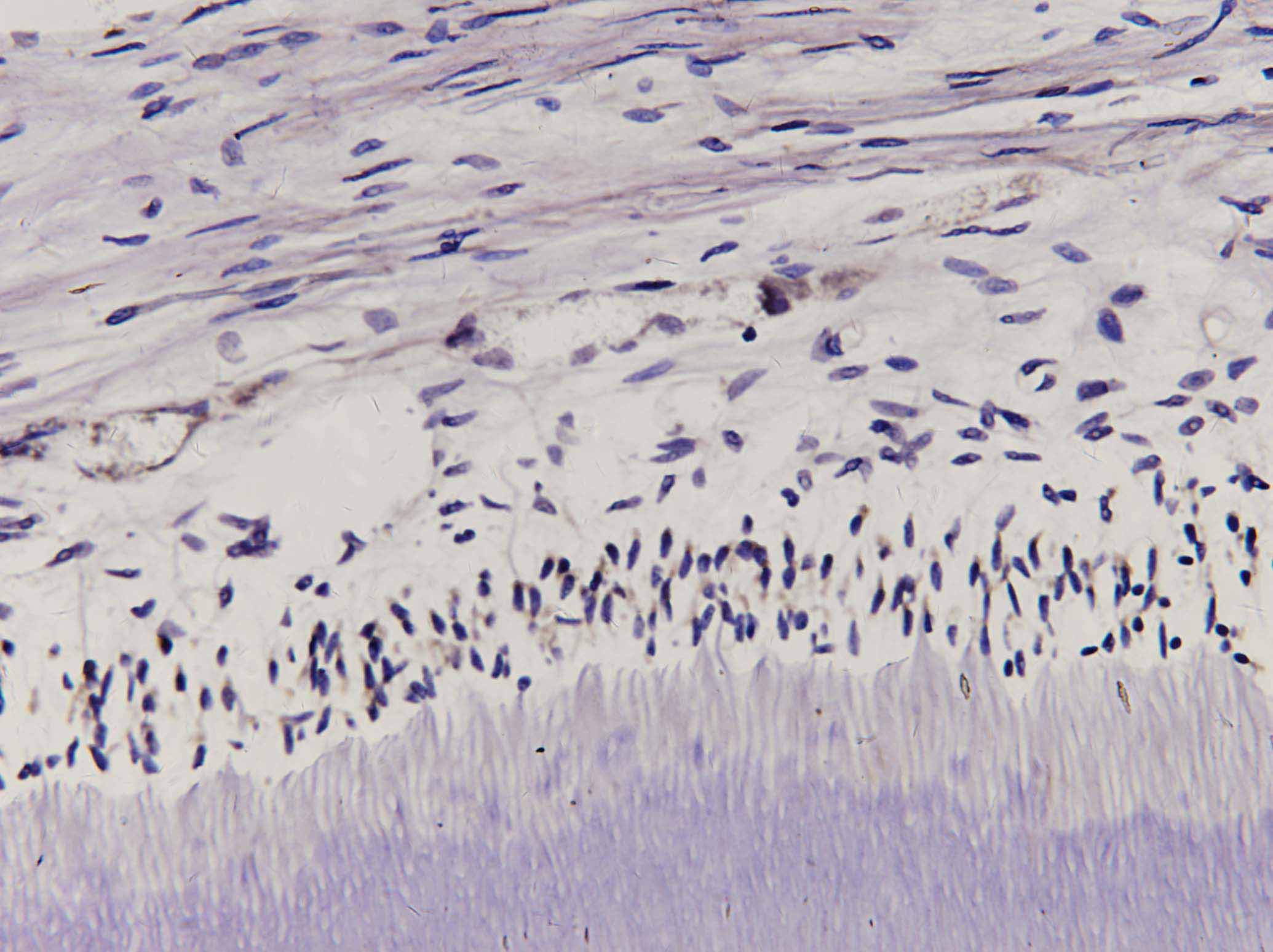

Supplement: Supplemental Information 1 — Immunohistochemical staining for sclerostin in young and senescent dental pulps. [file peerj-06-5808-s001.zip › Senescent/Image_9244.jpg]

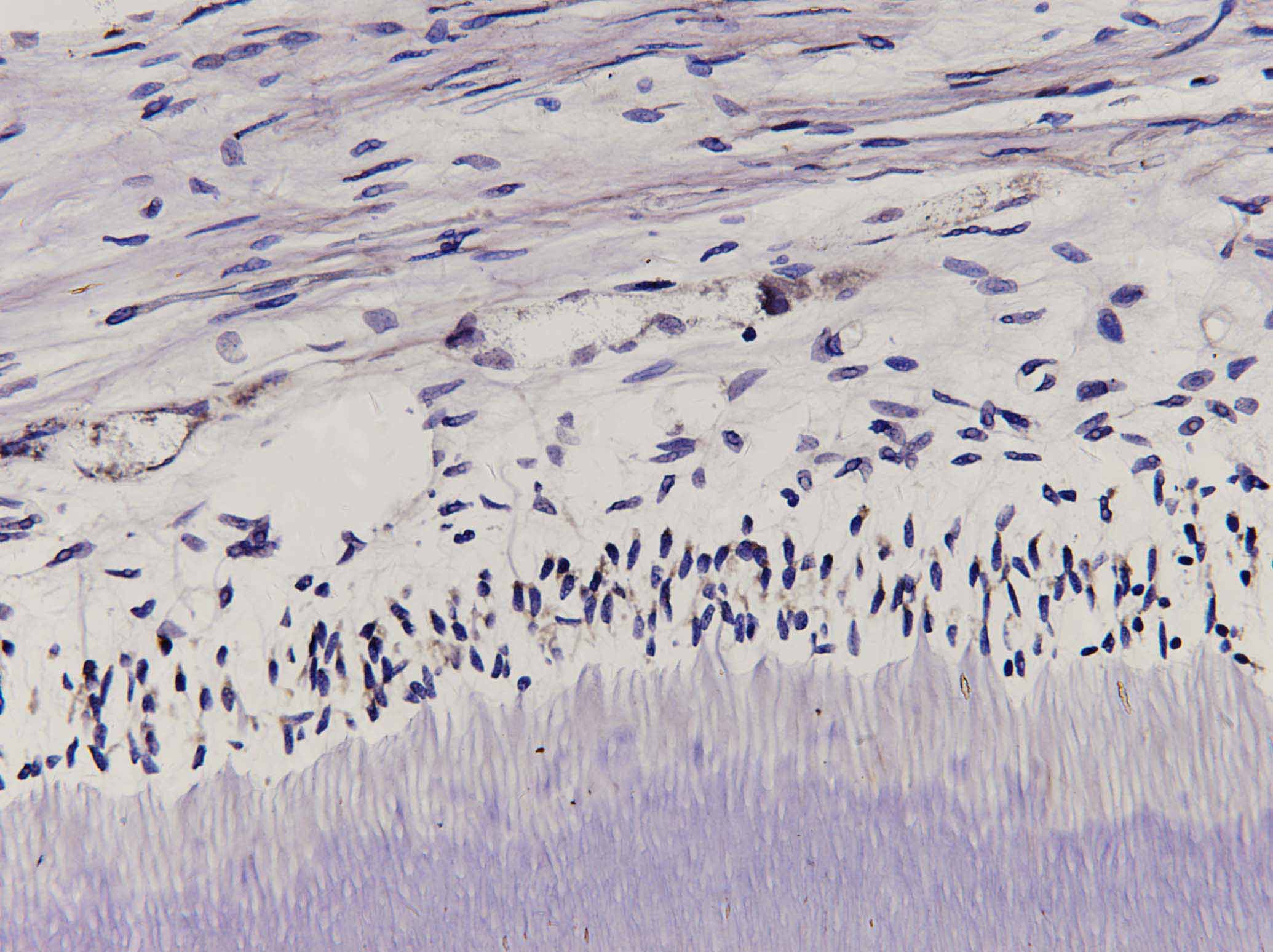

Supplement: Supplemental Information 1 — Immunohistochemical staining for sclerostin in young and senescent dental pulps. [file peerj-06-5808-s001.zip › Senescent/Image_9245.jpg]

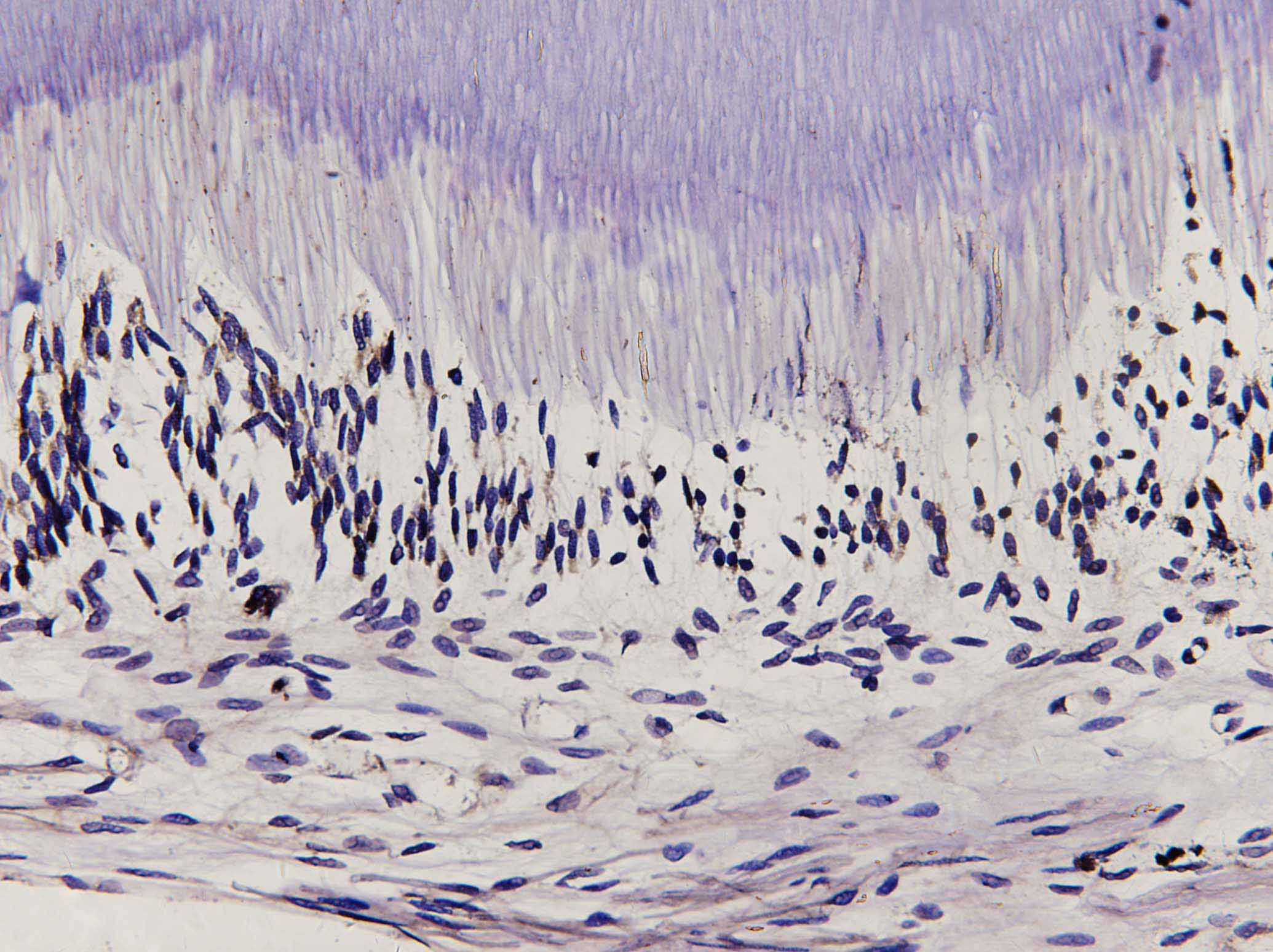

Supplement: Supplemental Information 1 — Immunohistochemical staining for sclerostin in young and senescent dental pulps. [file peerj-06-5808-s001.zip › Senescent/Image_9246.jpg]

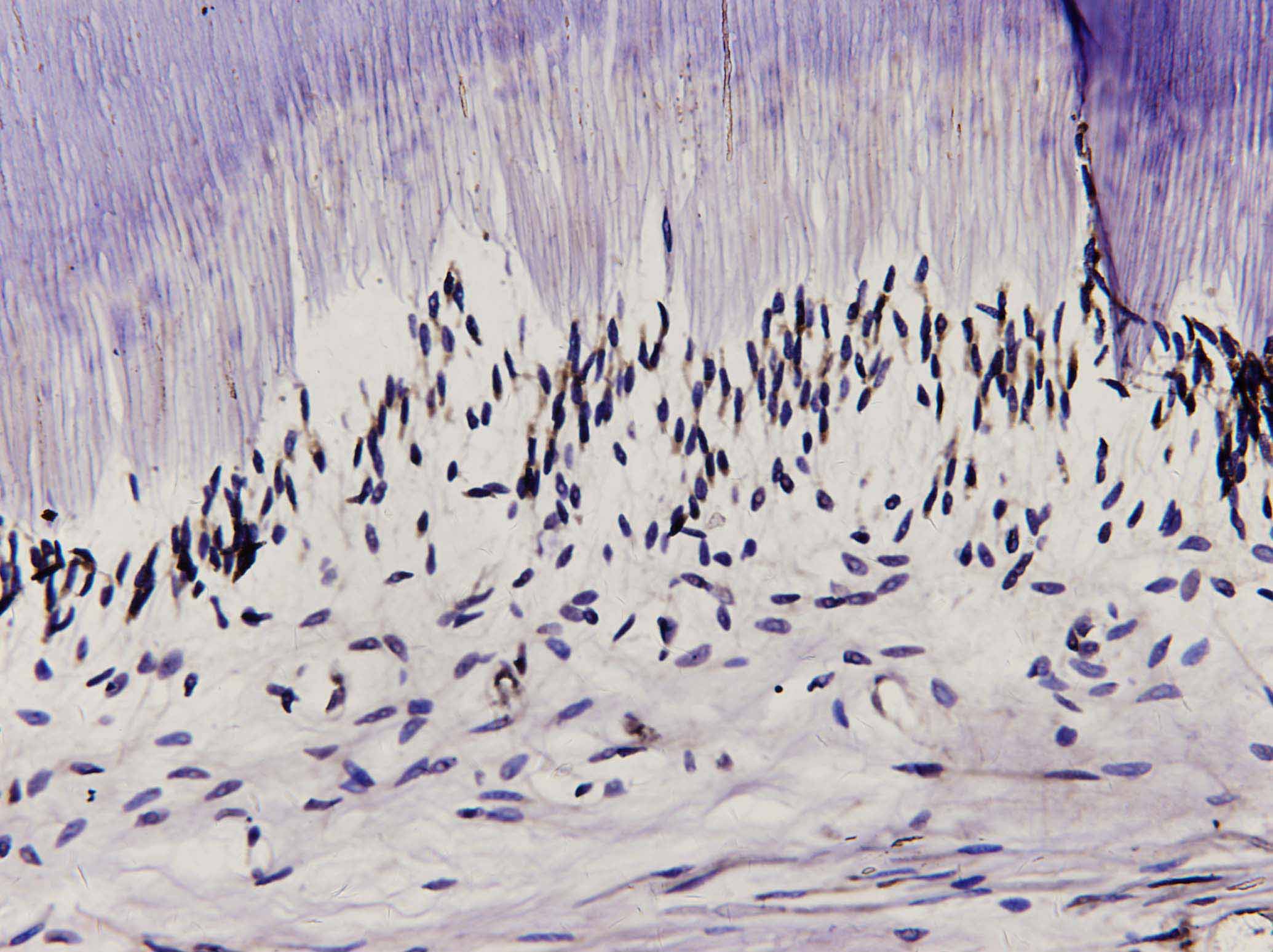

Supplement: Supplemental Information 1 — Immunohistochemical staining for sclerostin in young and senescent dental pulps. [file peerj-06-5808-s001.zip › Senescent/Image_9247.jpg]

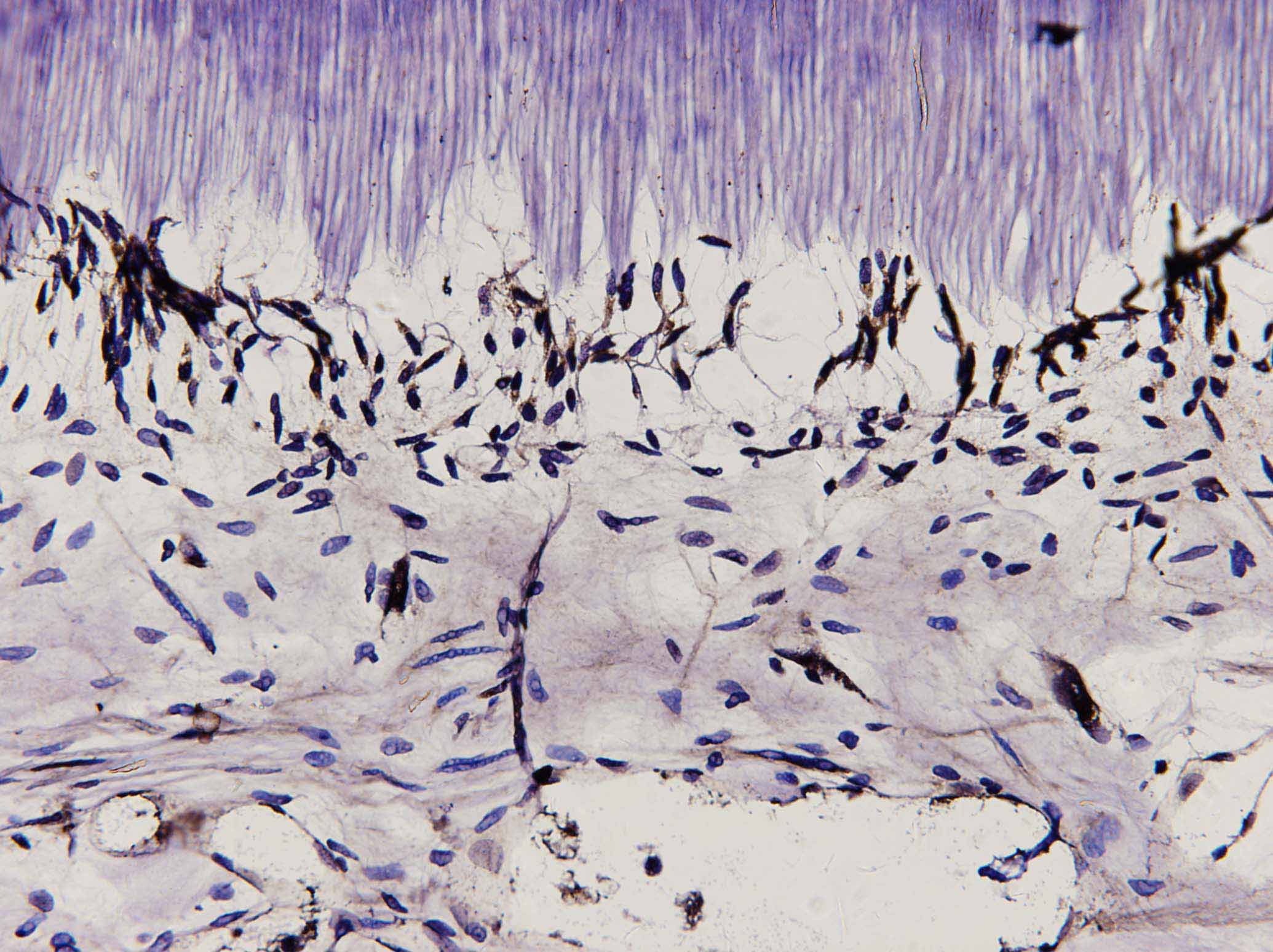

Supplement: Supplemental Information 1 — Immunohistochemical staining for sclerostin in young and senescent dental pulps. [file peerj-06-5808-s001.zip › Senescent/Image_9248.jpg]

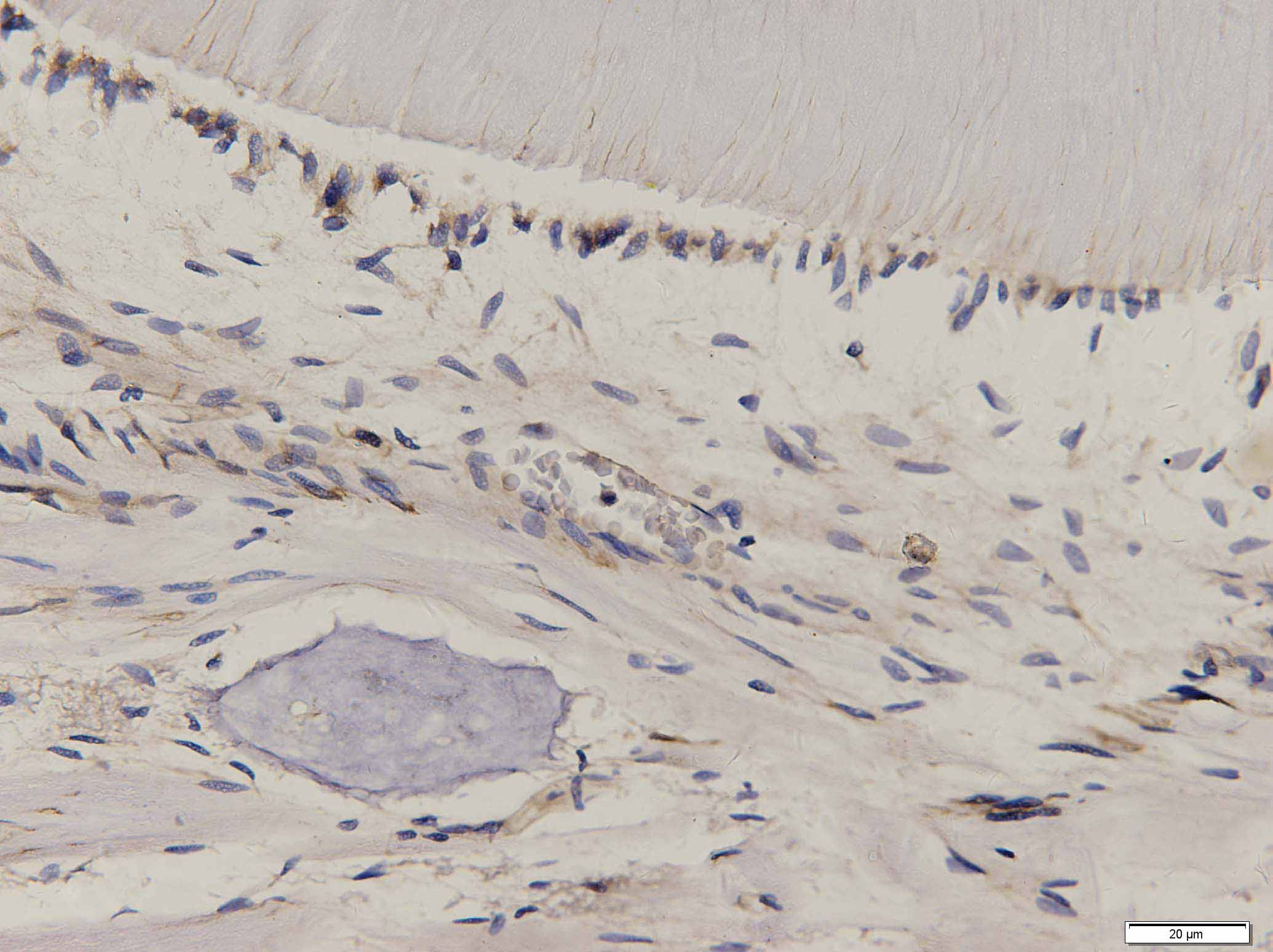

Supplement: Supplemental Information 1 — Immunohistochemical staining for sclerostin in young and senescent dental pulps. [file peerj-06-5808-s001.zip › Senescent/Image_9256.jpg]

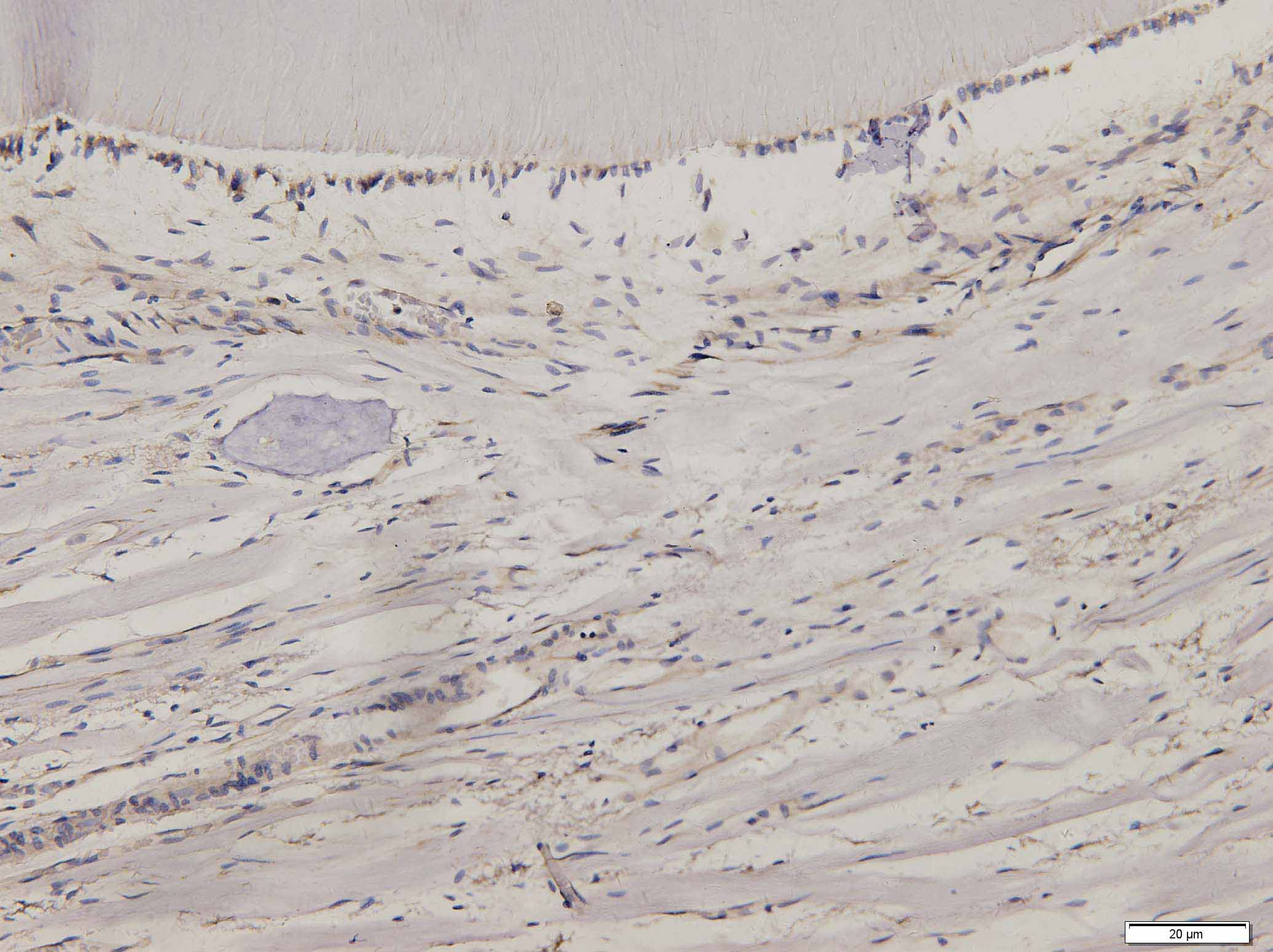

Supplement: Supplemental Information 1 — Immunohistochemical staining for sclerostin in young and senescent dental pulps. [file peerj-06-5808-s001.zip › Senescent/Image_9257.jpg]

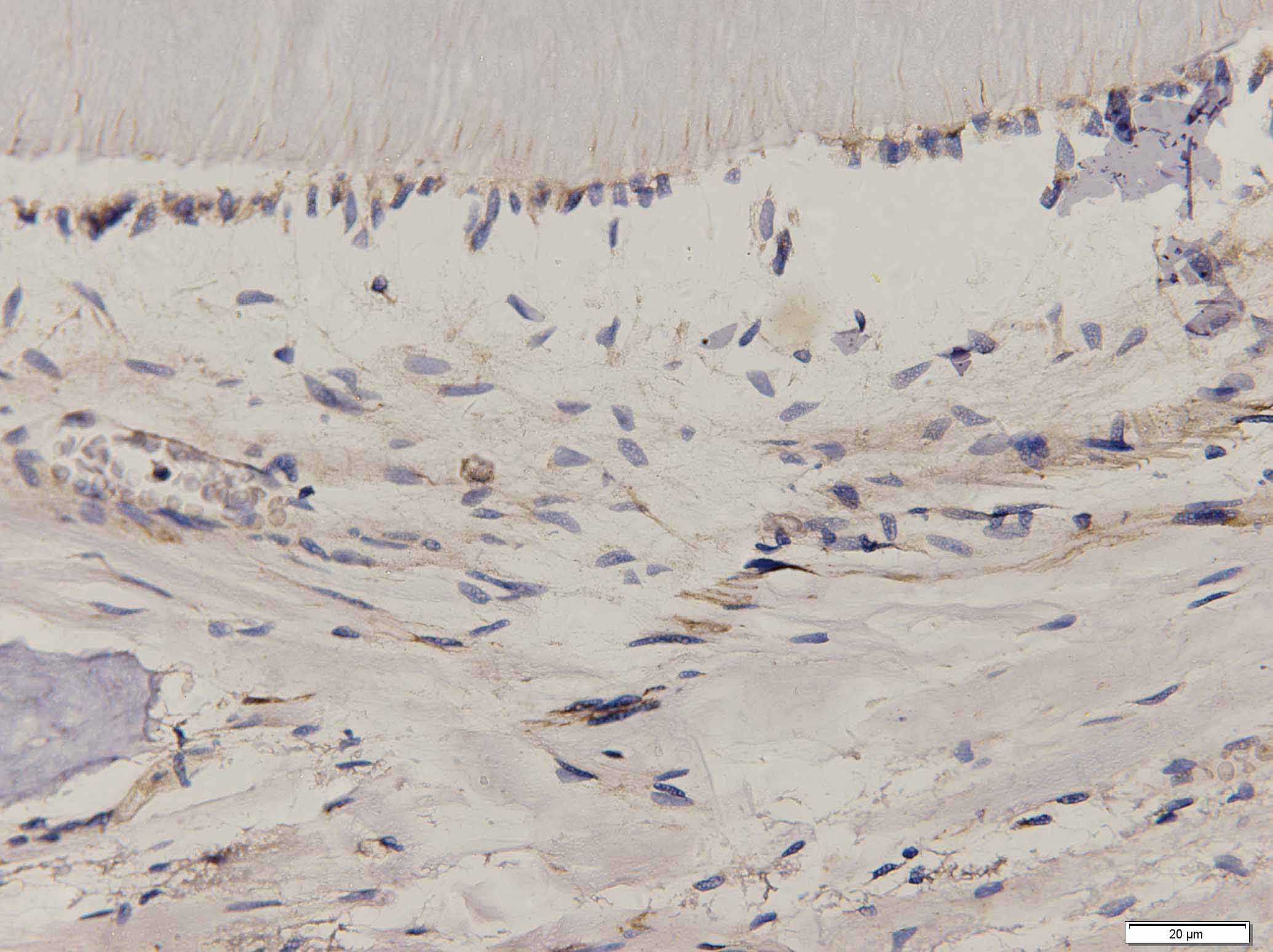

Supplement: Supplemental Information 1 — Immunohistochemical staining for sclerostin in young and senescent dental pulps. [file peerj-06-5808-s001.zip › Senescent/Image_9258.jpg]

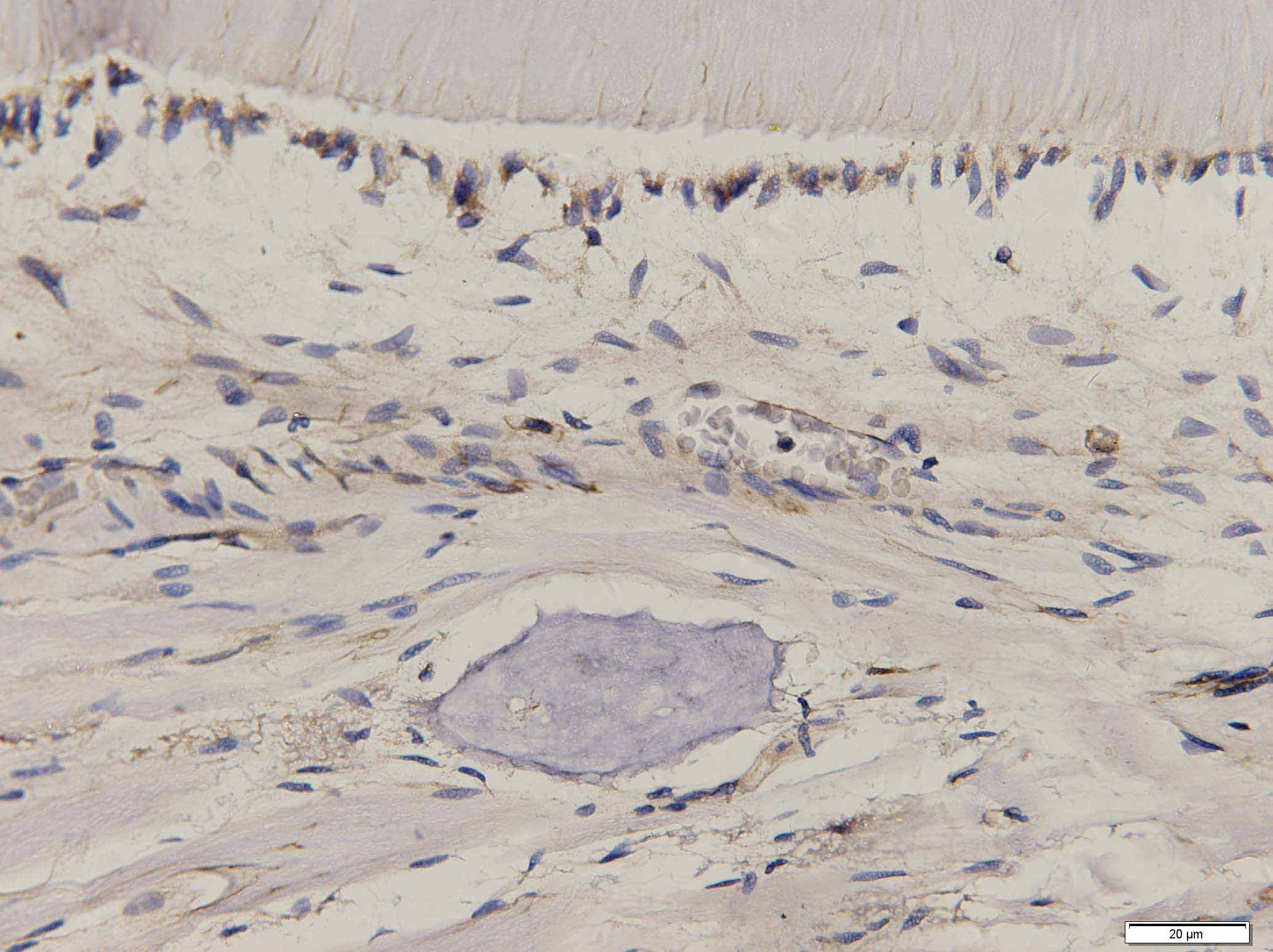

Supplement: Supplemental Information 1 — Immunohistochemical staining for sclerostin in young and senescent dental pulps. [file peerj-06-5808-s001.zip › Senescent/Image_9259.jpg]

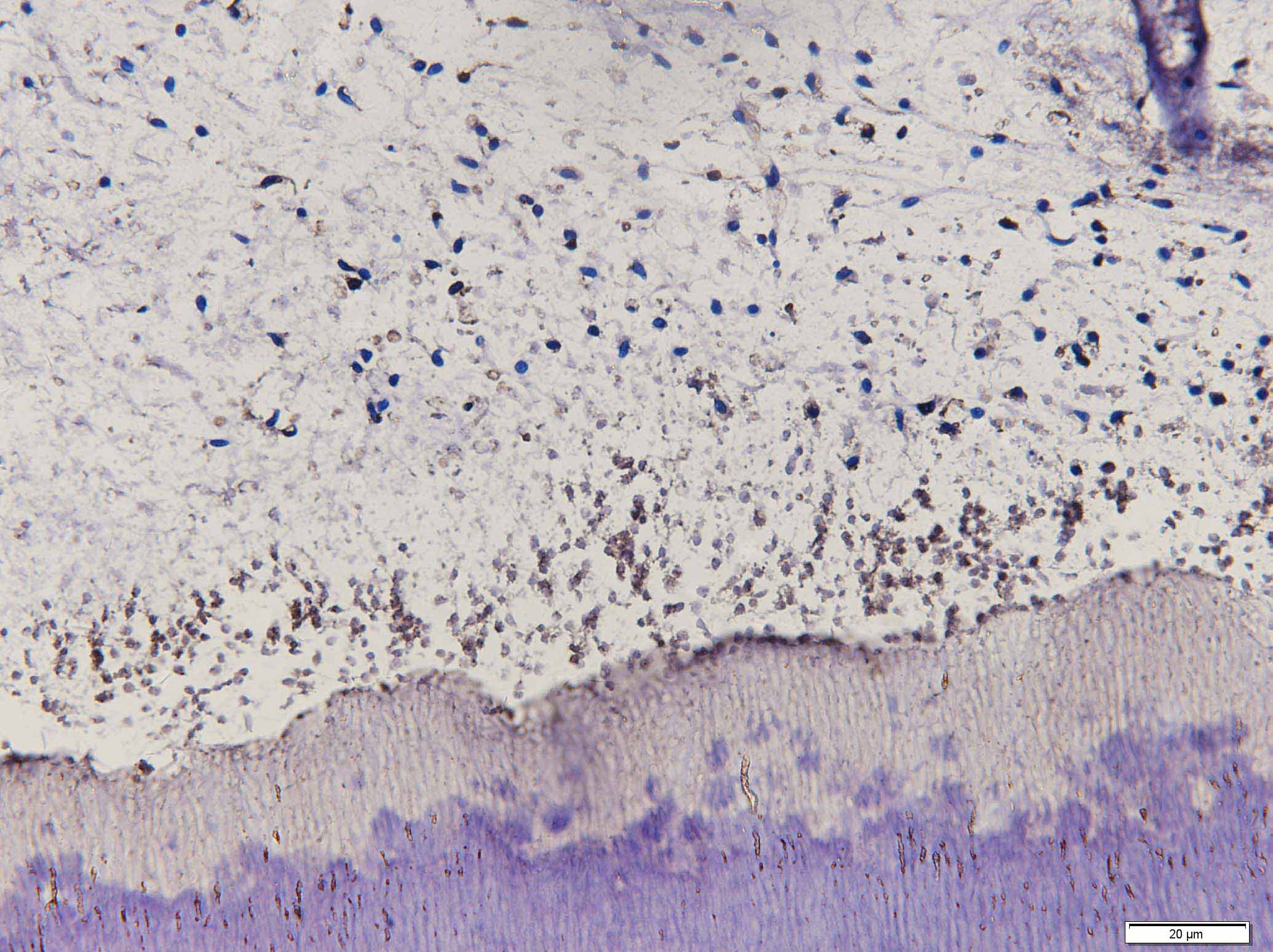

Supplement: Supplemental Information 1 — Immunohistochemical staining for sclerostin in young and senescent dental pulps. [file peerj-06-5808-s001.zip › Senescent/Image_9269.jpg]

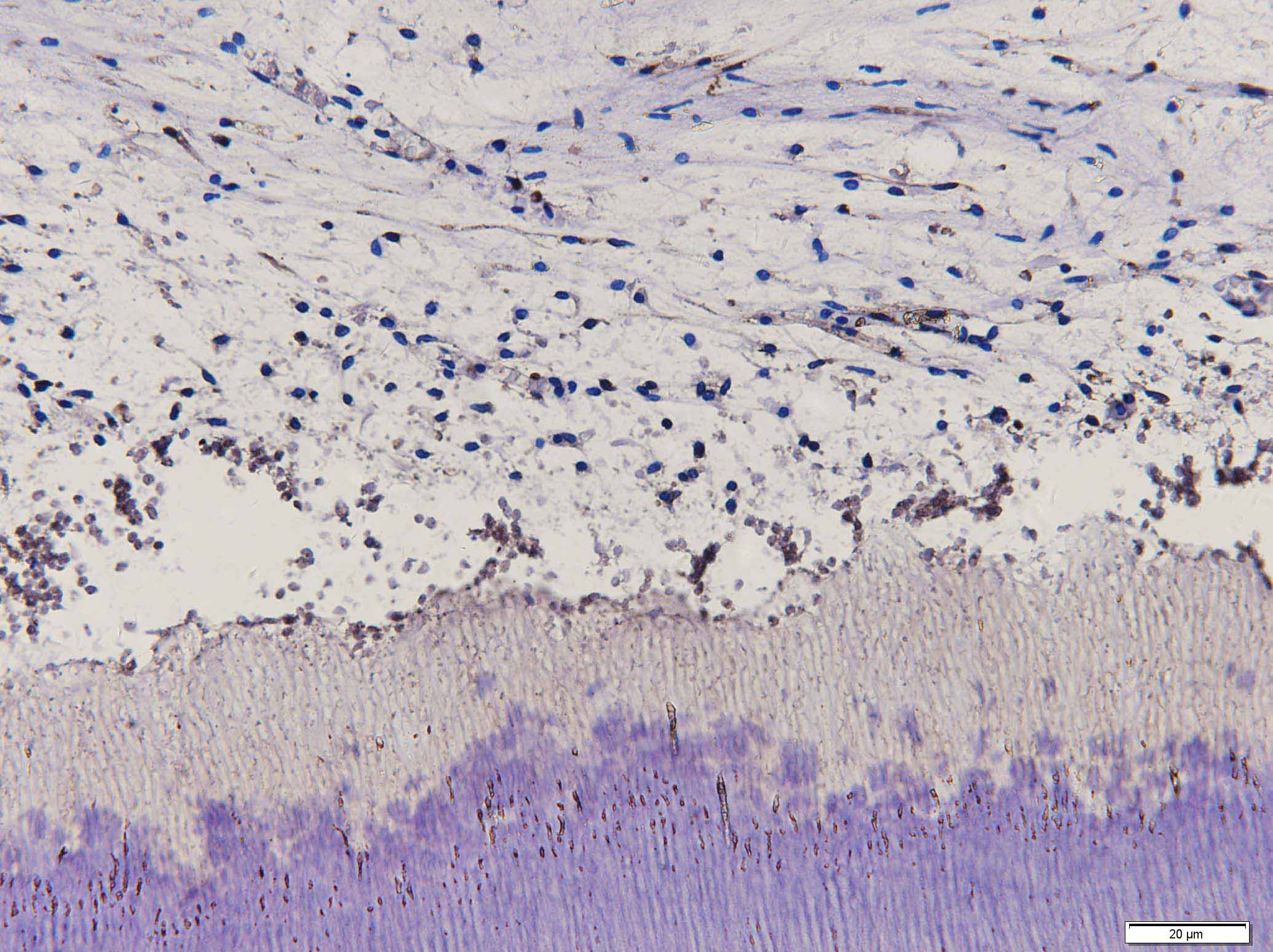

Supplement: Supplemental Information 1 — Immunohistochemical staining for sclerostin in young and senescent dental pulps. [file peerj-06-5808-s001.zip › Senescent/Image_9270.jpg]

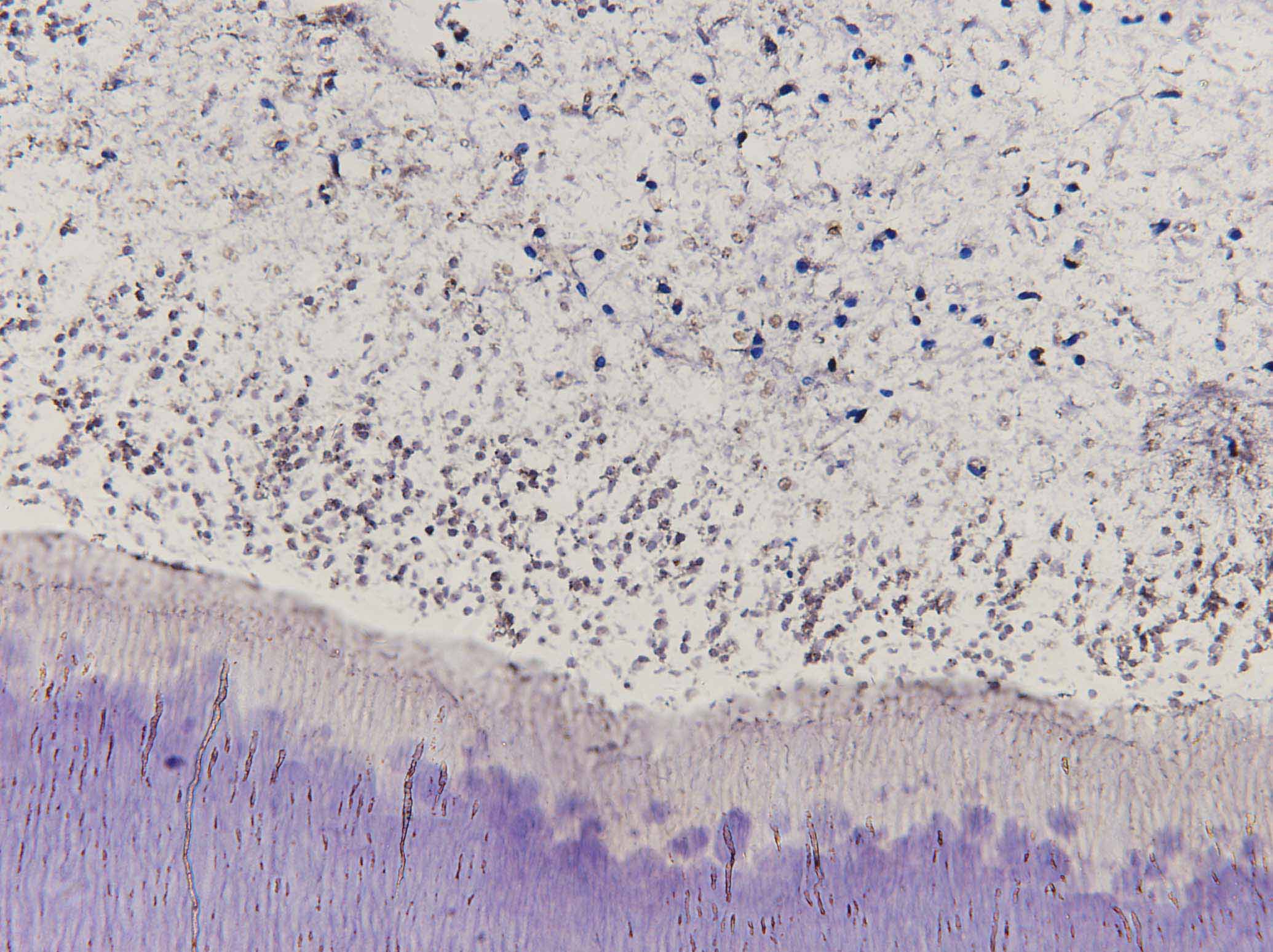

Supplement: Supplemental Information 1 — Immunohistochemical staining for sclerostin in young and senescent dental pulps. [file peerj-06-5808-s001.zip › Senescent/Image_9271.jpg]

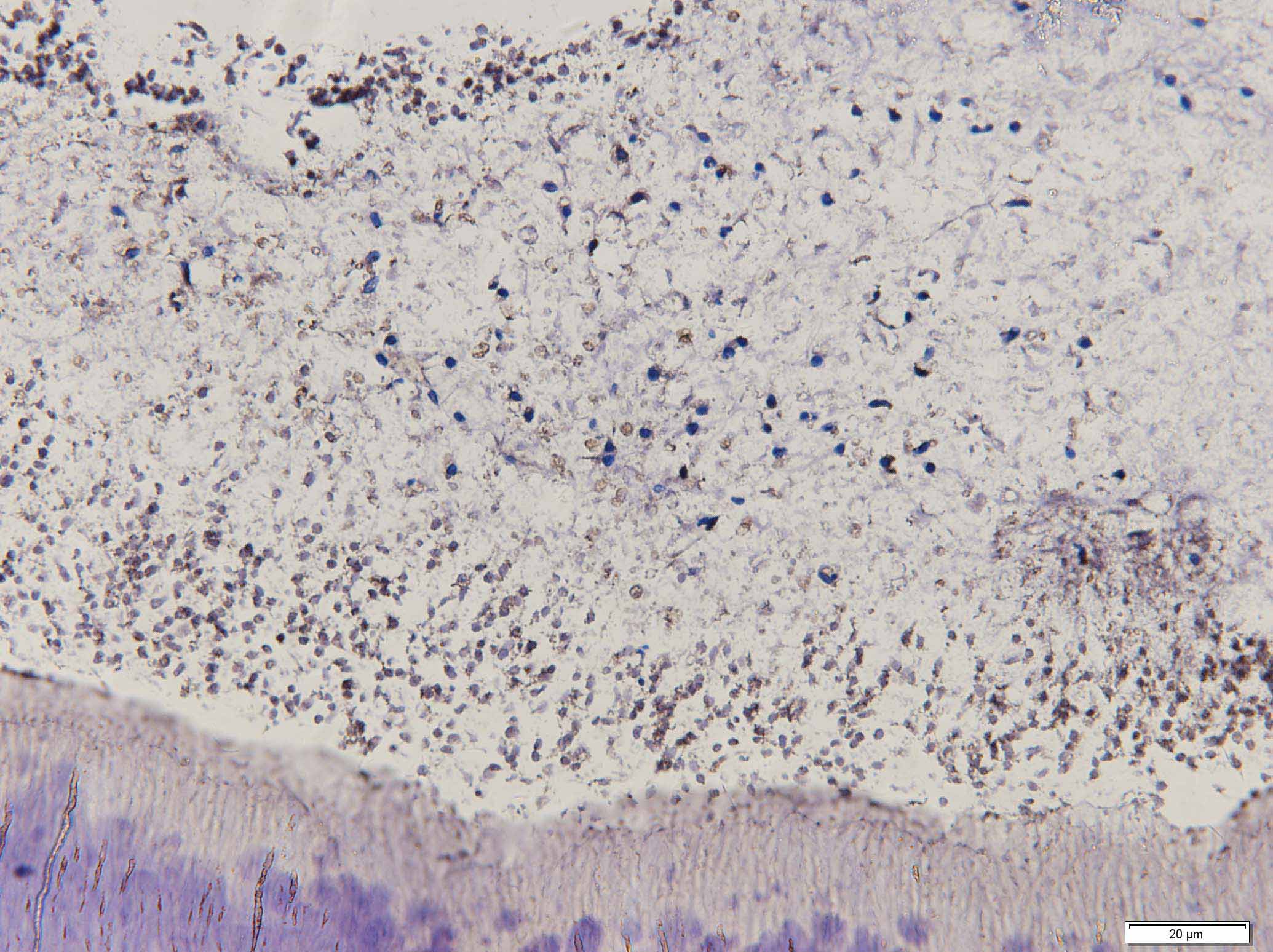

Supplement: Supplemental Information 1 — Immunohistochemical staining for sclerostin in young and senescent dental pulps. [file peerj-06-5808-s001.zip › Senescent/Image_9272.jpg]

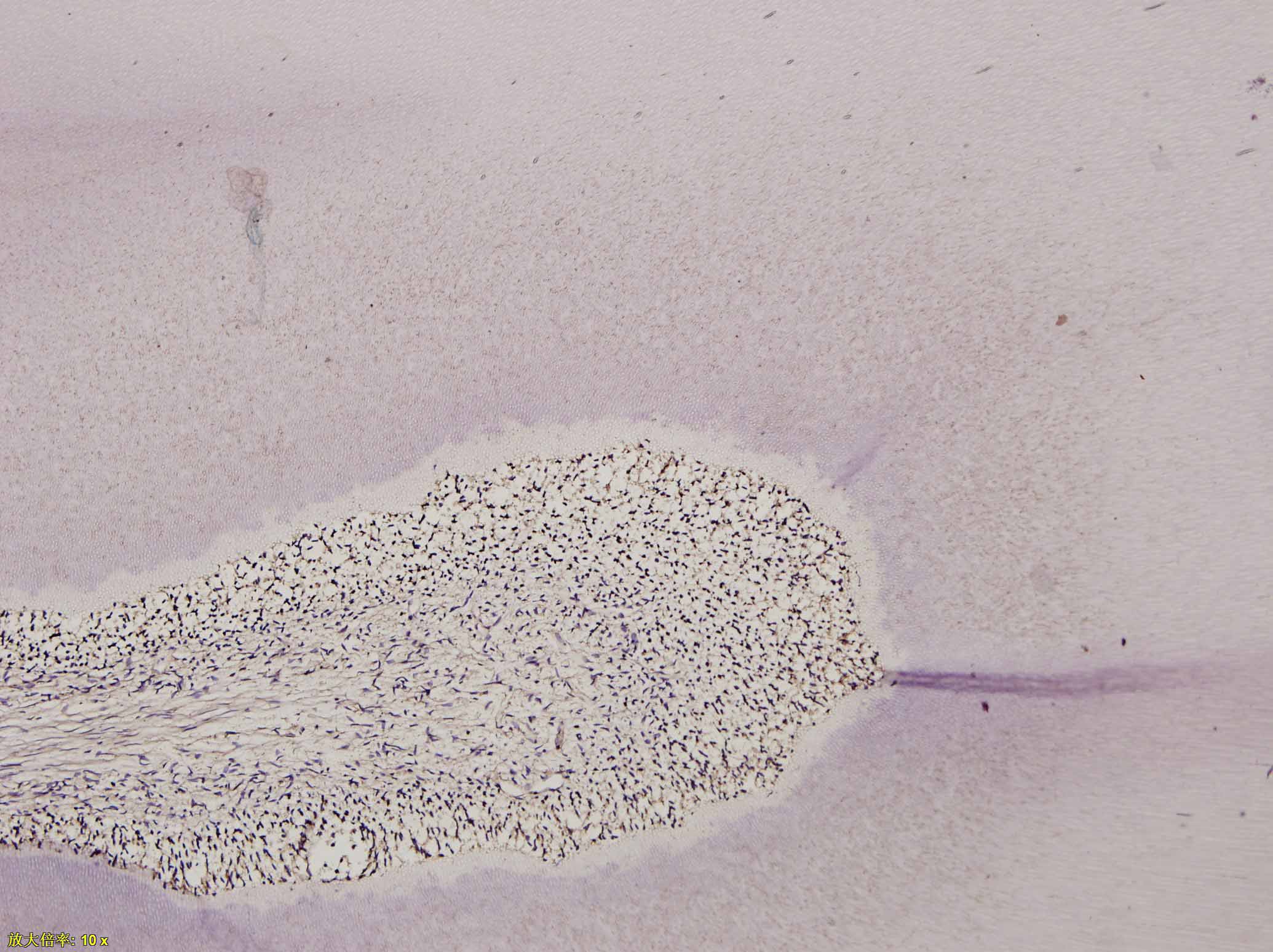

Supplement: Supplemental Information 1 — Immunohistochemical staining for sclerostin in young and senescent dental pulps. [file peerj-06-5808-s001.zip › Senescent/图像_8391.jpg]

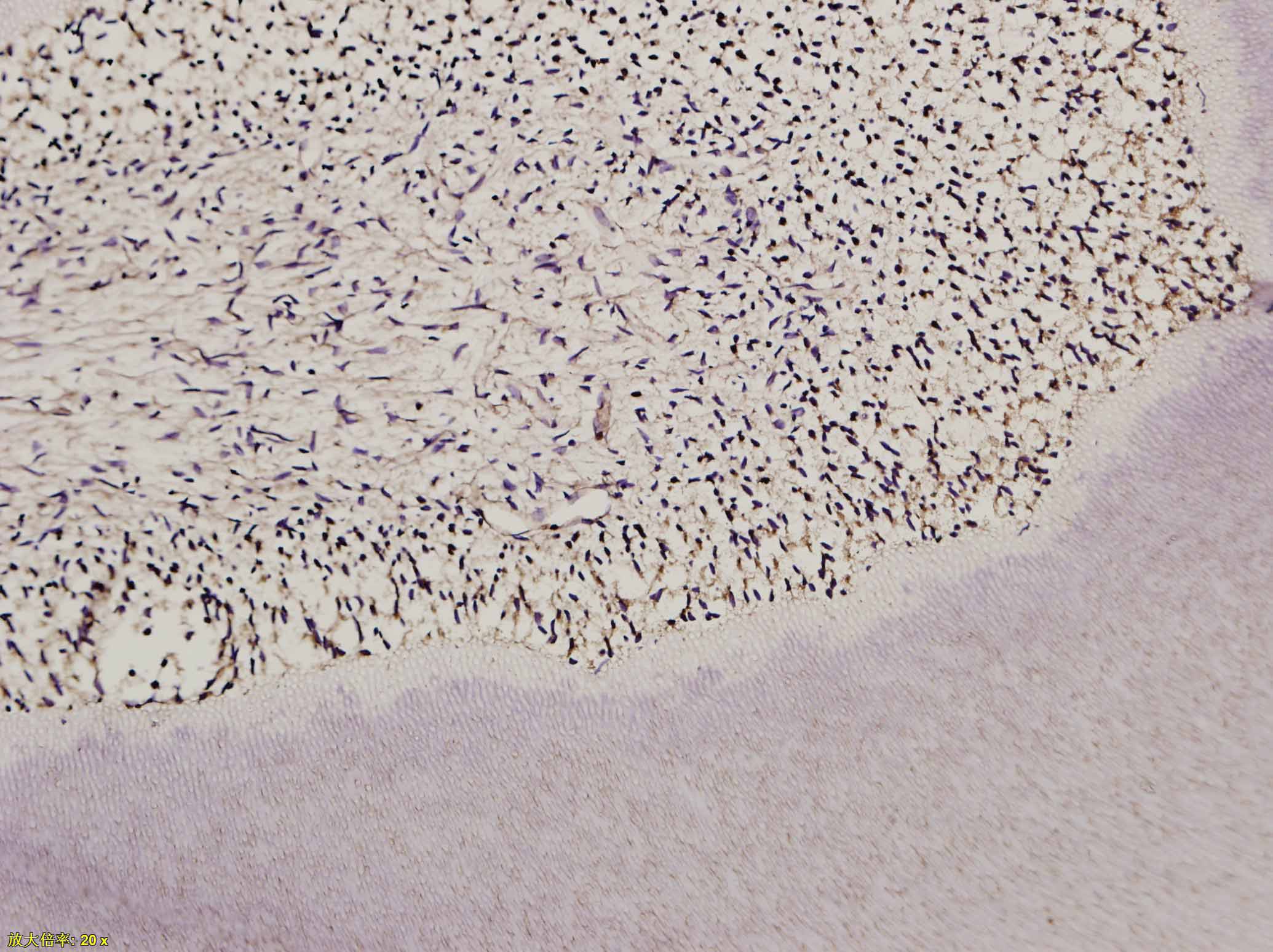

Supplement: Supplemental Information 1 — Immunohistochemical staining for sclerostin in young and senescent dental pulps. [file peerj-06-5808-s001.zip › Senescent/图像_8392.jpg]

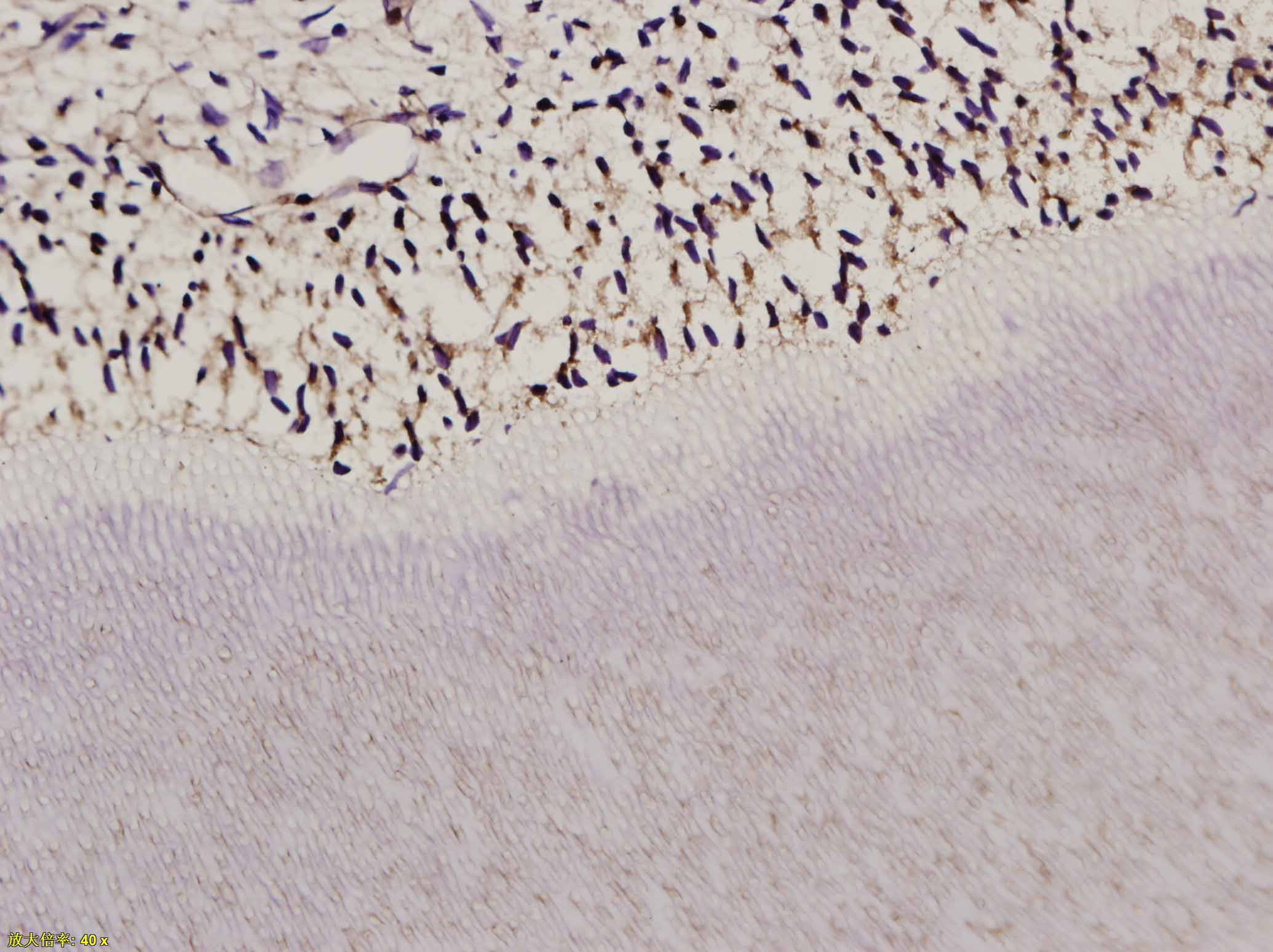

Supplement: Supplemental Information 1 — Immunohistochemical staining for sclerostin in young and senescent dental pulps. [file peerj-06-5808-s001.zip › Senescent/图像_8393.jpg]

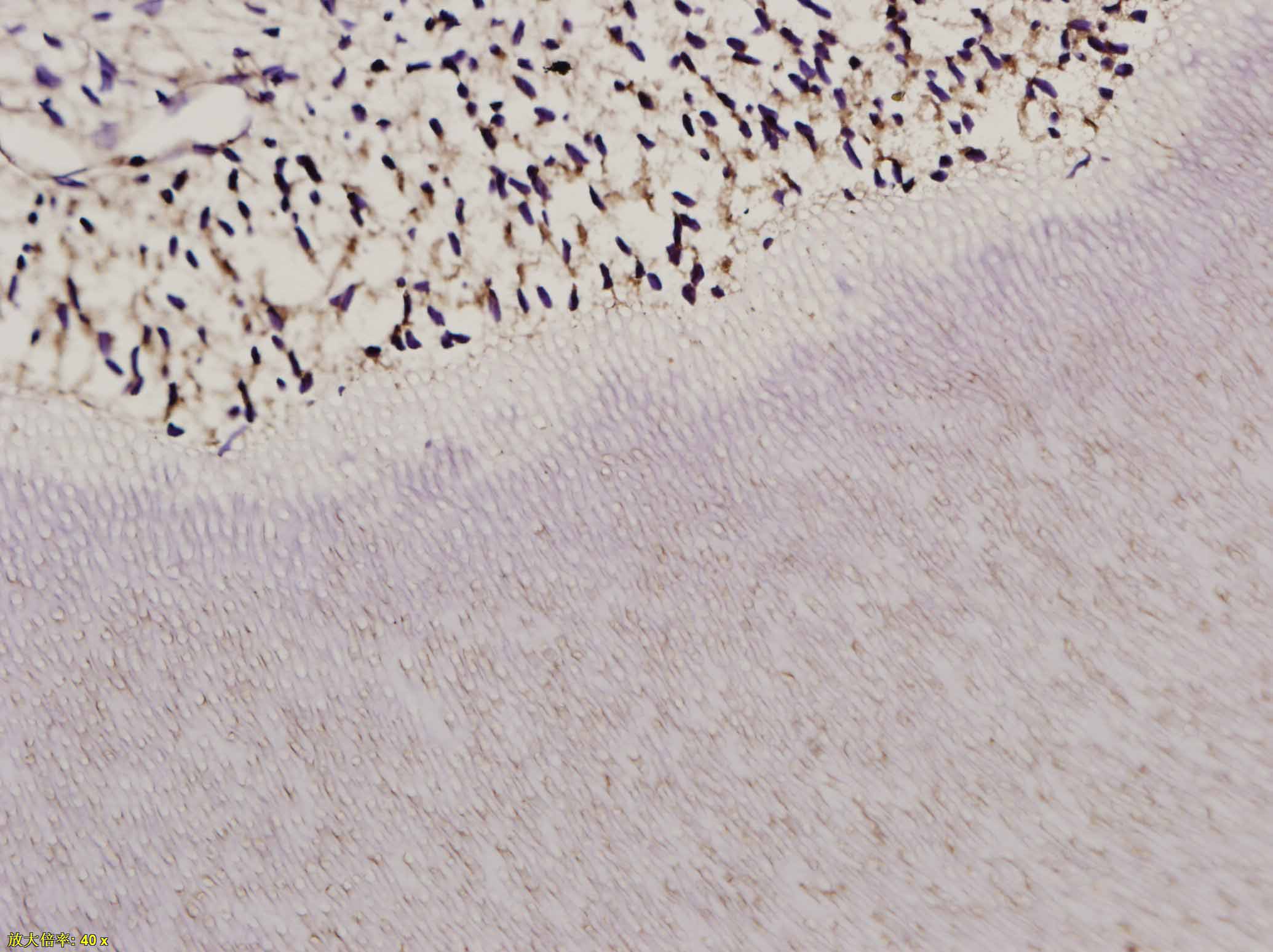

Supplement: Supplemental Information 1 — Immunohistochemical staining for sclerostin in young and senescent dental pulps. [file peerj-06-5808-s001.zip › Senescent/图像_8394.jpg]

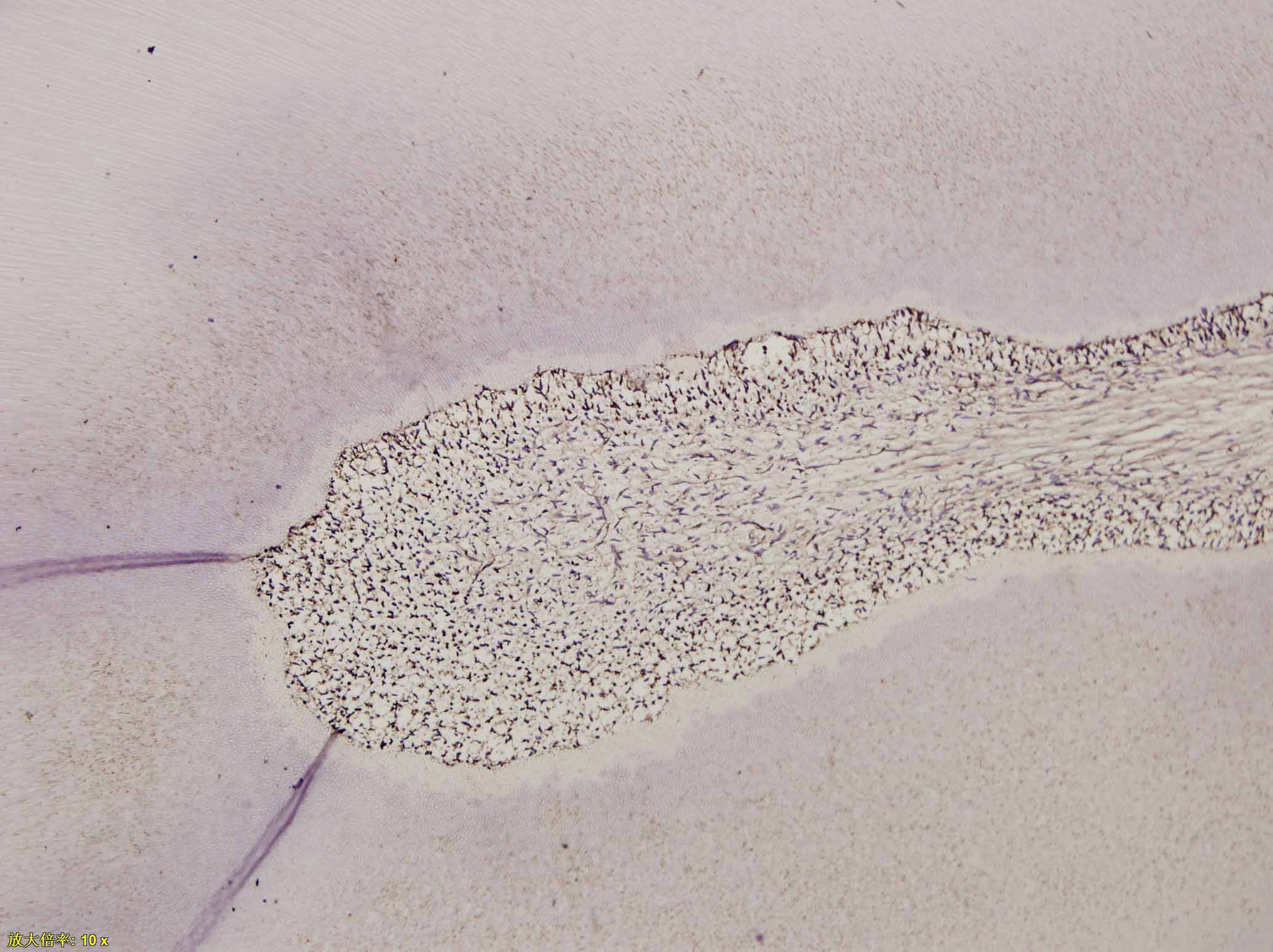

Supplement: Supplemental Information 1 — Immunohistochemical staining for sclerostin in young and senescent dental pulps. [file peerj-06-5808-s001.zip › Senescent/图像_8401.jpg]

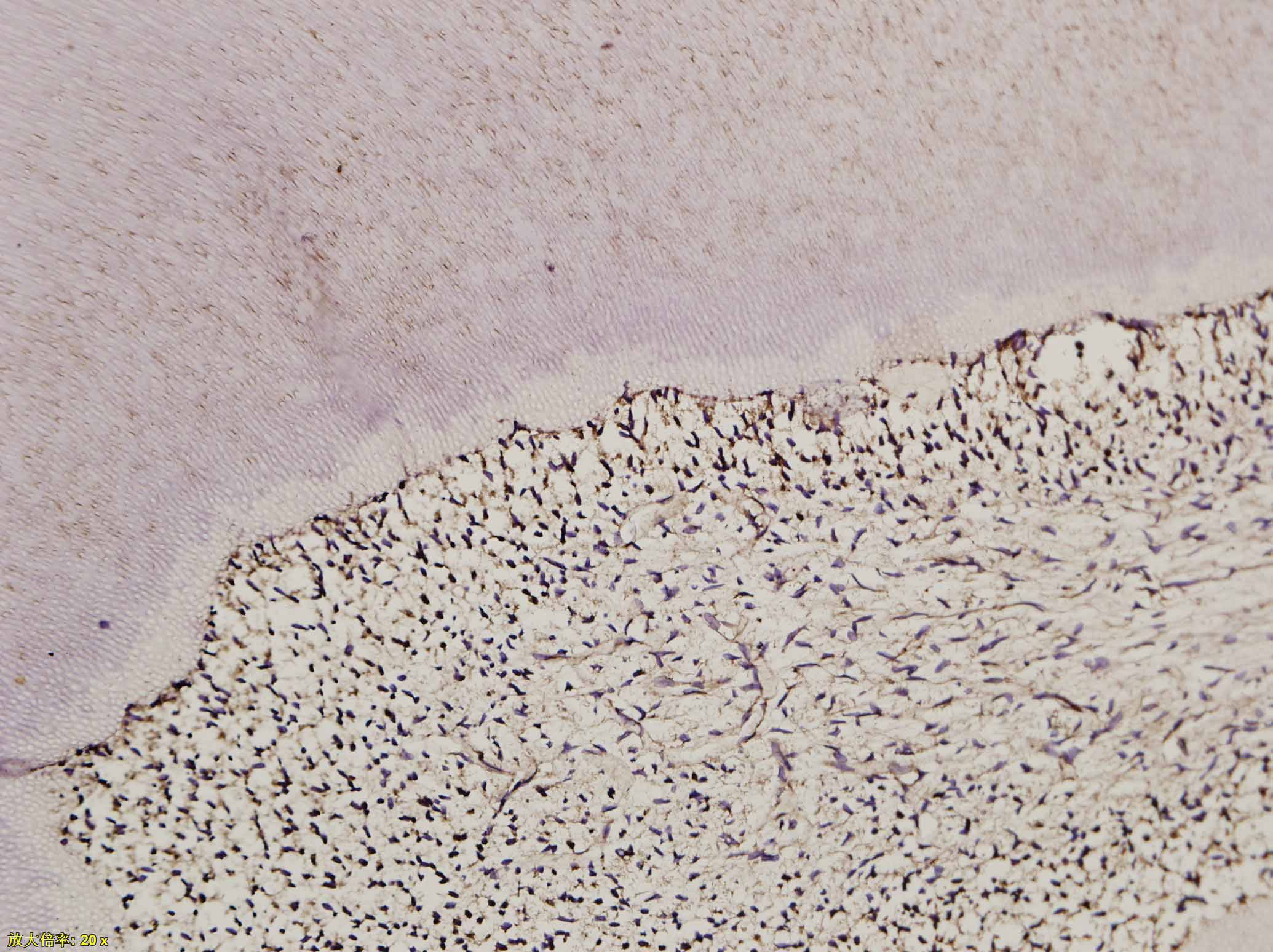

Supplement: Supplemental Information 1 — Immunohistochemical staining for sclerostin in young and senescent dental pulps. [file peerj-06-5808-s001.zip › Senescent/图像_8402.jpg]

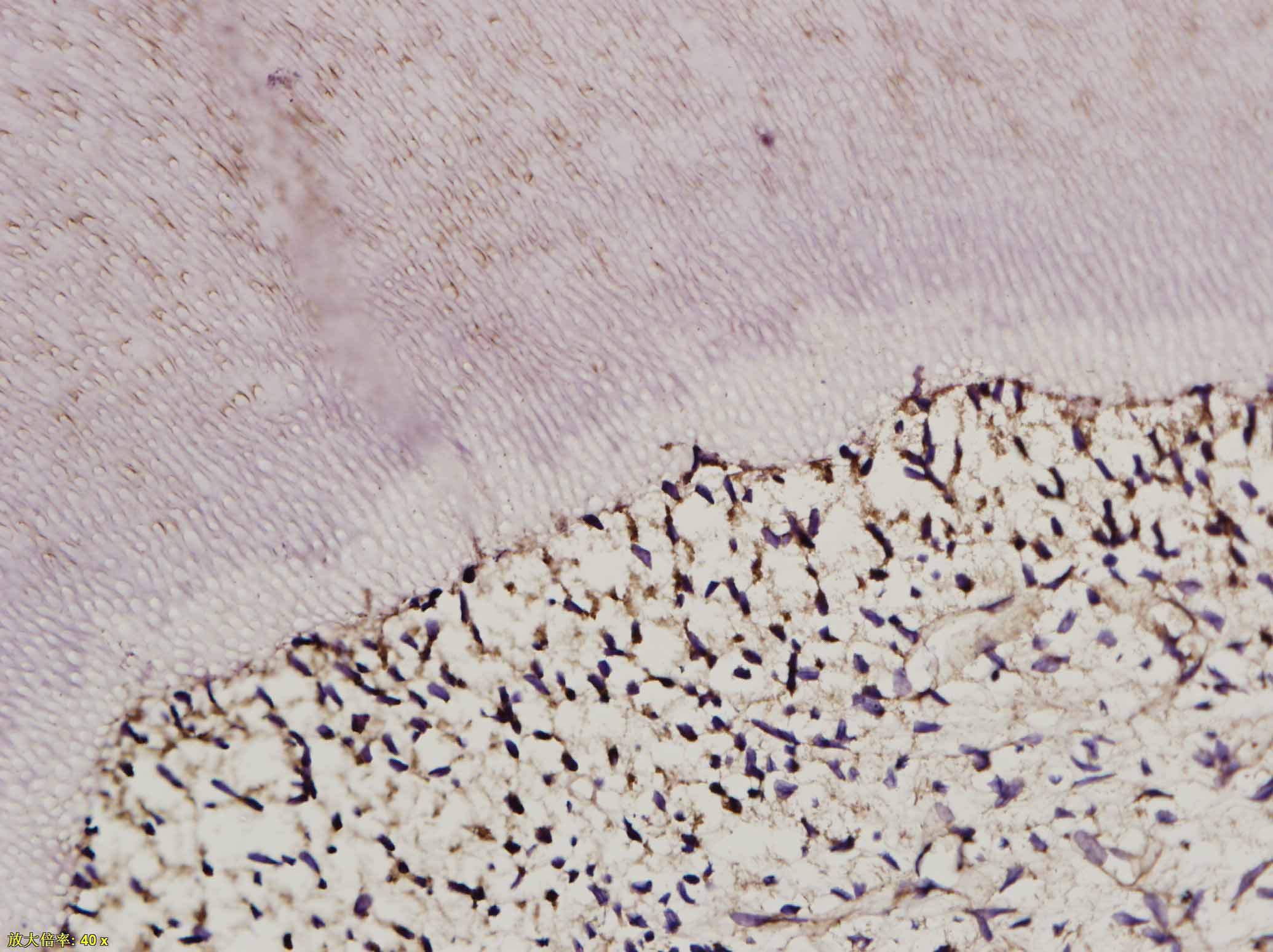

Supplement: Supplemental Information 1 — Immunohistochemical staining for sclerostin in young and senescent dental pulps. [file peerj-06-5808-s001.zip › Senescent/图像_8403.jpg]

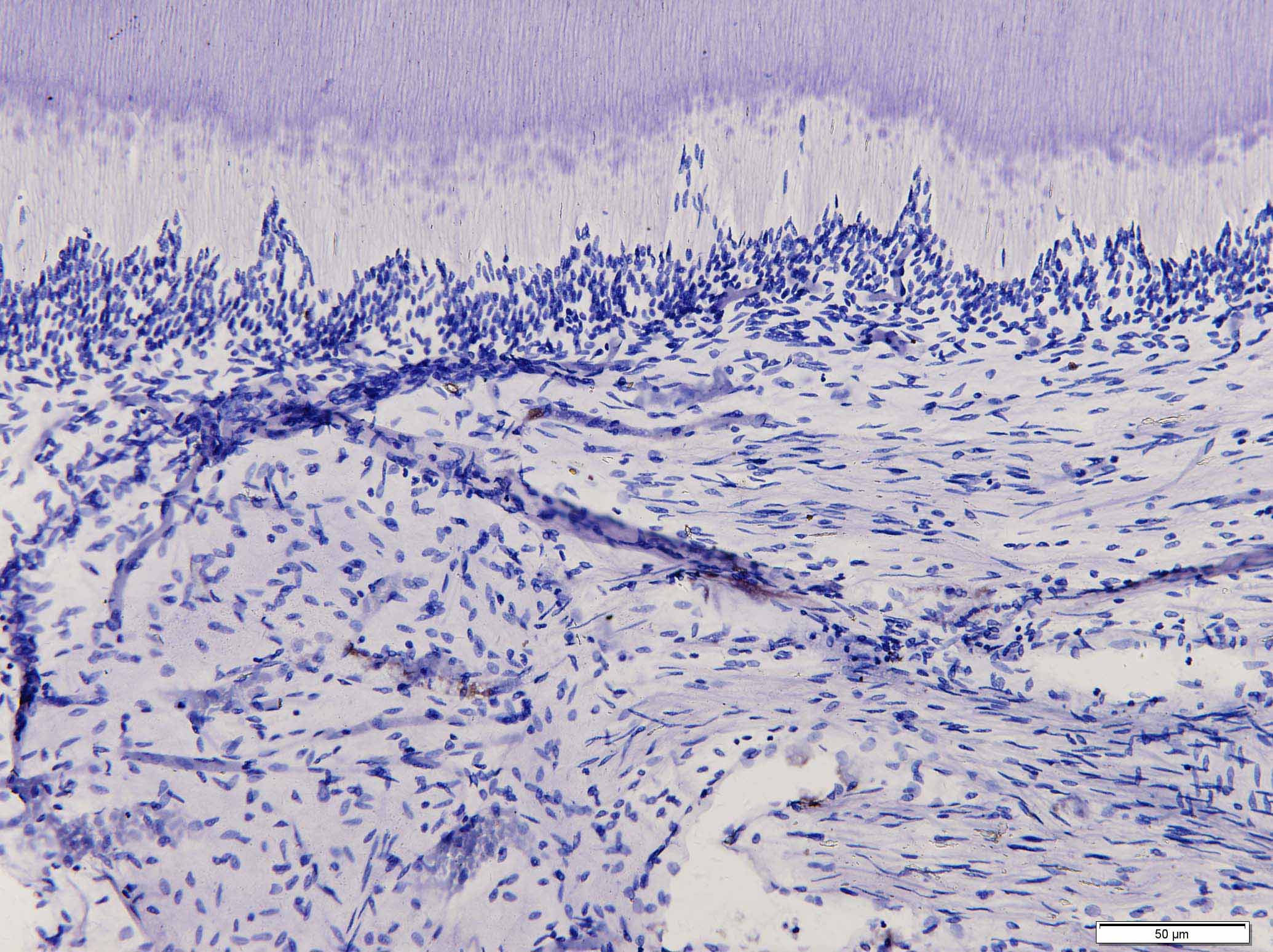

Supplement: Supplemental Information 1 — Immunohistochemical staining for sclerostin in young and senescent dental pulps. [file peerj-06-5808-s001.zip › Young/Image_9261.jpg]

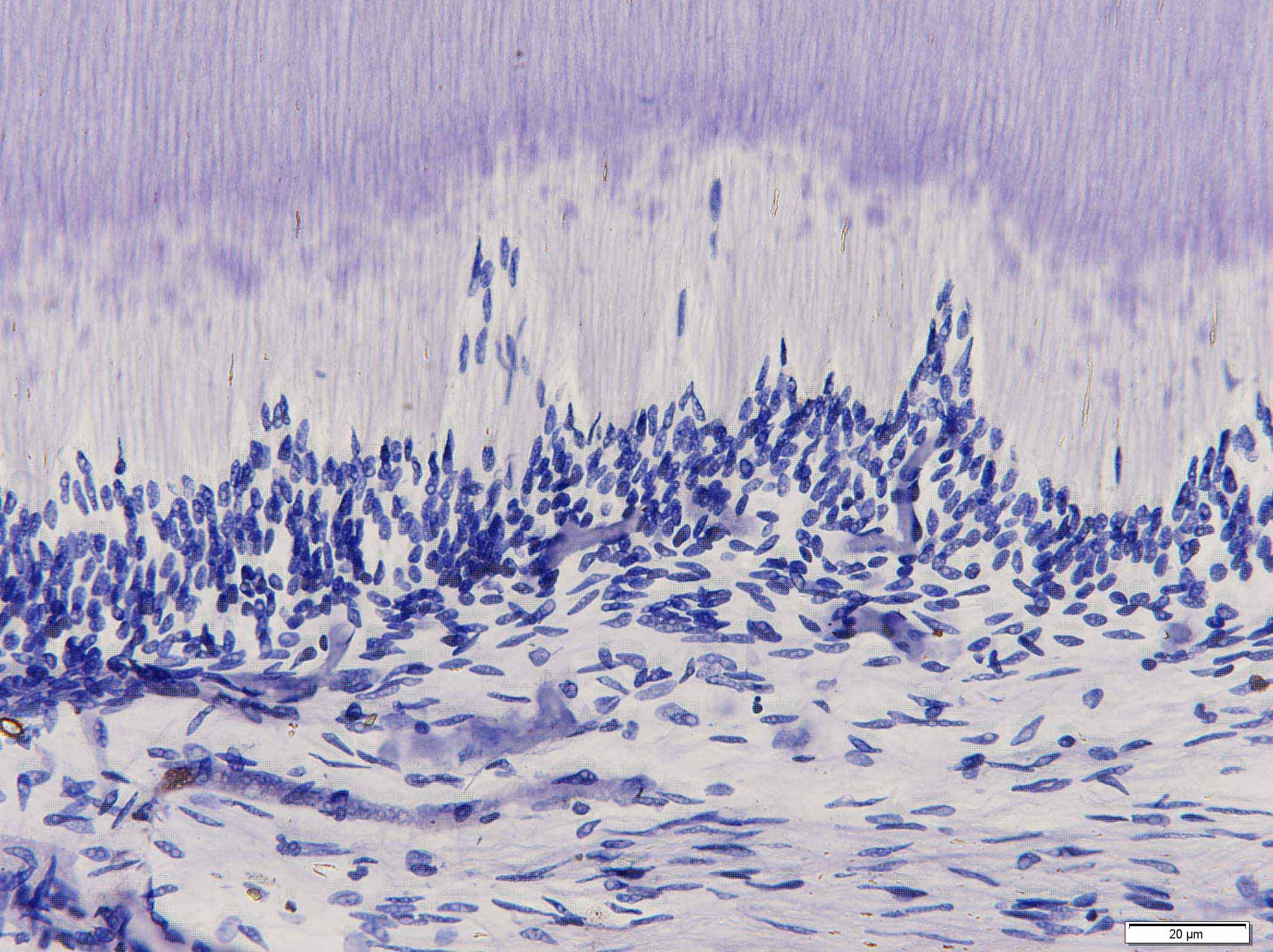

Supplement: Supplemental Information 1 — Immunohistochemical staining for sclerostin in young and senescent dental pulps. [file peerj-06-5808-s001.zip › Young/Image_9263.jpg]

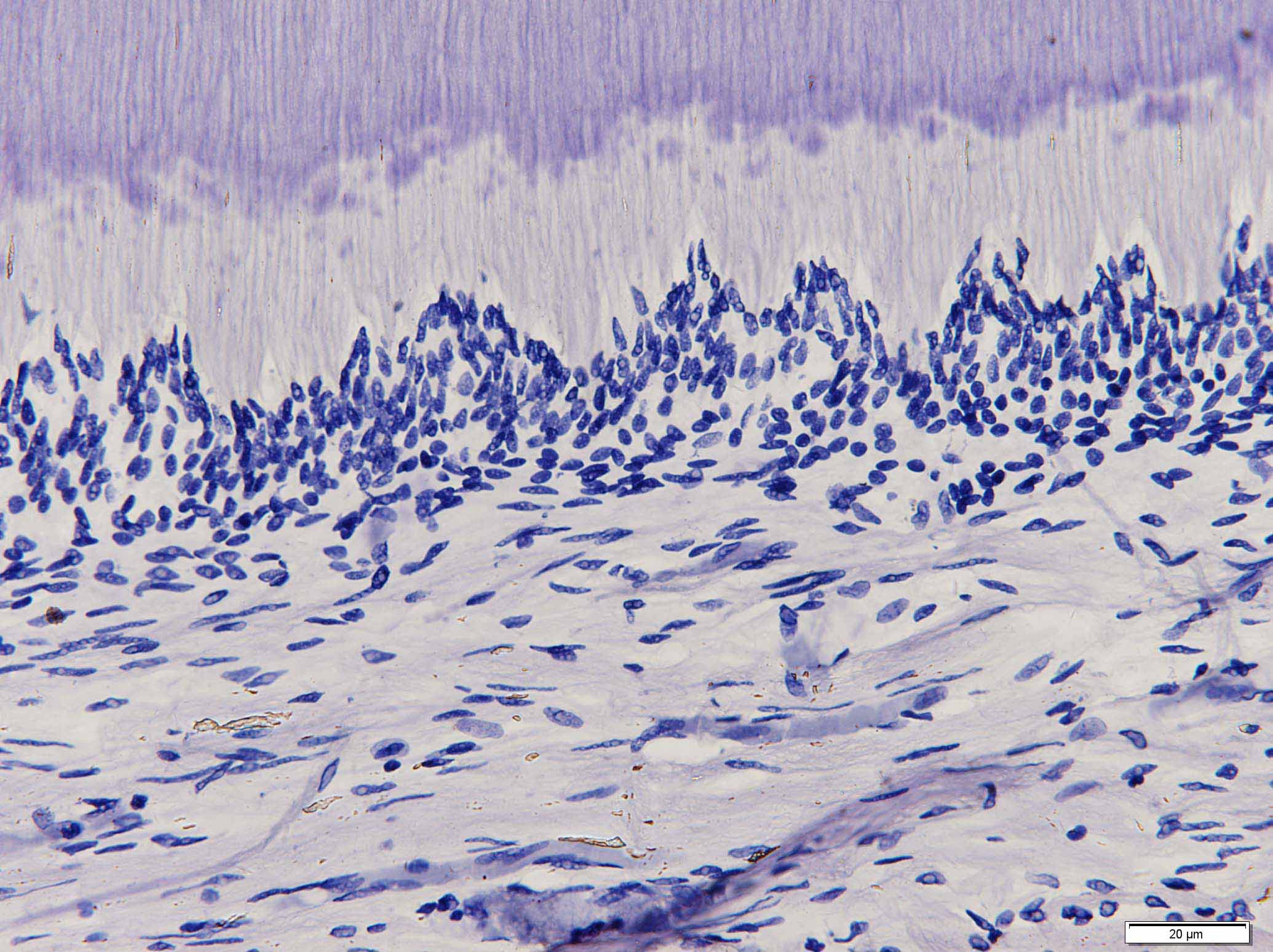

Supplement: Supplemental Information 1 — Immunohistochemical staining for sclerostin in young and senescent dental pulps. [file peerj-06-5808-s001.zip › Young/Image_9264.jpg]

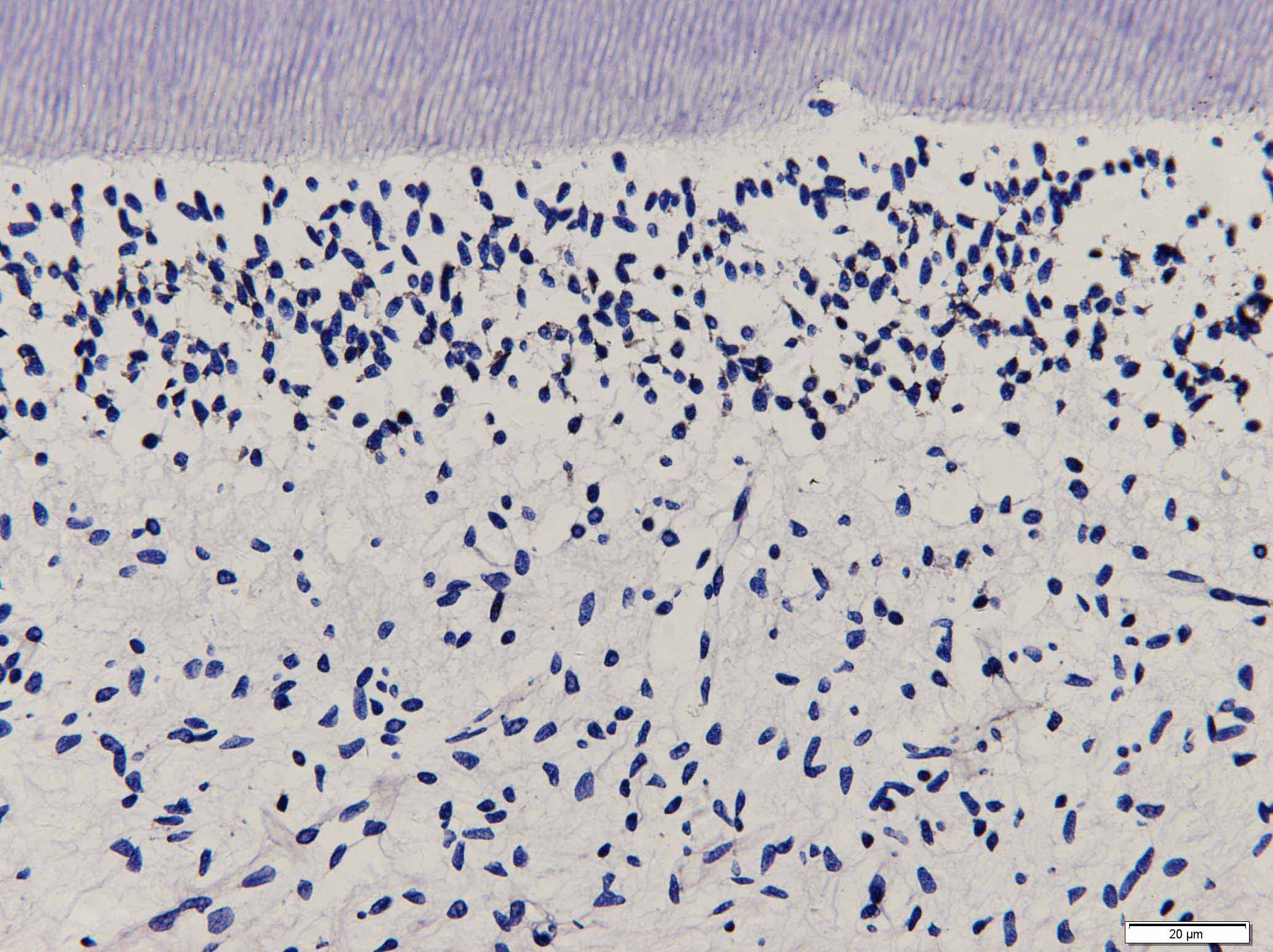

Supplement: Supplemental Information 1 — Immunohistochemical staining for sclerostin in young and senescent dental pulps. [file peerj-06-5808-s001.zip › Young/Image_9278.jpg]

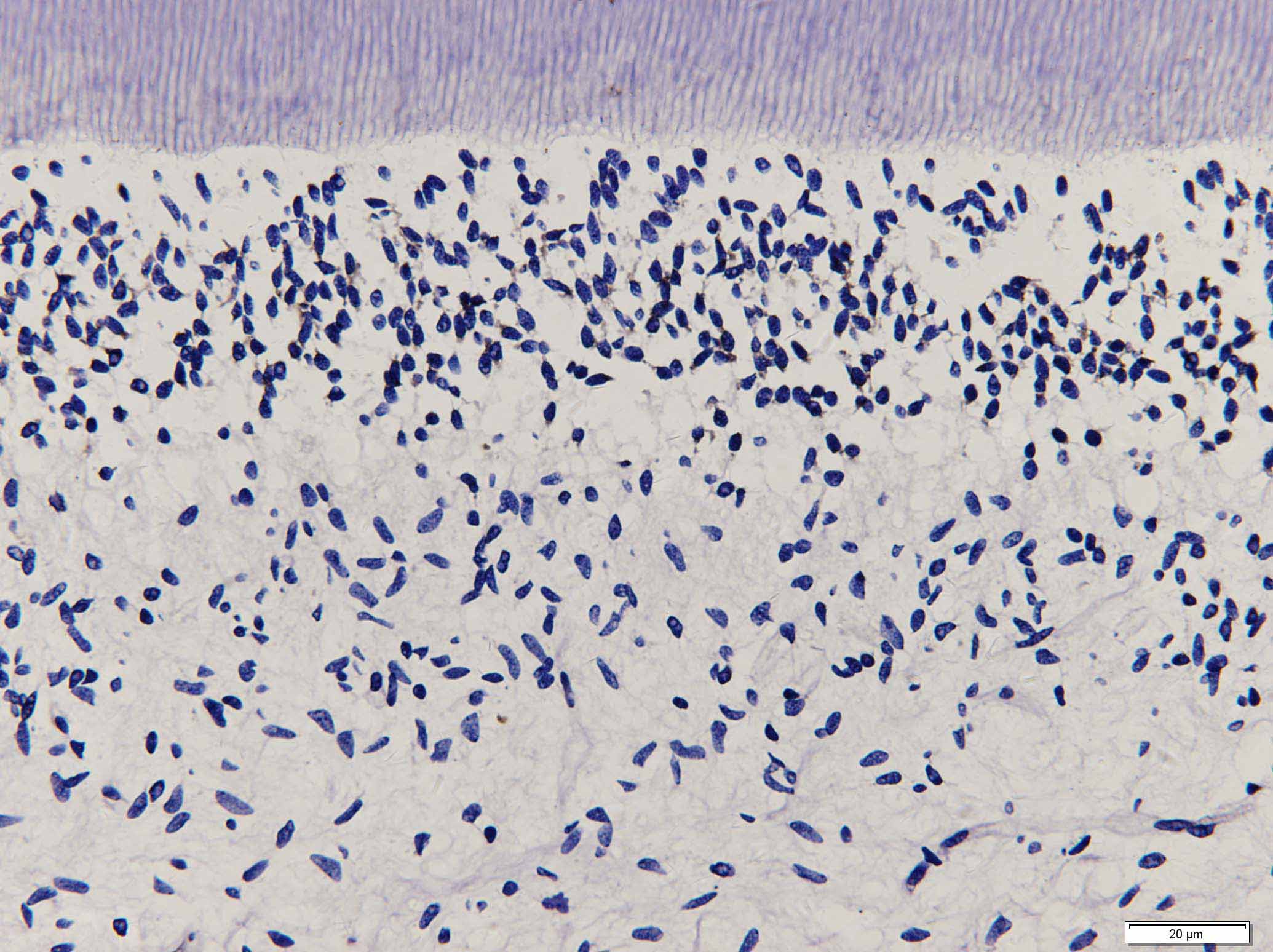

Supplement: Supplemental Information 1 — Immunohistochemical staining for sclerostin in young and senescent dental pulps. [file peerj-06-5808-s001.zip › Young/Image_9279.jpg]

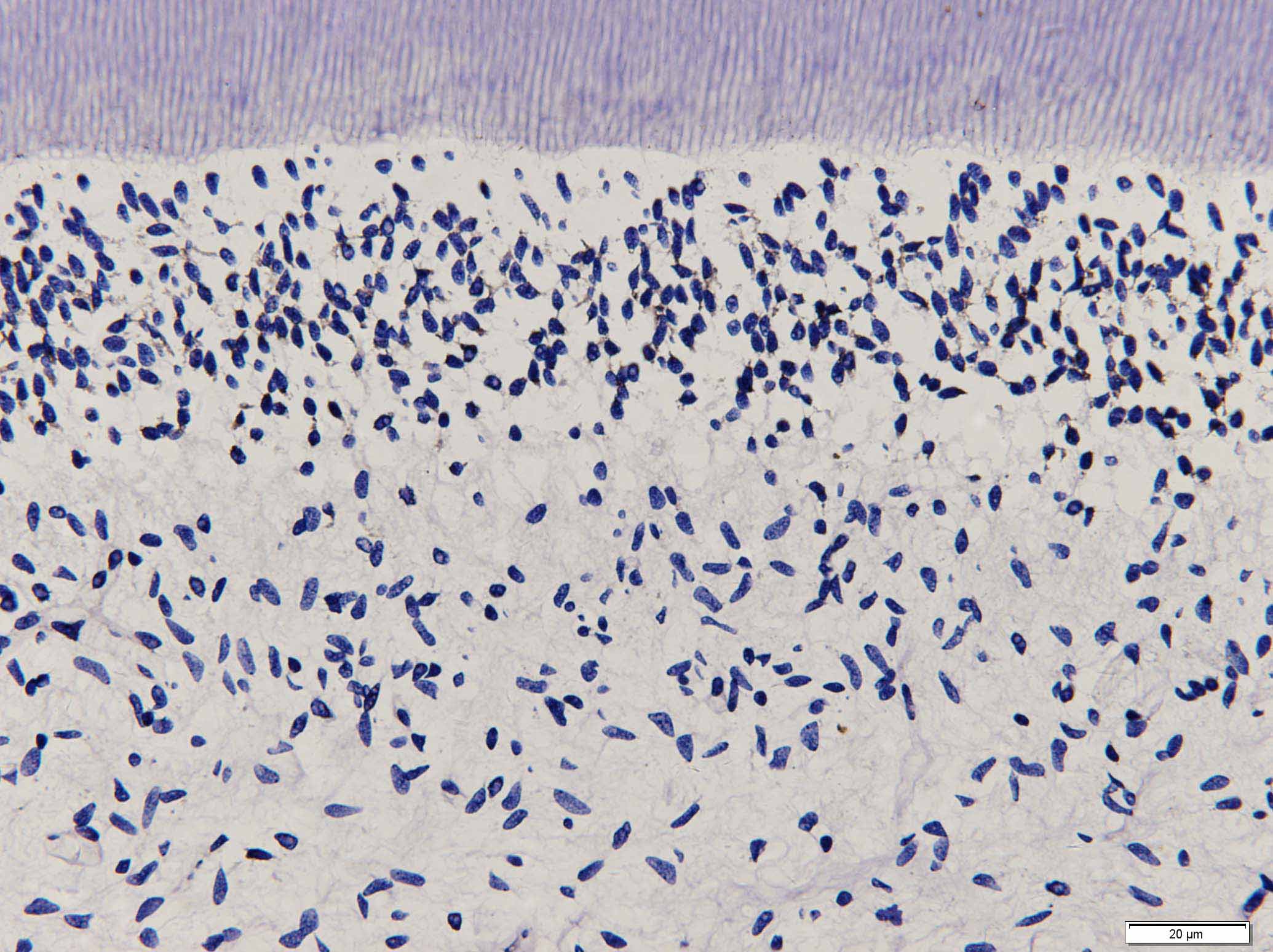

Supplement: Supplemental Information 1 — Immunohistochemical staining for sclerostin in young and senescent dental pulps. [file peerj-06-5808-s001.zip › Young/Image_9280.jpg]

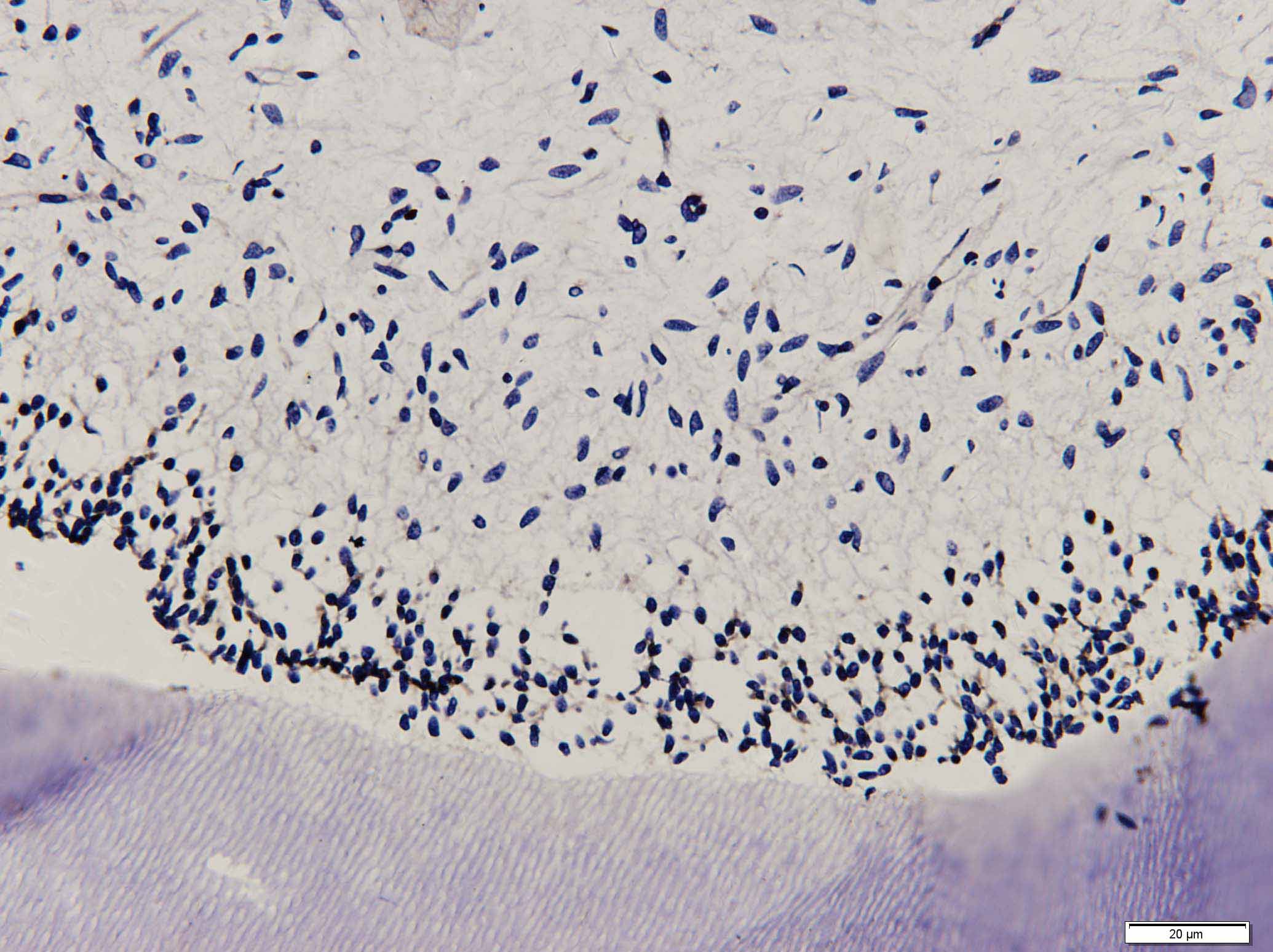

Supplement: Supplemental Information 1 — Immunohistochemical staining for sclerostin in young and senescent dental pulps. [file peerj-06-5808-s001.zip › Young/Image_9281.jpg]

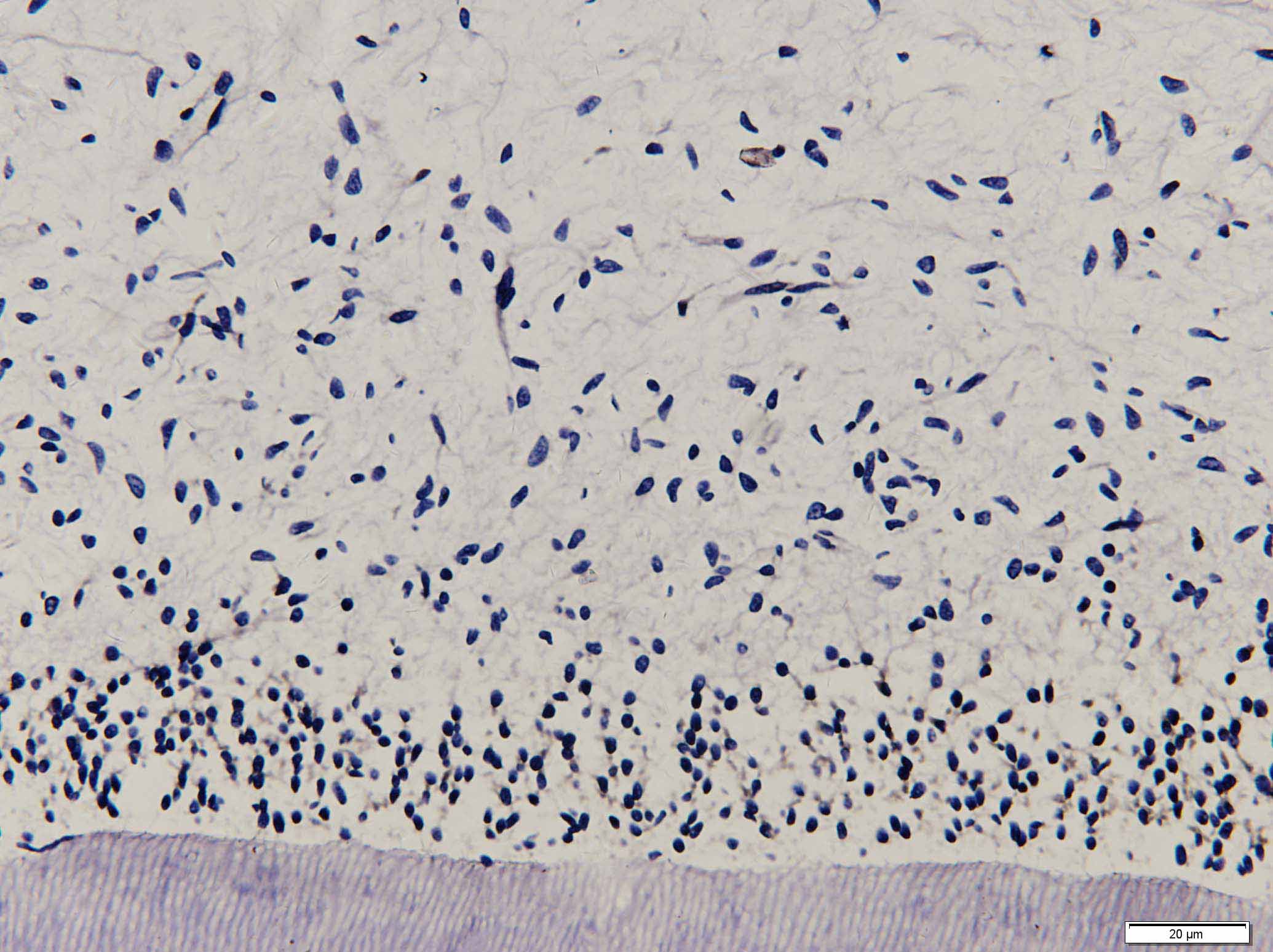

Supplement: Supplemental Information 1 — Immunohistochemical staining for sclerostin in young and senescent dental pulps. [file peerj-06-5808-s001.zip › Young/Image_9282.jpg]

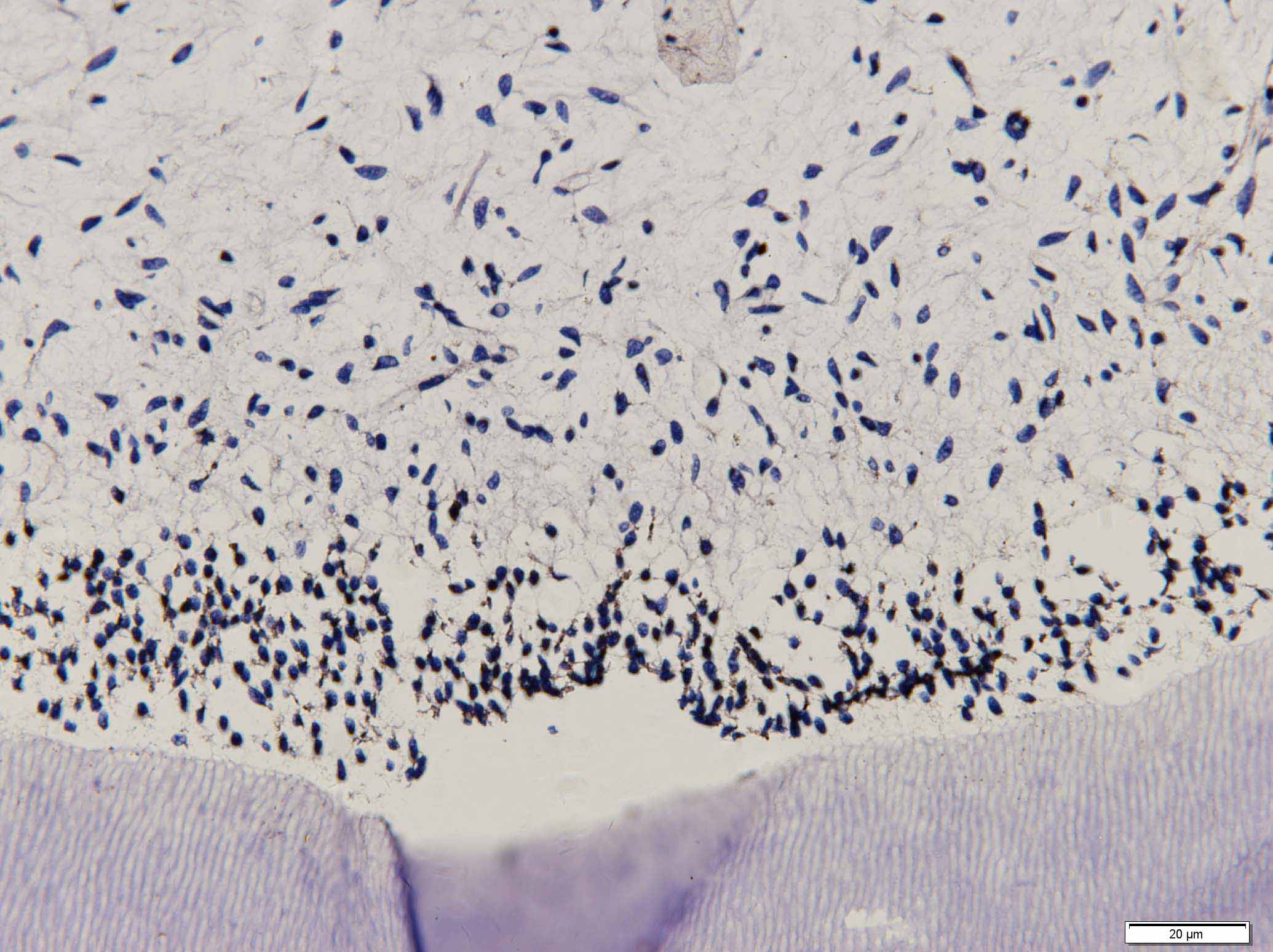

Supplement: Supplemental Information 1 — Immunohistochemical staining for sclerostin in young and senescent dental pulps. [file peerj-06-5808-s001.zip › Young/Image_9283.jpg]

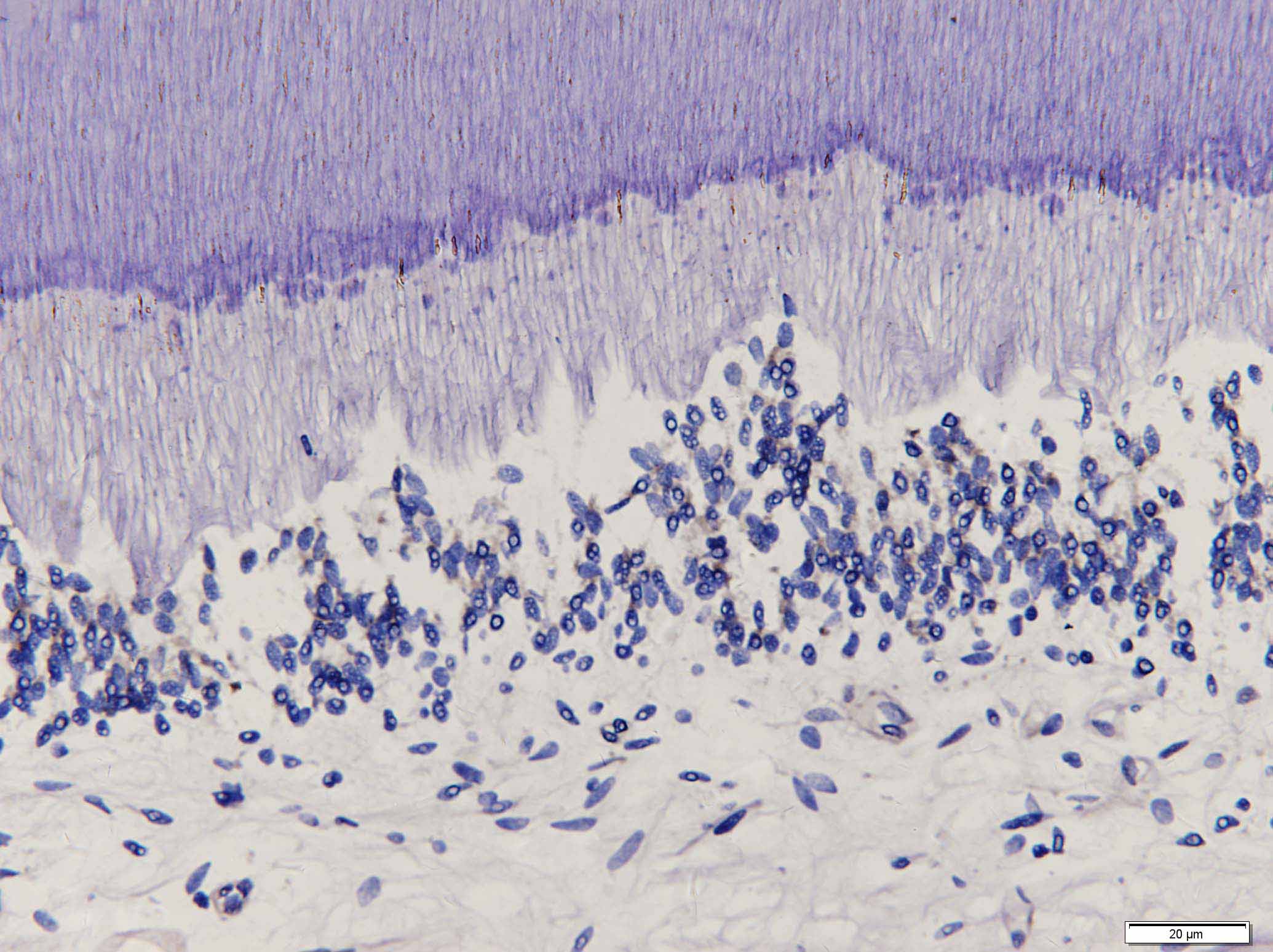

Supplement: Supplemental Information 1 — Immunohistochemical staining for sclerostin in young and senescent dental pulps. [file peerj-06-5808-s001.zip › Young/Image_9291.jpg]

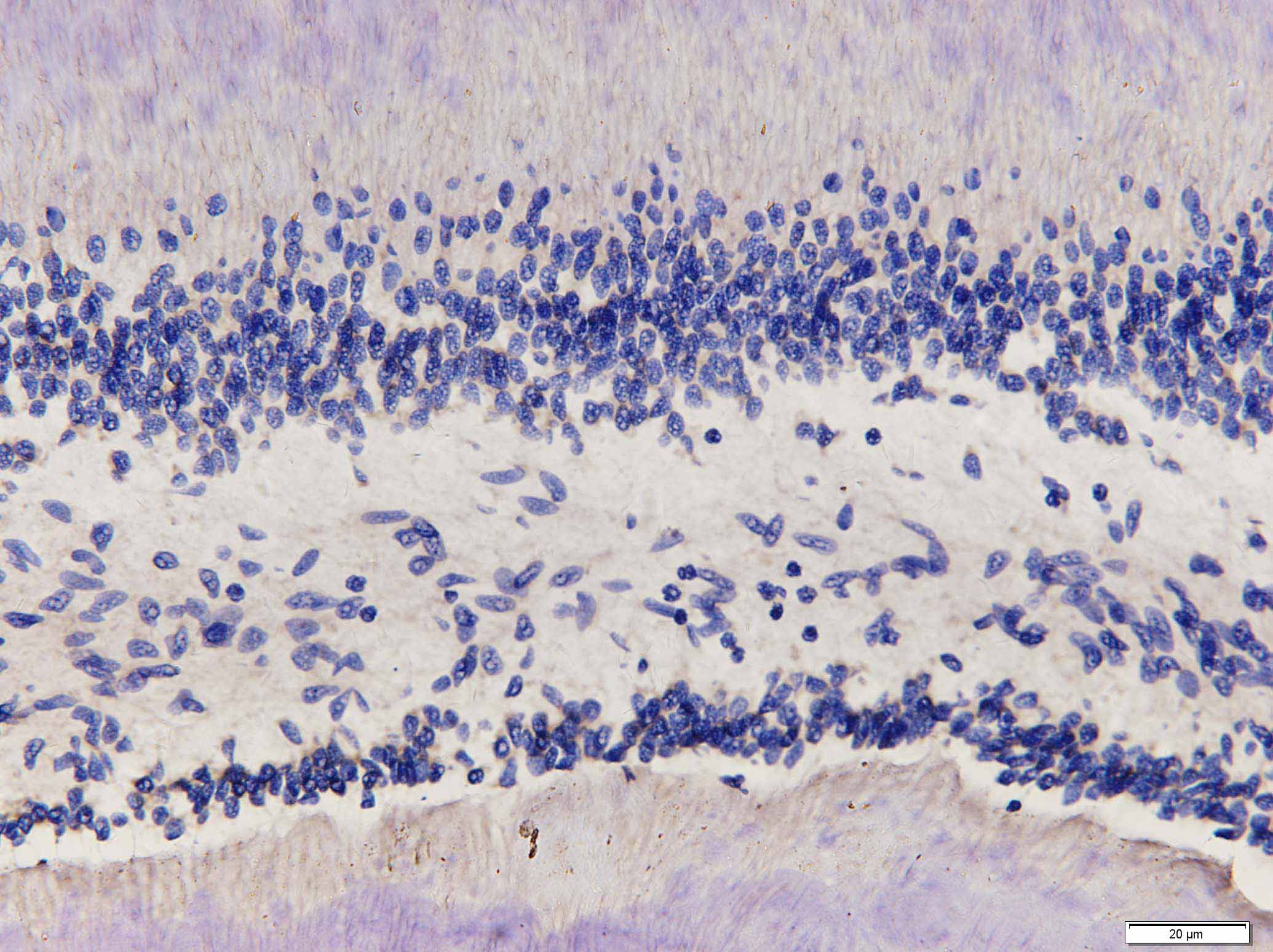

Supplement: Supplemental Information 1 — Immunohistochemical staining for sclerostin in young and senescent dental pulps. [file peerj-06-5808-s001.zip › Young/Image_9303.jpg]

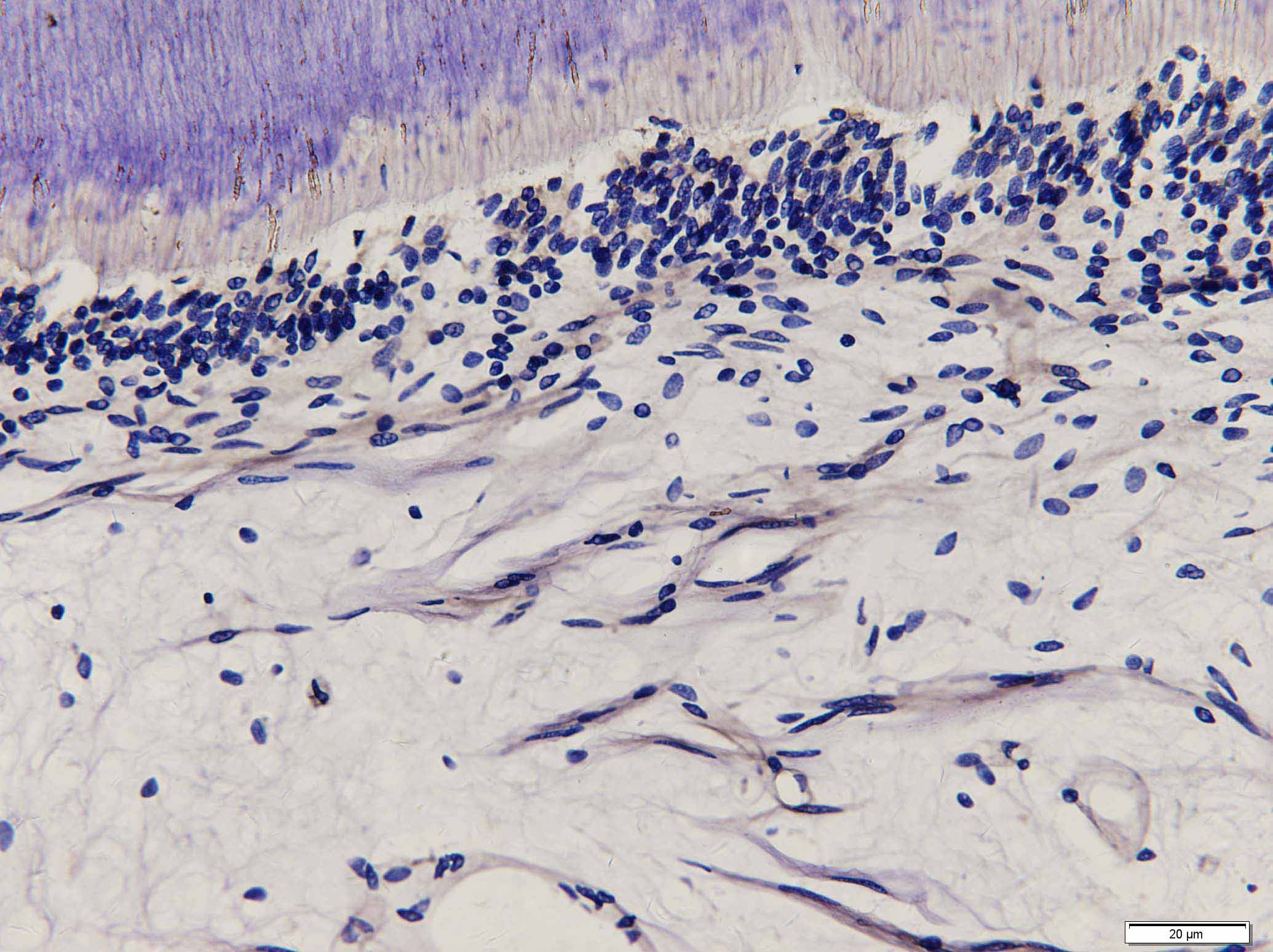

Supplement: Supplemental Information 1 — Immunohistochemical staining for sclerostin in young and senescent dental pulps. [file peerj-06-5808-s001.zip › Young/Image_9304.jpg]

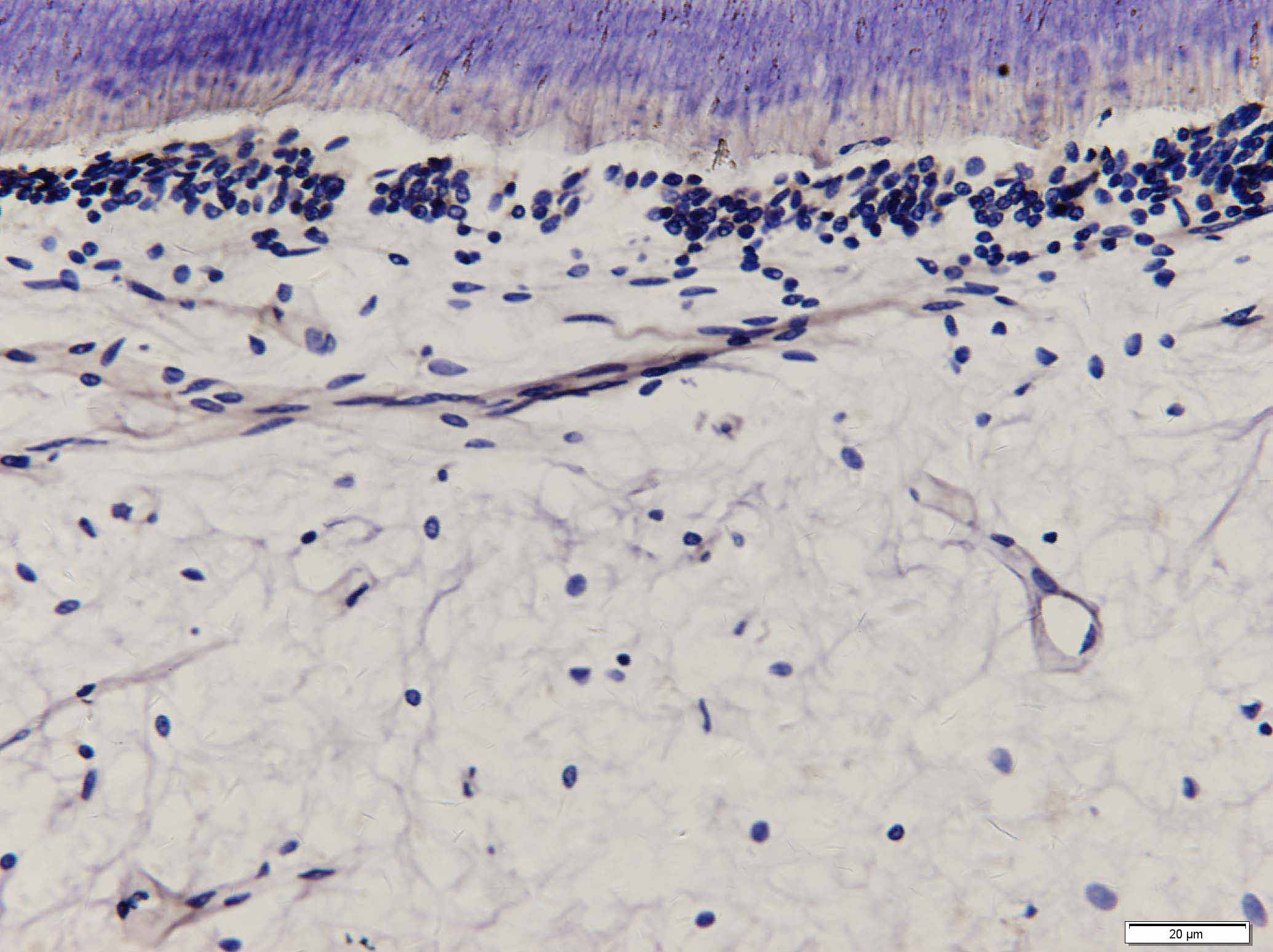

Supplement: Supplemental Information 1 — Immunohistochemical staining for sclerostin in young and senescent dental pulps. [file peerj-06-5808-s001.zip › Young/Image_9305.jpg]

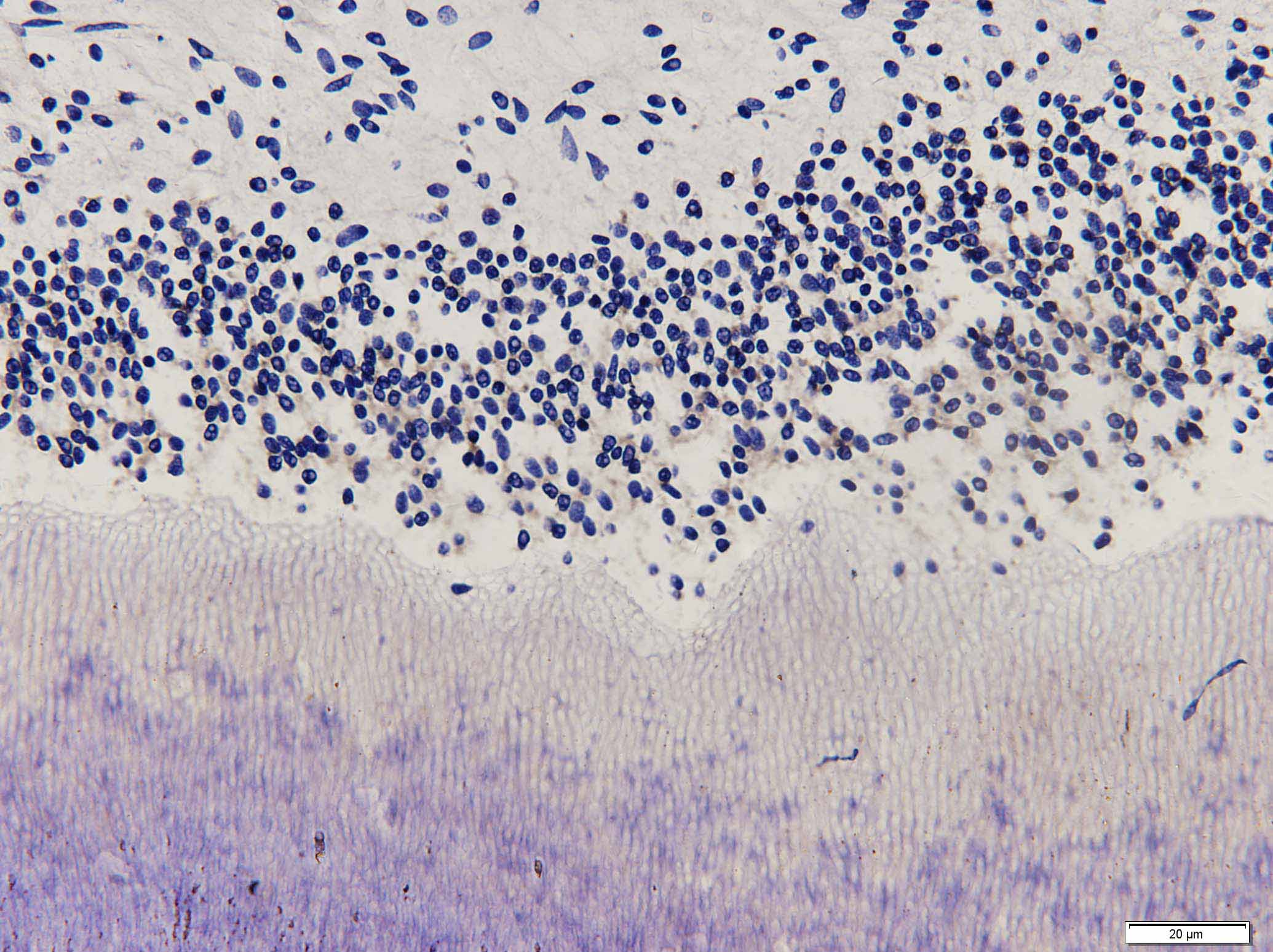

Supplement: Supplemental Information 1 — Immunohistochemical staining for sclerostin in young and senescent dental pulps. [file peerj-06-5808-s001.zip › Young/Image_9306.jpg]

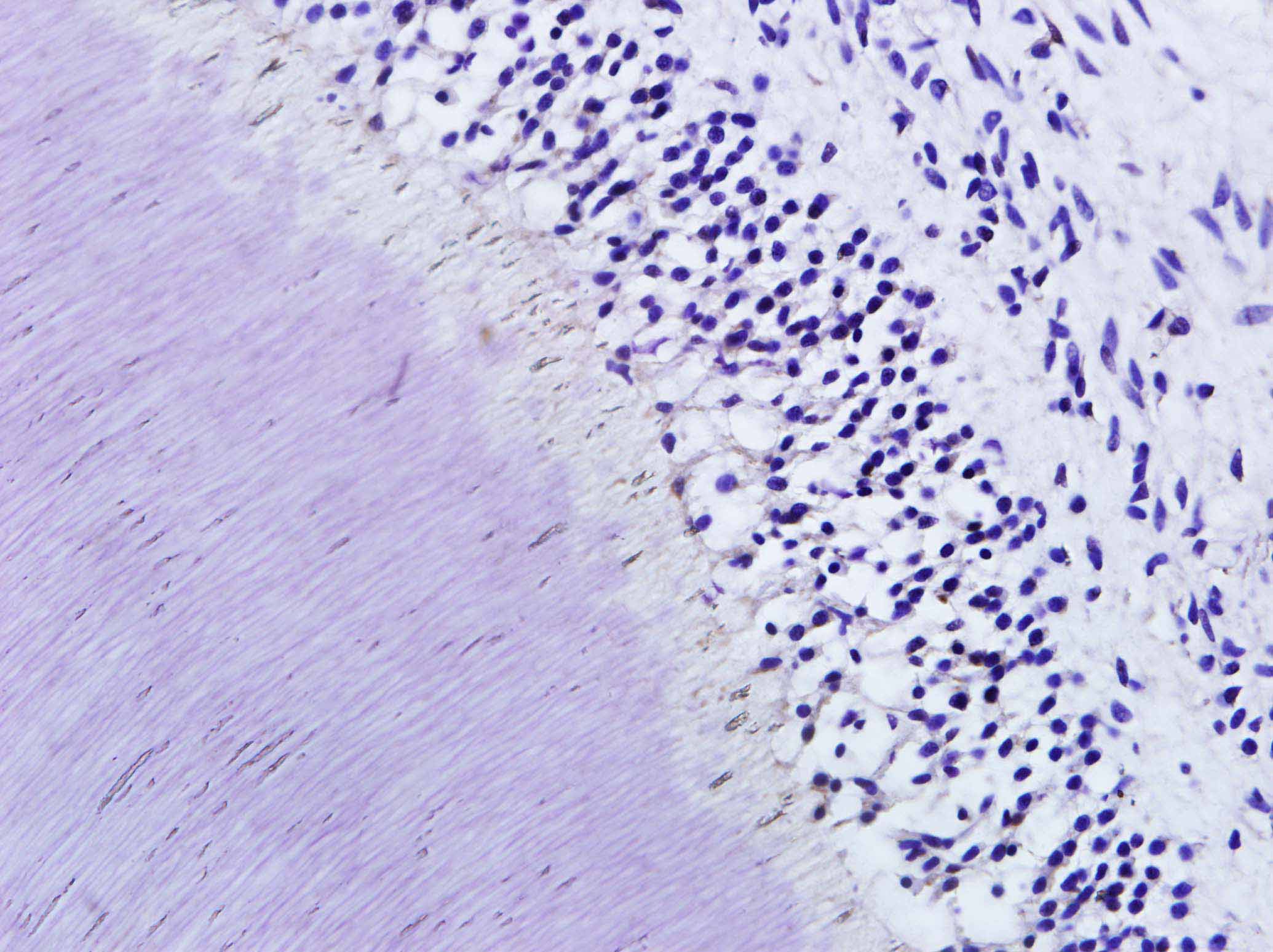

Supplement: Supplemental Information 1 — Immunohistochemical staining for sclerostin in young and senescent dental pulps. [file peerj-06-5808-s001.zip › Young/图像_29190.jpg]

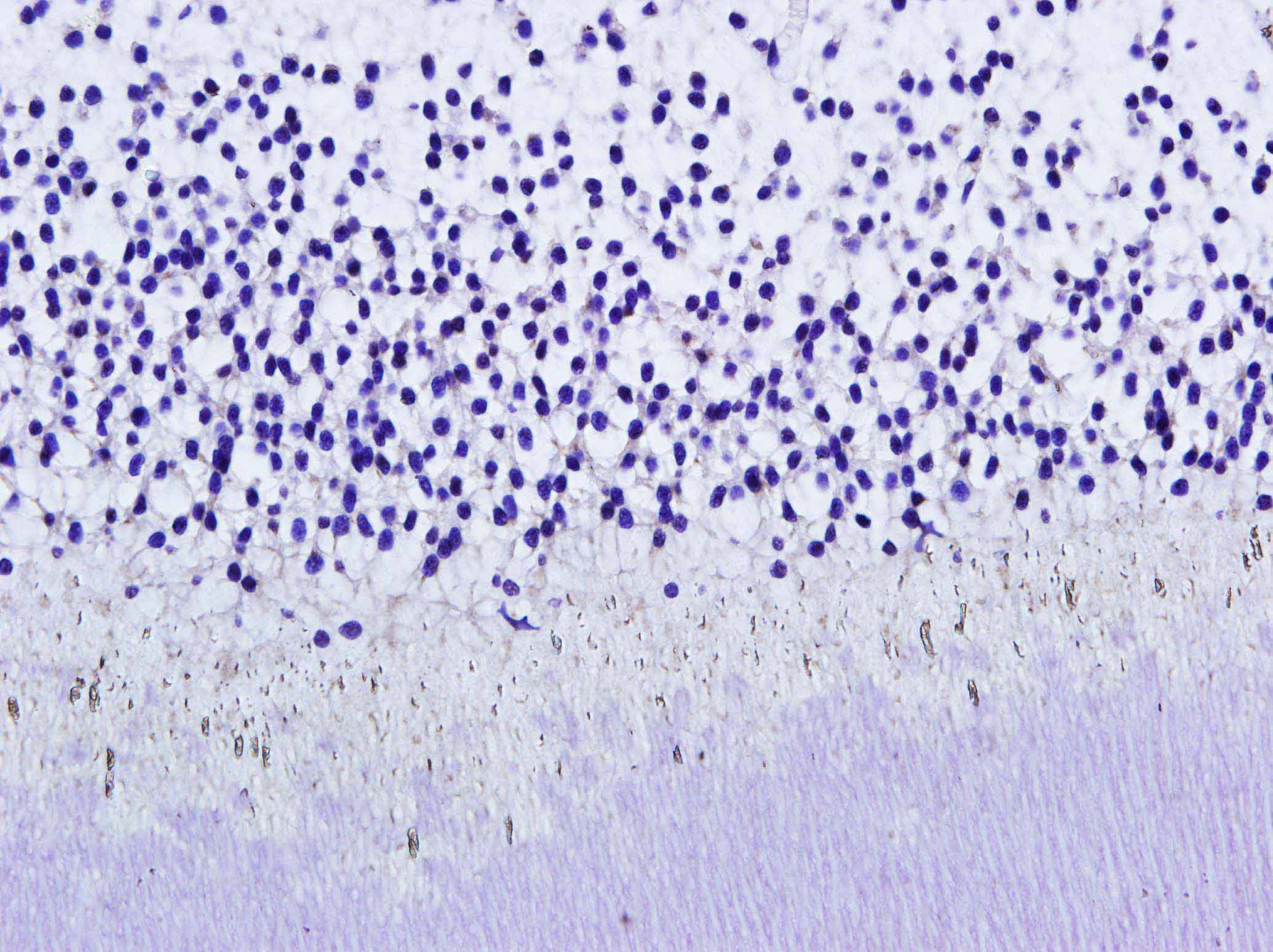

Supplement: Supplemental Information 1 — Immunohistochemical staining for sclerostin in young and senescent dental pulps. [file peerj-06-5808-s001.zip › Young/图像_29192.jpg]

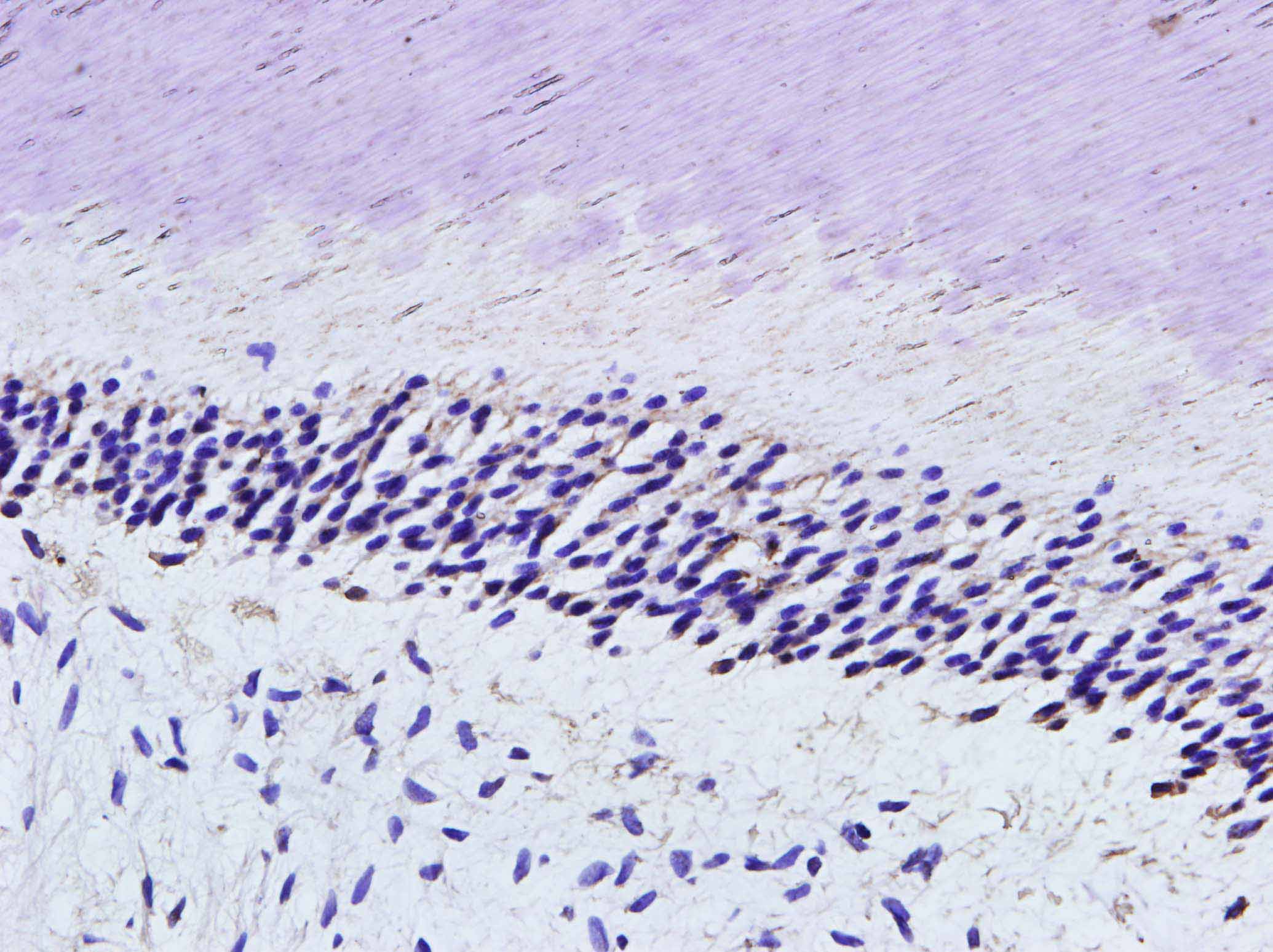

Supplement: Supplemental Information 1 — Immunohistochemical staining for sclerostin in young and senescent dental pulps. [file peerj-06-5808-s001.zip › Young/图像_29193.jpg]

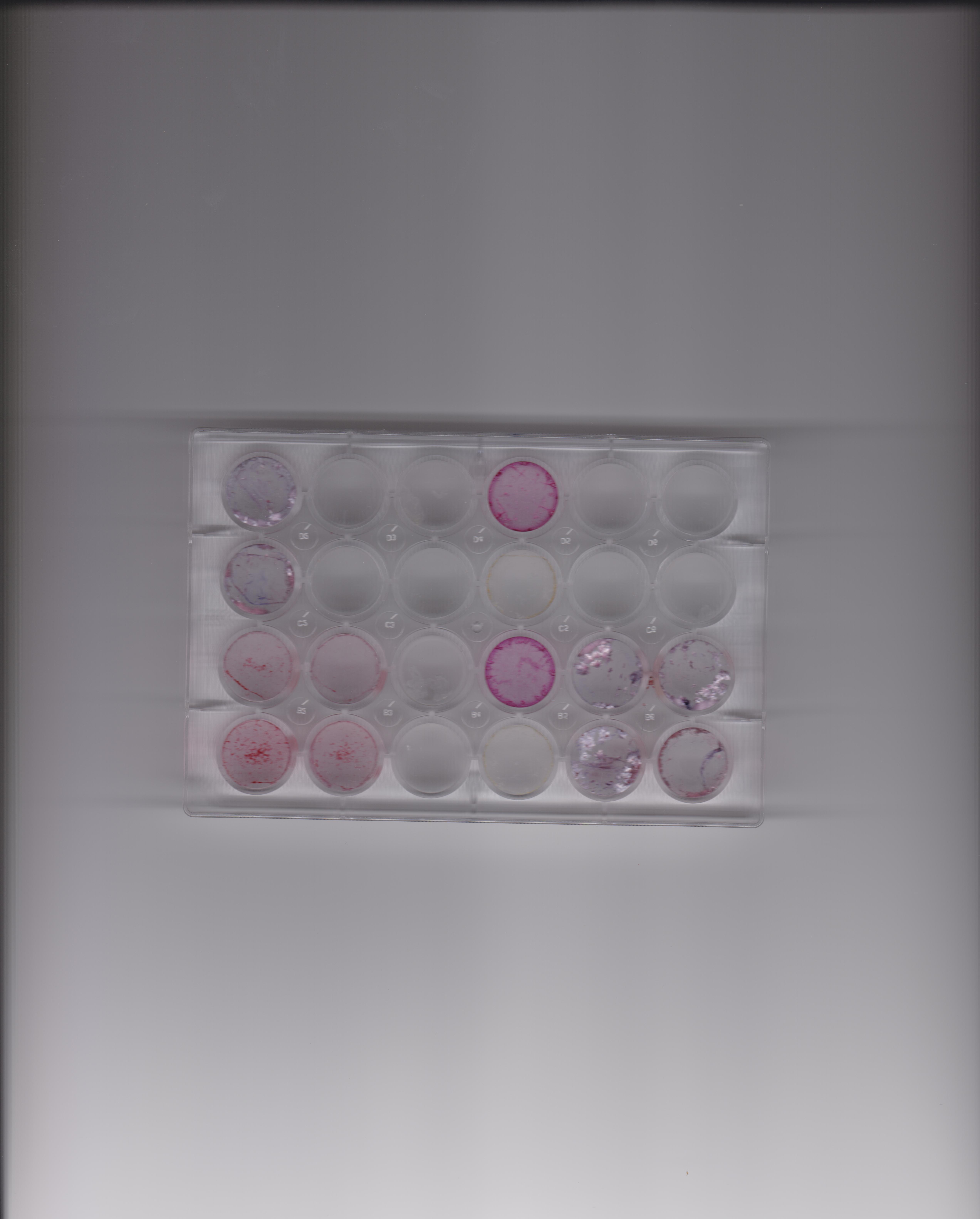

Supplement: Supplemental Information 2 — Proliferation, differentiation and other raw data from this study. [file peerj-06-5808-s002.zip › Alizarin Red/sh-SOST/Alizarin Red dpc 14days-1.jpg]

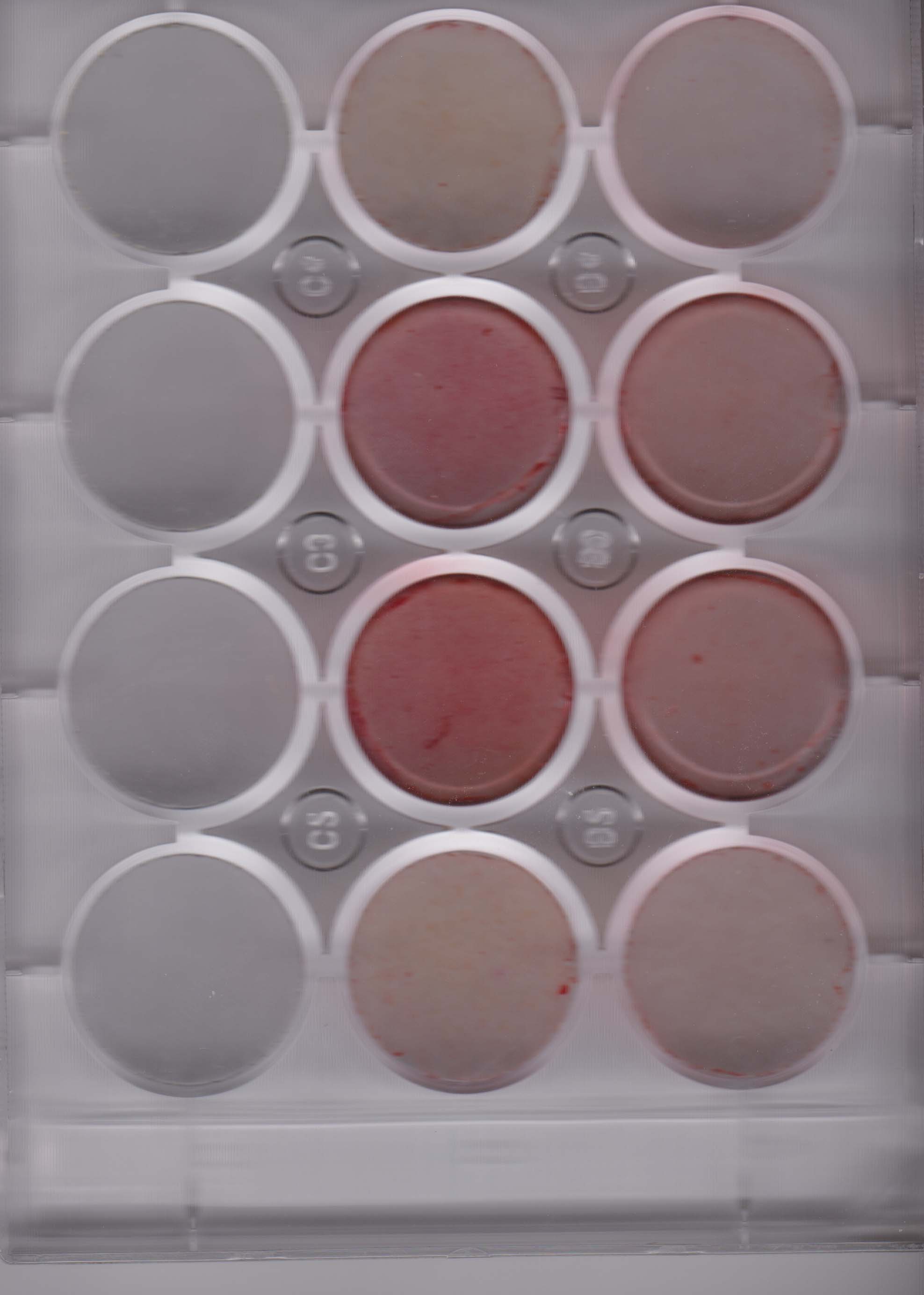

Supplement: Supplemental Information 2 — Proliferation, differentiation and other raw data from this study. [file peerj-06-5808-s002.zip › Alizarin Red/sh-SOST/Alizarin Red dpc 14days-2.jpg]

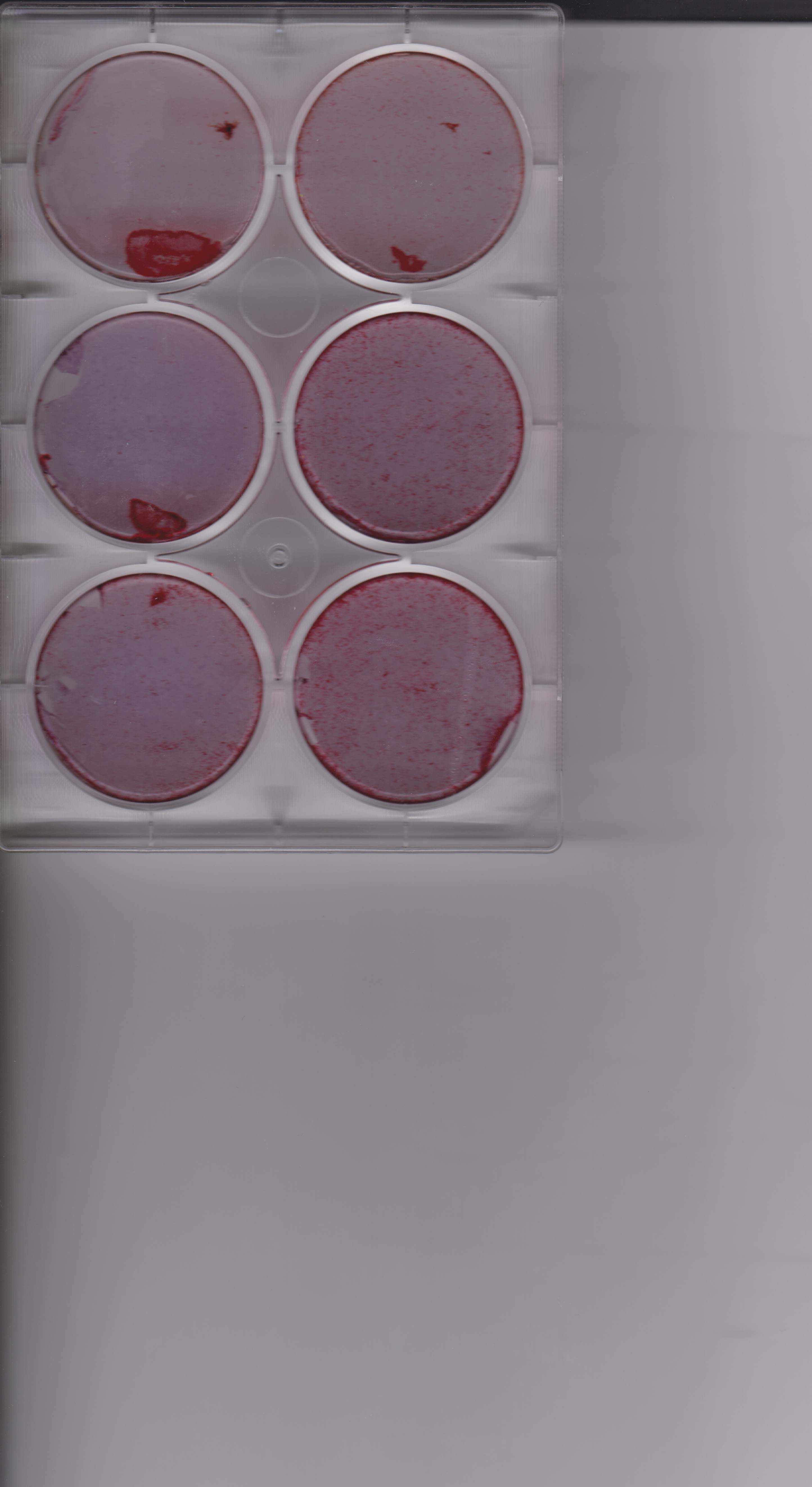

Supplement: Supplemental Information 2 — Proliferation, differentiation and other raw data from this study. [file peerj-06-5808-s002.zip › Alizarin Red/sh-SOST/Alizarin Red dpc 14days-3.jpg]

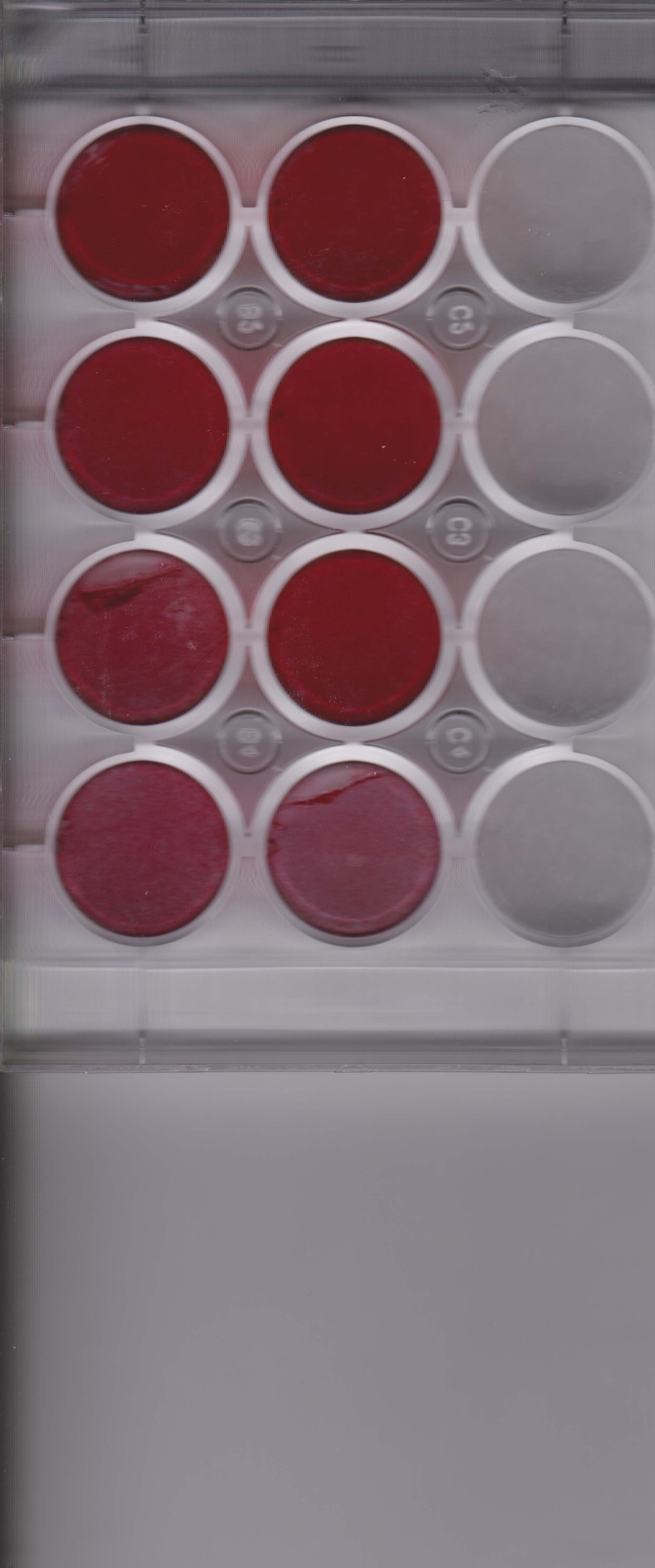

Supplement: Supplemental Information 2 — Proliferation, differentiation and other raw data from this study. [file peerj-06-5808-s002.zip › Alizarin Red/SOST-OVER/Alizarin Red dpc 14days-1.jpg]

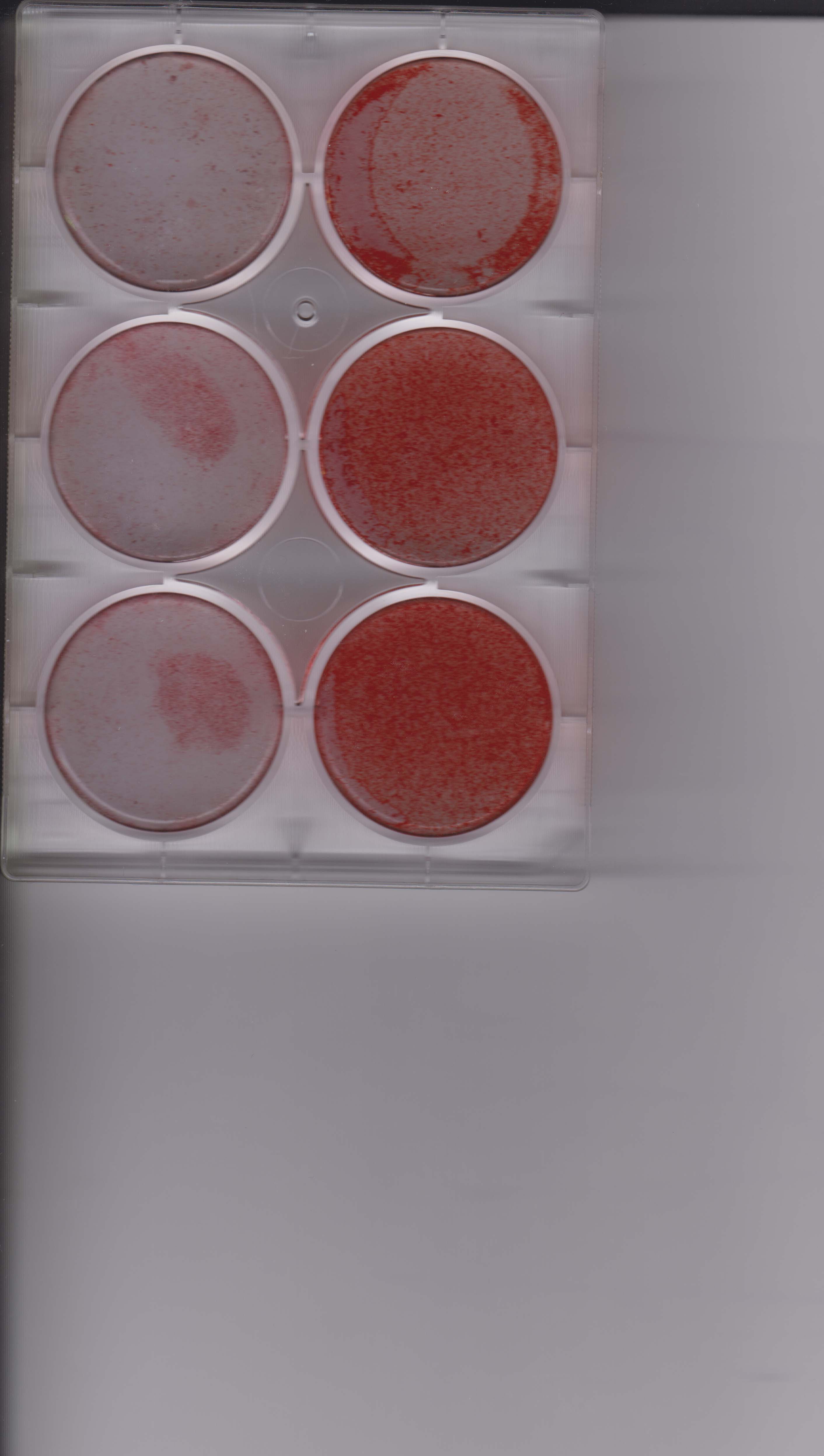

Supplement: Supplemental Information 2 — Proliferation, differentiation and other raw data from this study. [file peerj-06-5808-s002.zip › Alizarin Red/SOST-OVER/Alizarin Red dpc 14days-2.jpg]

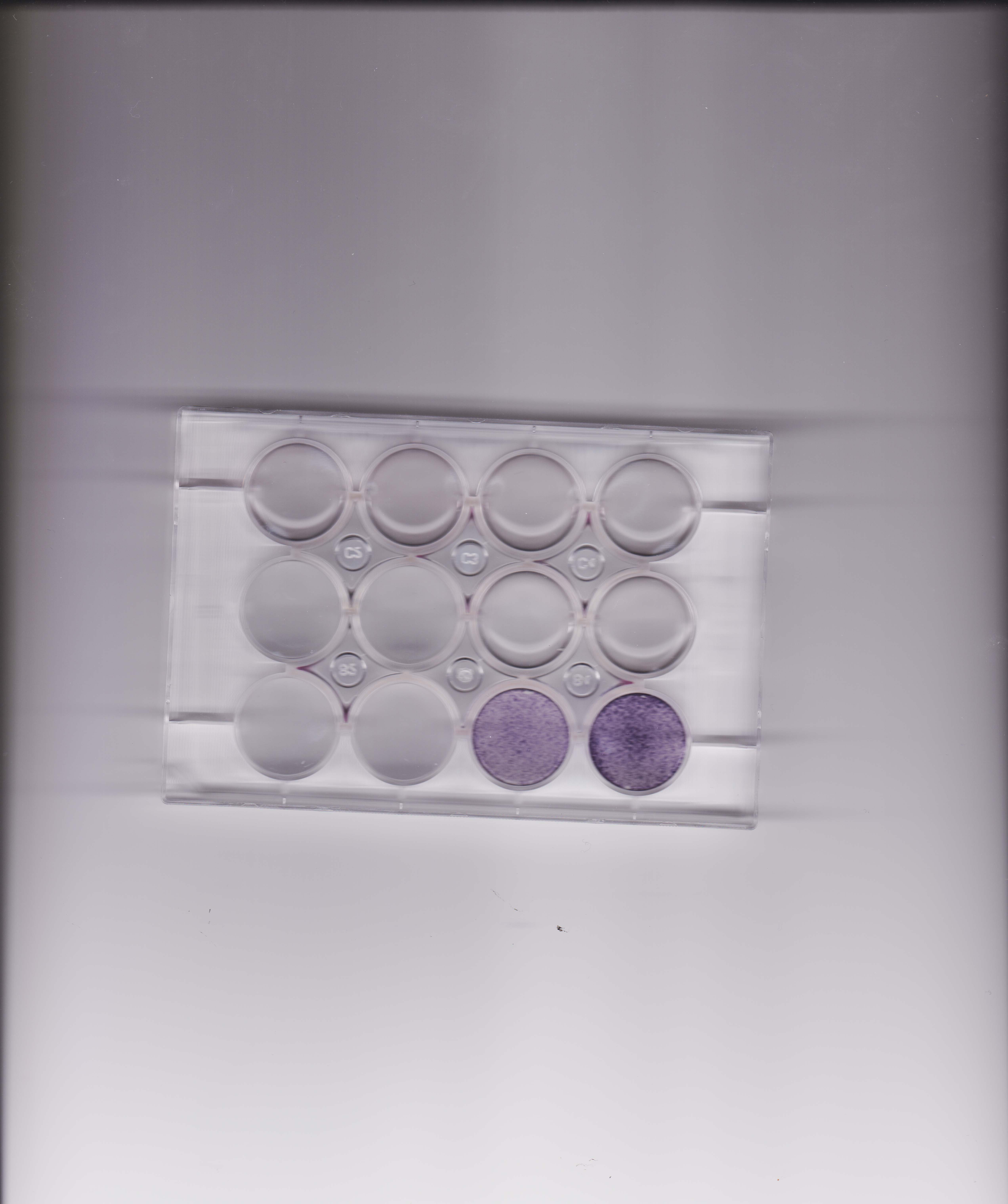

Supplement: Supplemental Information 2 — Proliferation, differentiation and other raw data from this study. [file peerj-06-5808-s002.zip › alp/sh-SOST/001.jpg]

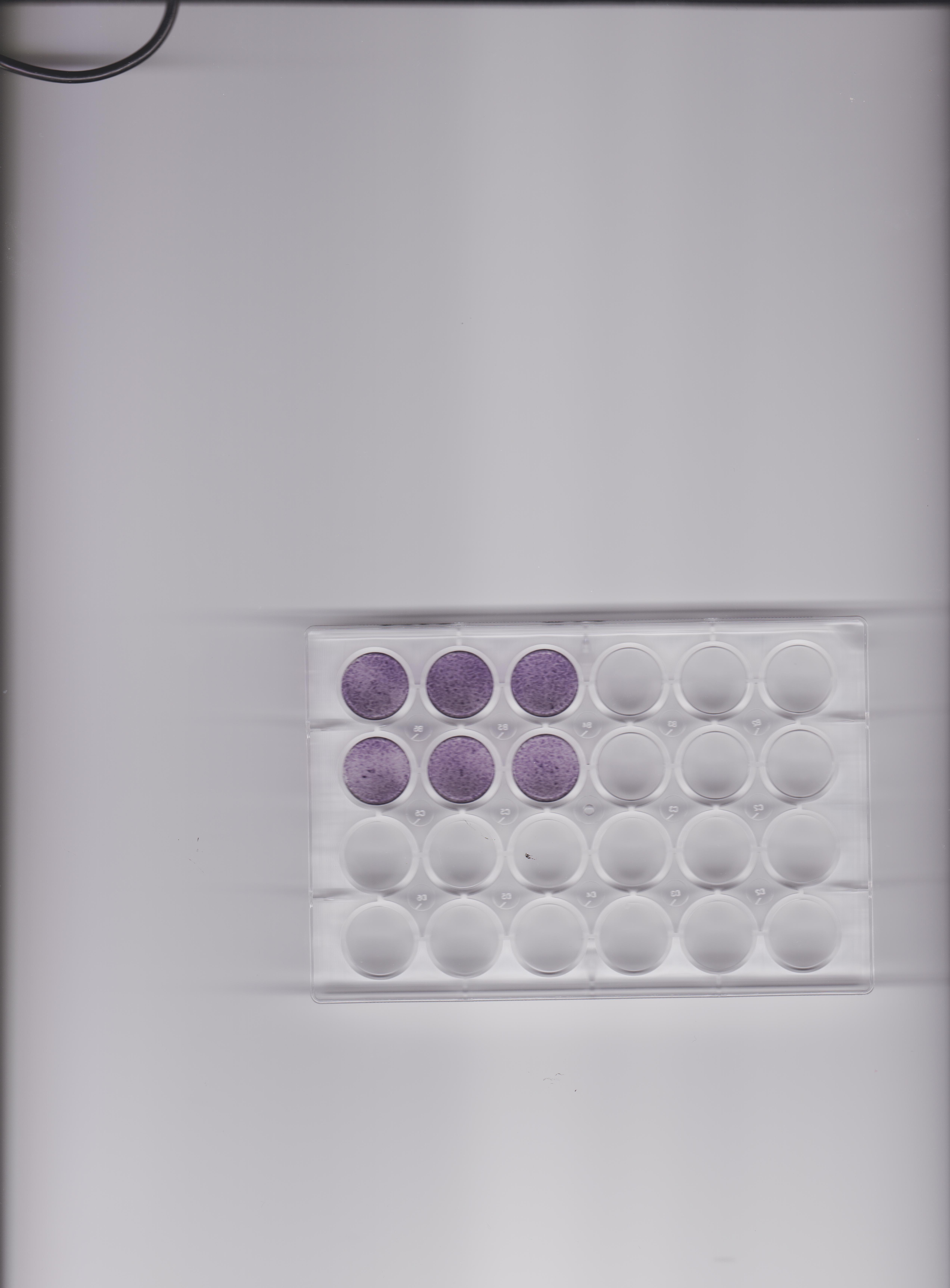

Supplement: Supplemental Information 2 — Proliferation, differentiation and other raw data from this study. [file peerj-06-5808-s002.zip › alp/sh-SOST/alp sh-sost.jpg]

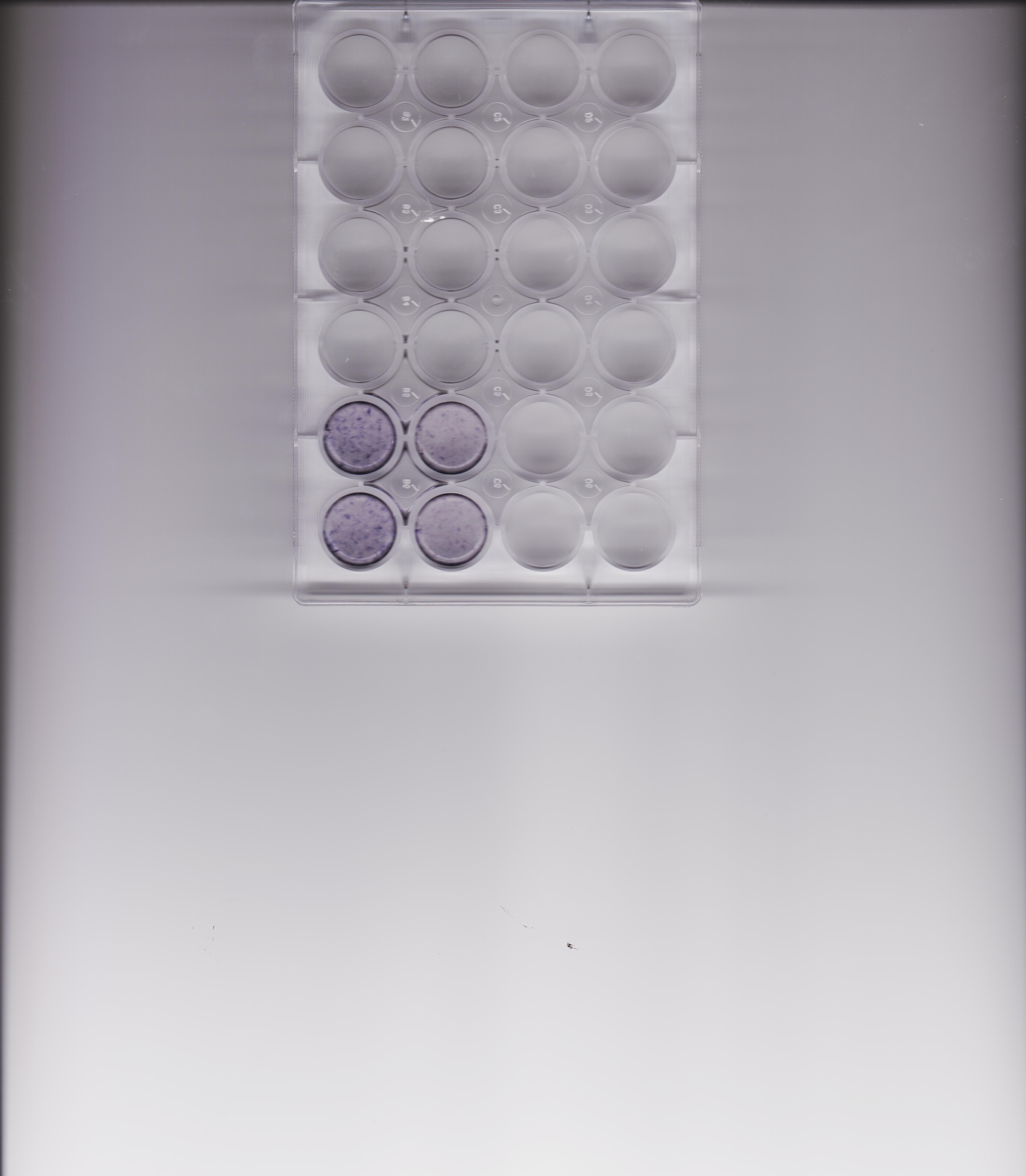

Supplement: Supplemental Information 2 — Proliferation, differentiation and other raw data from this study. [file peerj-06-5808-s002.zip › alp/sh-SOST/sh-sost ALP.jpg]

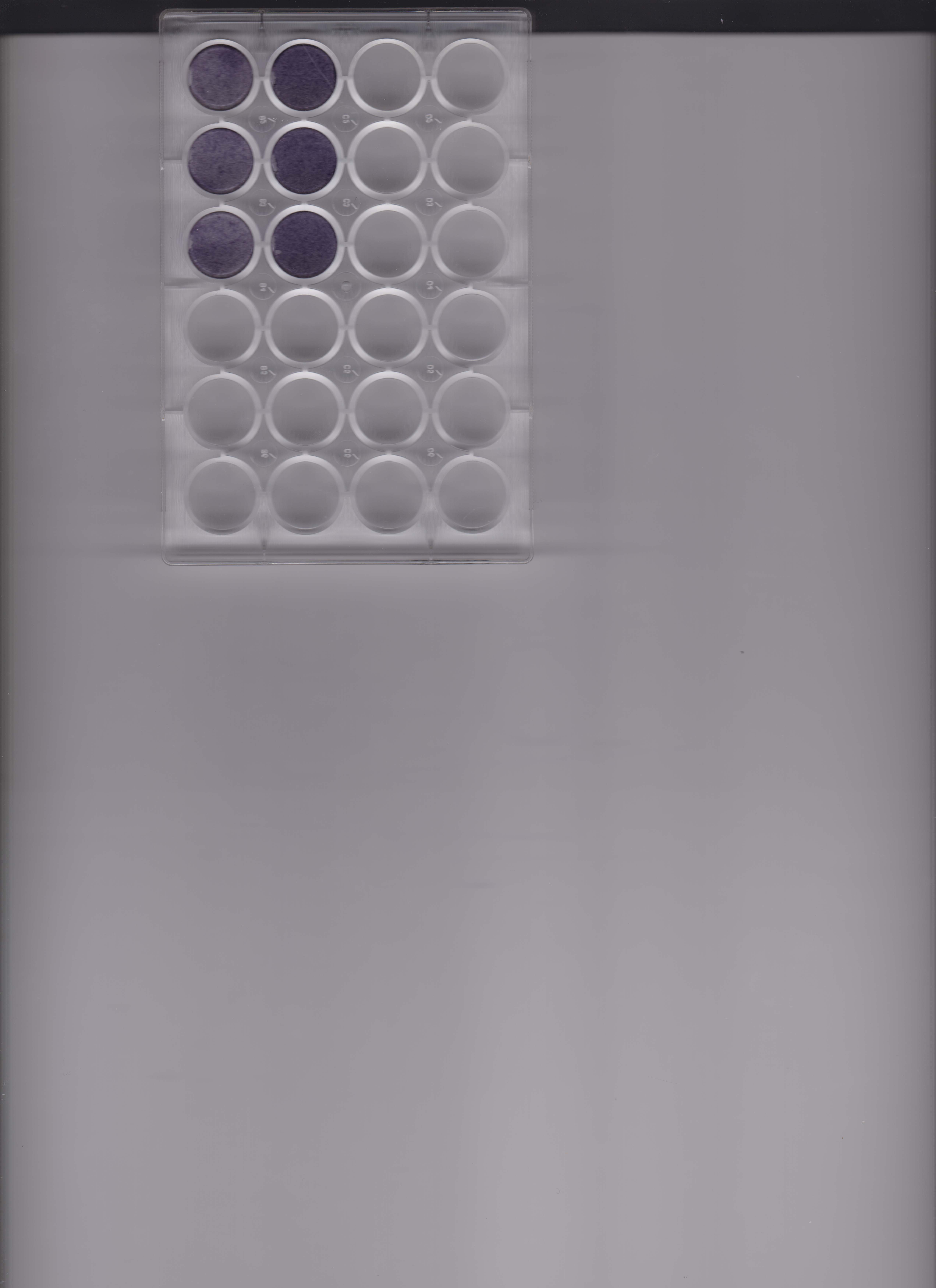

Supplement: Supplemental Information 2 — Proliferation, differentiation and other raw data from this study. [file peerj-06-5808-s002.zip › alp/SOST-OVER/ALP 001.jpg]

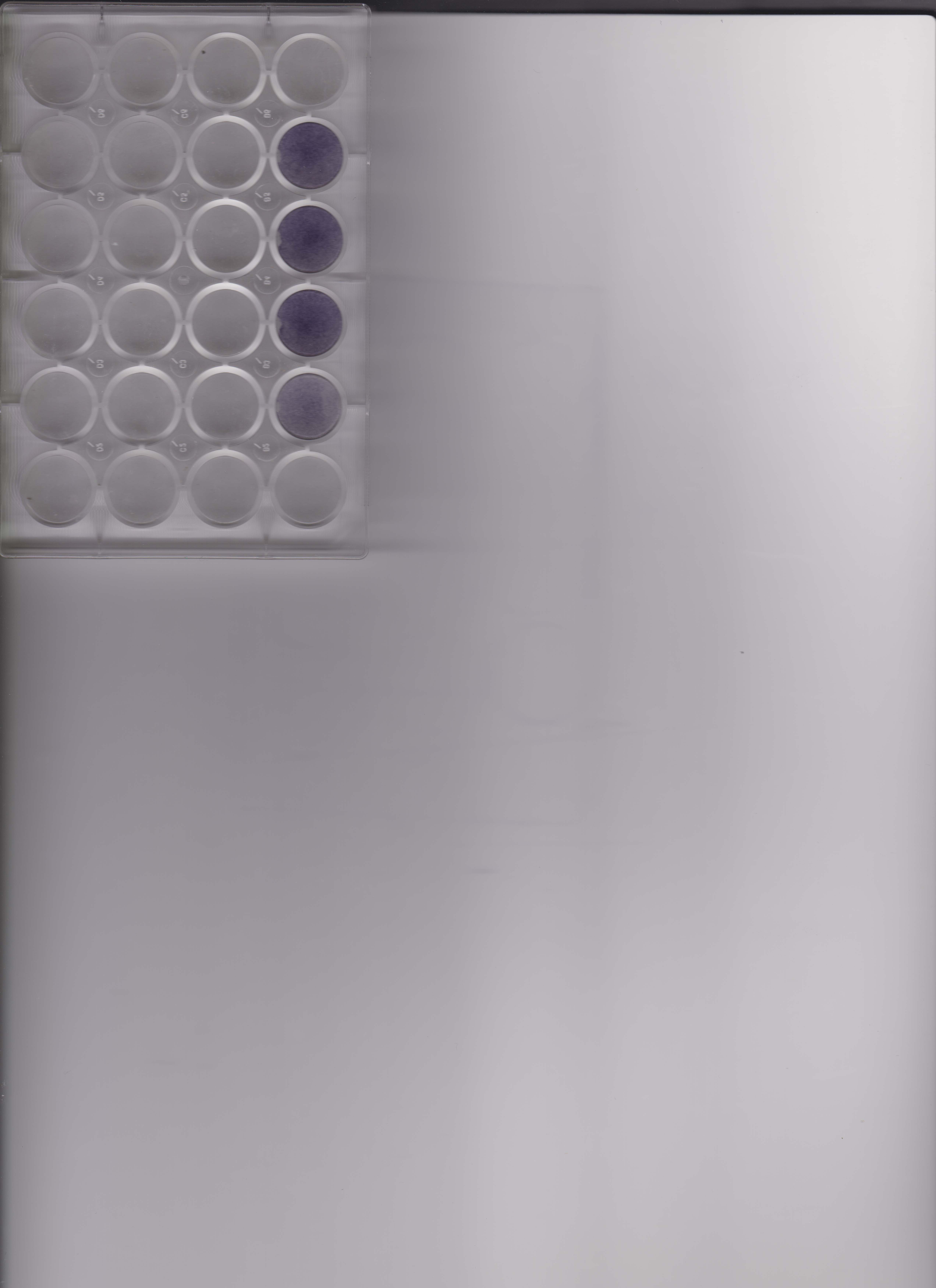

Supplement: Supplemental Information 2 — Proliferation, differentiation and other raw data from this study. [file peerj-06-5808-s002.zip › alp/SOST-OVER/alp 002.jpg]

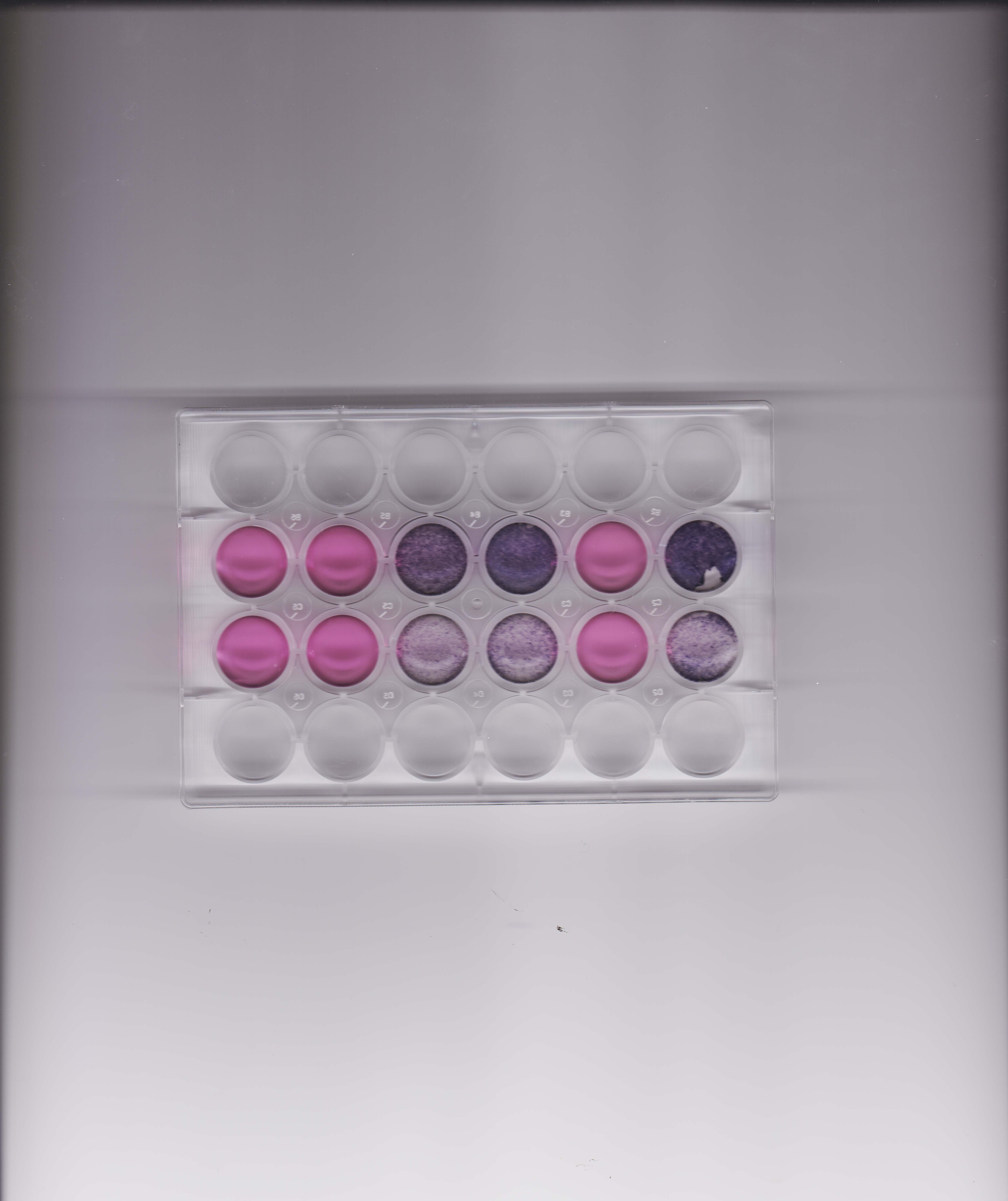

Supplement: Supplemental Information 2 — Proliferation, differentiation and other raw data from this study. [file peerj-06-5808-s002.zip › alp/SOST-OVER/sost over ALP.jpg]

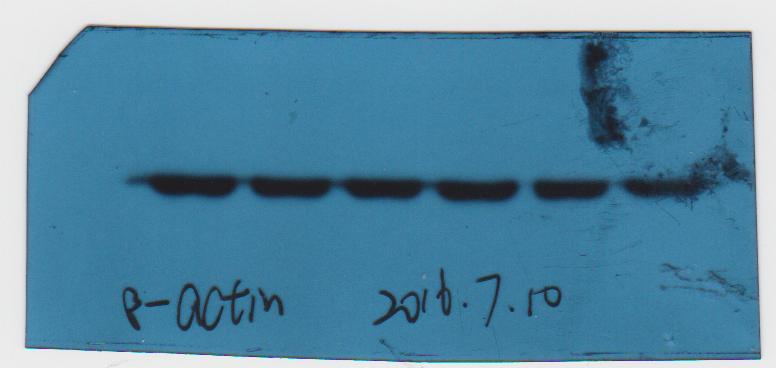

Supplement: Supplemental Information 2 — Proliferation, differentiation and other raw data from this study. [file peerj-06-5808-s002.zip › Western blot/sh-sost actin.jpg]

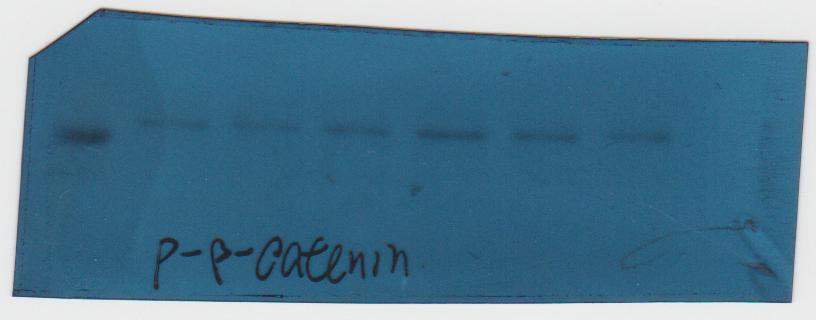

Supplement: Supplemental Information 2 — Proliferation, differentiation and other raw data from this study. [file peerj-06-5808-s002.zip › Western blot/sh-sost p-b-catenin-2.jpg]

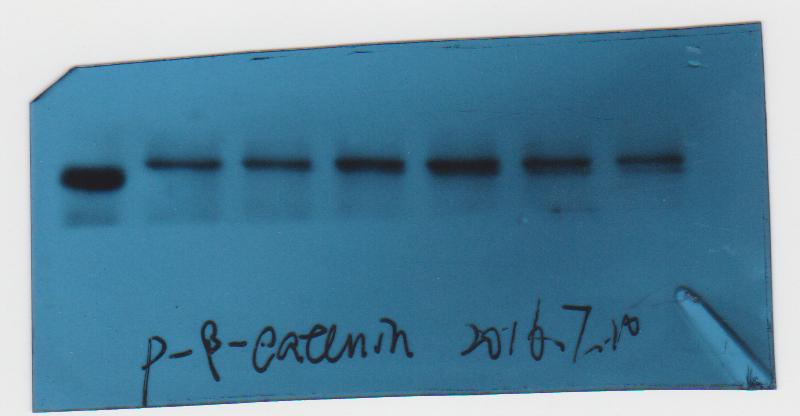

Supplement: Supplemental Information 2 — Proliferation, differentiation and other raw data from this study. [file peerj-06-5808-s002.zip › Western blot/sh-sost p-b-catenin.jpg]

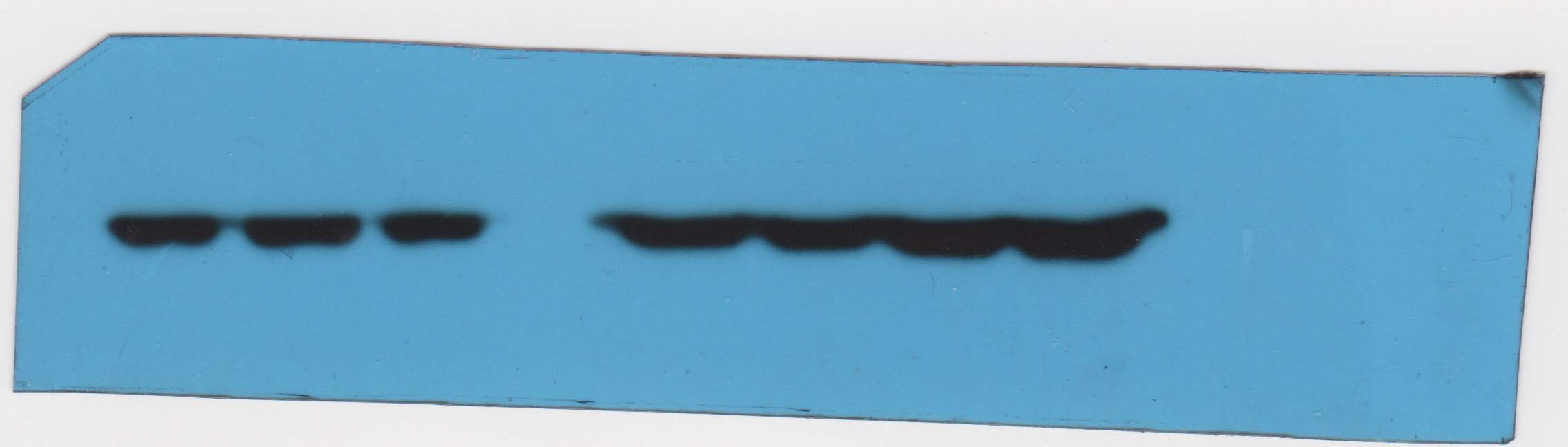

Supplement: Supplemental Information 2 — Proliferation, differentiation and other raw data from this study. [file peerj-06-5808-s002.zip › Western blot/sost over actin.jpg]

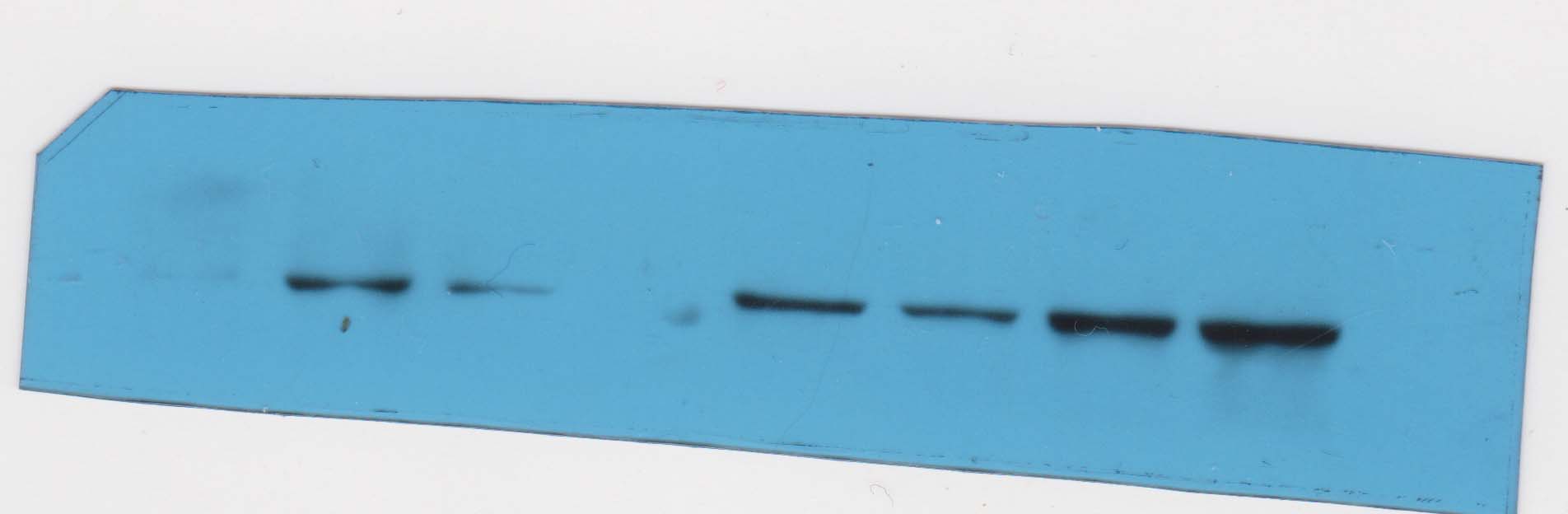

Supplement: Supplemental Information 2 — Proliferation, differentiation and other raw data from this study. [file peerj-06-5808-s002.zip › Western blot/sost over p-b-catenin.jpg]

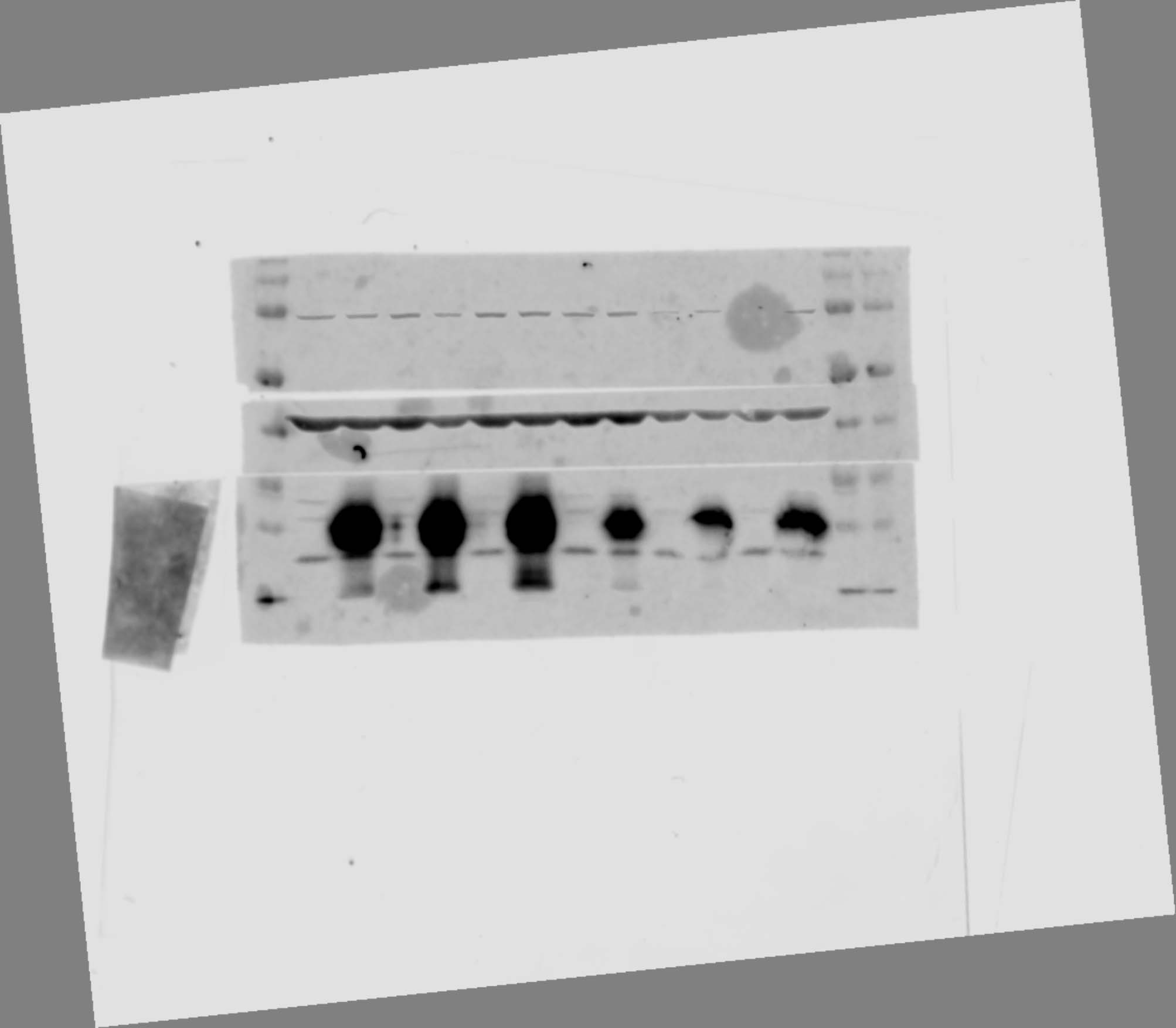

Supplement: Supplemental Information 2 — Proliferation, differentiation and other raw data from this study. [file peerj-06-5808-s002.zip › Western blot/SOST with marker.jpg]

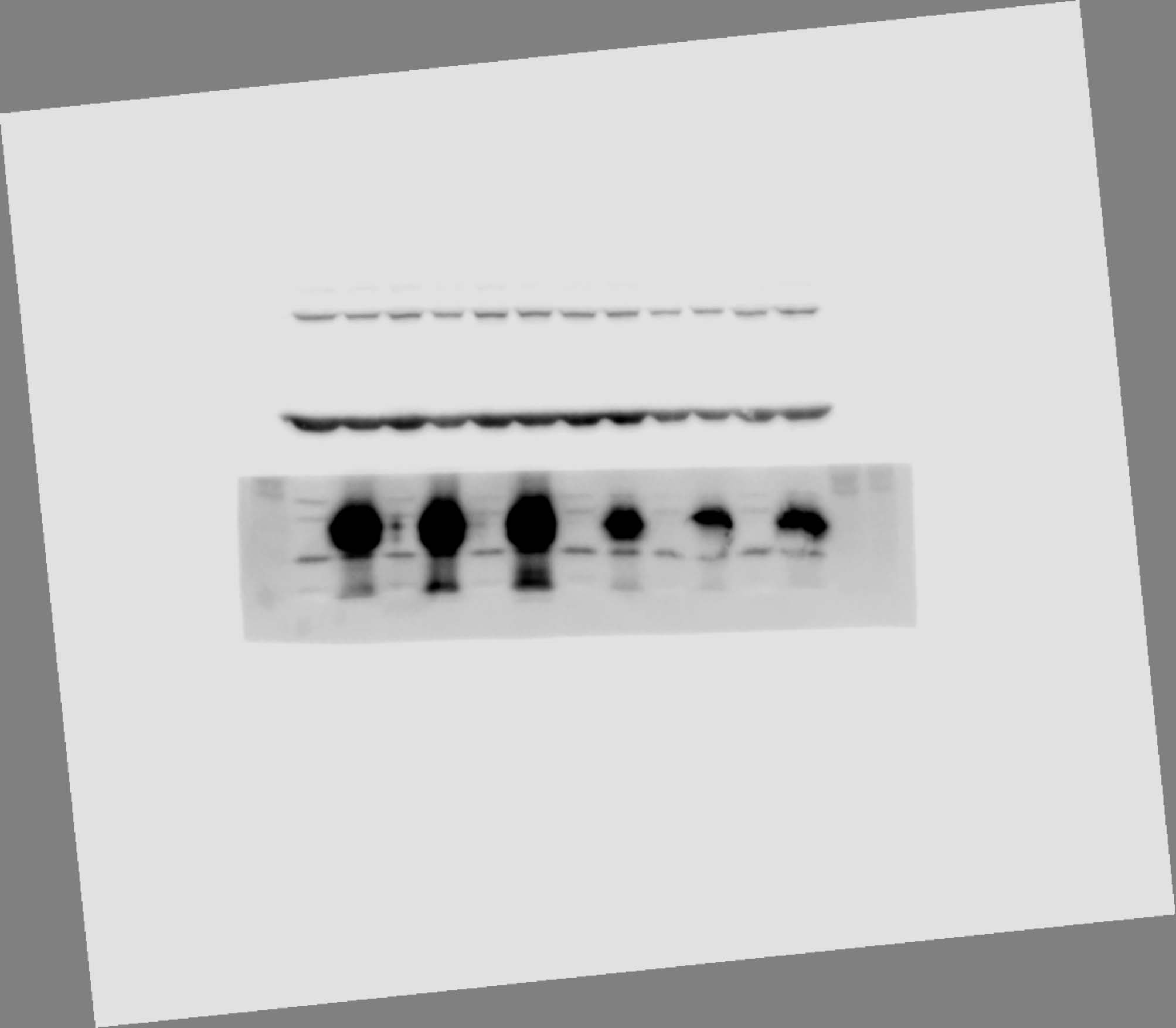

Supplement: Supplemental Information 2 — Proliferation, differentiation and other raw data from this study. [file peerj-06-5808-s002.zip › Western blot/sost.jpg]

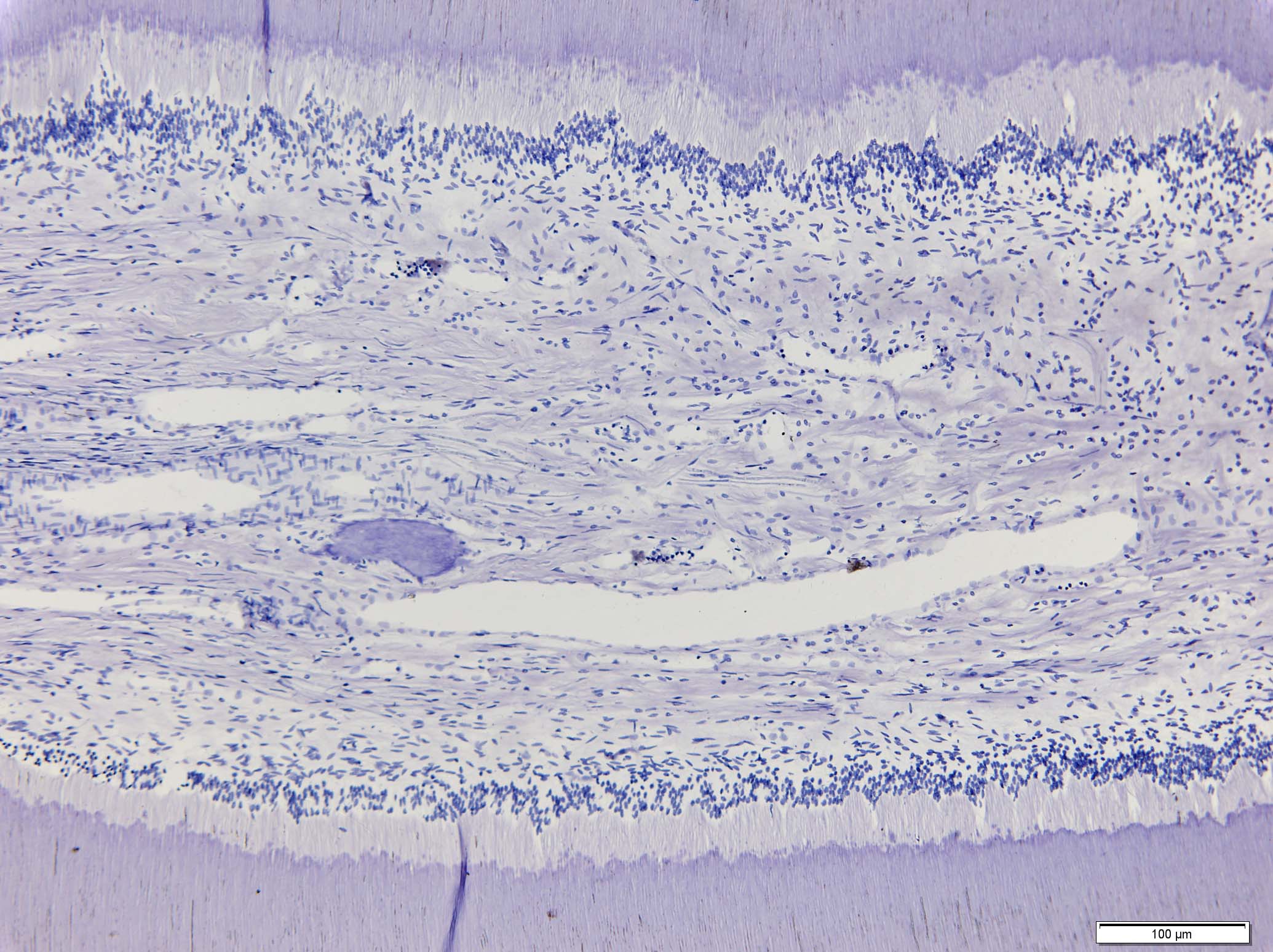

Supplement: Supplemental Information 3 — Sections were incubated without primary antibody, and then the same procedures were done. [file peerj-06-5808-s003.zip › negative control/Image_9237.jpg]

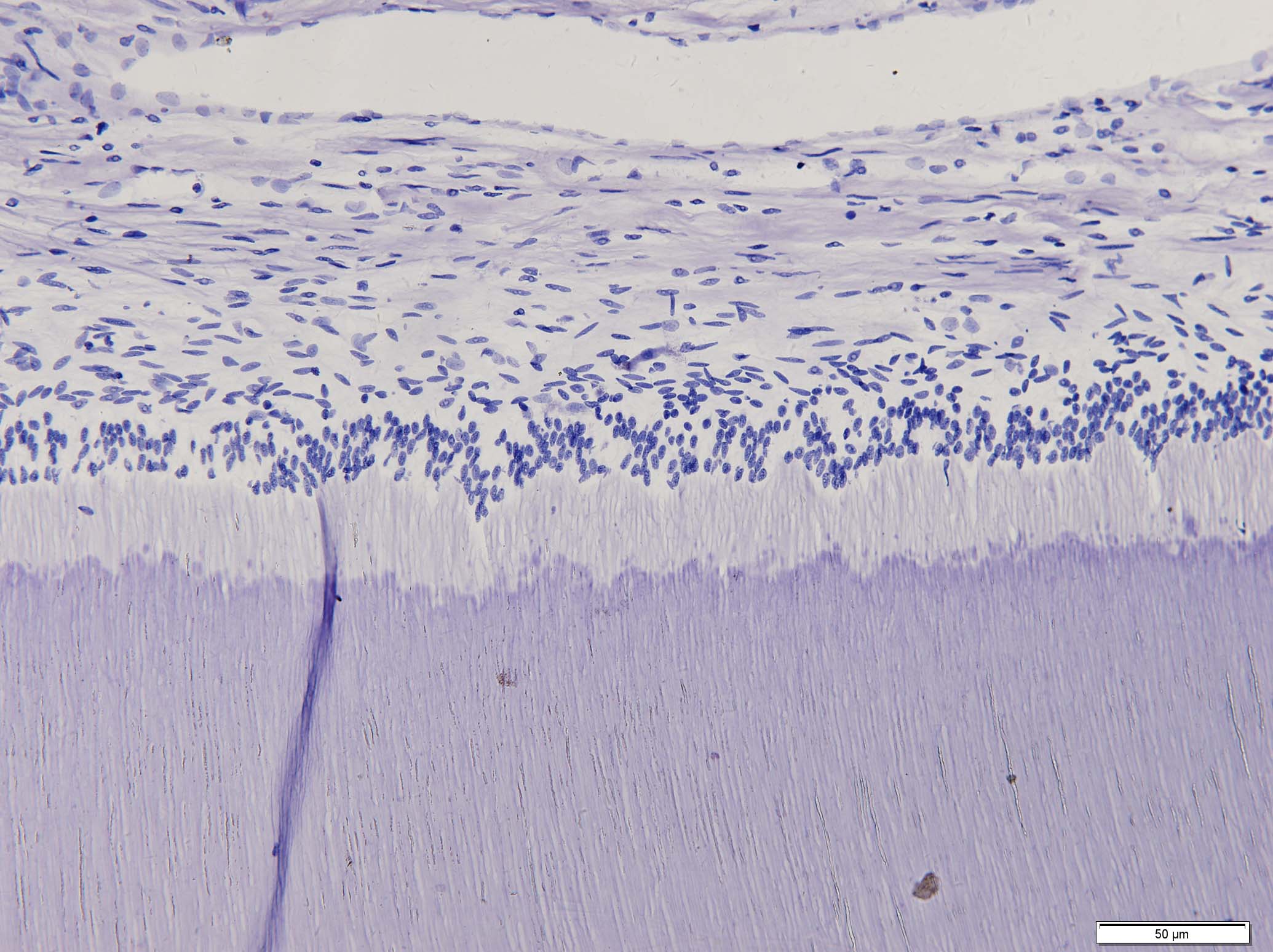

Supplement: Supplemental Information 3 — Sections were incubated without primary antibody, and then the same procedures were done. [file peerj-06-5808-s003.zip › negative control/Image_9238.jpg]

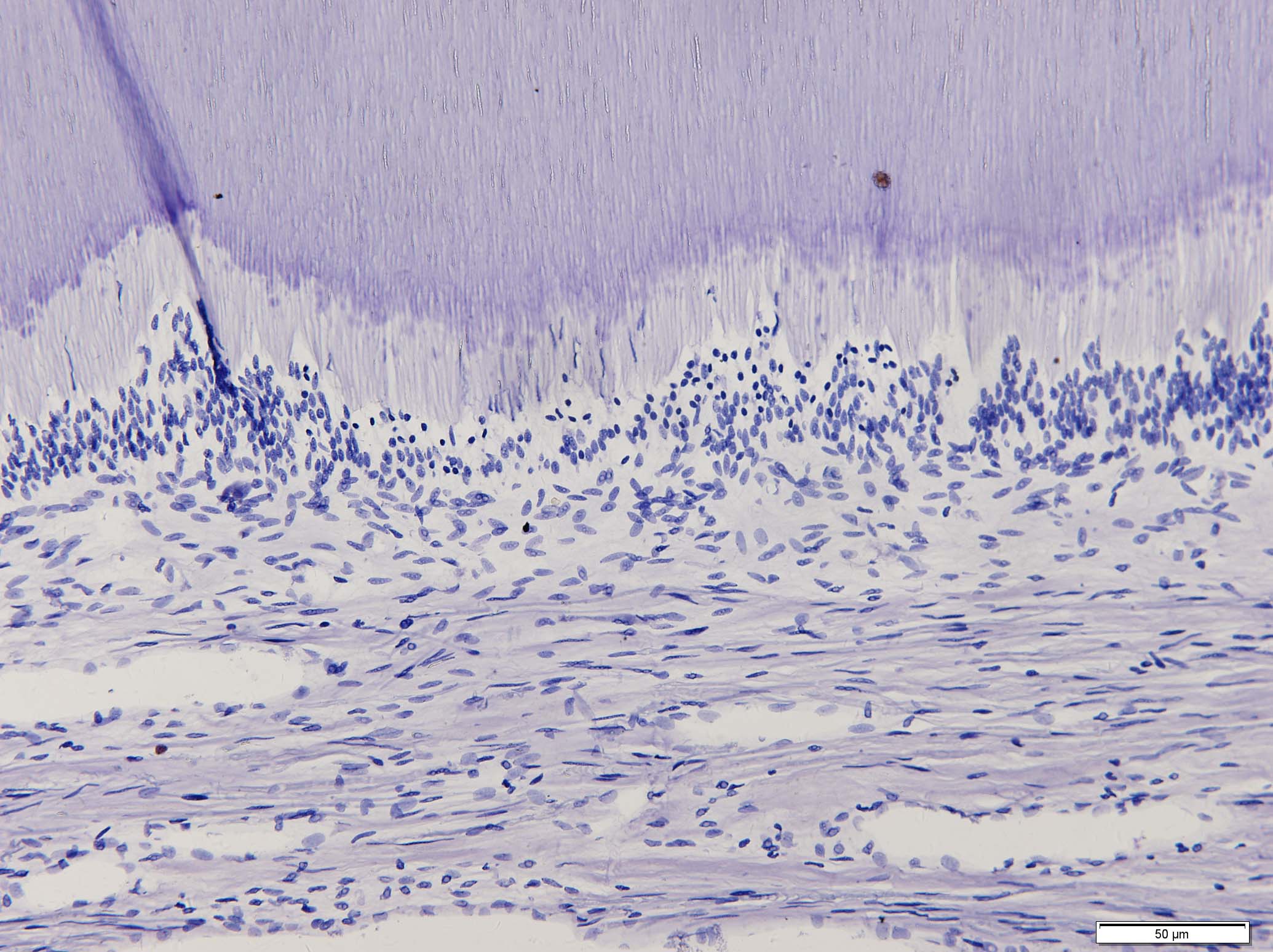

Supplement: Supplemental Information 3 — Sections were incubated without primary antibody, and then the same procedures were done. [file peerj-06-5808-s003.zip › negative control/Image_9240.jpg]

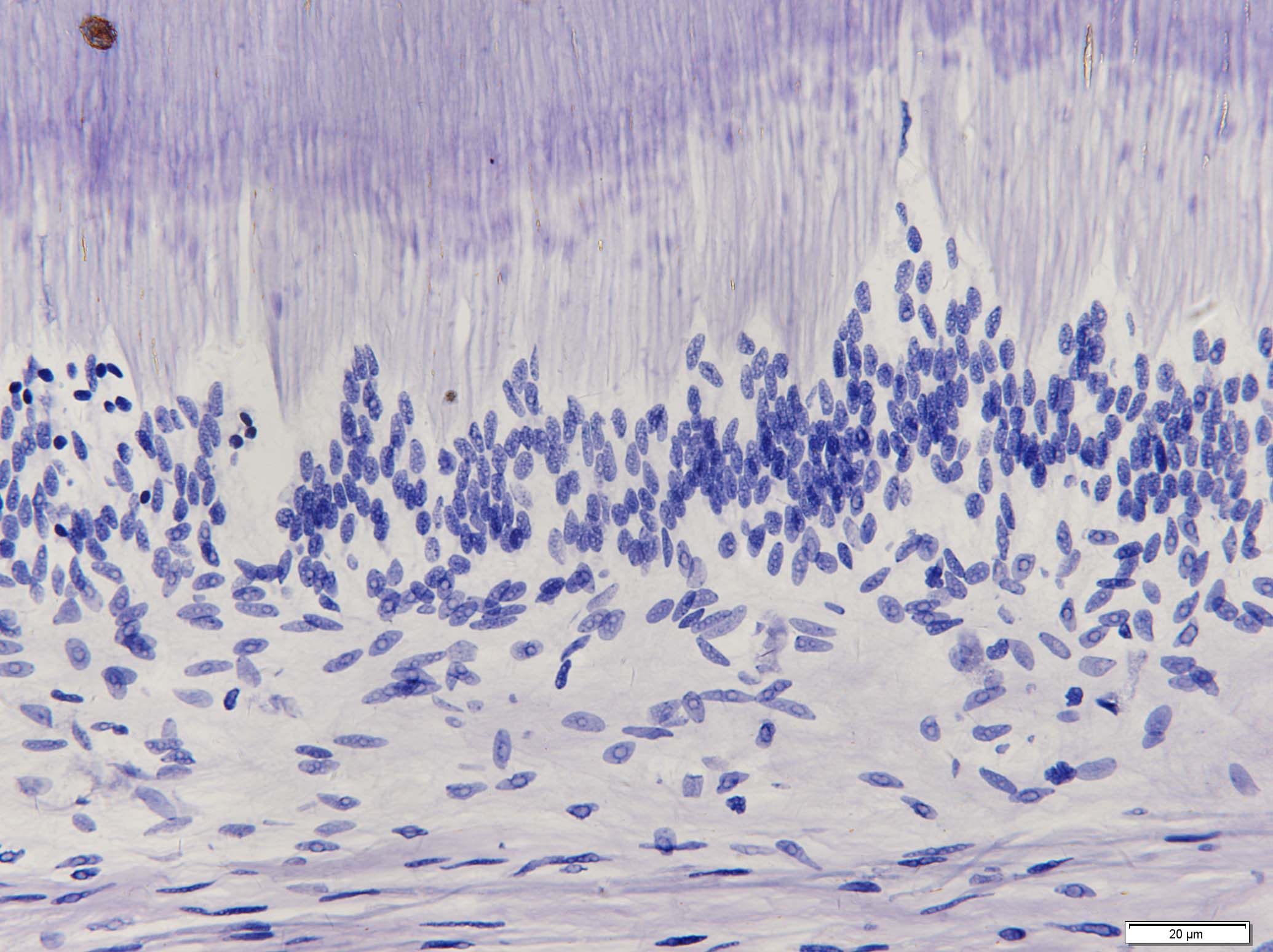

Supplement: Supplemental Information 3 — Sections were incubated without primary antibody, and then the same procedures were done. [file peerj-06-5808-s003.zip › negative control/Image_9241.jpg]

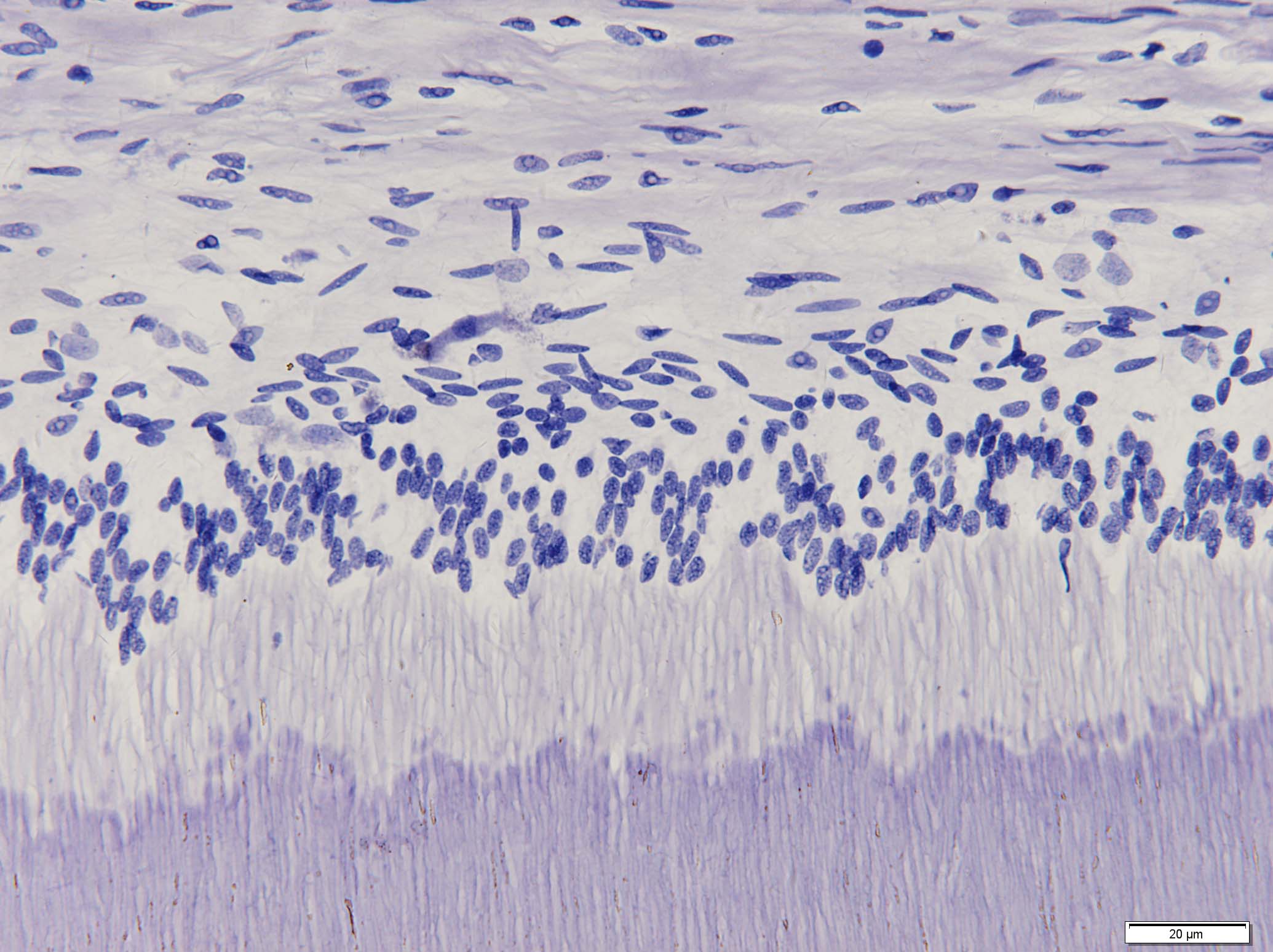

Supplement: Supplemental Information 3 — Sections were incubated without primary antibody, and then the same procedures were done. [file peerj-06-5808-s003.zip › negative control/Image_9242.jpg]

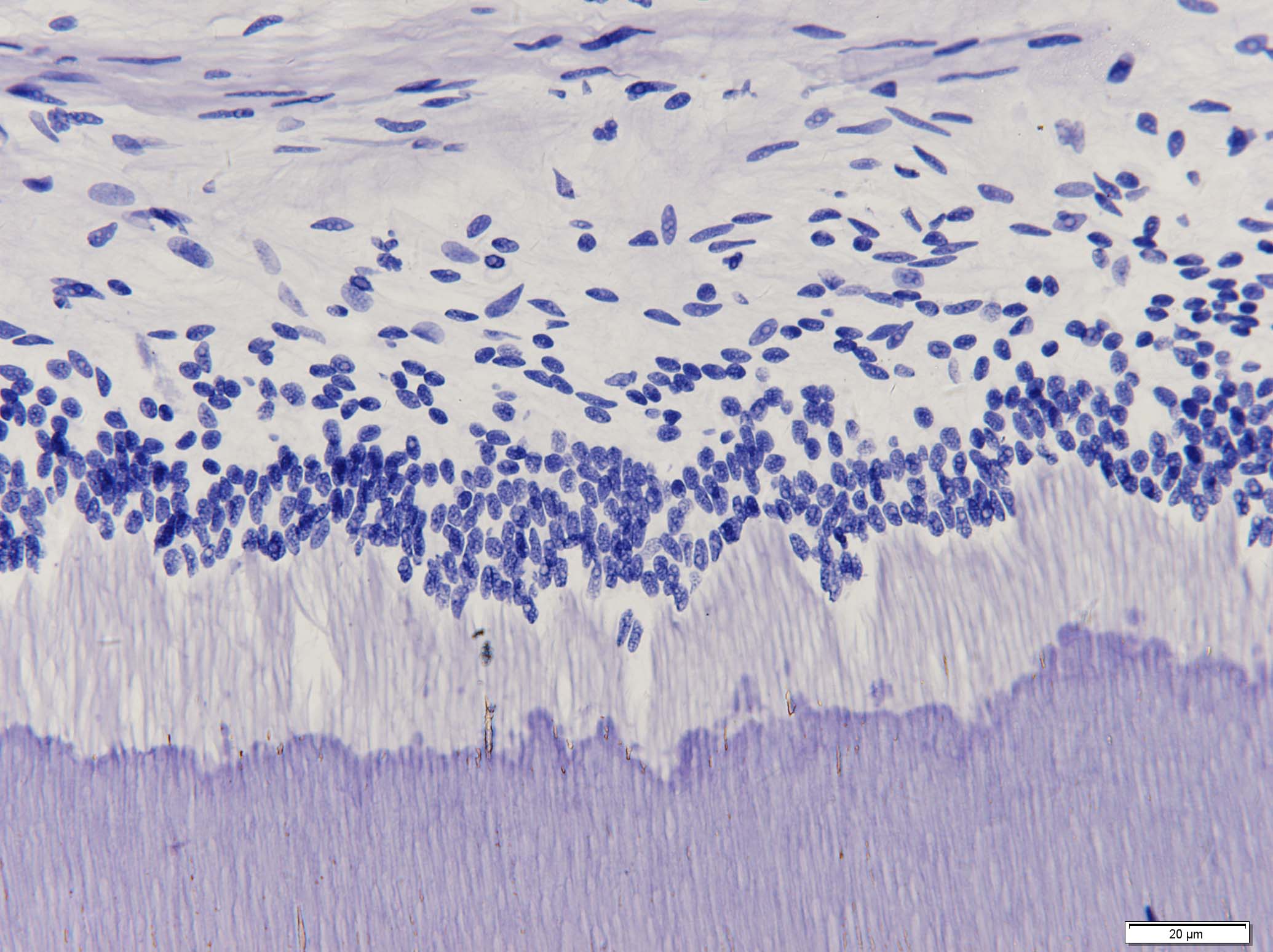

Supplement: Supplemental Information 3 — Sections were incubated without primary antibody, and then the same procedures were done. [file peerj-06-5808-s003.zip › negative control/Image_9243.jpg]

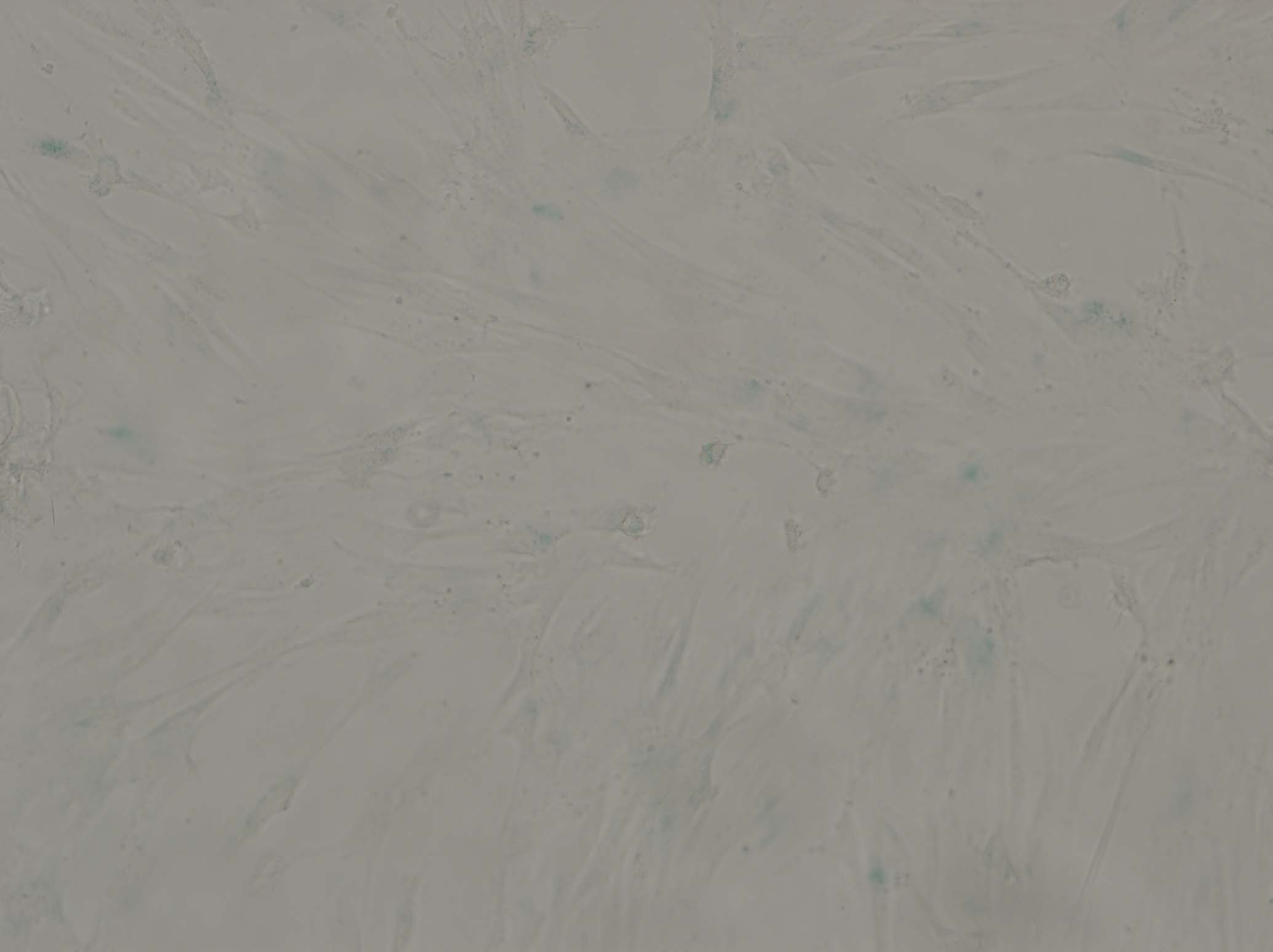

Supplement: Supplemental Information 4 — SA-β-Gal staining of human dental pulp cells with sclerostin overexpression and knockdown. [file peerj-06-5808-s004.zip › SA-B-Gal/SOST OVER/PCDH/═╝╧±_12073.jpg]

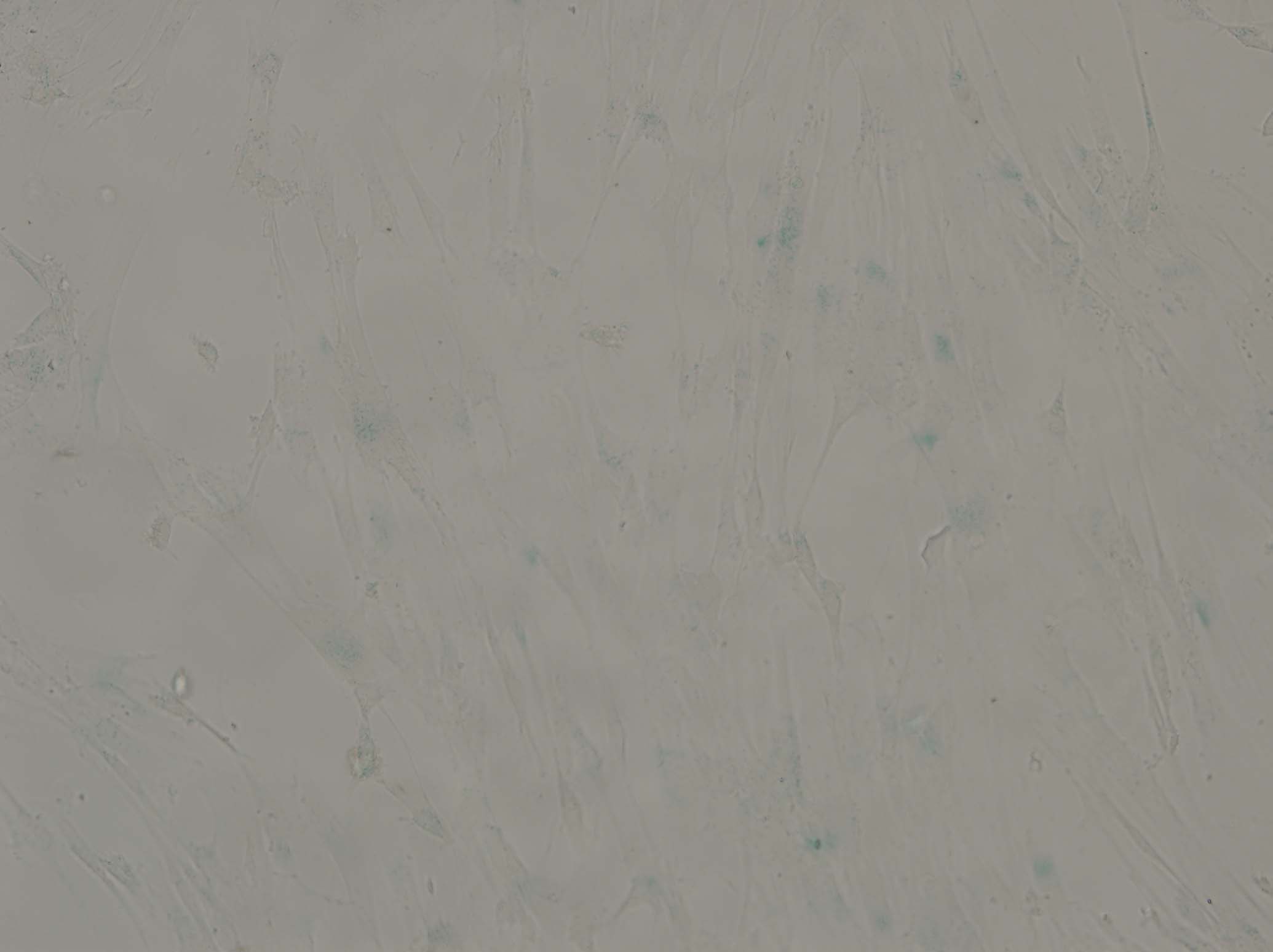

Supplement: Supplemental Information 4 — SA-β-Gal staining of human dental pulp cells with sclerostin overexpression and knockdown. [file peerj-06-5808-s004.zip › SA-B-Gal/SOST OVER/PCDH/═╝╧±_12074.jpg]

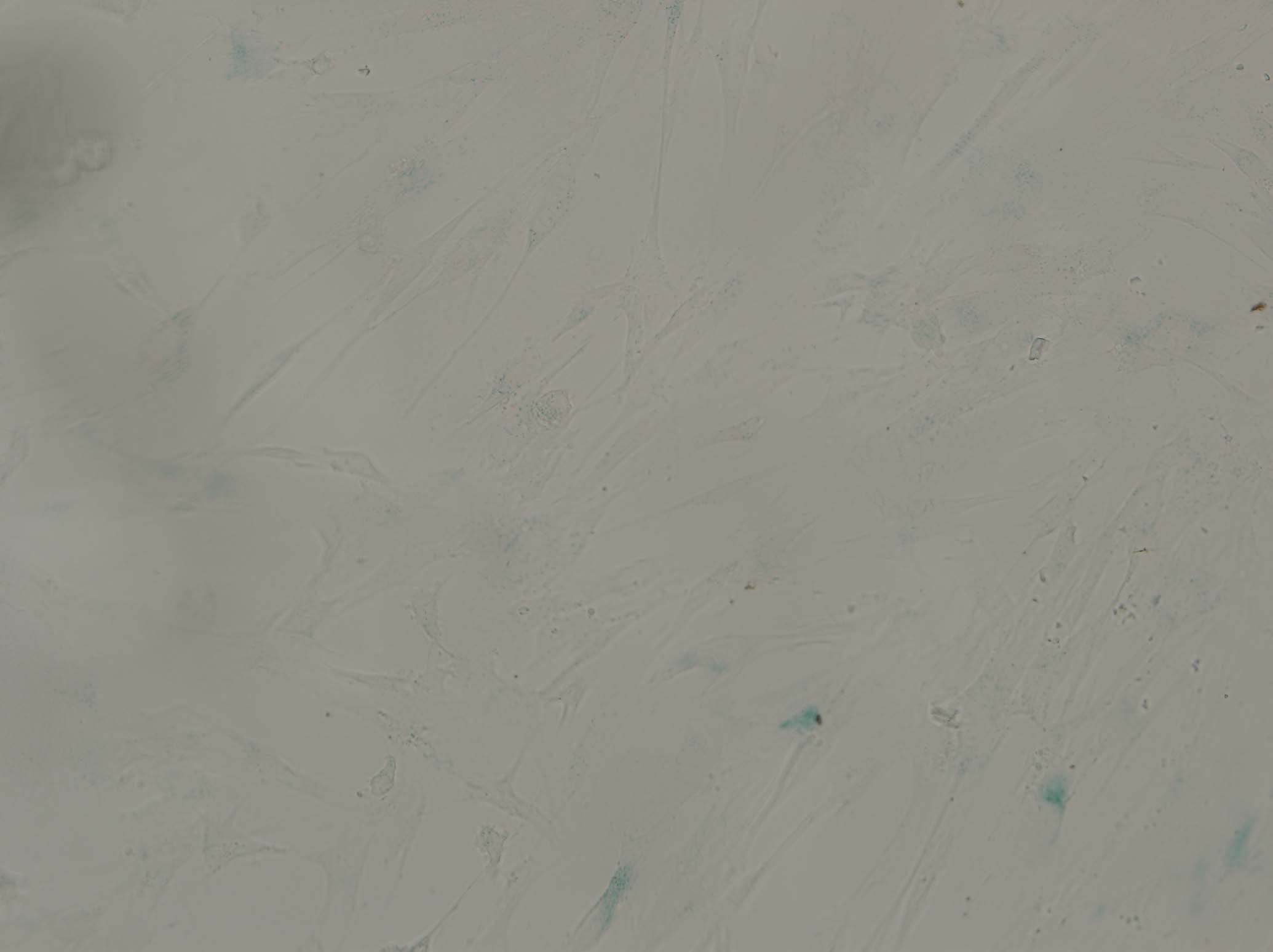

Supplement: Supplemental Information 4 — SA-β-Gal staining of human dental pulp cells with sclerostin overexpression and knockdown. [file peerj-06-5808-s004.zip › SA-B-Gal/SOST OVER/PCDH/═╝╧±_12075.jpg]

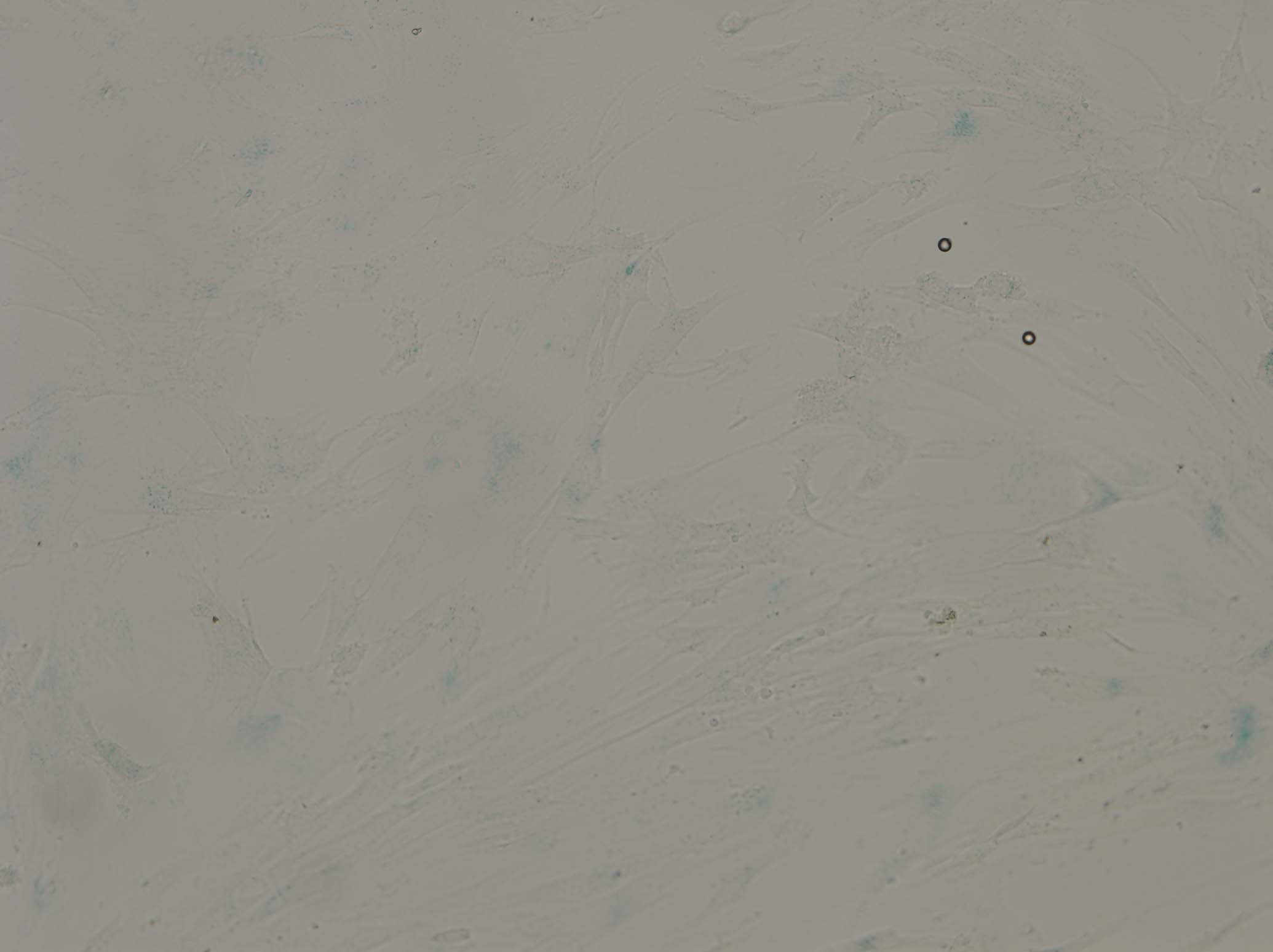

Supplement: Supplemental Information 4 — SA-β-Gal staining of human dental pulp cells with sclerostin overexpression and knockdown. [file peerj-06-5808-s004.zip › SA-B-Gal/SOST OVER/PCDH/═╝╧±_12076.jpg]

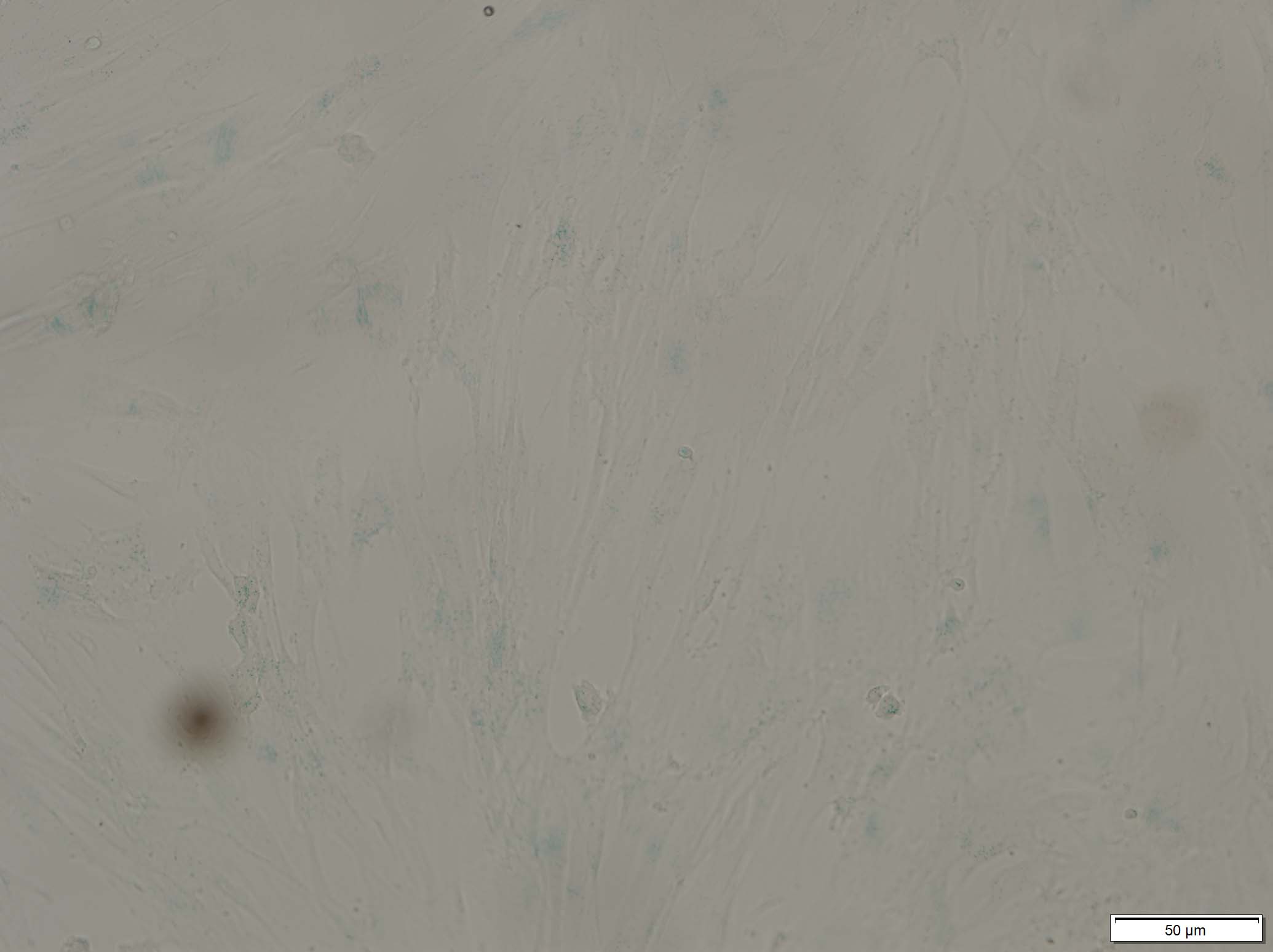

Supplement: Supplemental Information 4 — SA-β-Gal staining of human dental pulp cells with sclerostin overexpression and knockdown. [file peerj-06-5808-s004.zip › SA-B-Gal/SOST OVER/PCDH/═╝╧±_12077.jpg]

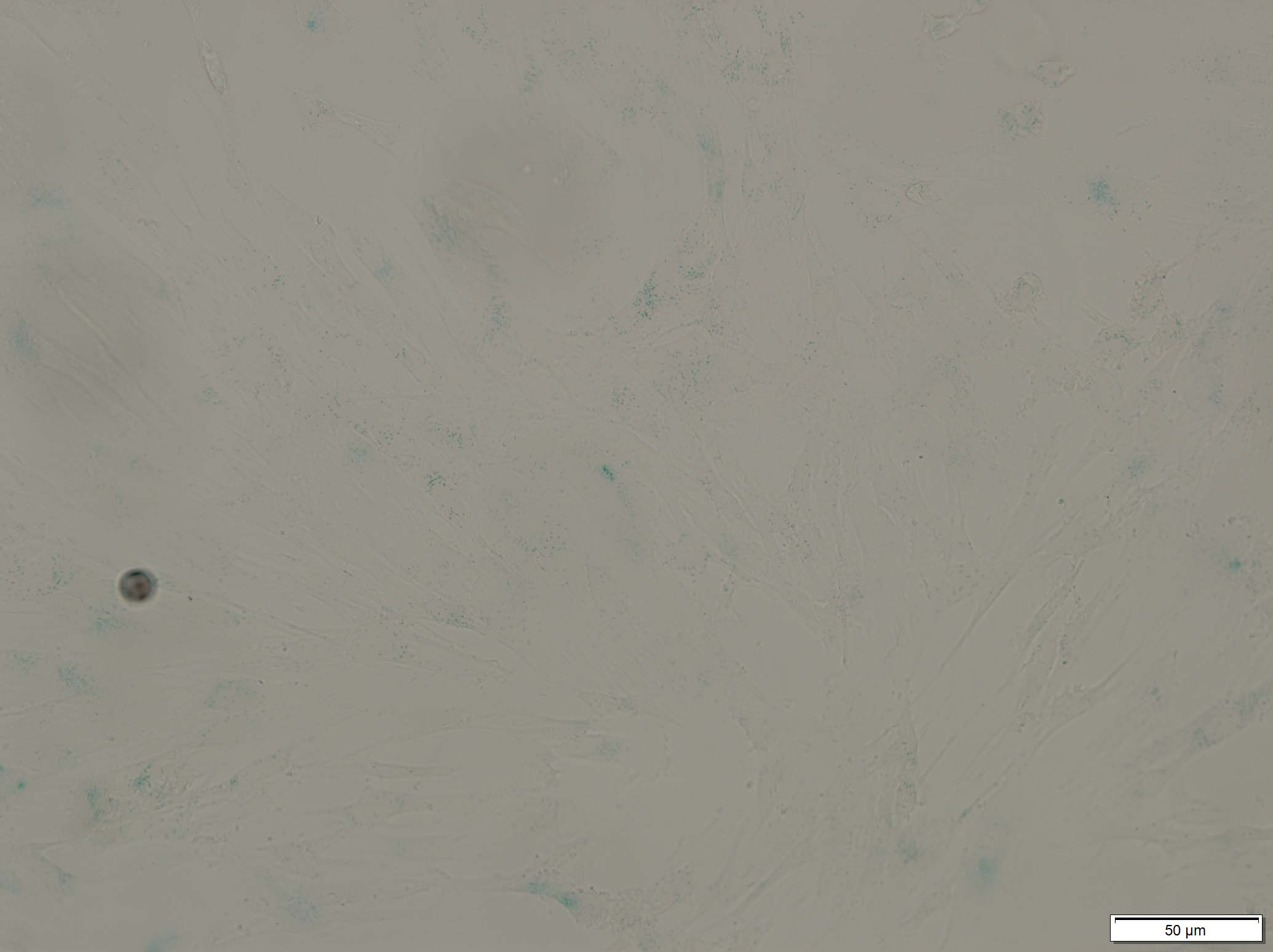

Supplement: Supplemental Information 4 — SA-β-Gal staining of human dental pulp cells with sclerostin overexpression and knockdown. [file peerj-06-5808-s004.zip › SA-B-Gal/SOST OVER/PCDH/═╝╧±_12078.jpg]

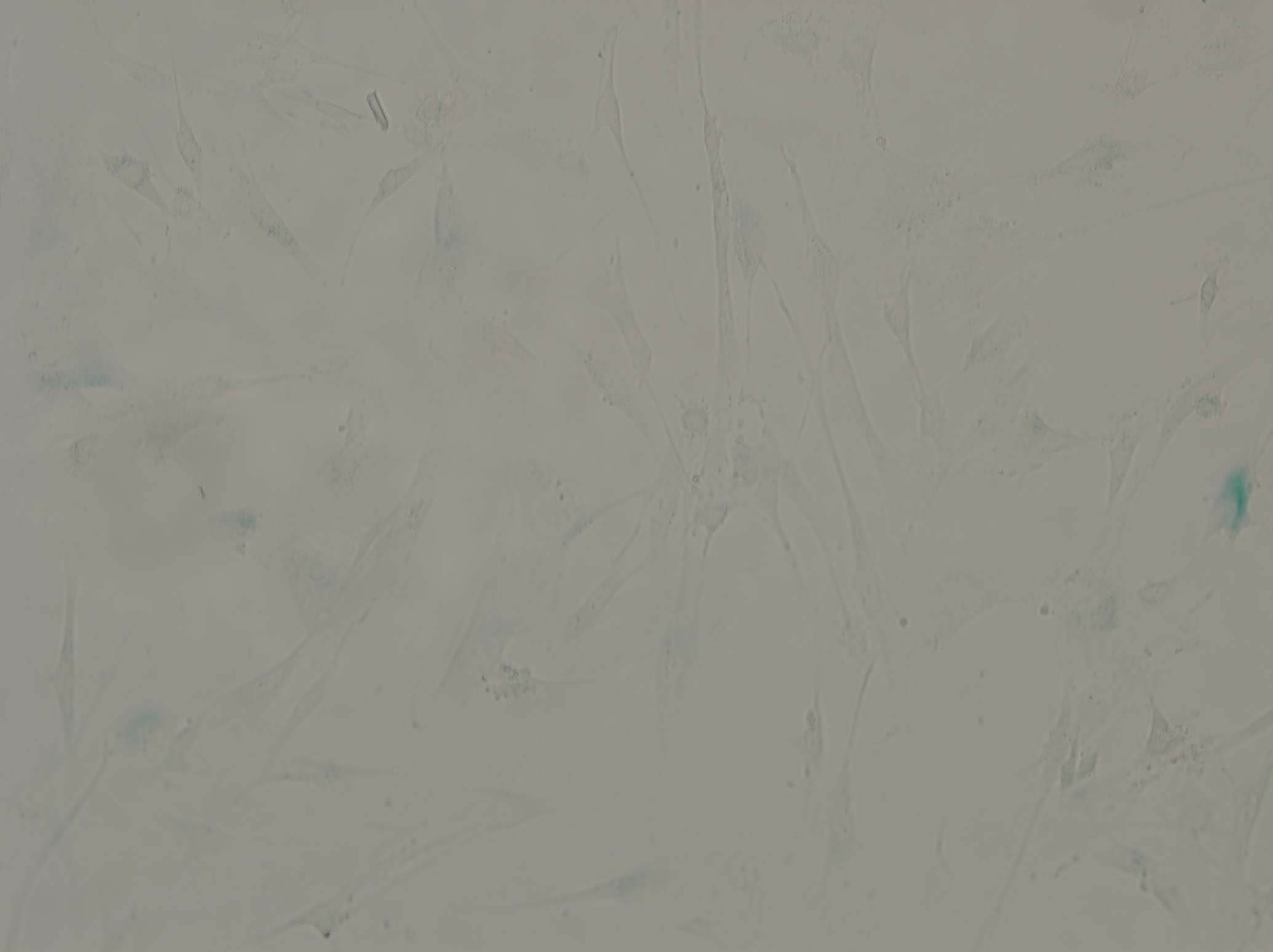

Supplement: Supplemental Information 4 — SA-β-Gal staining of human dental pulp cells with sclerostin overexpression and knockdown. [file peerj-06-5808-s004.zip › SA-B-Gal/SOST OVER/PCDH/═╝╧±_12083.jpg]

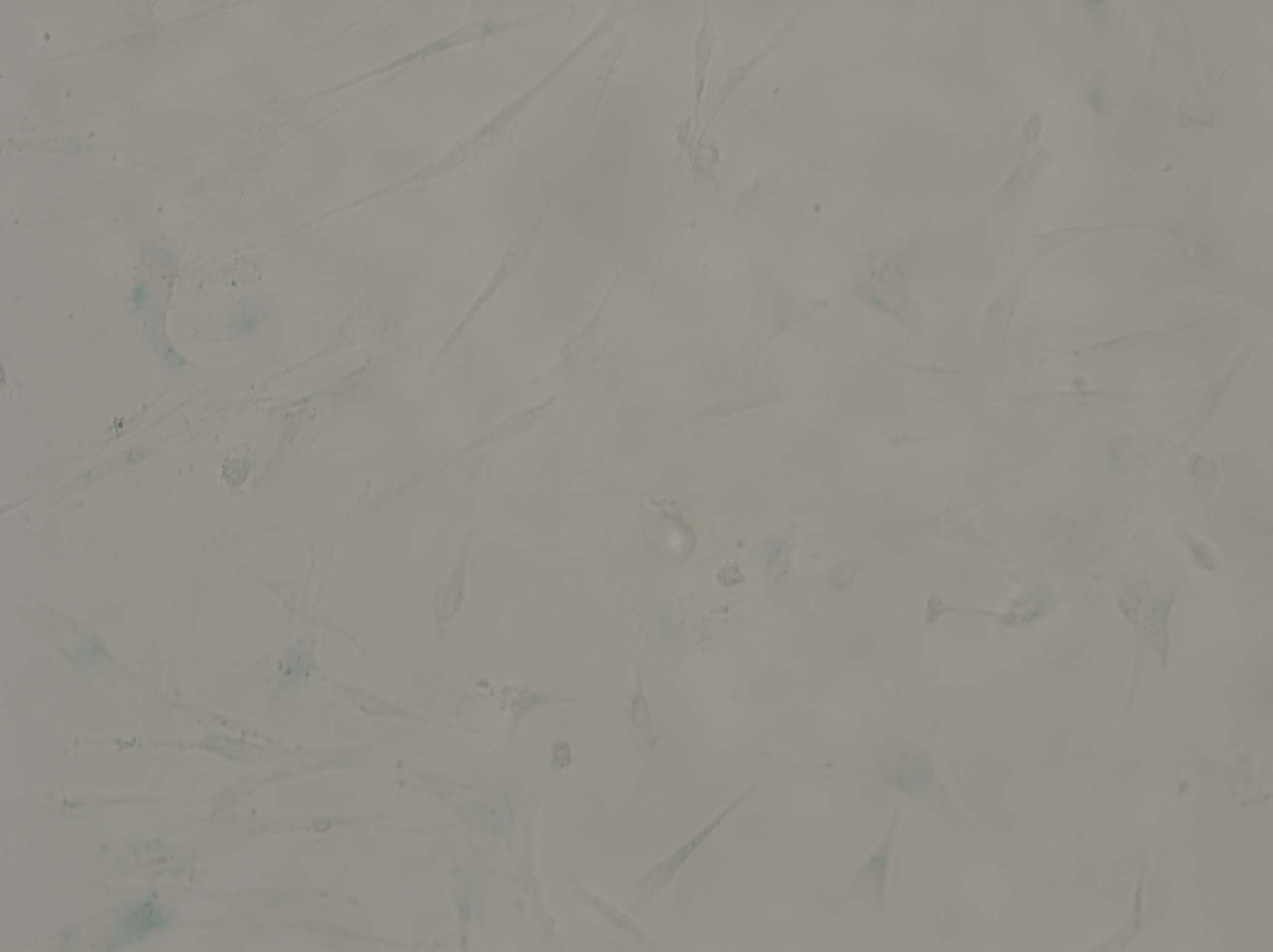

Supplement: Supplemental Information 4 — SA-β-Gal staining of human dental pulp cells with sclerostin overexpression and knockdown. [file peerj-06-5808-s004.zip › SA-B-Gal/SOST OVER/PCDH/═╝╧±_12084.jpg]

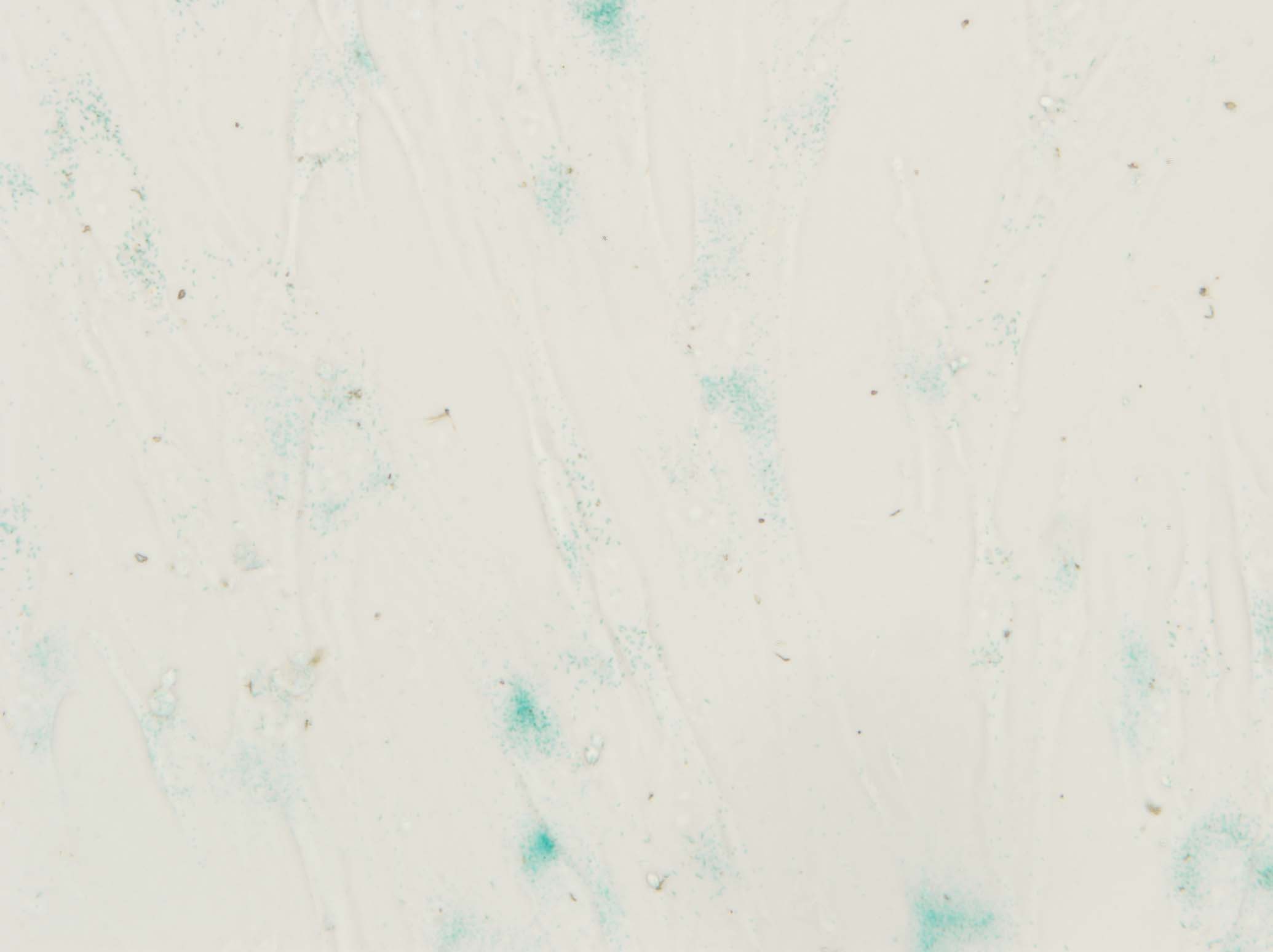

Supplement: Supplemental Information 4 — SA-β-Gal staining of human dental pulp cells with sclerostin overexpression and knockdown. [file peerj-06-5808-s004.zip › SA-B-Gal/SOST OVER/PCDH/═╝╧±_13413.jpg]

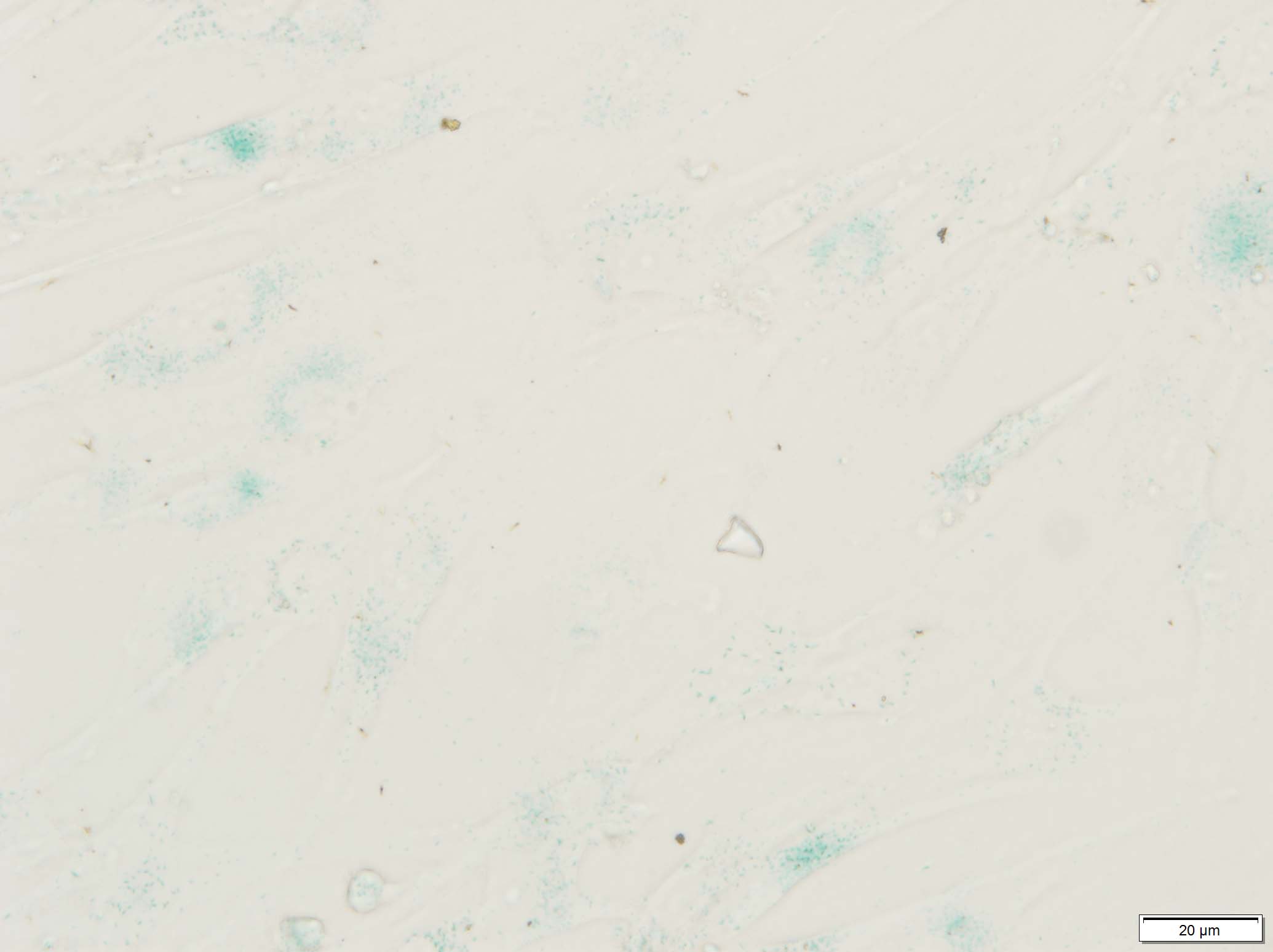

Supplement: Supplemental Information 4 — SA-β-Gal staining of human dental pulp cells with sclerostin overexpression and knockdown. [file peerj-06-5808-s004.zip › SA-B-Gal/SOST OVER/PCDH/═╝╧±_13414.jpg]

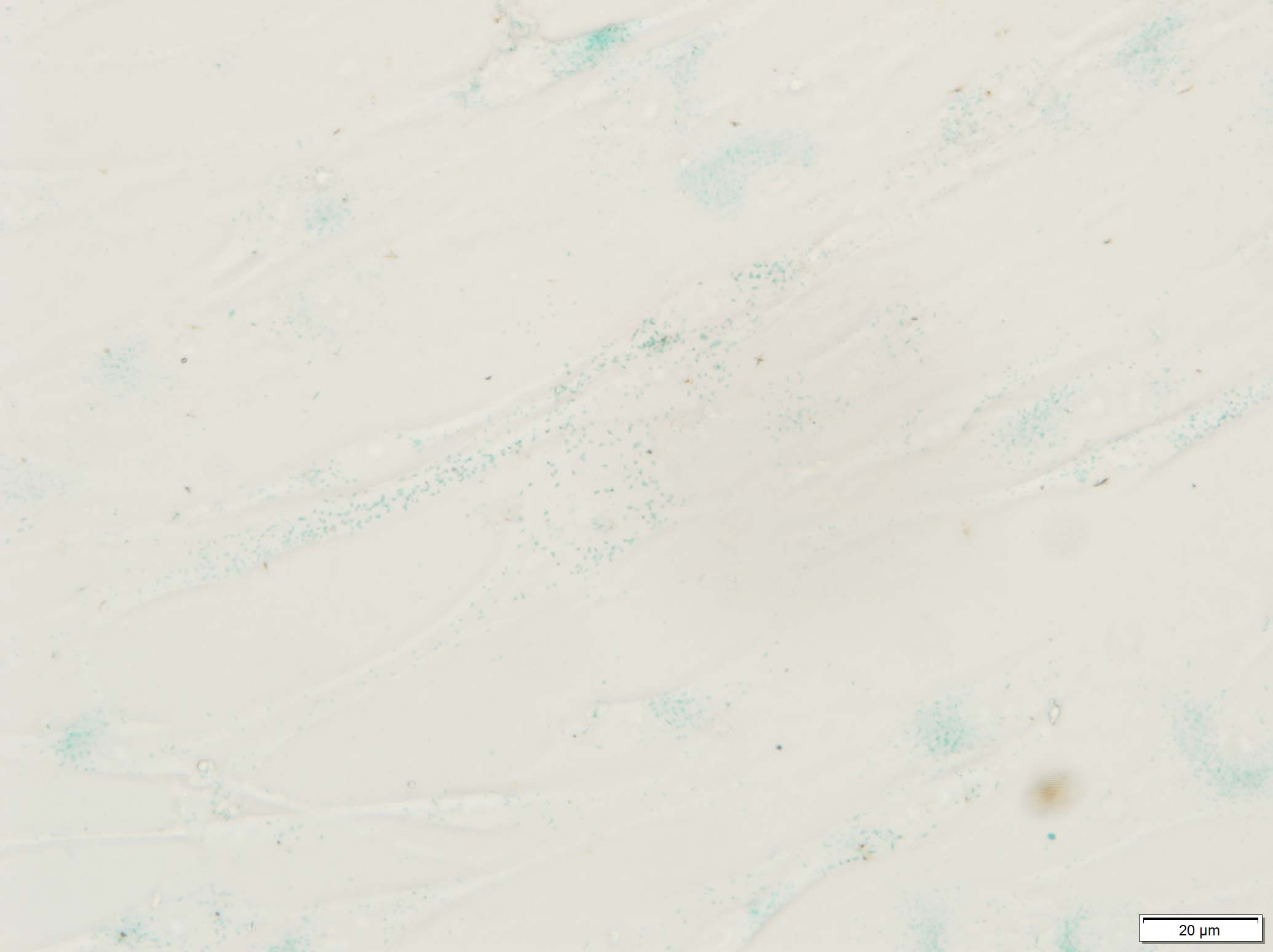

Supplement: Supplemental Information 4 — SA-β-Gal staining of human dental pulp cells with sclerostin overexpression and knockdown. [file peerj-06-5808-s004.zip › SA-B-Gal/SOST OVER/PCDH/═╝╧±_13415.jpg]

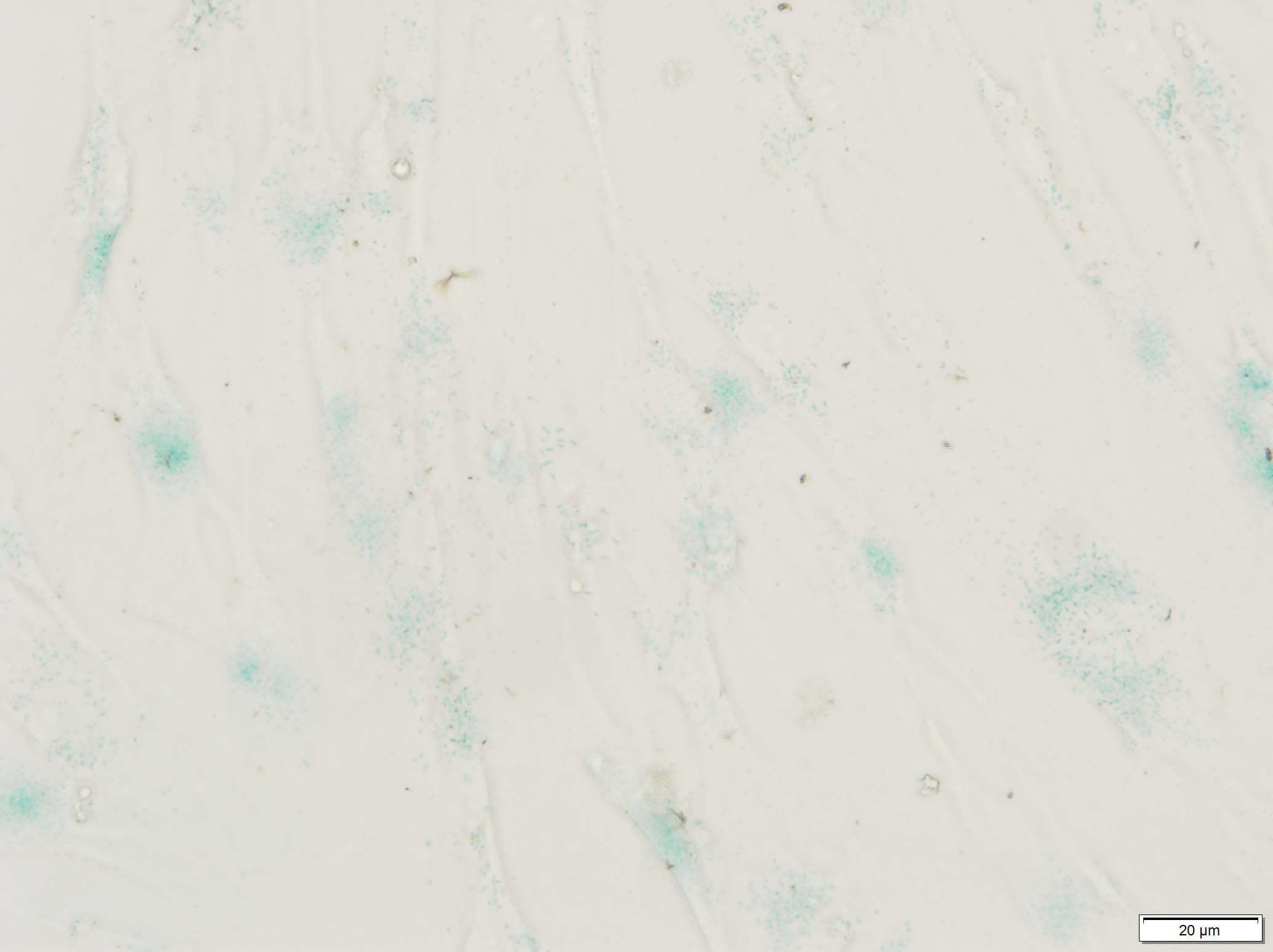

Supplement: Supplemental Information 4 — SA-β-Gal staining of human dental pulp cells with sclerostin overexpression and knockdown. [file peerj-06-5808-s004.zip › SA-B-Gal/SOST OVER/PCDH/═╝╧±_13416.jpg]

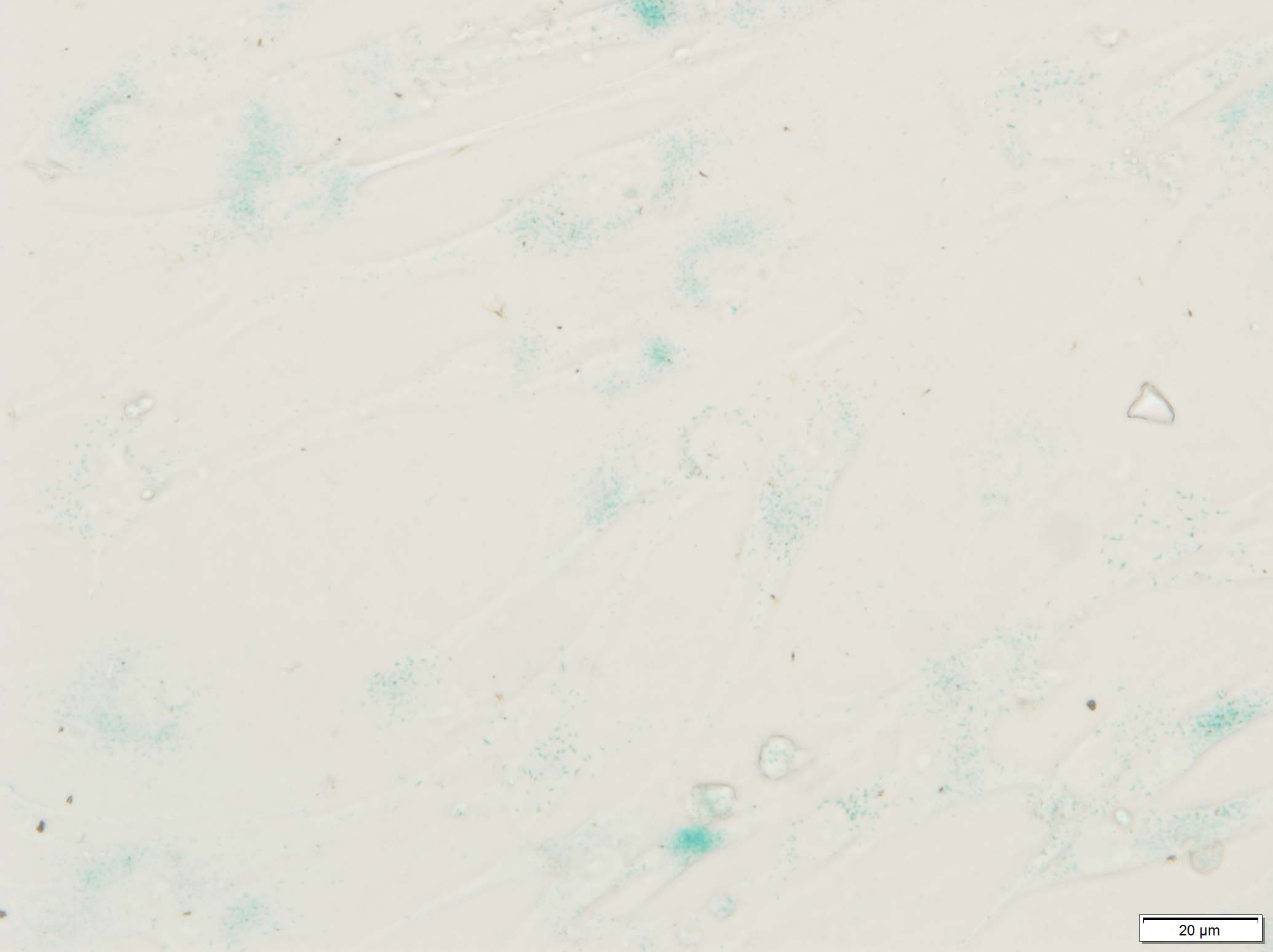

Supplement: Supplemental Information 4 — SA-β-Gal staining of human dental pulp cells with sclerostin overexpression and knockdown. [file peerj-06-5808-s004.zip › SA-B-Gal/SOST OVER/PCDH/═╝╧±_13417.jpg]

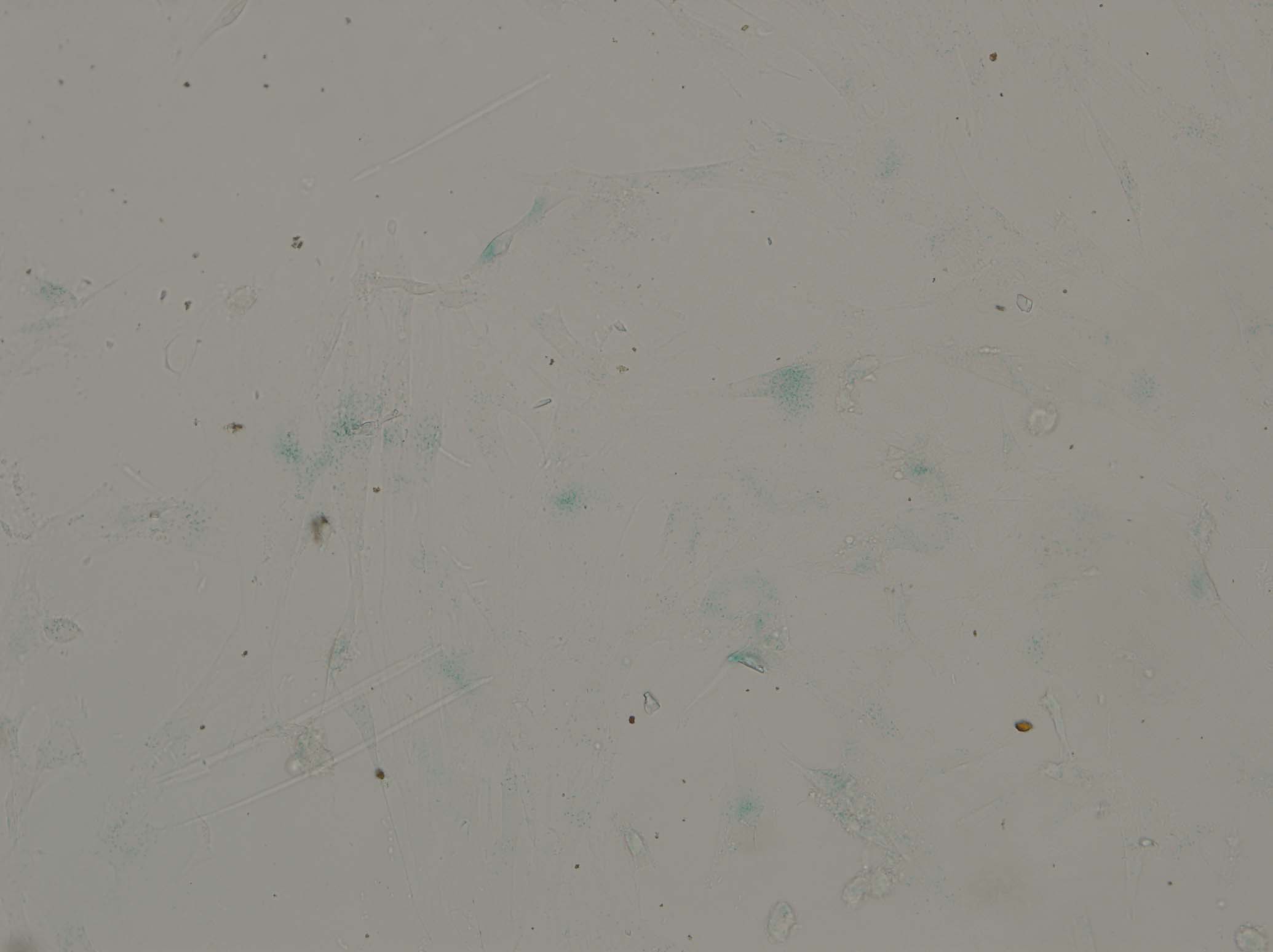

Supplement: Supplemental Information 4 — SA-β-Gal staining of human dental pulp cells with sclerostin overexpression and knockdown. [file peerj-06-5808-s004.zip › SA-B-Gal/SOST OVER/SOST-OVER/═╝╧±_12056.jpg]

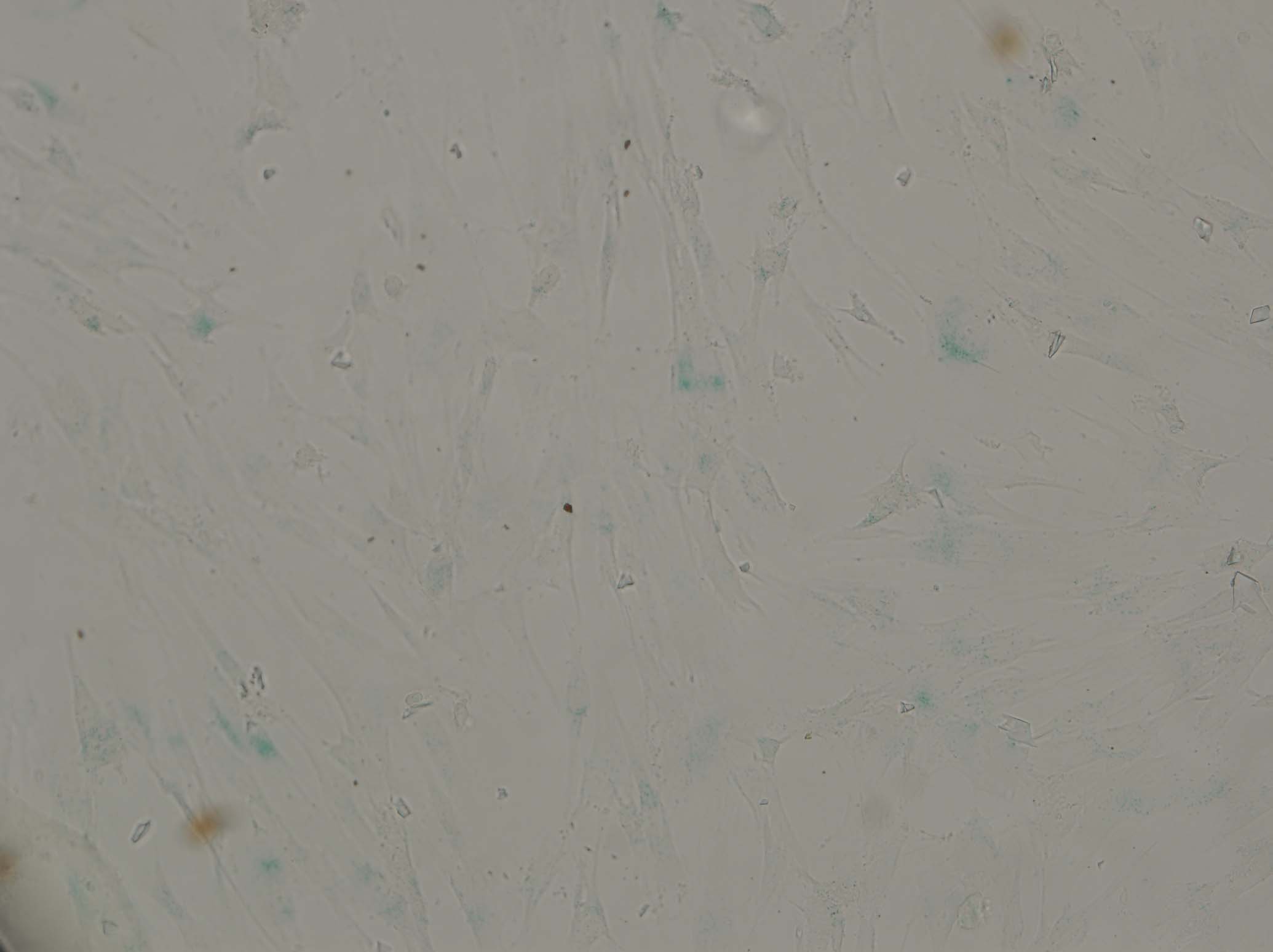

Supplement: Supplemental Information 4 — SA-β-Gal staining of human dental pulp cells with sclerostin overexpression and knockdown. [file peerj-06-5808-s004.zip › SA-B-Gal/SOST OVER/SOST-OVER/═╝╧±_12057.jpg]

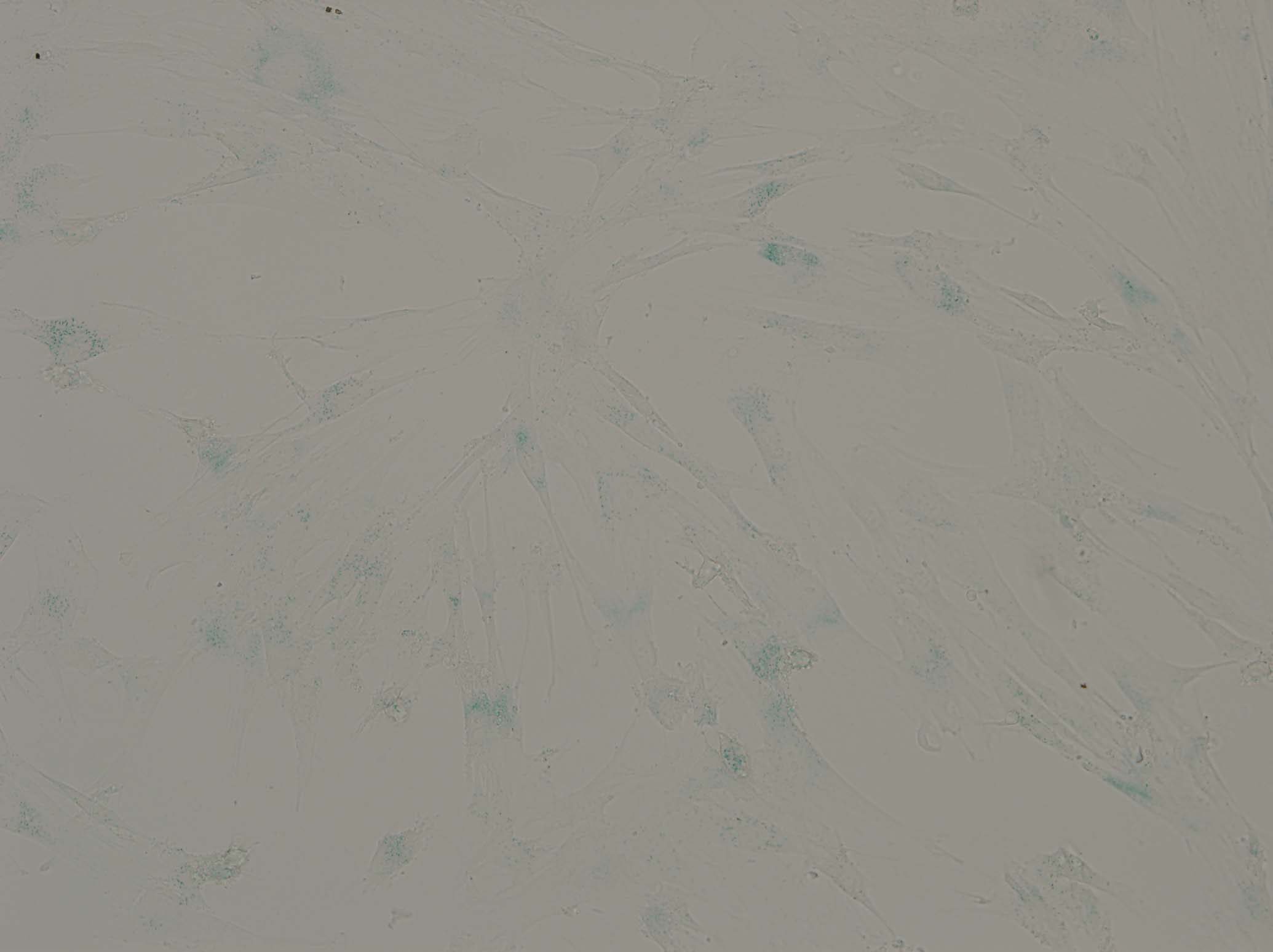

Supplement: Supplemental Information 4 — SA-β-Gal staining of human dental pulp cells with sclerostin overexpression and knockdown. [file peerj-06-5808-s004.zip › SA-B-Gal/SOST OVER/SOST-OVER/═╝╧±_12063.jpg]

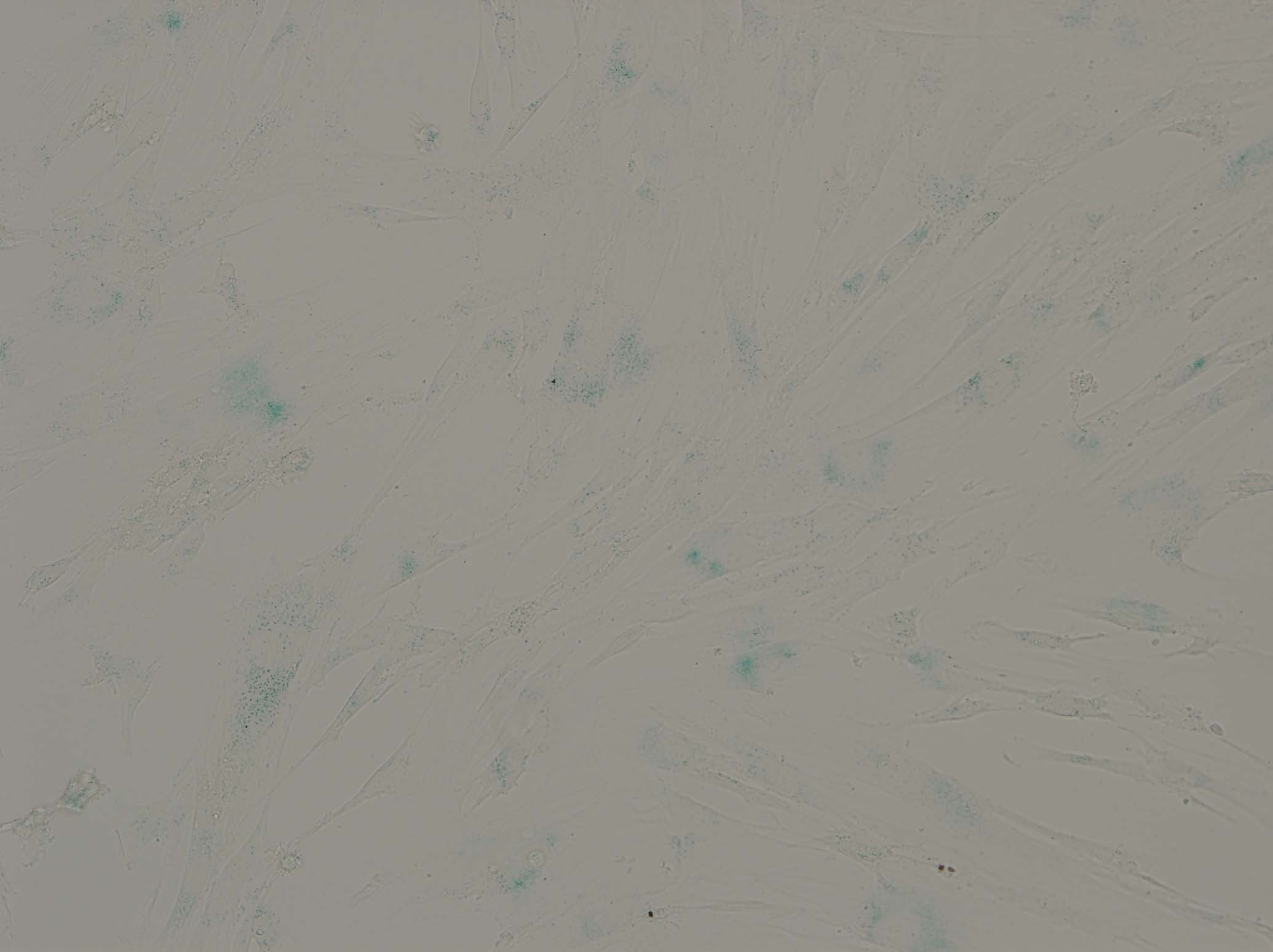

Supplement: Supplemental Information 4 — SA-β-Gal staining of human dental pulp cells with sclerostin overexpression and knockdown. [file peerj-06-5808-s004.zip › SA-B-Gal/SOST OVER/SOST-OVER/═╝╧±_12064.jpg]

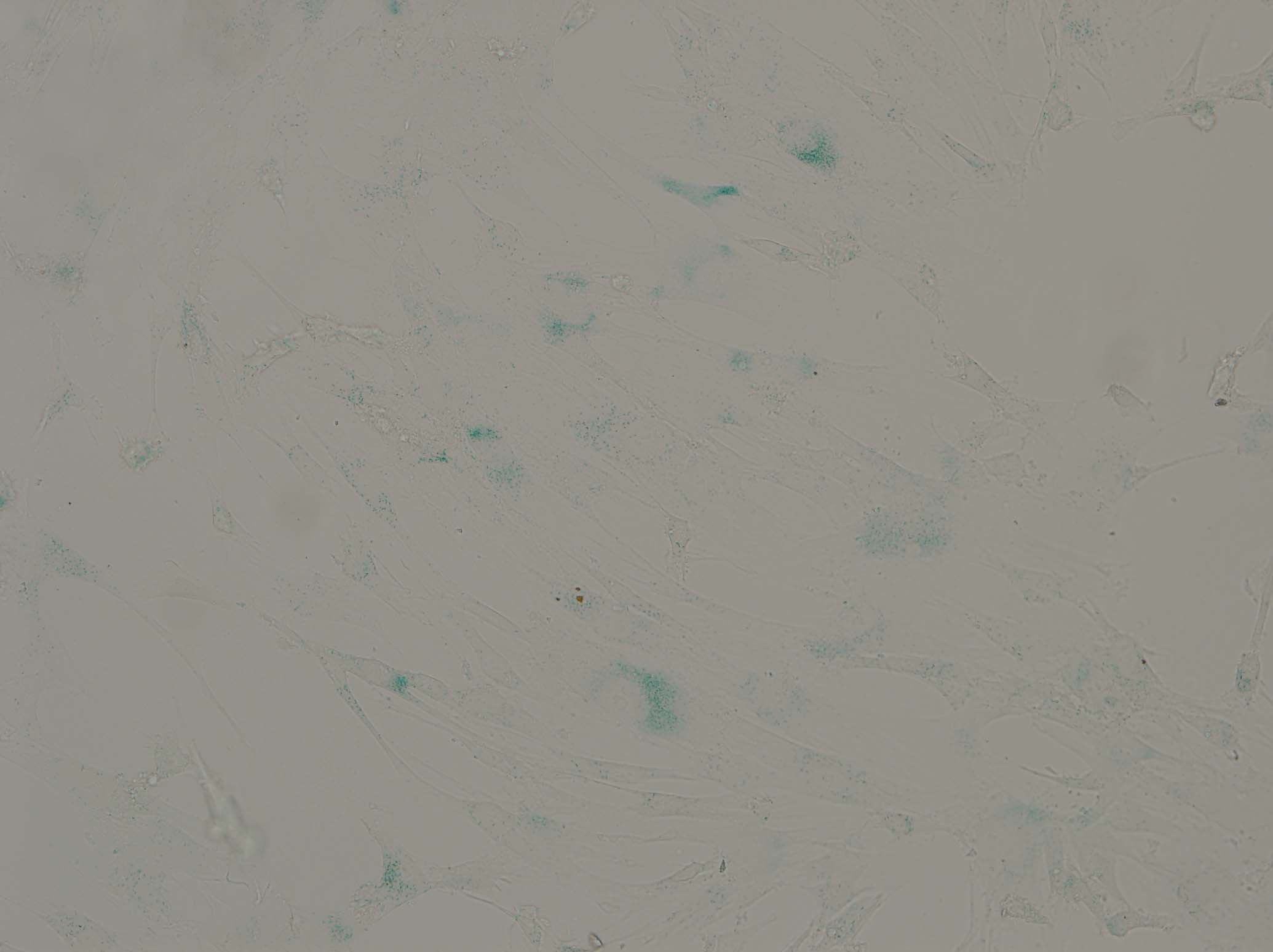

Supplement: Supplemental Information 4 — SA-β-Gal staining of human dental pulp cells with sclerostin overexpression and knockdown. [file peerj-06-5808-s004.zip › SA-B-Gal/SOST OVER/SOST-OVER/═╝╧±_12065.jpg]

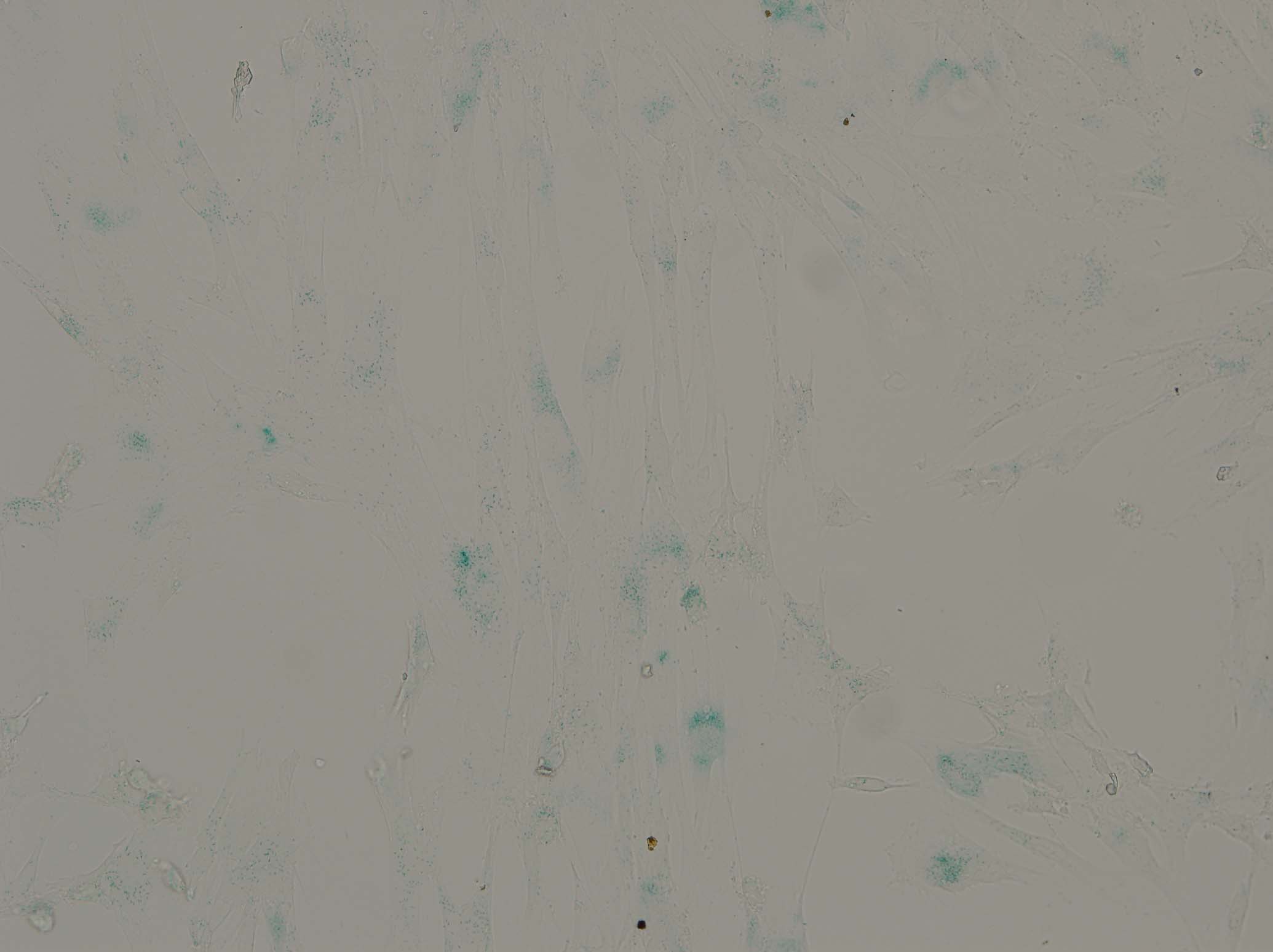

Supplement: Supplemental Information 4 — SA-β-Gal staining of human dental pulp cells with sclerostin overexpression and knockdown. [file peerj-06-5808-s004.zip › SA-B-Gal/SOST OVER/SOST-OVER/═╝╧±_12066.jpg]

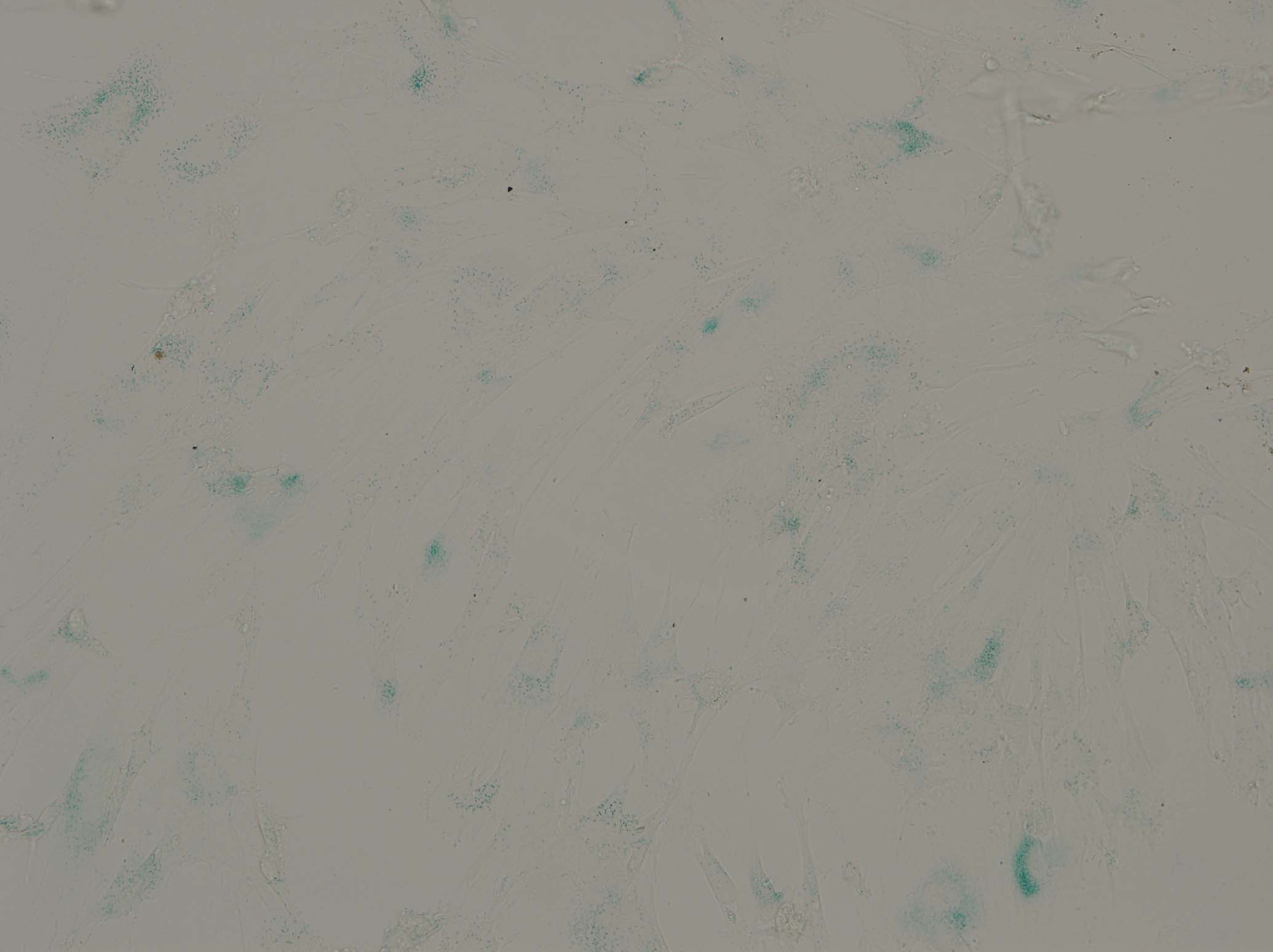

Supplement: Supplemental Information 4 — SA-β-Gal staining of human dental pulp cells with sclerostin overexpression and knockdown. [file peerj-06-5808-s004.zip › SA-B-Gal/SOST OVER/SOST-OVER/═╝╧±_12067.jpg]

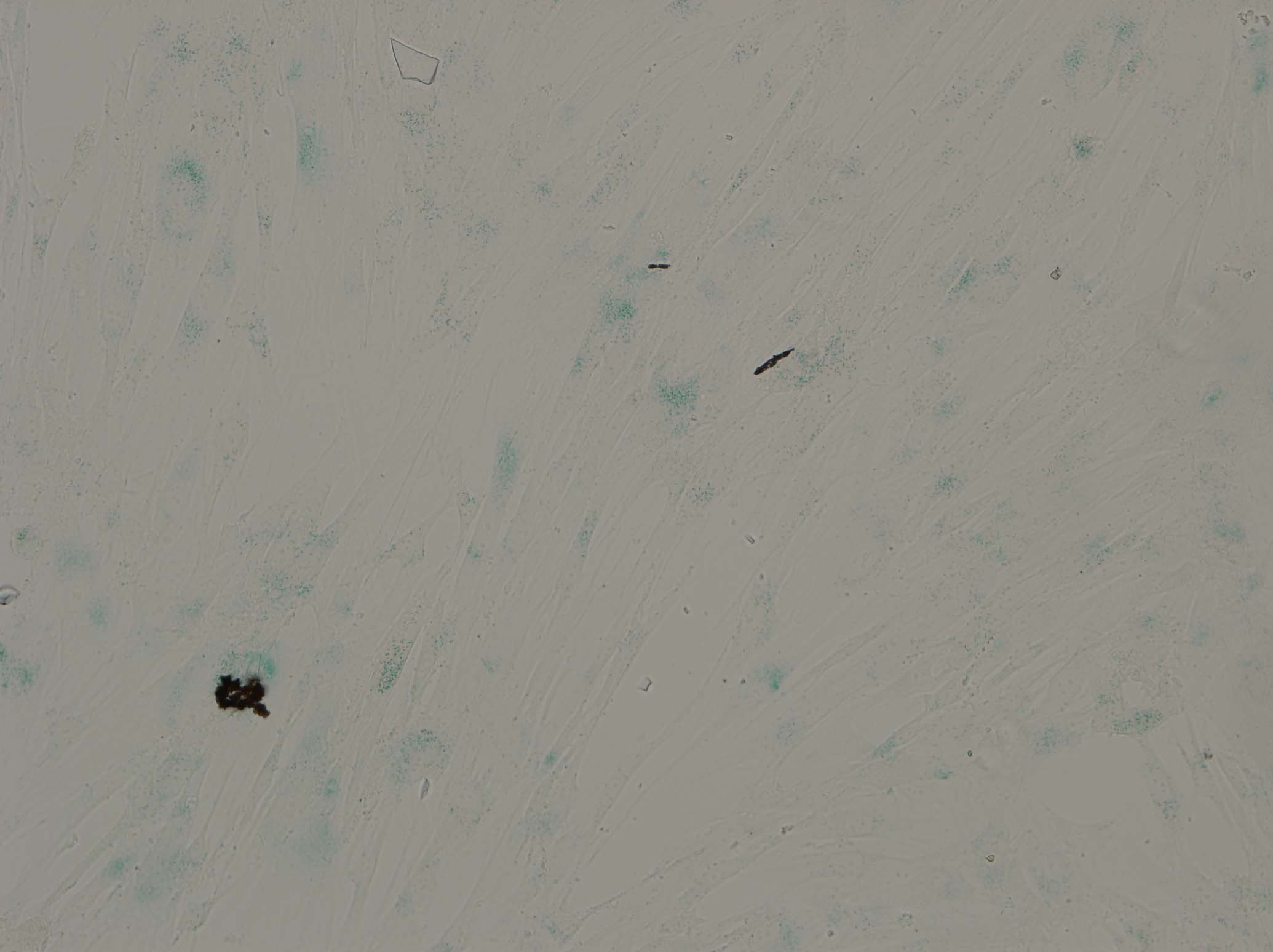

Supplement: Supplemental Information 4 — SA-β-Gal staining of human dental pulp cells with sclerostin overexpression and knockdown. [file peerj-06-5808-s004.zip › SA-B-Gal/SOST OVER/SOST-OVER/═╝╧±_12068.jpg]

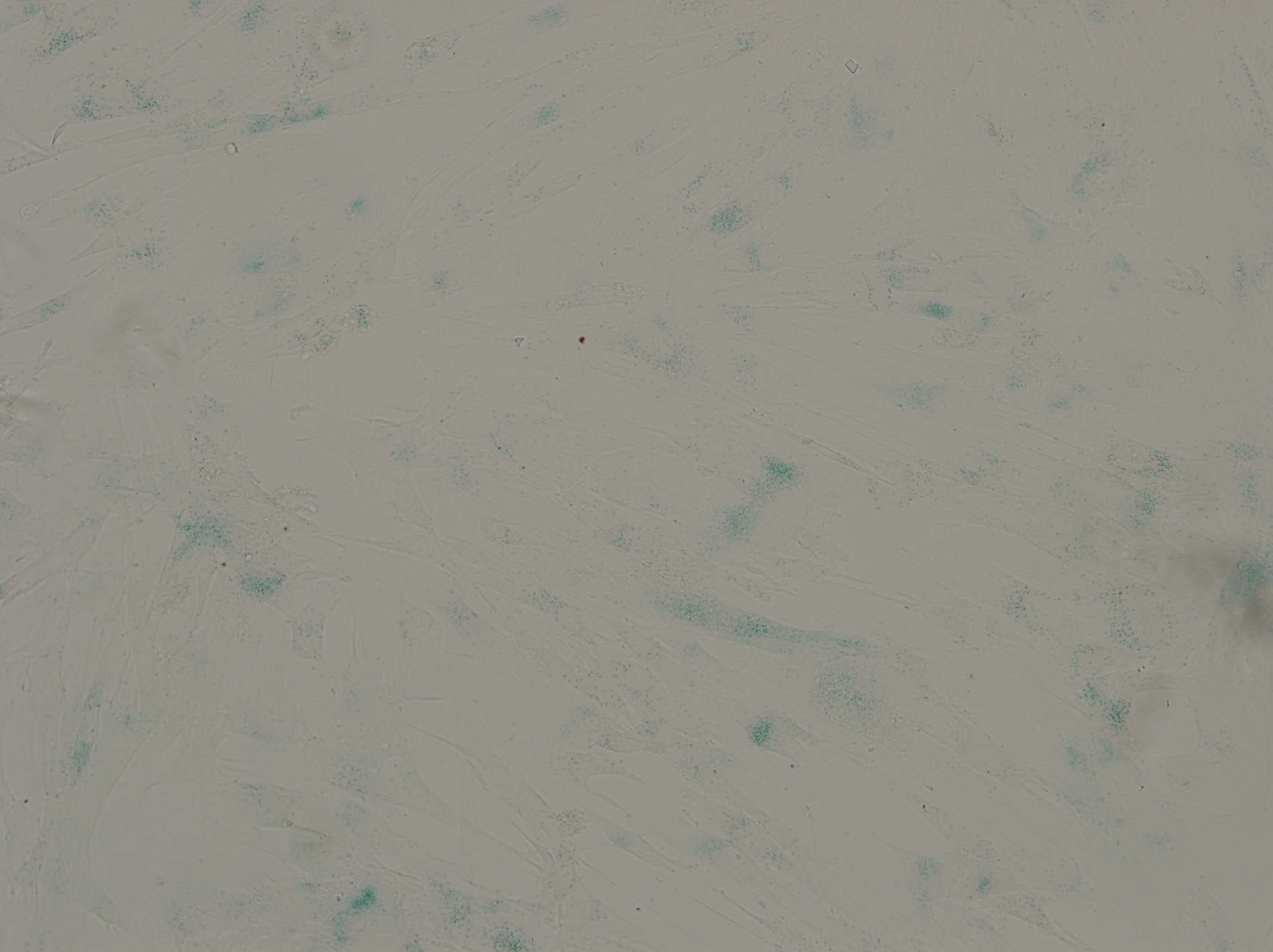

Supplement: Supplemental Information 4 — SA-β-Gal staining of human dental pulp cells with sclerostin overexpression and knockdown. [file peerj-06-5808-s004.zip › SA-B-Gal/SOST OVER/SOST-OVER/═╝╧±_12069.jpg]

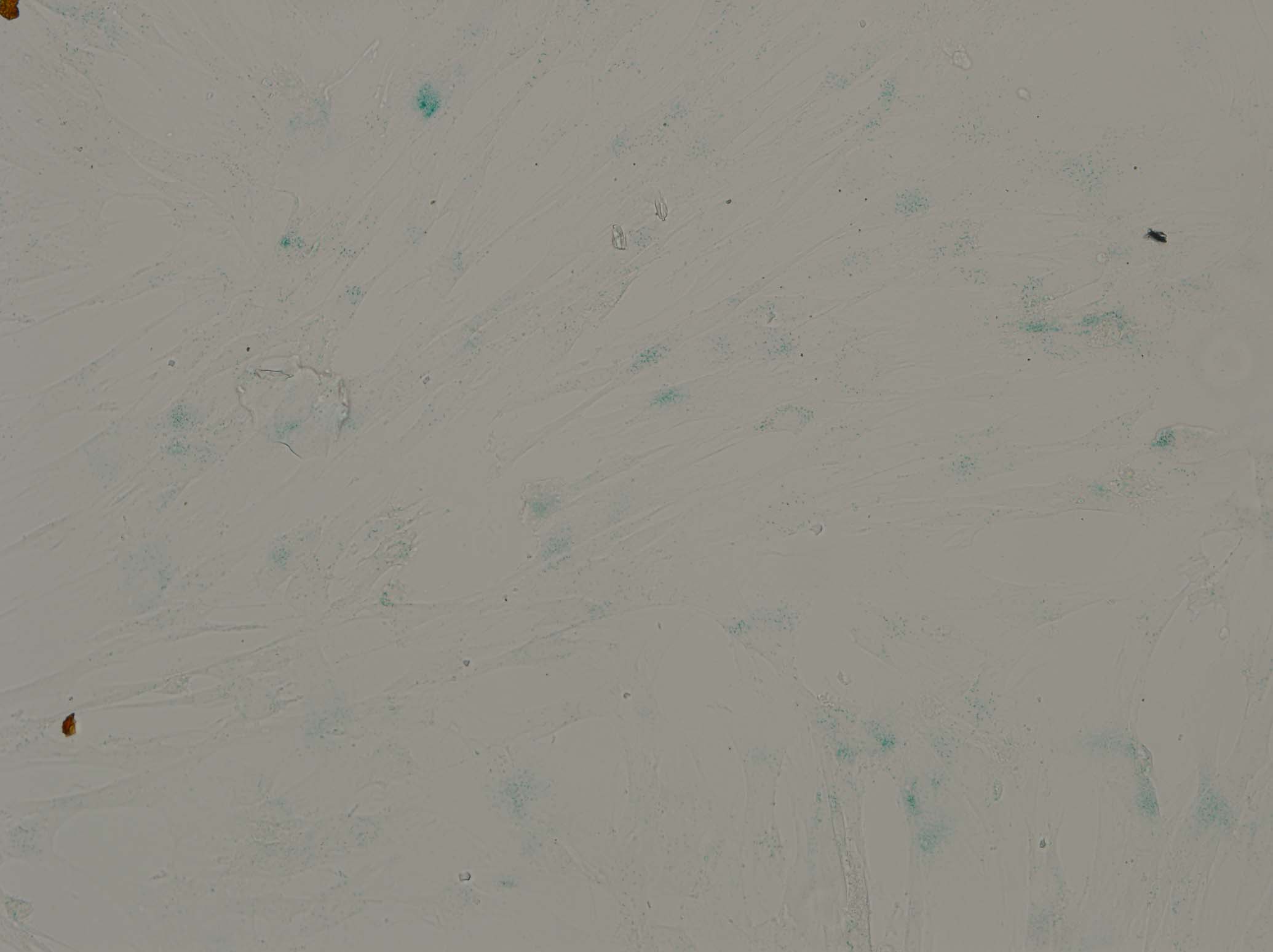

Supplement: Supplemental Information 4 — SA-β-Gal staining of human dental pulp cells with sclerostin overexpression and knockdown. [file peerj-06-5808-s004.zip › SA-B-Gal/SOST OVER/SOST-OVER/═╝╧±_12070.jpg]

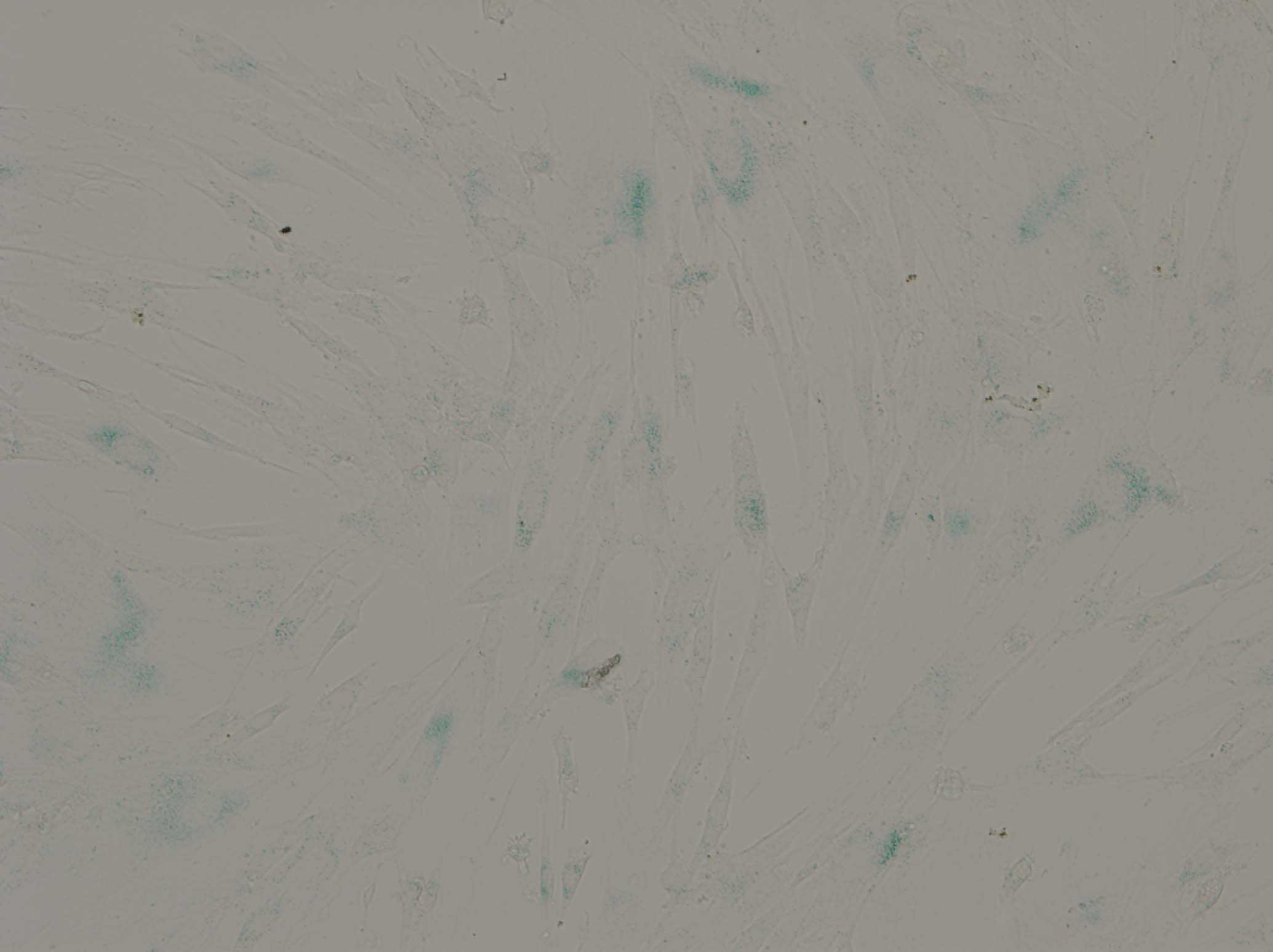

Supplement: Supplemental Information 4 — SA-β-Gal staining of human dental pulp cells with sclerostin overexpression and knockdown. [file peerj-06-5808-s004.zip › SA-B-Gal/SOST OVER/SOST-OVER/═╝╧±_12079.jpg]

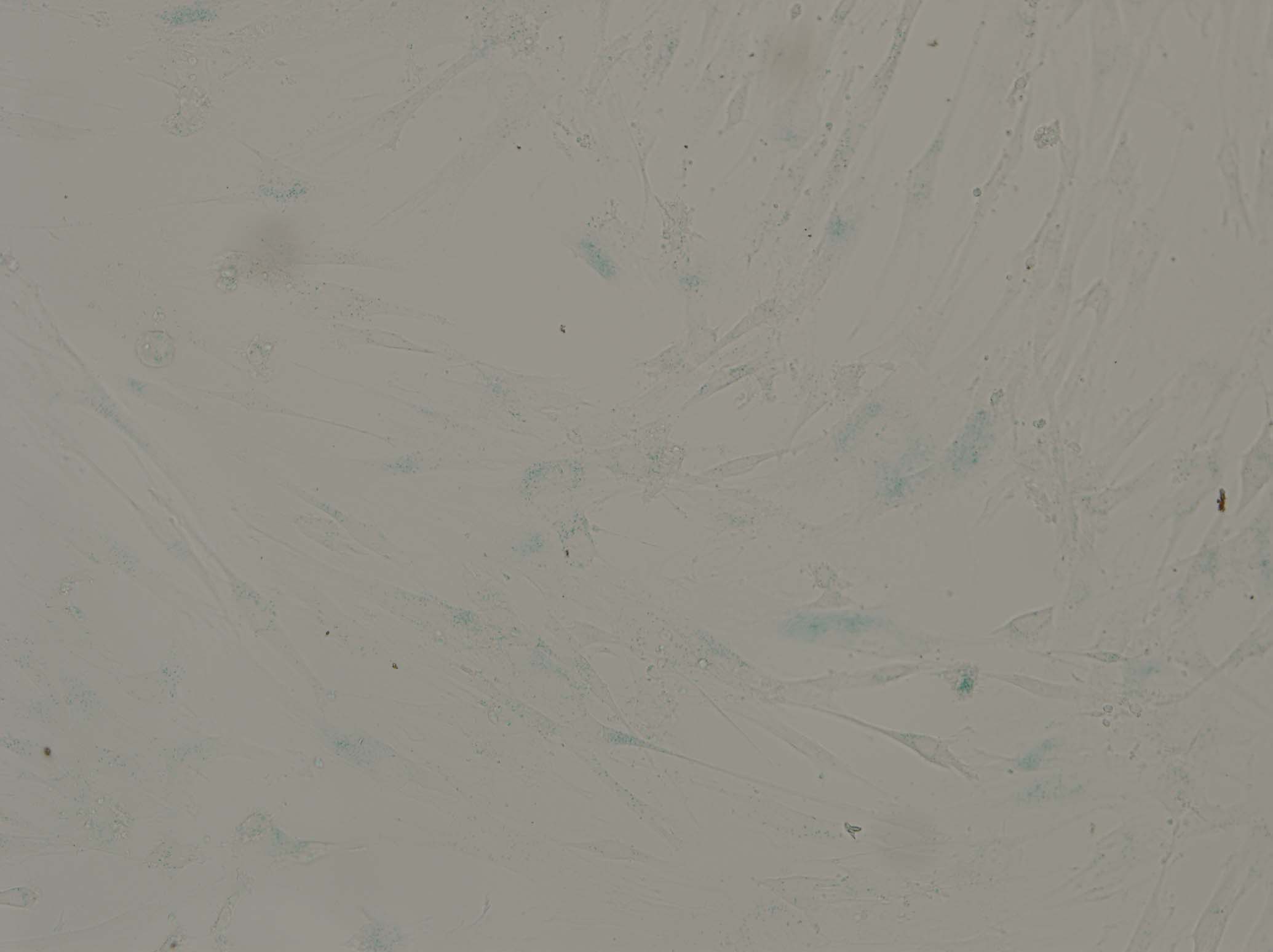

Supplement: Supplemental Information 4 — SA-β-Gal staining of human dental pulp cells with sclerostin overexpression and knockdown. [file peerj-06-5808-s004.zip › SA-B-Gal/SOST OVER/SOST-OVER/═╝╧±_12080.jpg]

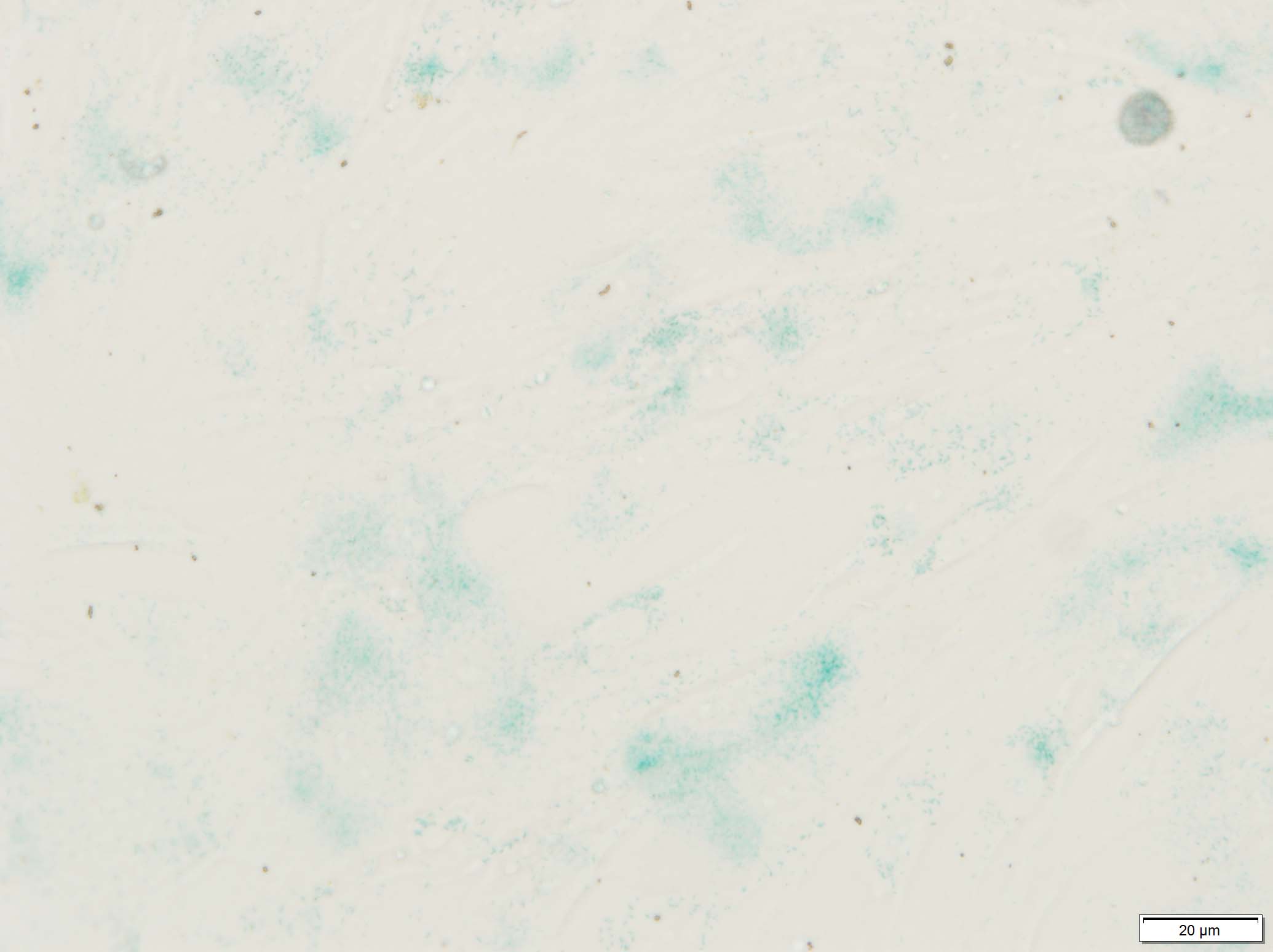

Supplement: Supplemental Information 4 — SA-β-Gal staining of human dental pulp cells with sclerostin overexpression and knockdown. [file peerj-06-5808-s004.zip › SA-B-Gal/SOST OVER/SOST-OVER/═╝╧±_13418.jpg]

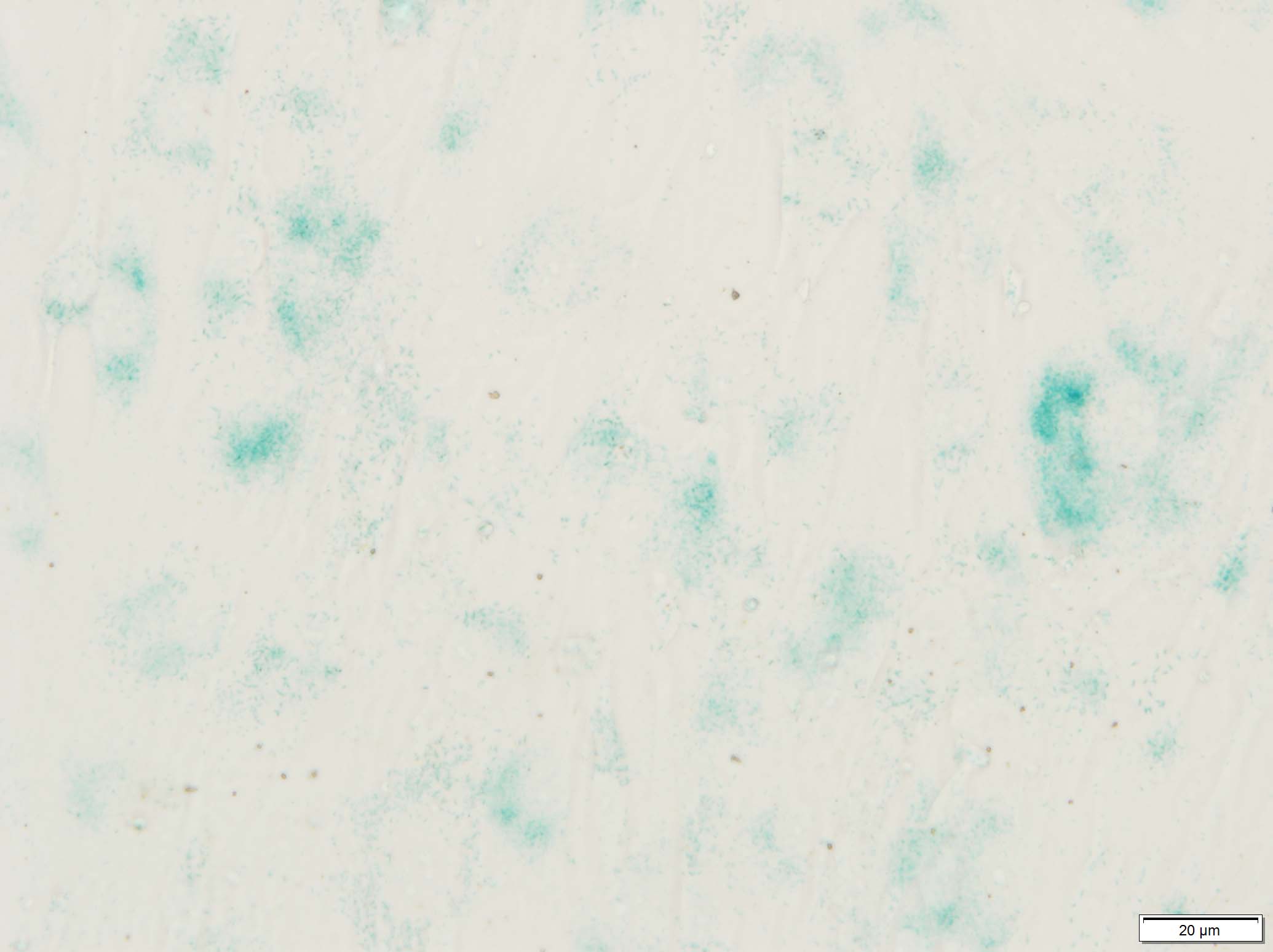

Supplement: Supplemental Information 4 — SA-β-Gal staining of human dental pulp cells with sclerostin overexpression and knockdown. [file peerj-06-5808-s004.zip › SA-B-Gal/SOST OVER/SOST-OVER/═╝╧±_13419.jpg]
