# Supplementary figures and images for: Sclerostin promotes human dental pulp cells senescence (part 2 of 2)
Source: PeerJ. 2018 Oct 17;6:e5808. doi: 10.7717/peerj.5808 (PMC6195797; doi:10.7717/peerj.5808)

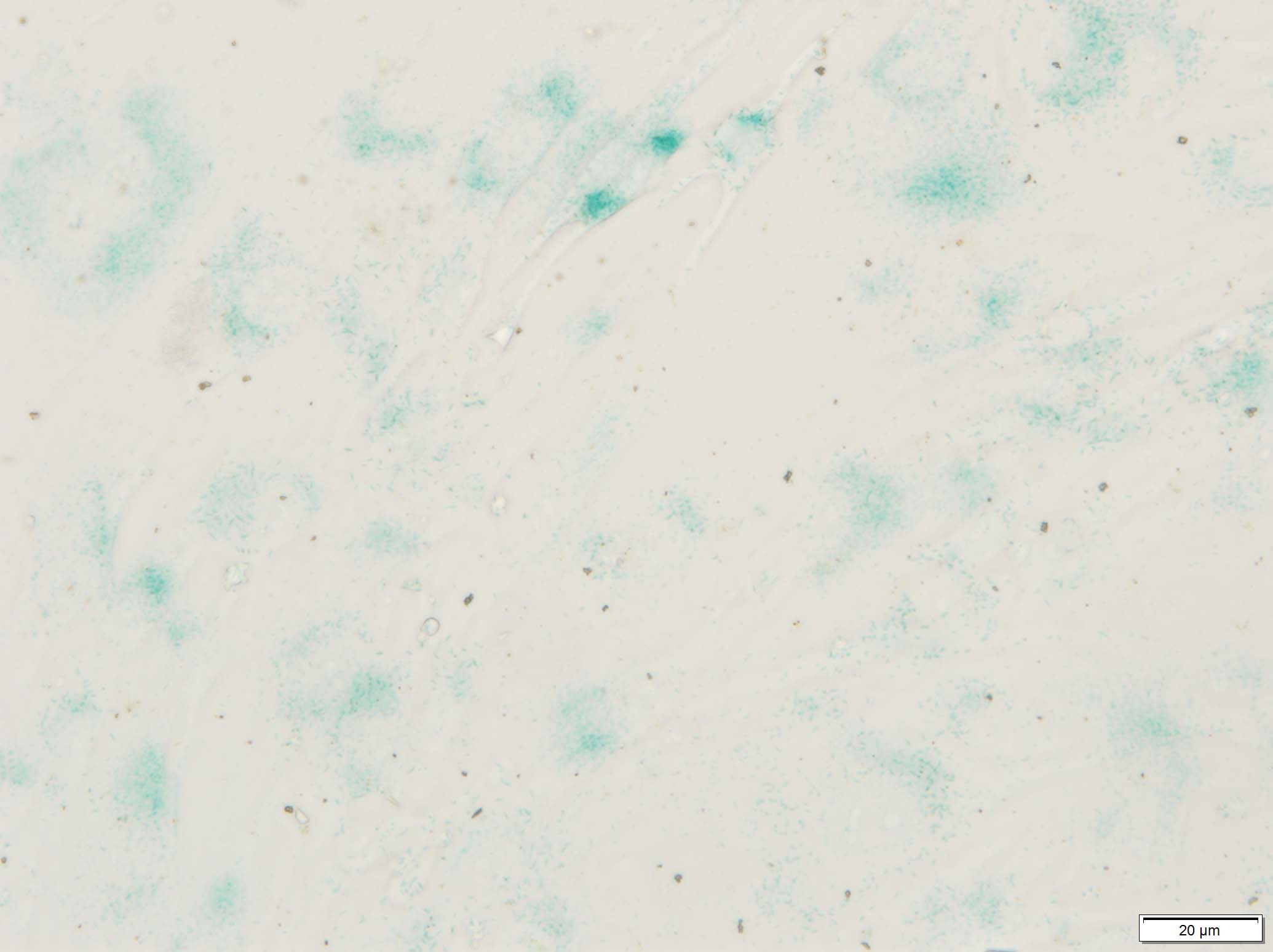

Supplement: Supplemental Information 4 — SA-β-Gal staining of human dental pulp cells with sclerostin overexpression and knockdown. [file peerj-06-5808-s004.zip › SA-B-Gal/SOST OVER/SOST-OVER/═╝╧±_13420.jpg]

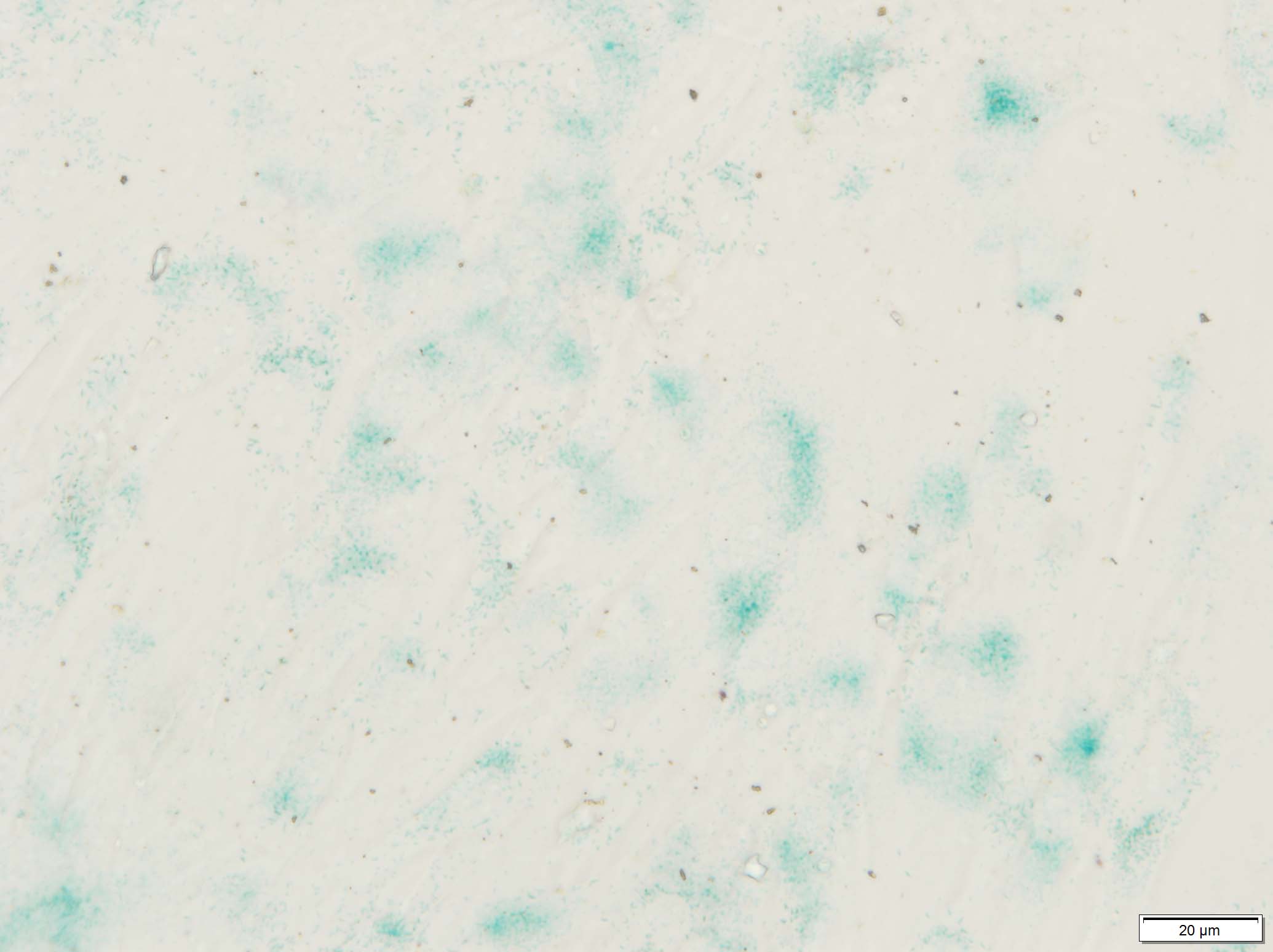

Supplement: Supplemental Information 4 — SA-β-Gal staining of human dental pulp cells with sclerostin overexpression and knockdown. [file peerj-06-5808-s004.zip › SA-B-Gal/SOST OVER/SOST-OVER/═╝╧±_13421.jpg]

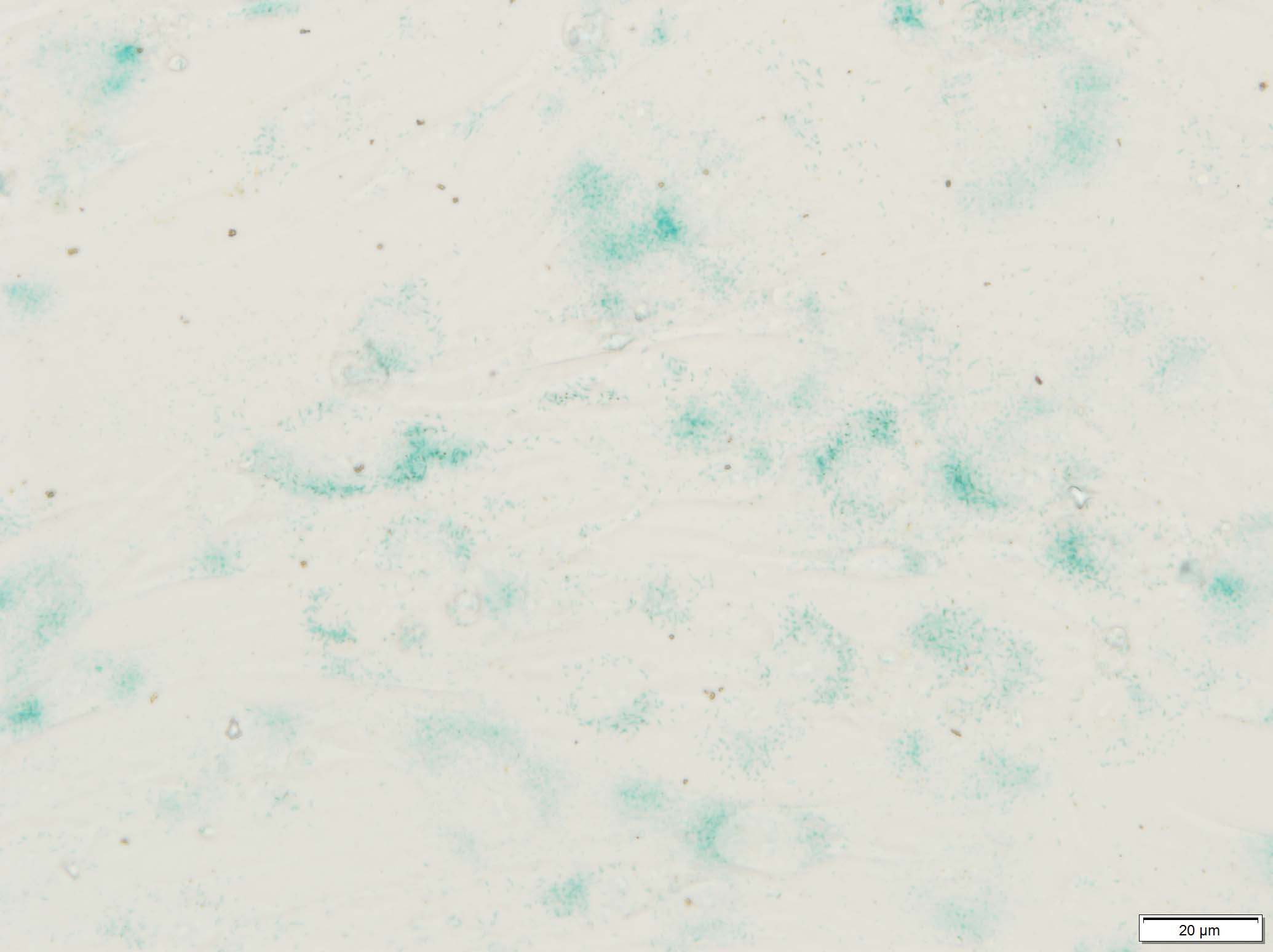

Supplement: Supplemental Information 4 — SA-β-Gal staining of human dental pulp cells with sclerostin overexpression and knockdown. [file peerj-06-5808-s004.zip › SA-B-Gal/SOST OVER/SOST-OVER/═╝╧±_13422.jpg]

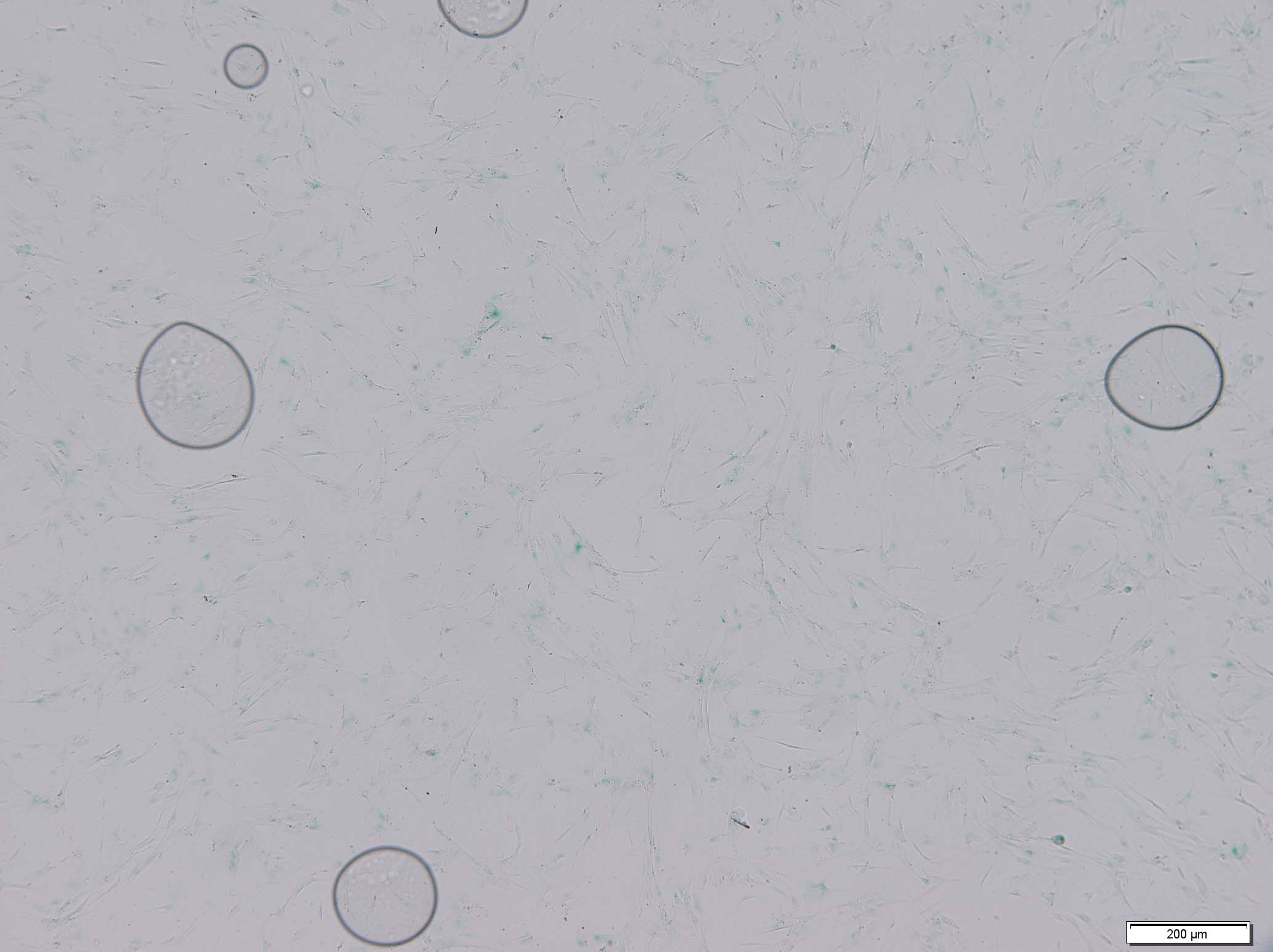

Supplement: Supplemental Information 4 — SA-β-Gal staining of human dental pulp cells with sclerostin overexpression and knockdown. [file peerj-06-5808-s004.zip › SA-B-Gal/sh-SOST/Ctrl/Image_4449.jpg]

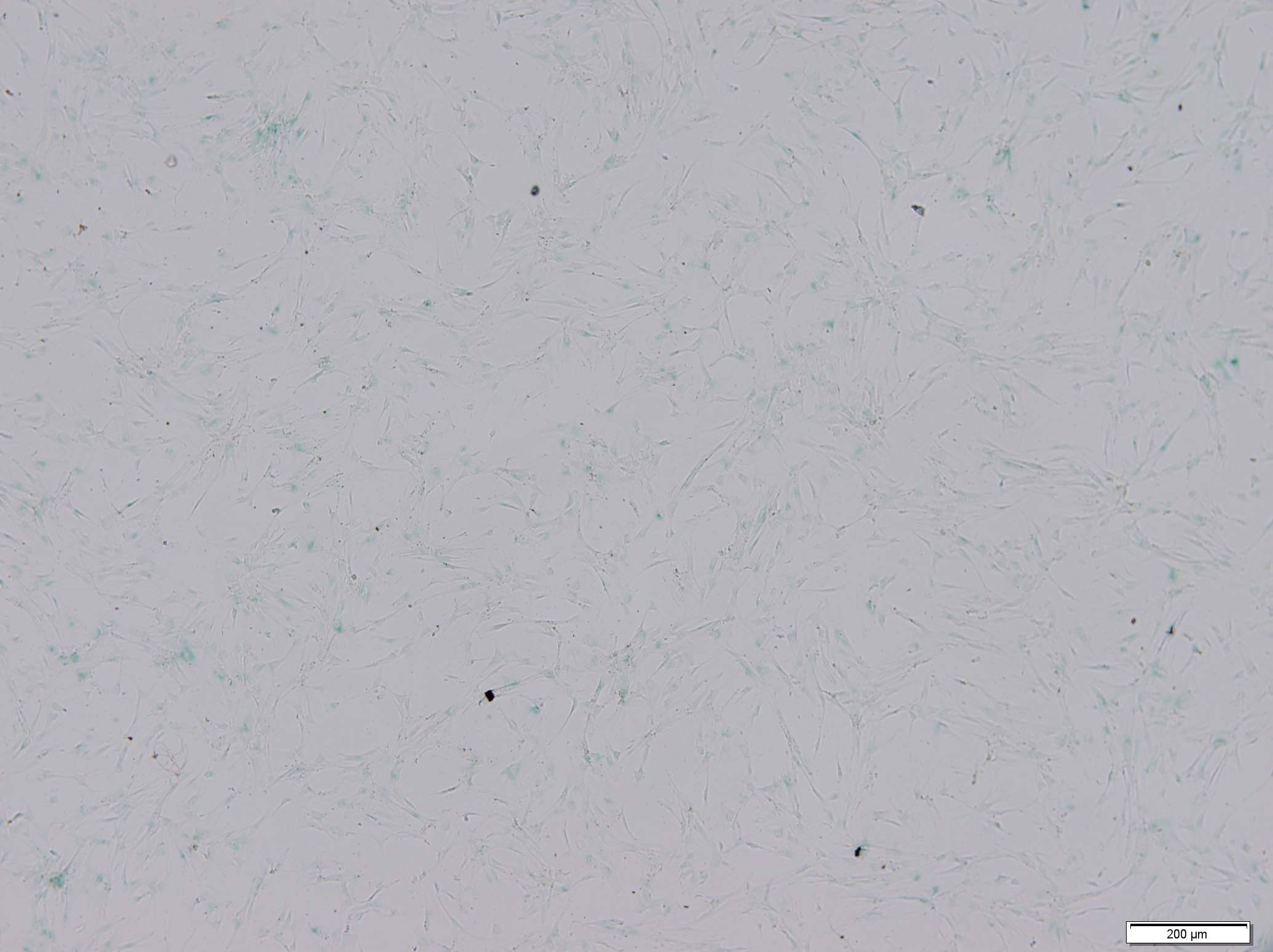

Supplement: Supplemental Information 4 — SA-β-Gal staining of human dental pulp cells with sclerostin overexpression and knockdown. [file peerj-06-5808-s004.zip › SA-B-Gal/sh-SOST/Ctrl/Image_4450.jpg]

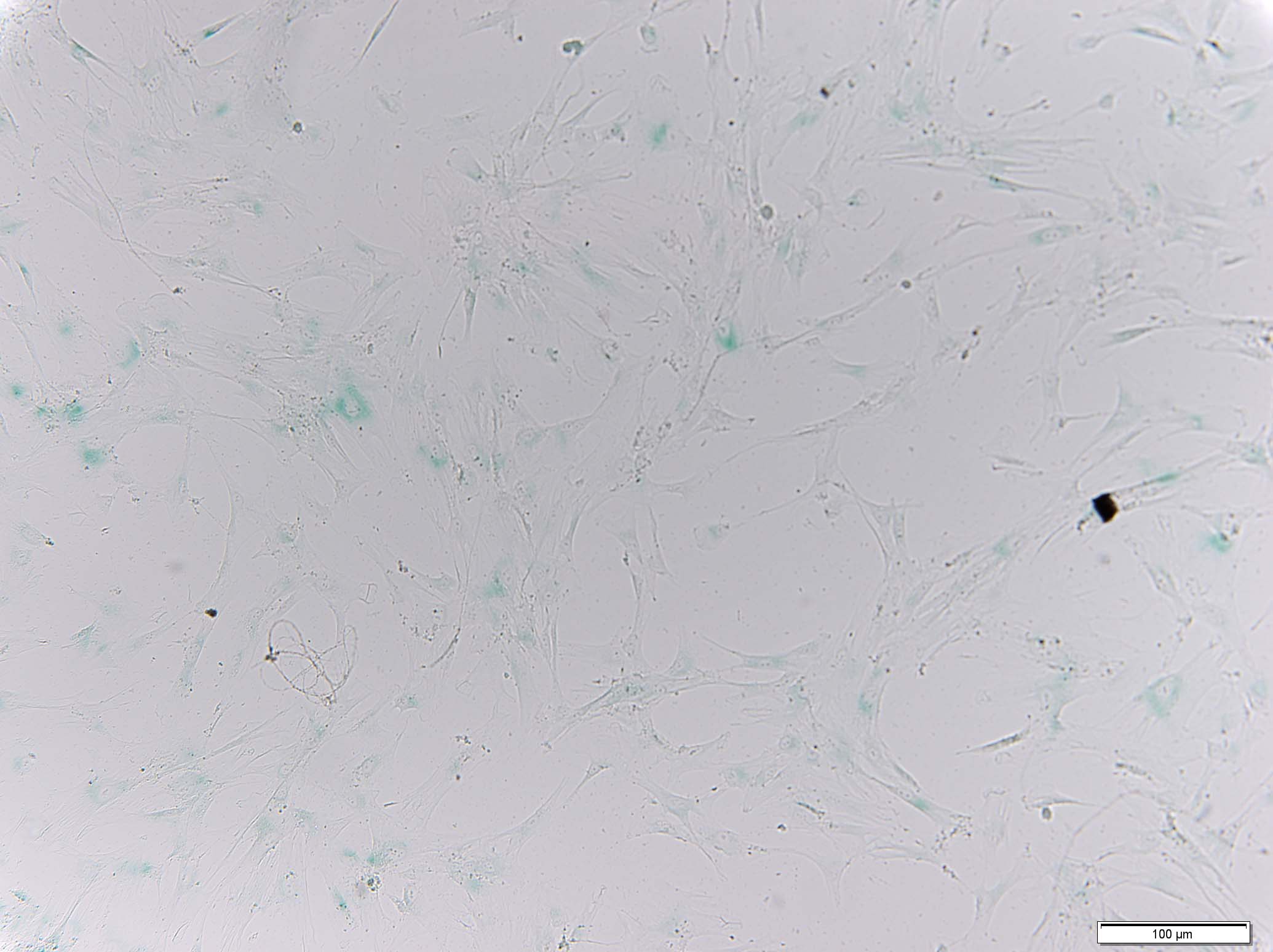

Supplement: Supplemental Information 4 — SA-β-Gal staining of human dental pulp cells with sclerostin overexpression and knockdown. [file peerj-06-5808-s004.zip › SA-B-Gal/sh-SOST/Ctrl/Image_4451.jpg]

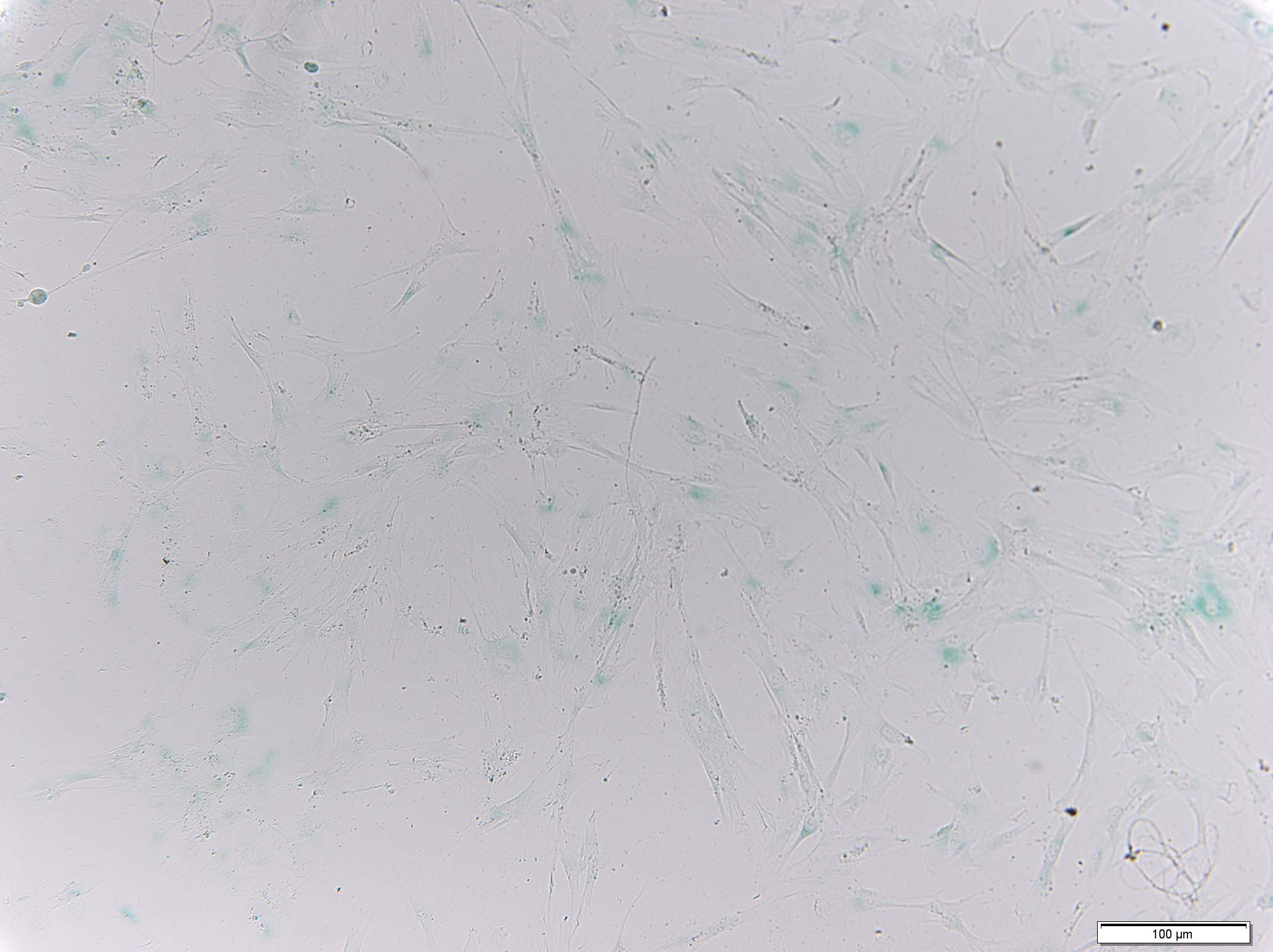

Supplement: Supplemental Information 4 — SA-β-Gal staining of human dental pulp cells with sclerostin overexpression and knockdown. [file peerj-06-5808-s004.zip › SA-B-Gal/sh-SOST/Ctrl/Image_4452.jpg]

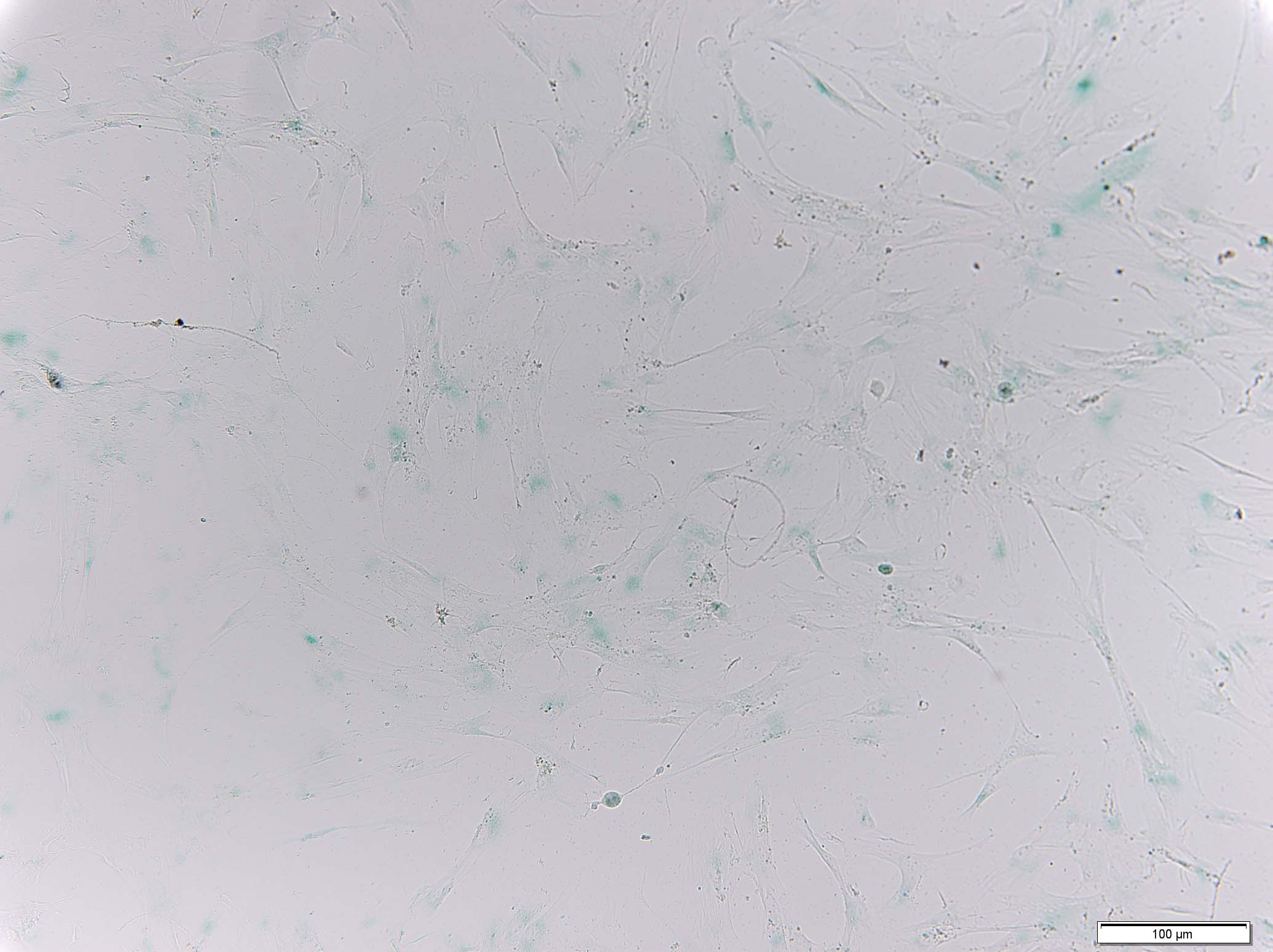

Supplement: Supplemental Information 4 — SA-β-Gal staining of human dental pulp cells with sclerostin overexpression and knockdown. [file peerj-06-5808-s004.zip › SA-B-Gal/sh-SOST/Ctrl/Image_4453.jpg]

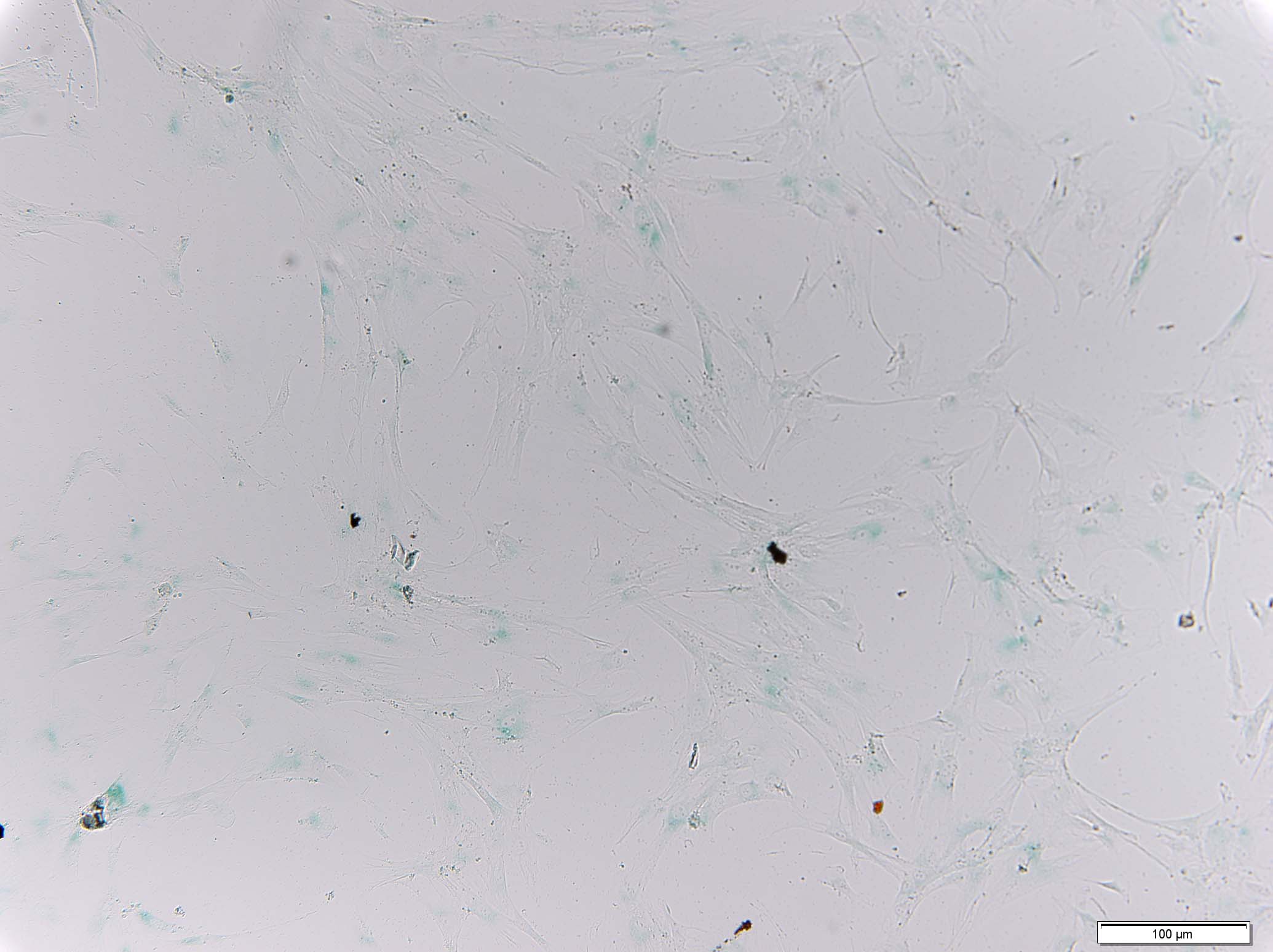

Supplement: Supplemental Information 4 — SA-β-Gal staining of human dental pulp cells with sclerostin overexpression and knockdown. [file peerj-06-5808-s004.zip › SA-B-Gal/sh-SOST/Ctrl/Image_4454.jpg]

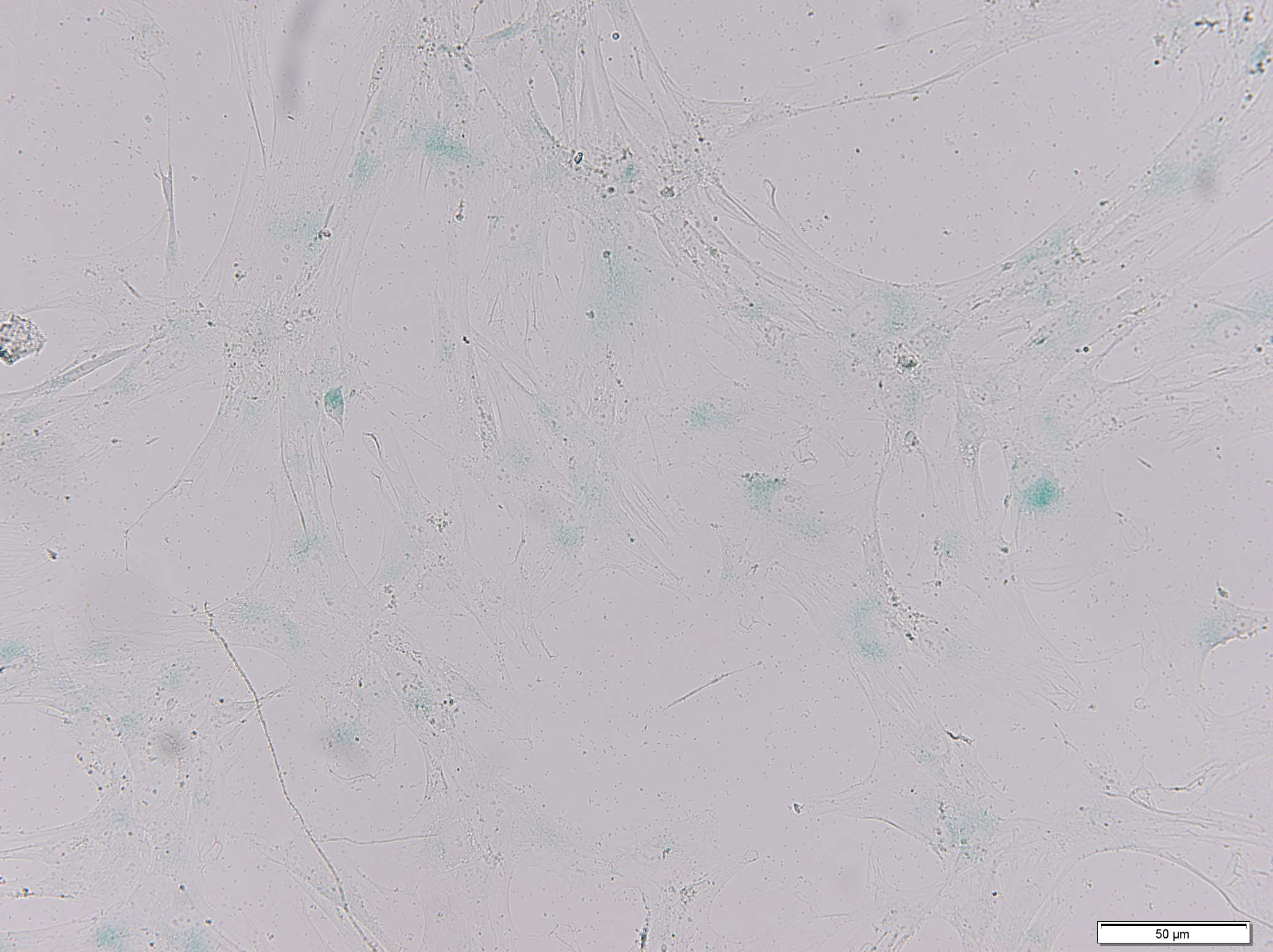

Supplement: Supplemental Information 4 — SA-β-Gal staining of human dental pulp cells with sclerostin overexpression and knockdown. [file peerj-06-5808-s004.zip › SA-B-Gal/sh-SOST/Ctrl/Image_4455.jpg]

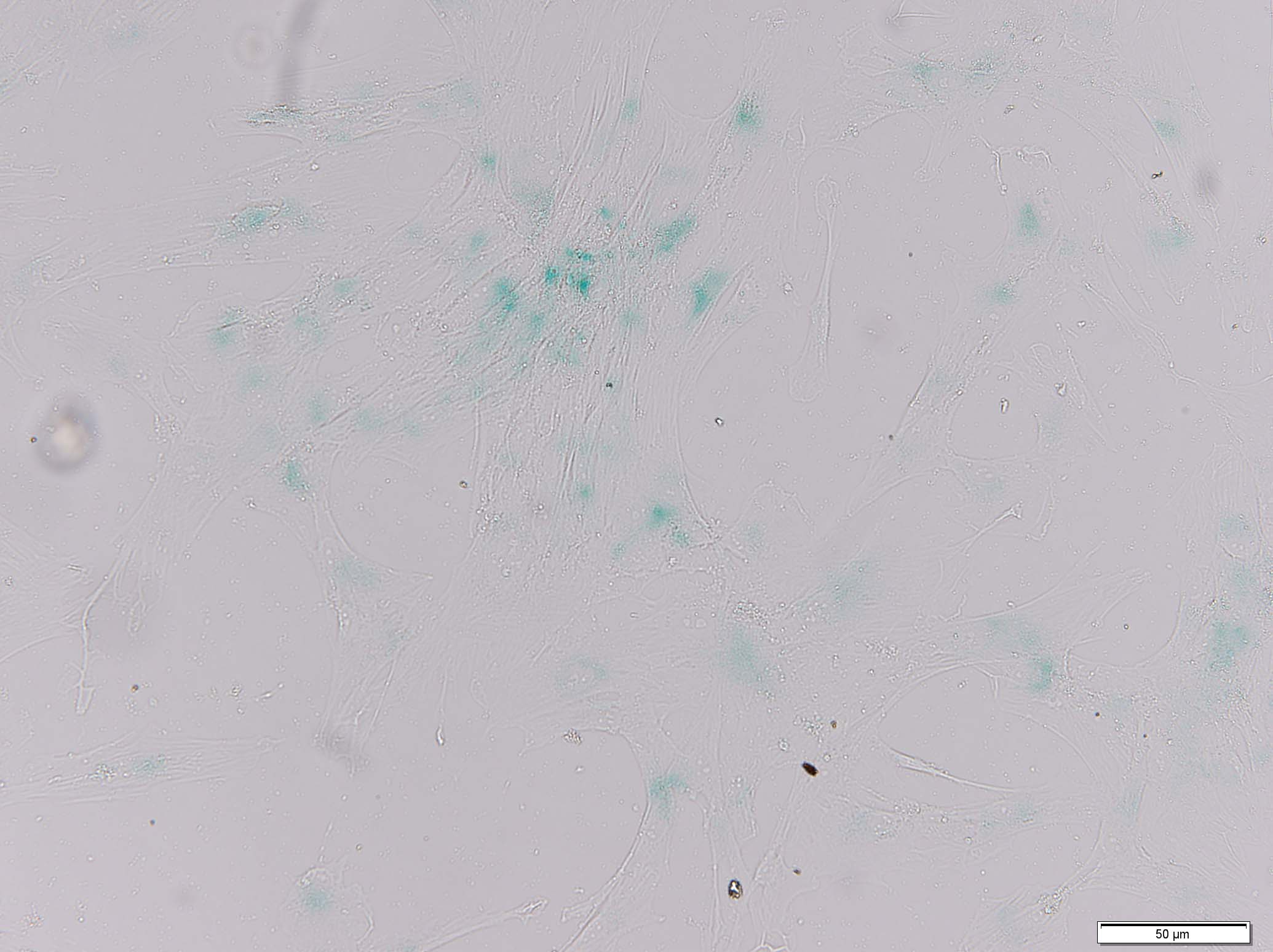

Supplement: Supplemental Information 4 — SA-β-Gal staining of human dental pulp cells with sclerostin overexpression and knockdown. [file peerj-06-5808-s004.zip › SA-B-Gal/sh-SOST/Ctrl/Image_4456.jpg]

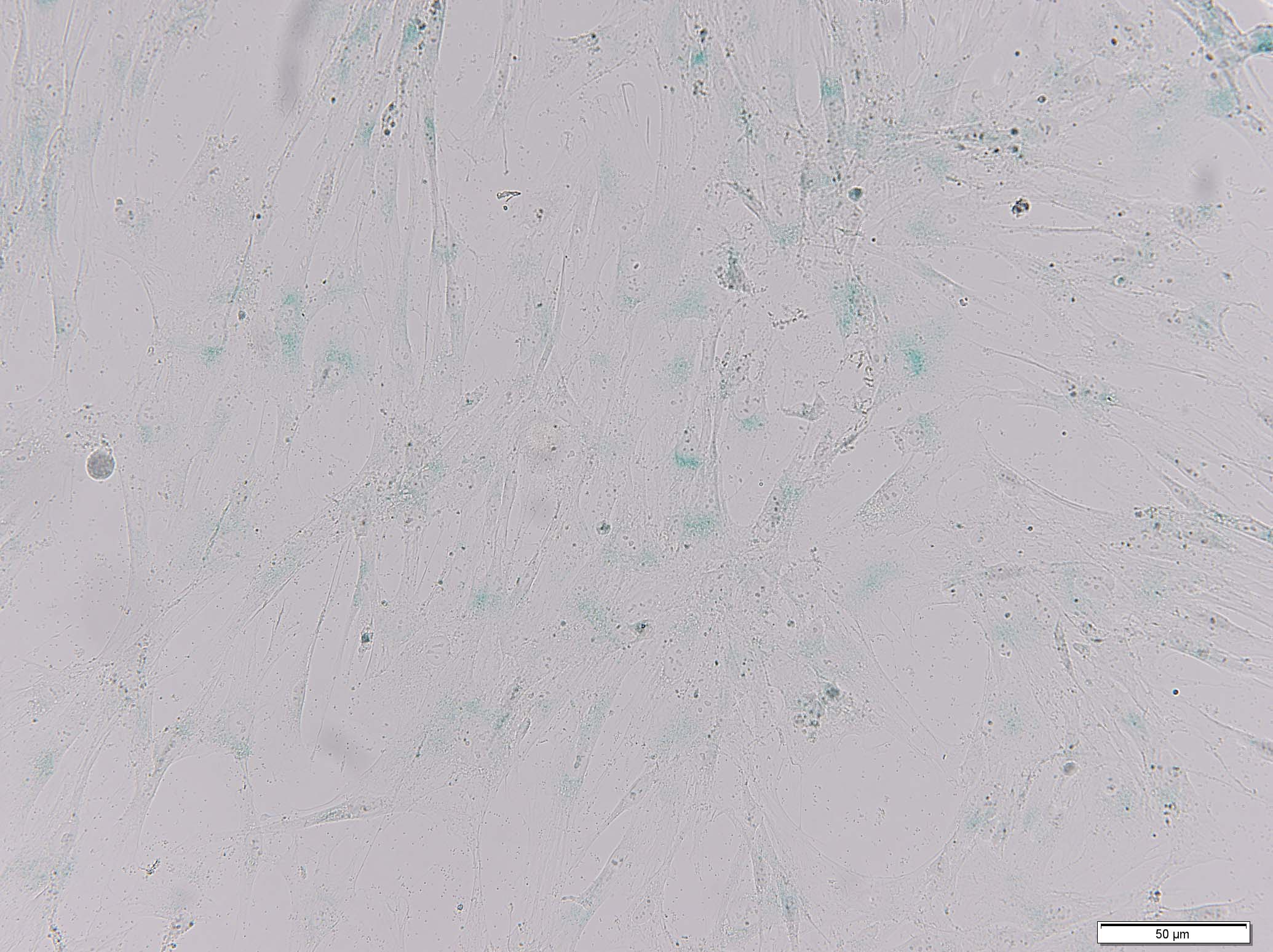

Supplement: Supplemental Information 4 — SA-β-Gal staining of human dental pulp cells with sclerostin overexpression and knockdown. [file peerj-06-5808-s004.zip › SA-B-Gal/sh-SOST/Ctrl/Image_4457.jpg]

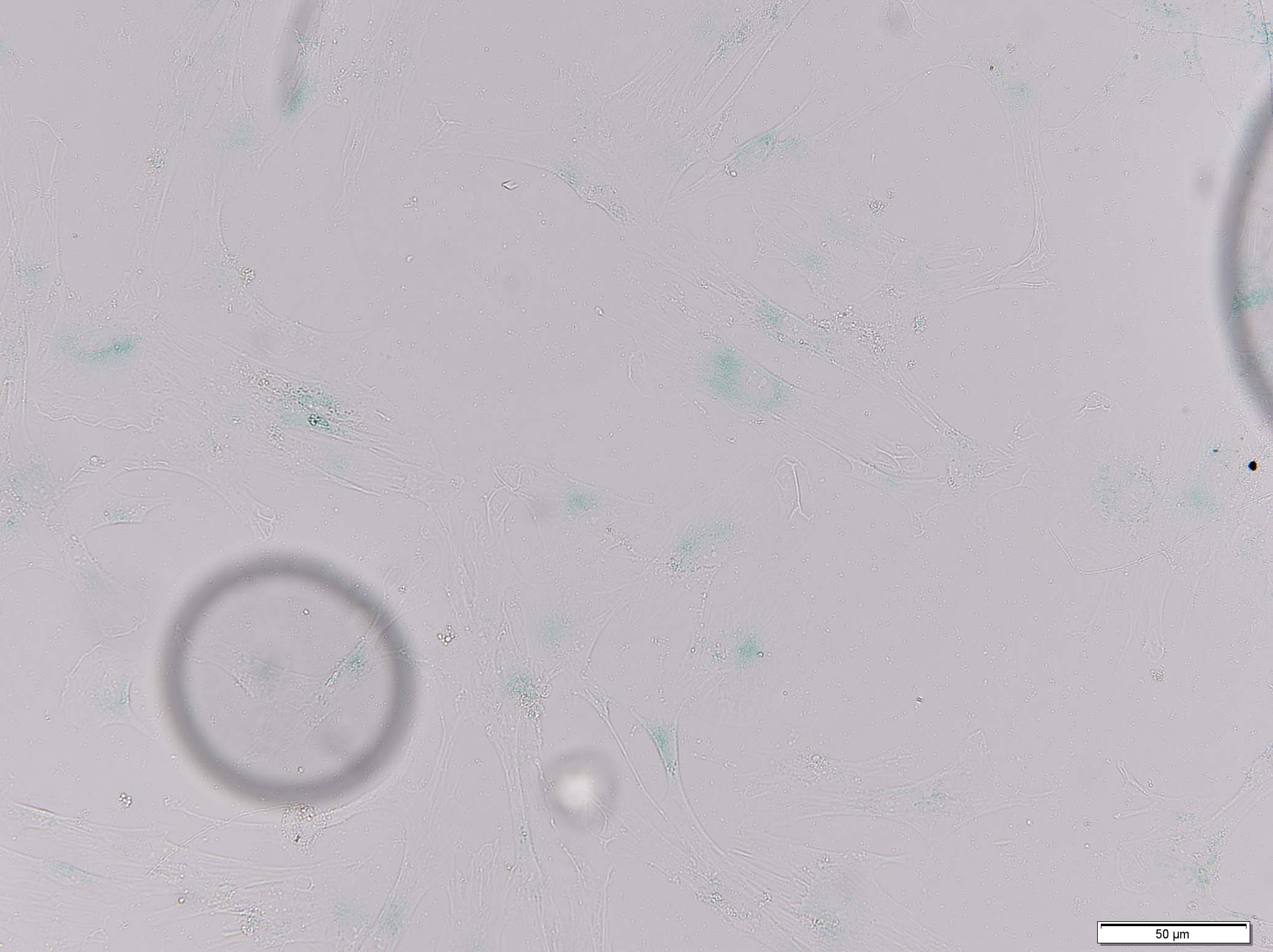

Supplement: Supplemental Information 4 — SA-β-Gal staining of human dental pulp cells with sclerostin overexpression and knockdown. [file peerj-06-5808-s004.zip › SA-B-Gal/sh-SOST/Ctrl/Image_4458.jpg]

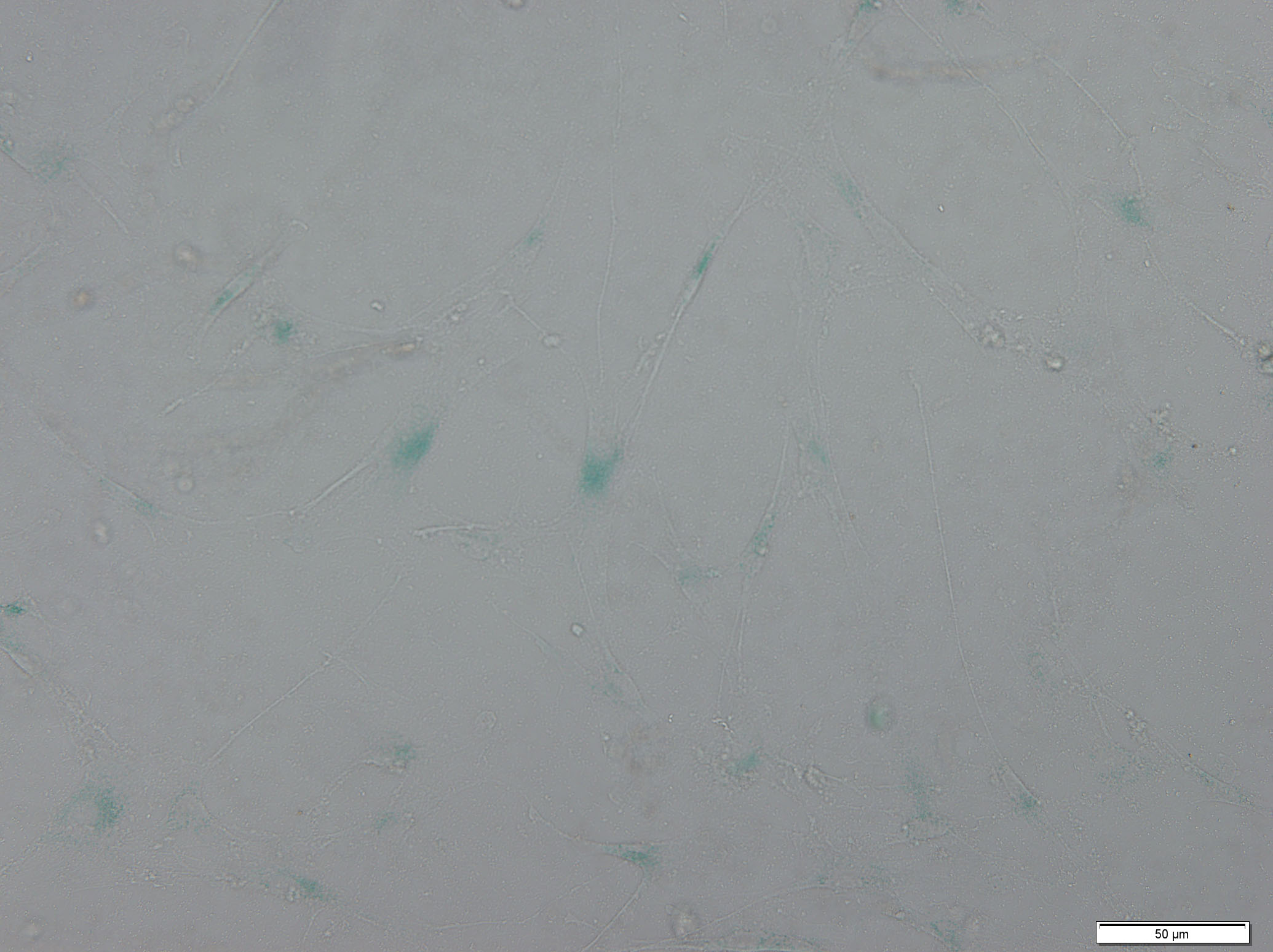

Supplement: Supplemental Information 4 — SA-β-Gal staining of human dental pulp cells with sclerostin overexpression and knockdown. [file peerj-06-5808-s004.zip › SA-B-Gal/sh-SOST/Ctrl/Image_9066.jpg]

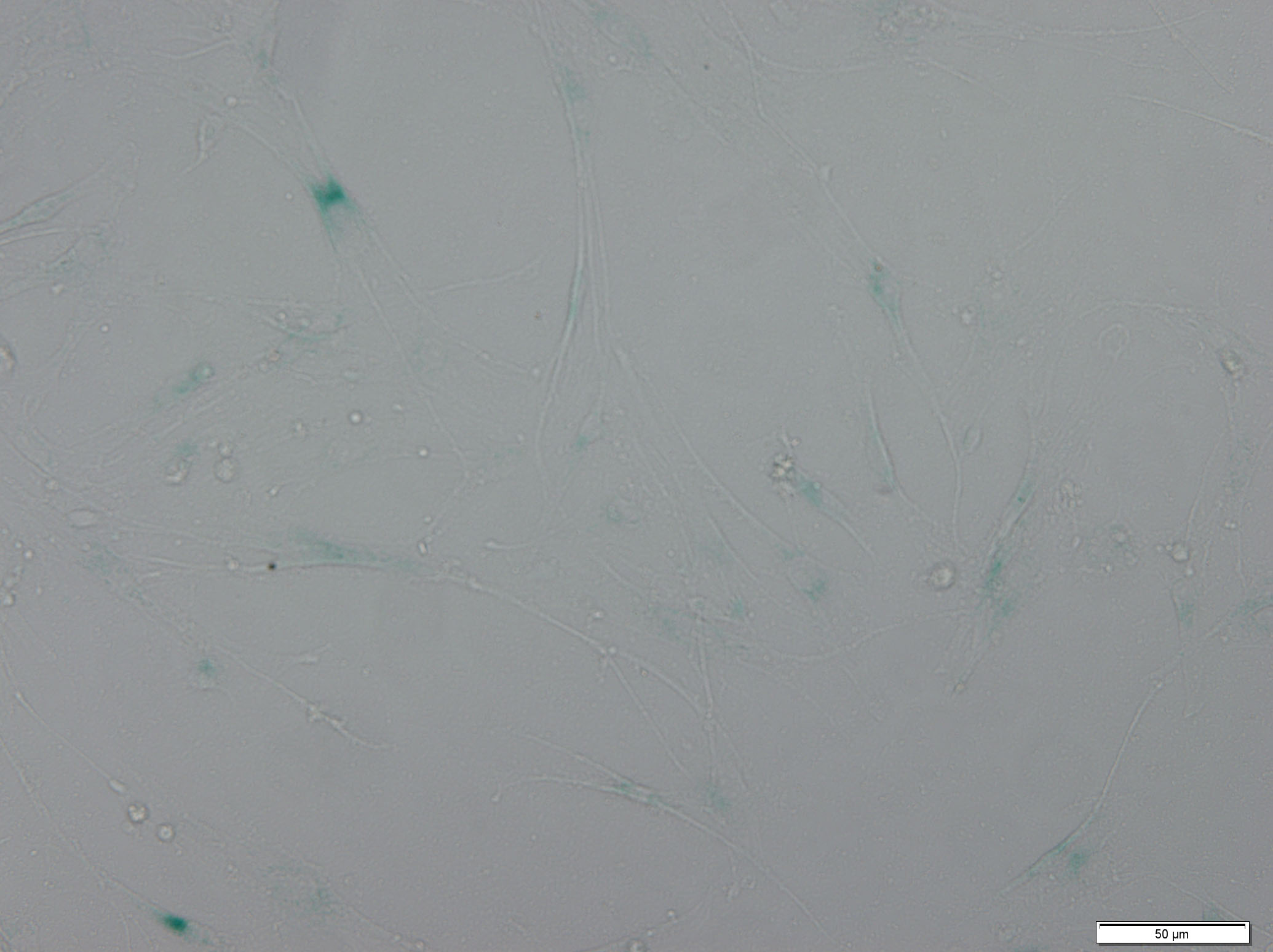

Supplement: Supplemental Information 4 — SA-β-Gal staining of human dental pulp cells with sclerostin overexpression and knockdown. [file peerj-06-5808-s004.zip › SA-B-Gal/sh-SOST/Ctrl/Image_9067.jpg]

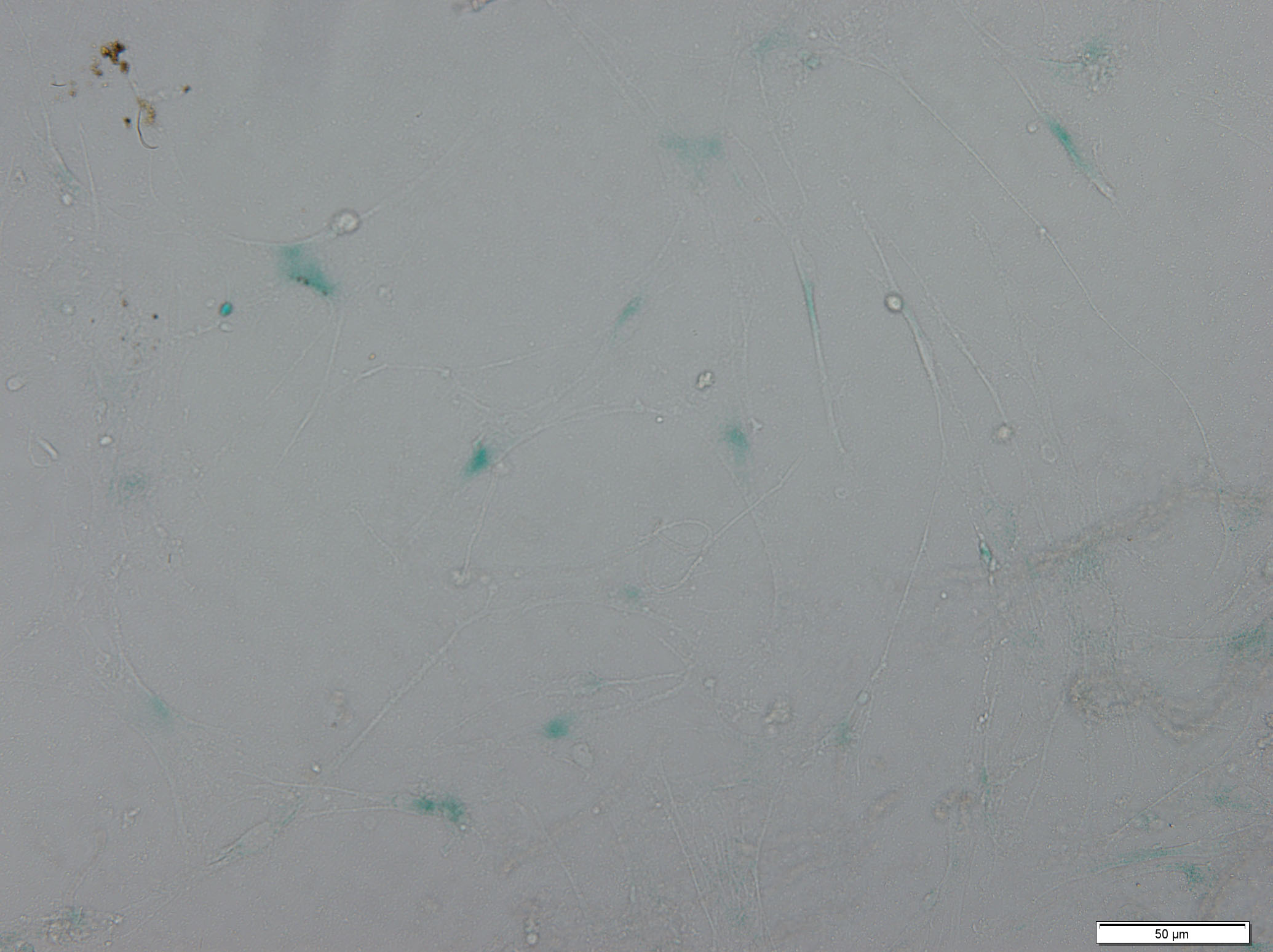

Supplement: Supplemental Information 4 — SA-β-Gal staining of human dental pulp cells with sclerostin overexpression and knockdown. [file peerj-06-5808-s004.zip › SA-B-Gal/sh-SOST/Ctrl/Image_9068.jpg]

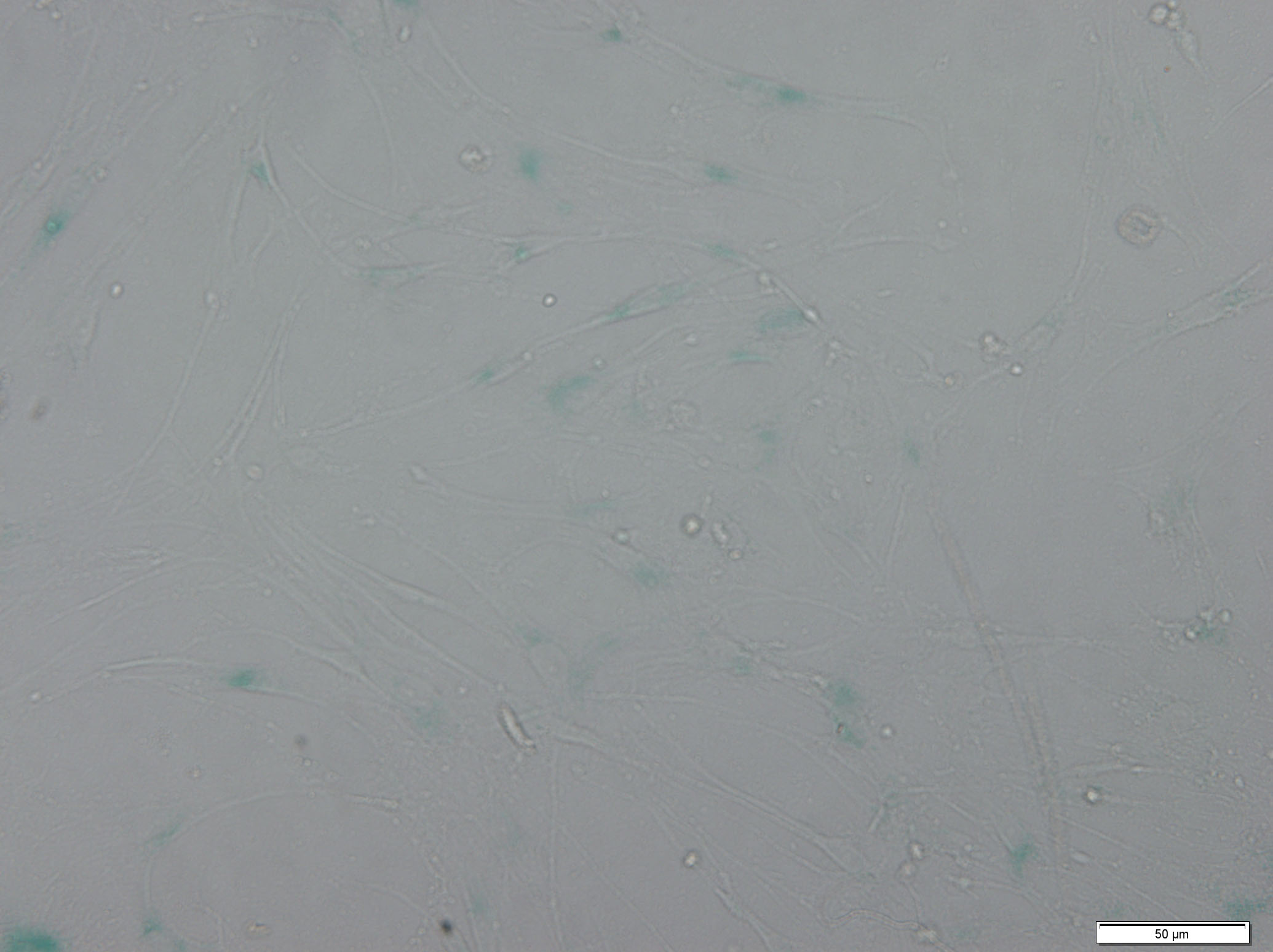

Supplement: Supplemental Information 4 — SA-β-Gal staining of human dental pulp cells with sclerostin overexpression and knockdown. [file peerj-06-5808-s004.zip › SA-B-Gal/sh-SOST/Ctrl/Image_9069.jpg]

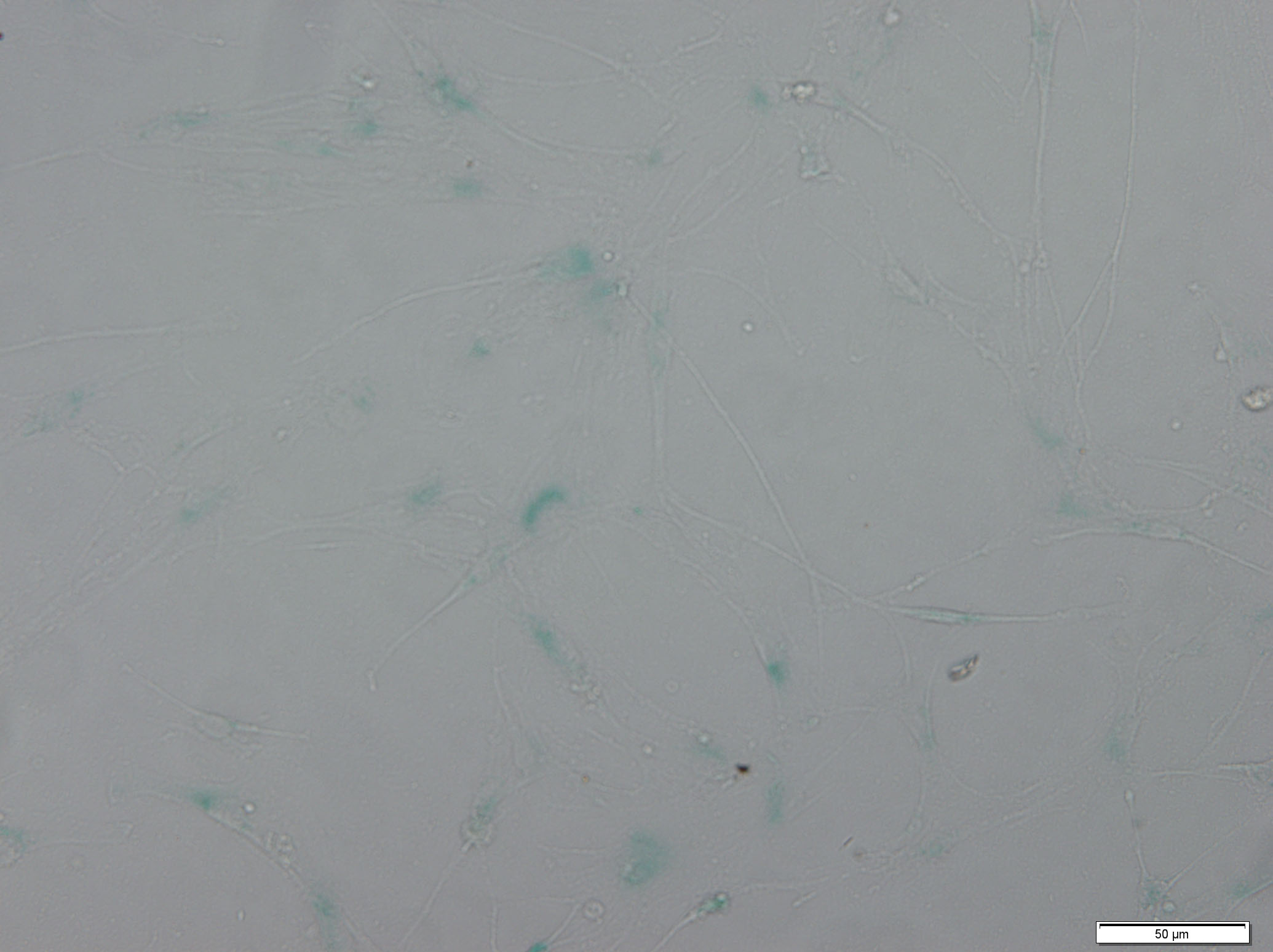

Supplement: Supplemental Information 4 — SA-β-Gal staining of human dental pulp cells with sclerostin overexpression and knockdown. [file peerj-06-5808-s004.zip › SA-B-Gal/sh-SOST/Ctrl/Image_9070.jpg]

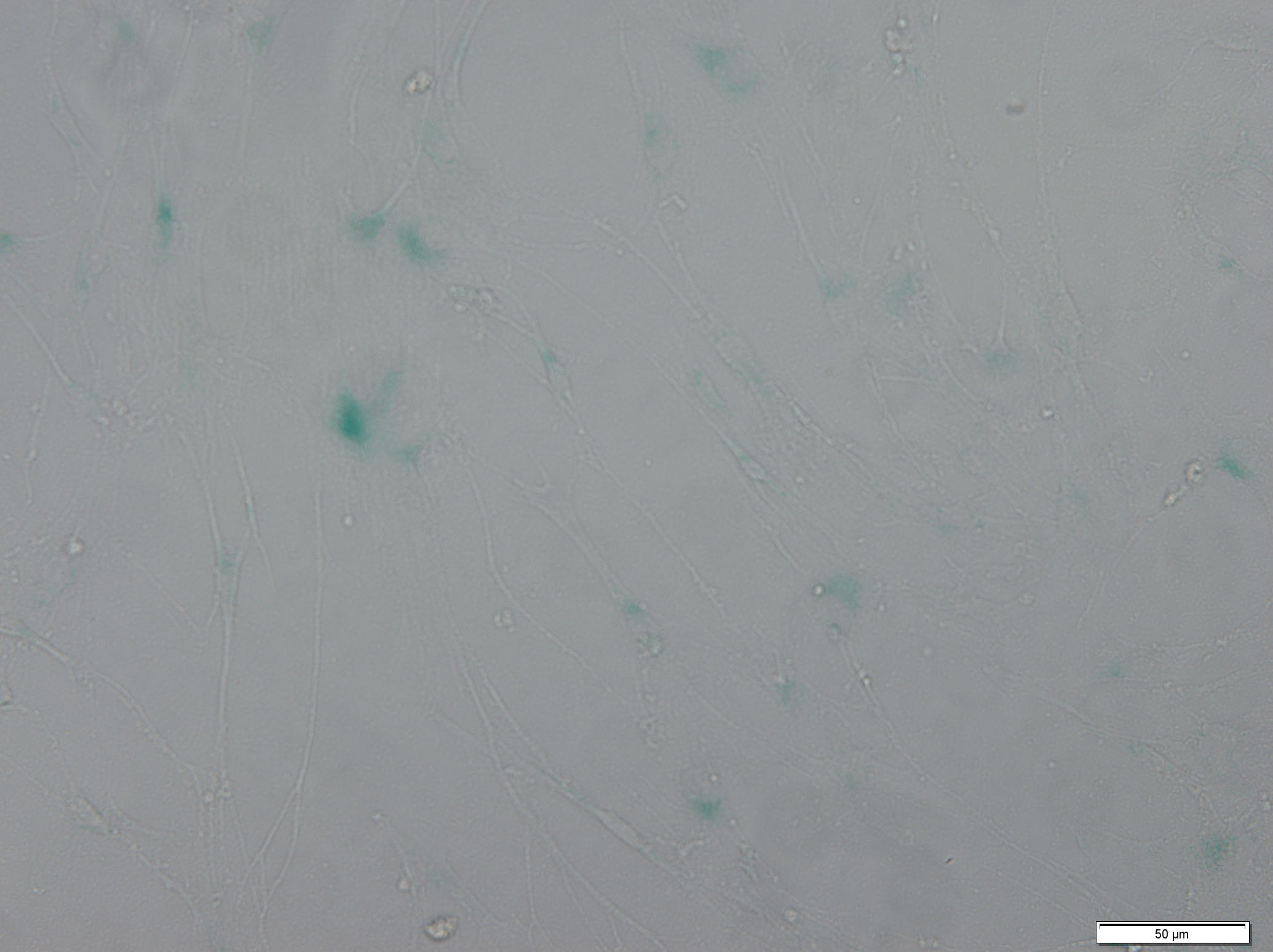

Supplement: Supplemental Information 4 — SA-β-Gal staining of human dental pulp cells with sclerostin overexpression and knockdown. [file peerj-06-5808-s004.zip › SA-B-Gal/sh-SOST/Ctrl/Image_9071.jpg]

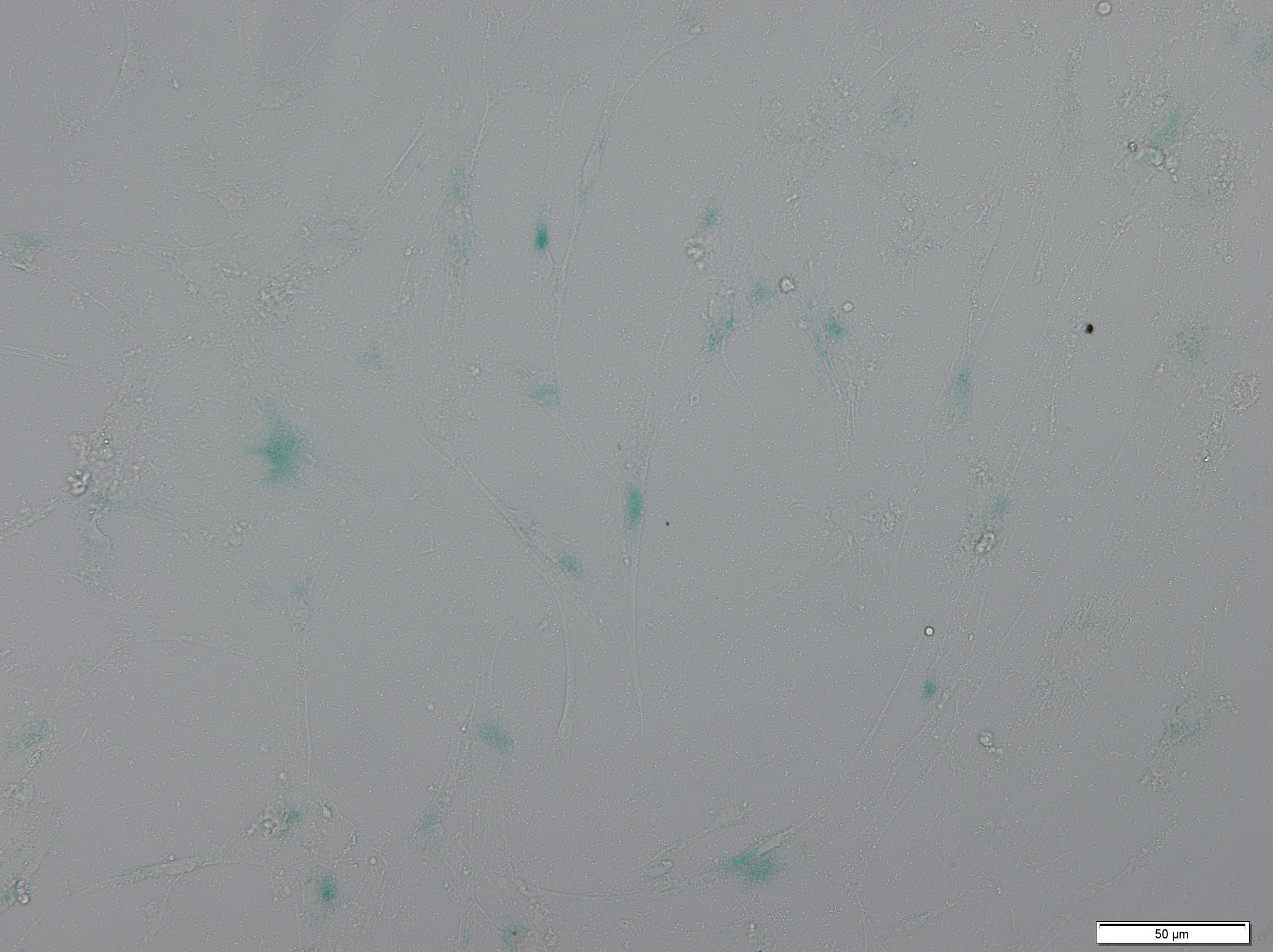

Supplement: Supplemental Information 4 — SA-β-Gal staining of human dental pulp cells with sclerostin overexpression and knockdown. [file peerj-06-5808-s004.zip › SA-B-Gal/sh-SOST/Ctrl/Image_9072.jpg]

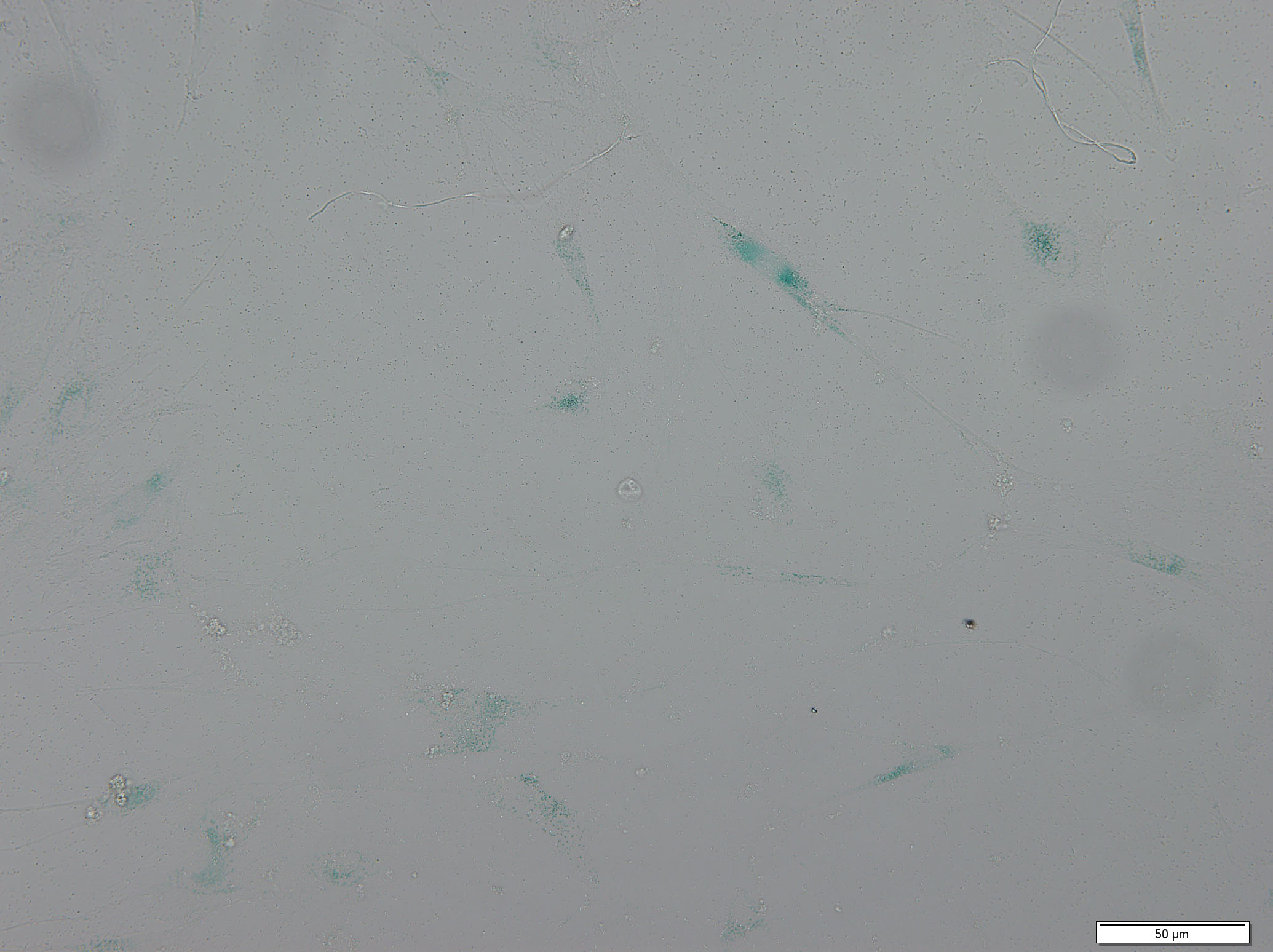

Supplement: Supplemental Information 4 — SA-β-Gal staining of human dental pulp cells with sclerostin overexpression and knockdown. [file peerj-06-5808-s004.zip › SA-B-Gal/sh-SOST/Ctrl/Image_9073.jpg]

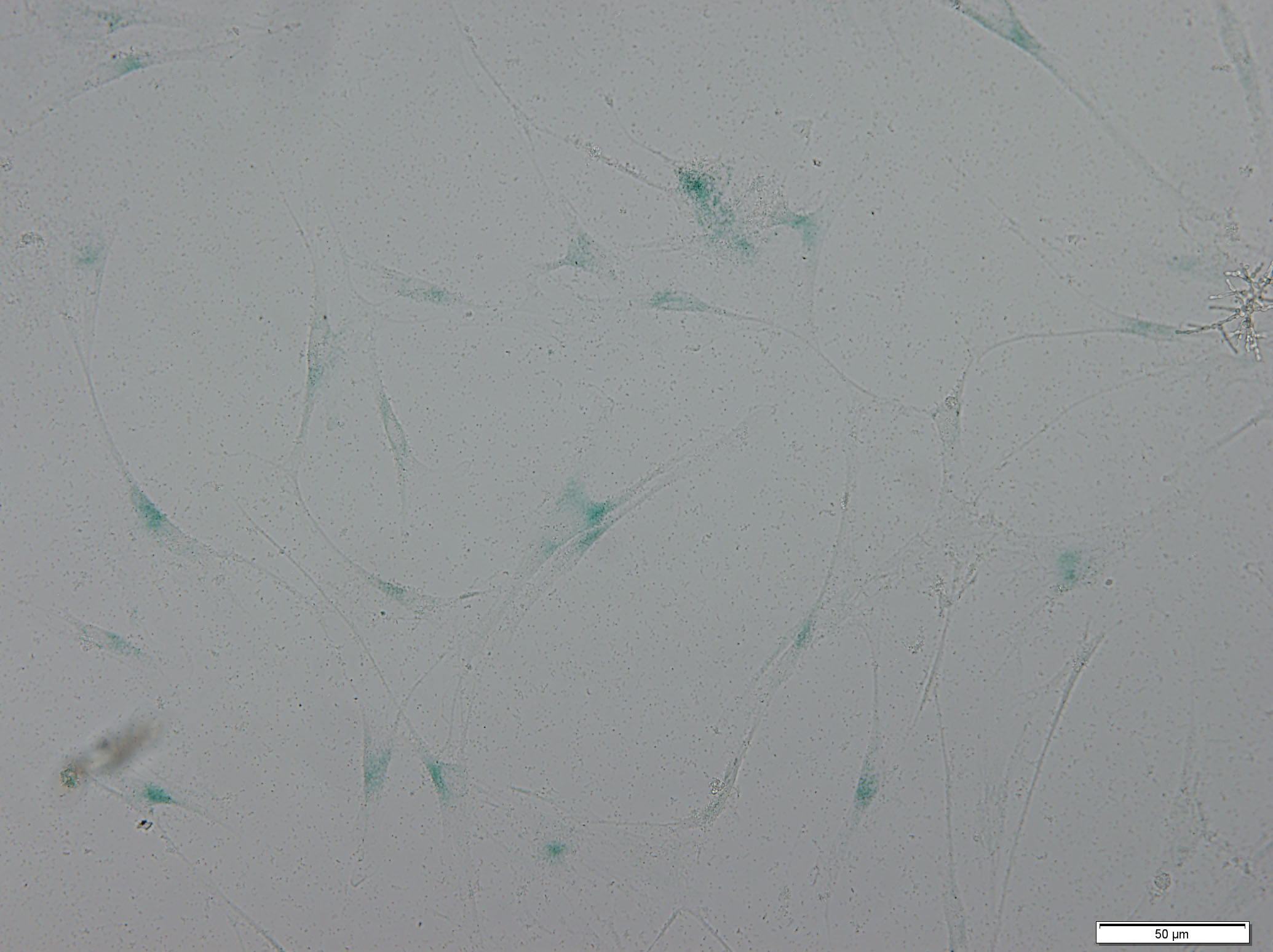

Supplement: Supplemental Information 4 — SA-β-Gal staining of human dental pulp cells with sclerostin overexpression and knockdown. [file peerj-06-5808-s004.zip › SA-B-Gal/sh-SOST/Ctrl/Image_9074.jpg]

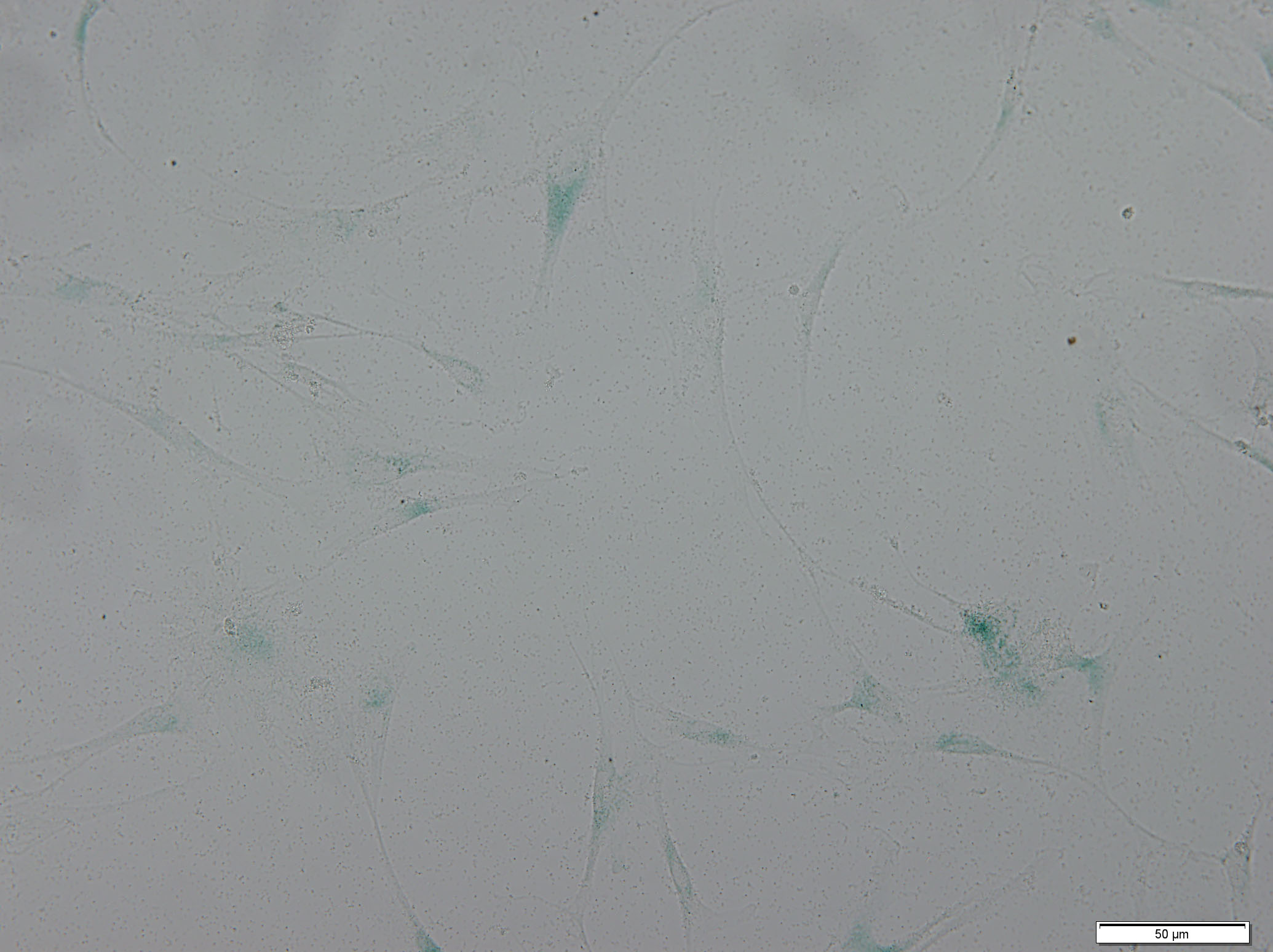

Supplement: Supplemental Information 4 — SA-β-Gal staining of human dental pulp cells with sclerostin overexpression and knockdown. [file peerj-06-5808-s004.zip › SA-B-Gal/sh-SOST/Ctrl/Image_9075.jpg]

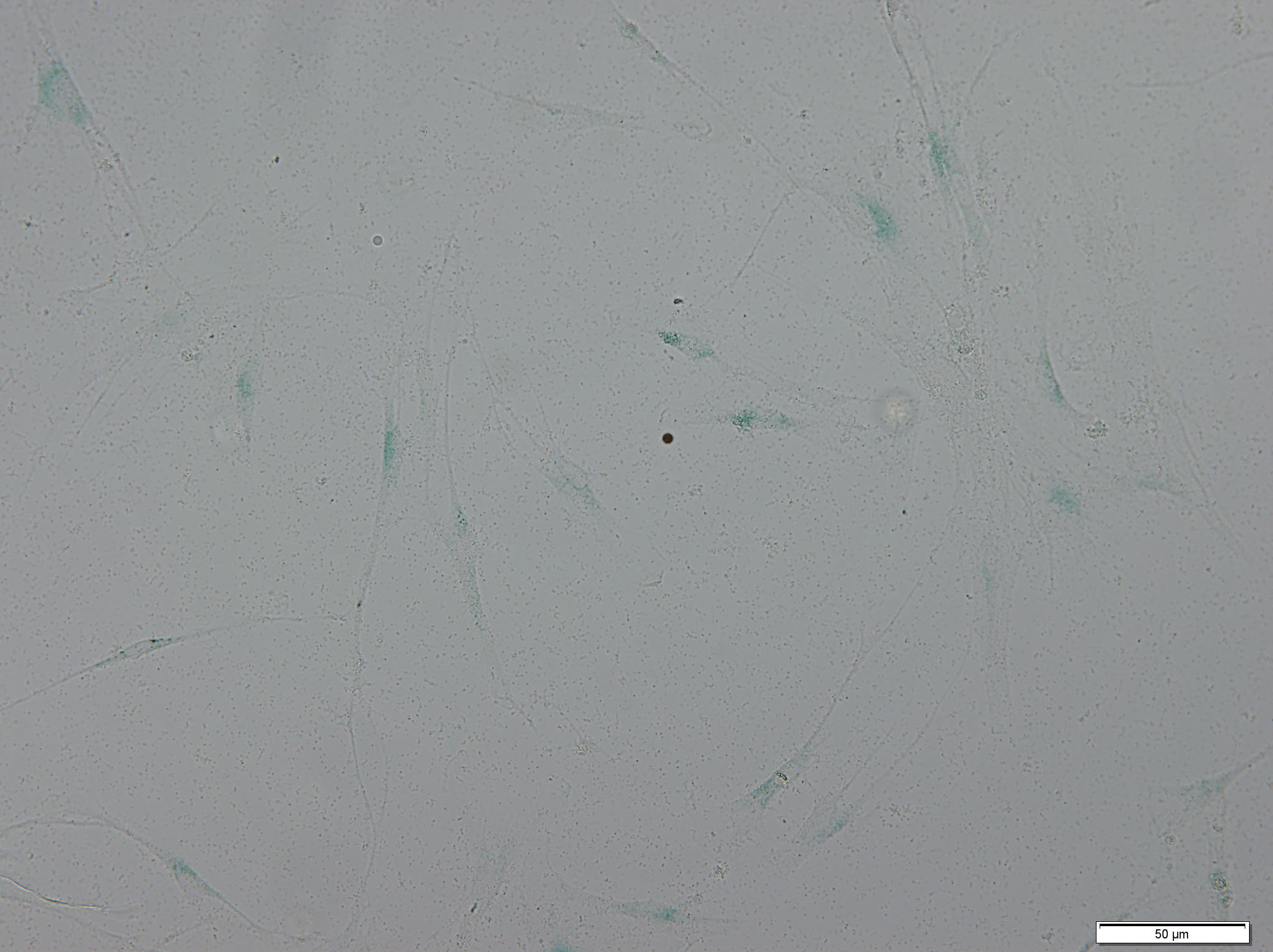

Supplement: Supplemental Information 4 — SA-β-Gal staining of human dental pulp cells with sclerostin overexpression and knockdown. [file peerj-06-5808-s004.zip › SA-B-Gal/sh-SOST/Ctrl/Image_9076.jpg]

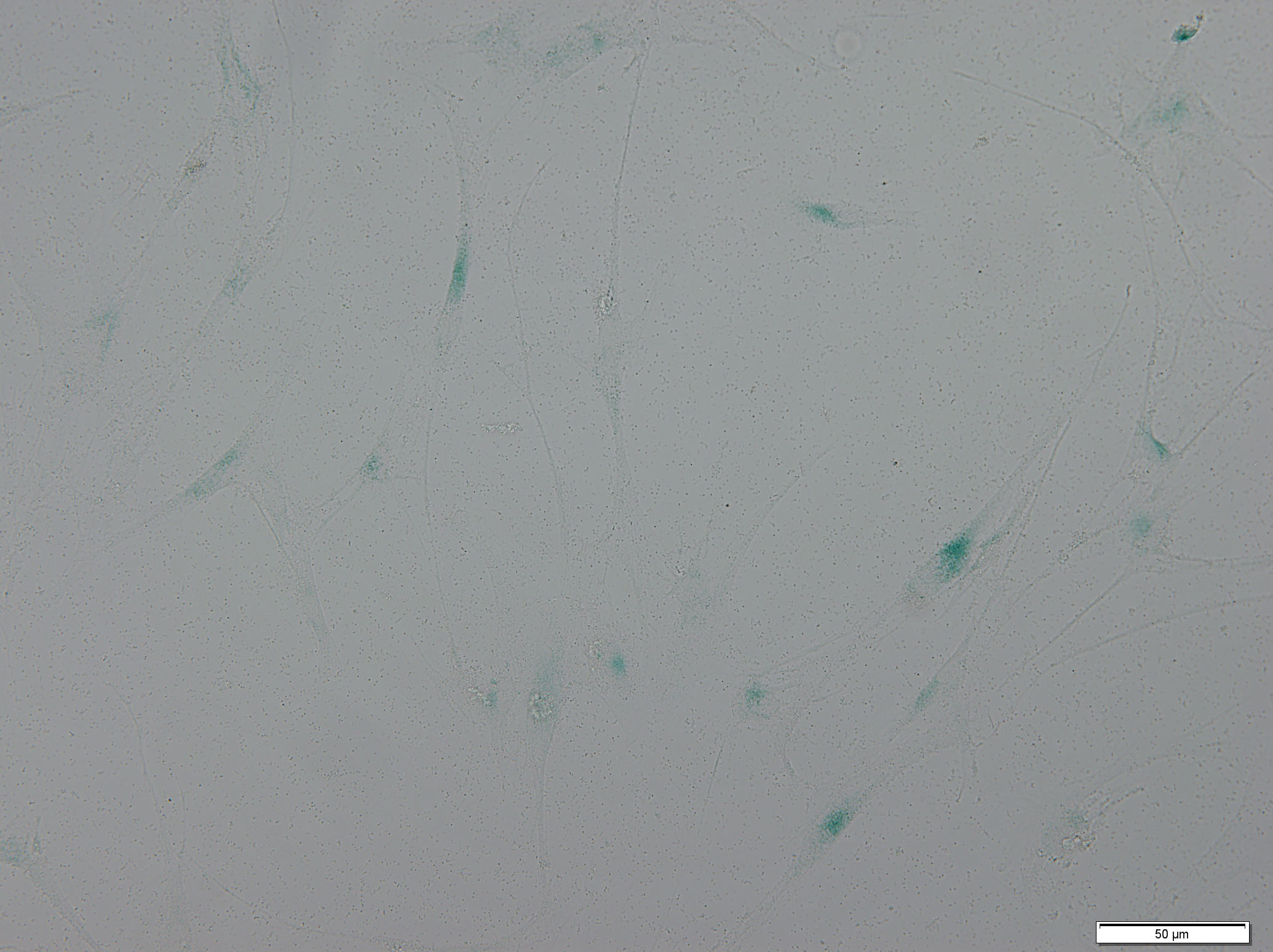

Supplement: Supplemental Information 4 — SA-β-Gal staining of human dental pulp cells with sclerostin overexpression and knockdown. [file peerj-06-5808-s004.zip › SA-B-Gal/sh-SOST/Ctrl/Image_9077.jpg]

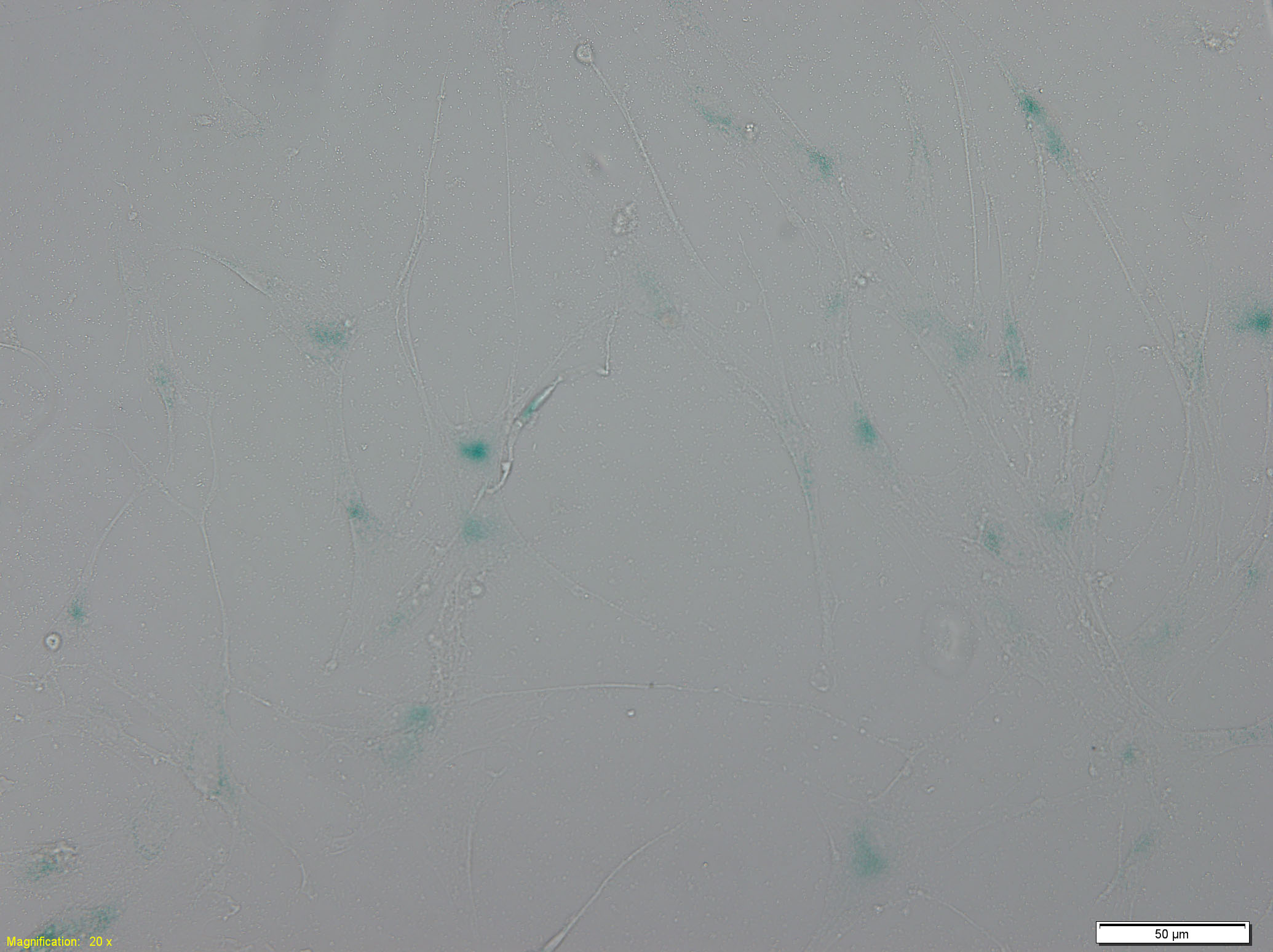

Supplement: Supplemental Information 4 — SA-β-Gal staining of human dental pulp cells with sclerostin overexpression and knockdown. [file peerj-06-5808-s004.zip › SA-B-Gal/sh-SOST/Ctrl/Image_9078.jpg]

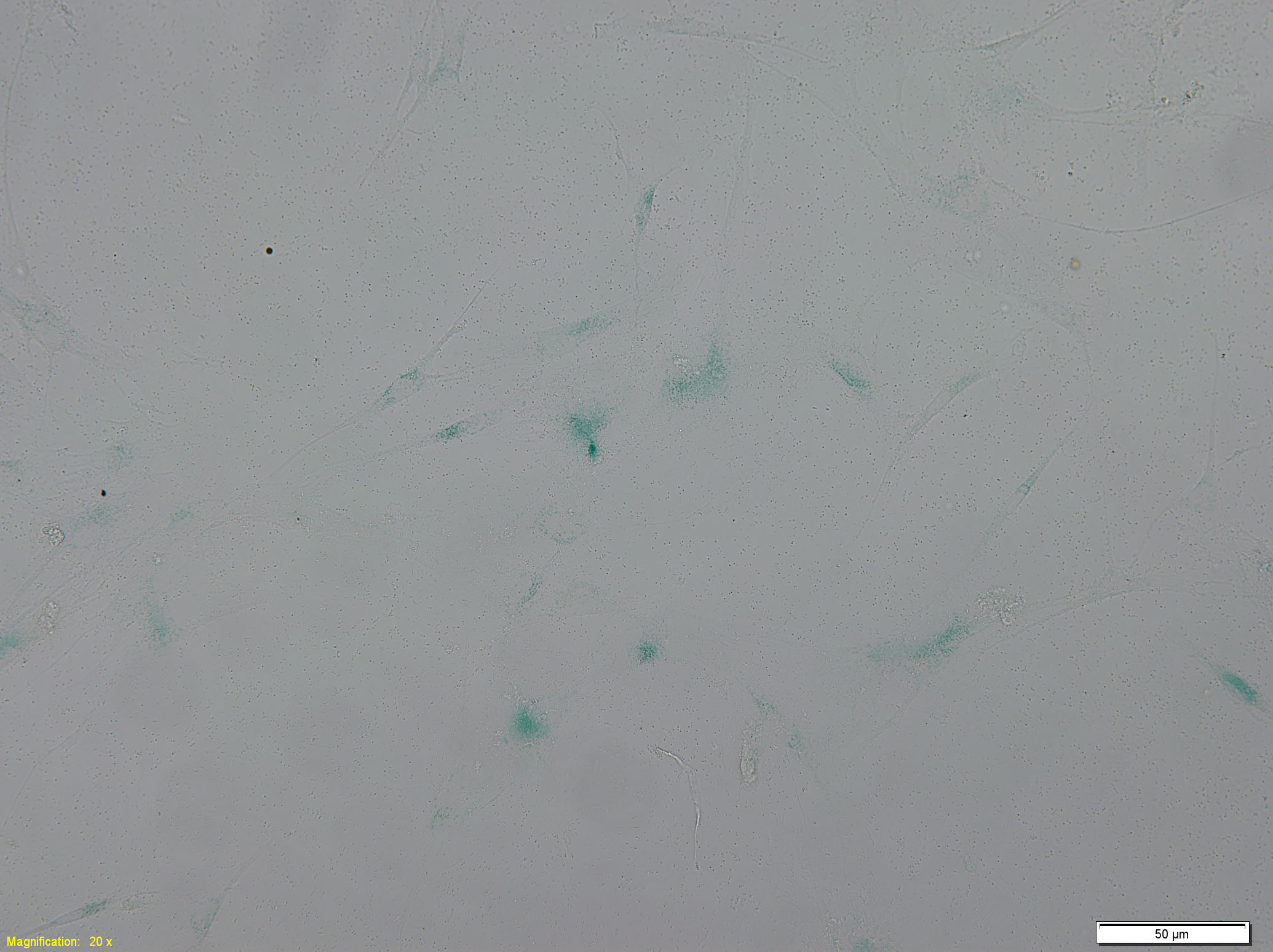

Supplement: Supplemental Information 4 — SA-β-Gal staining of human dental pulp cells with sclerostin overexpression and knockdown. [file peerj-06-5808-s004.zip › SA-B-Gal/sh-SOST/Ctrl/Image_9079.jpg]

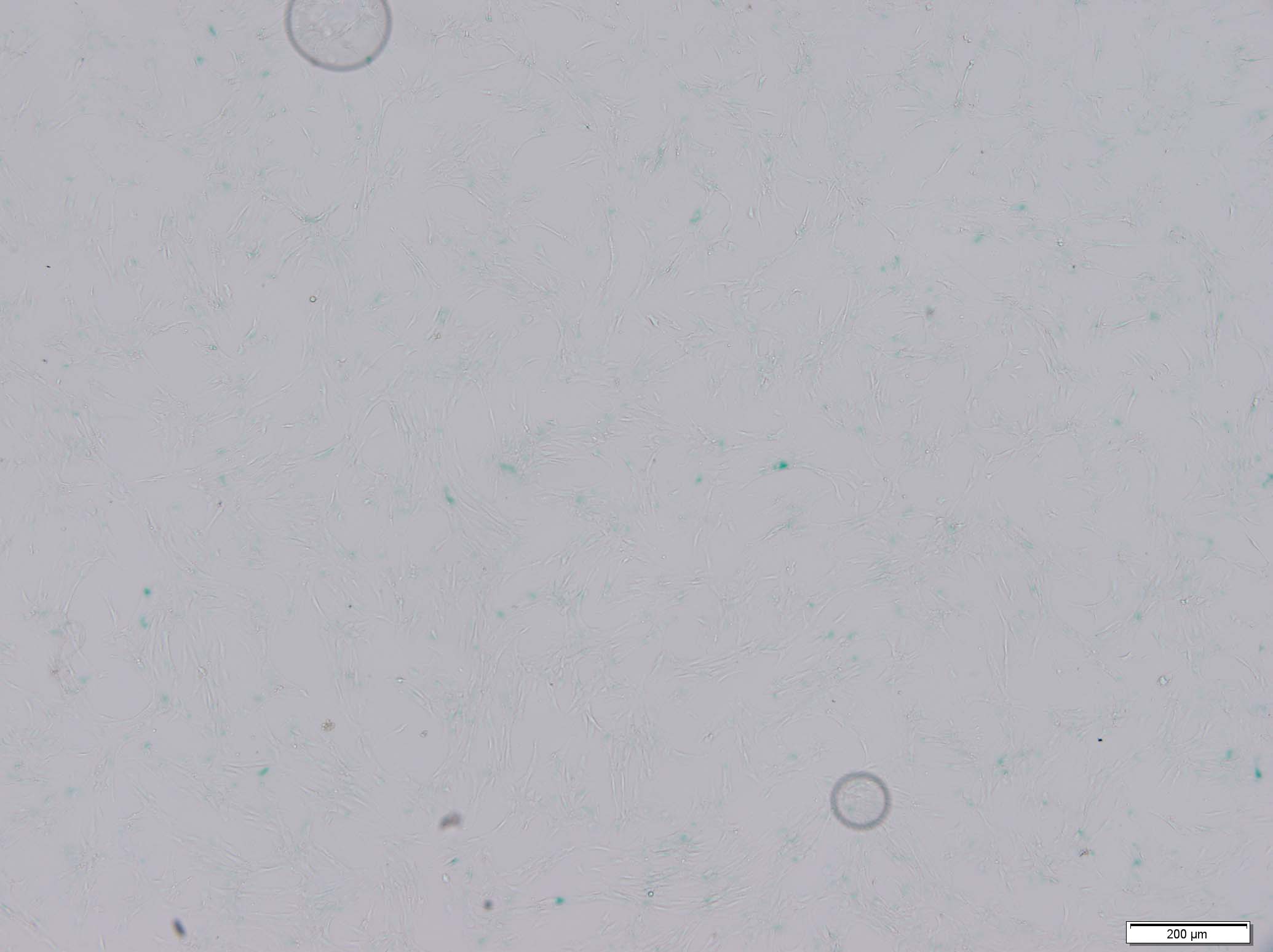

Supplement: Supplemental Information 4 — SA-β-Gal staining of human dental pulp cells with sclerostin overexpression and knockdown. [file peerj-06-5808-s004.zip › SA-B-Gal/sh-SOST/sh-sost/Image_4441.jpg]

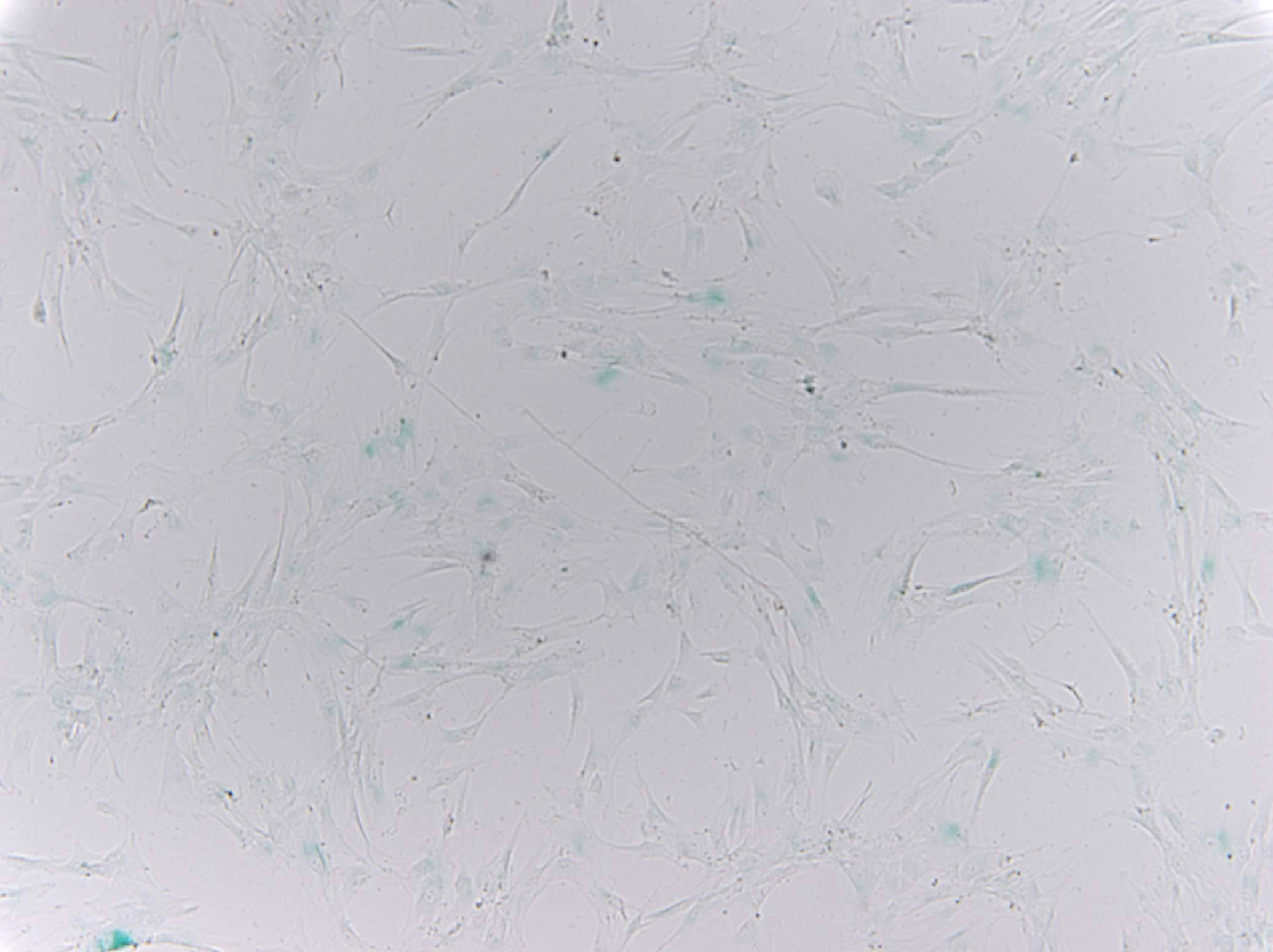

Supplement: Supplemental Information 4 — SA-β-Gal staining of human dental pulp cells with sclerostin overexpression and knockdown. [file peerj-06-5808-s004.zip › SA-B-Gal/sh-SOST/sh-sost/Image_4442.jpg]

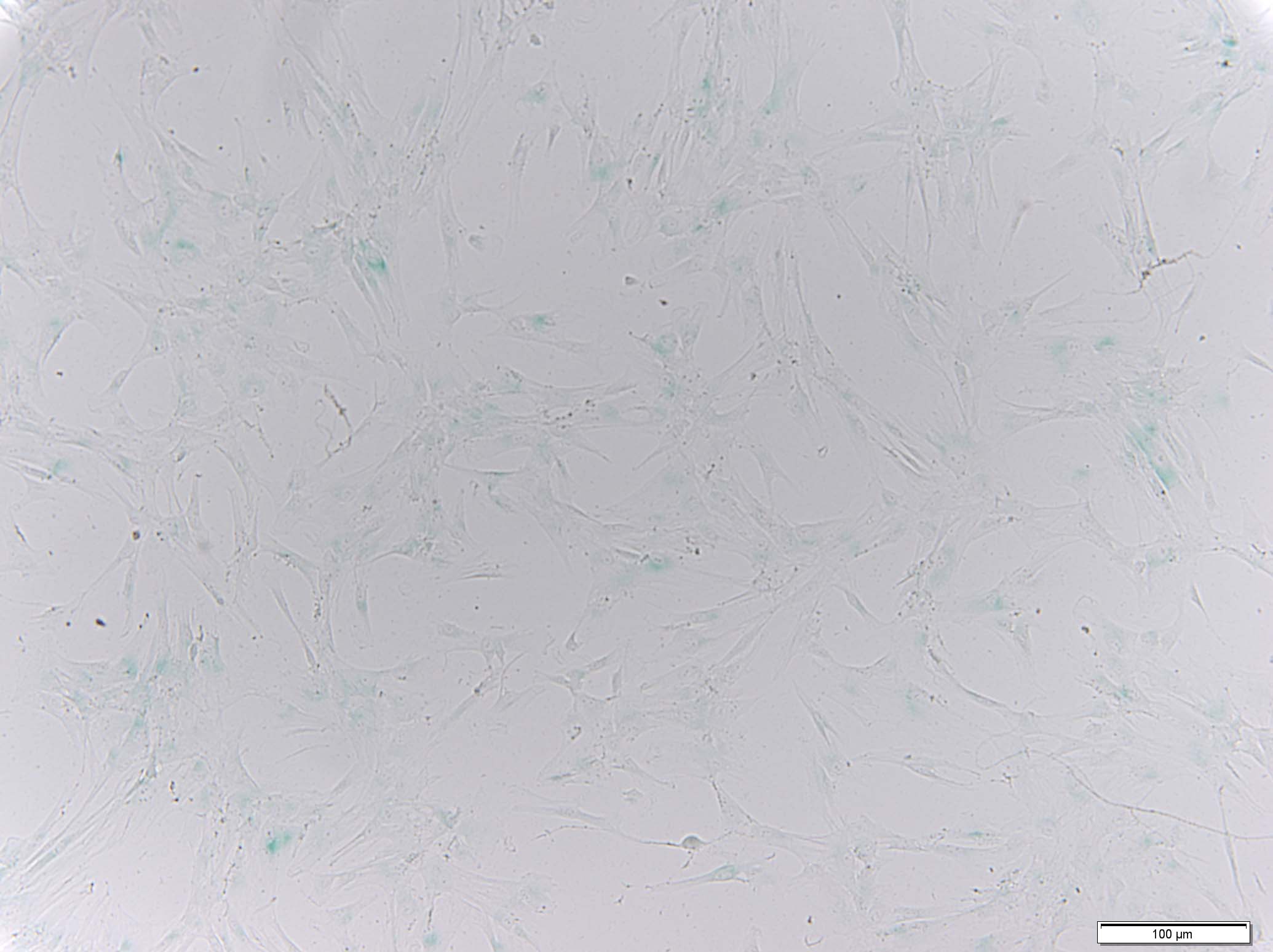

Supplement: Supplemental Information 4 — SA-β-Gal staining of human dental pulp cells with sclerostin overexpression and knockdown. [file peerj-06-5808-s004.zip › SA-B-Gal/sh-SOST/sh-sost/Image_4444.jpg]

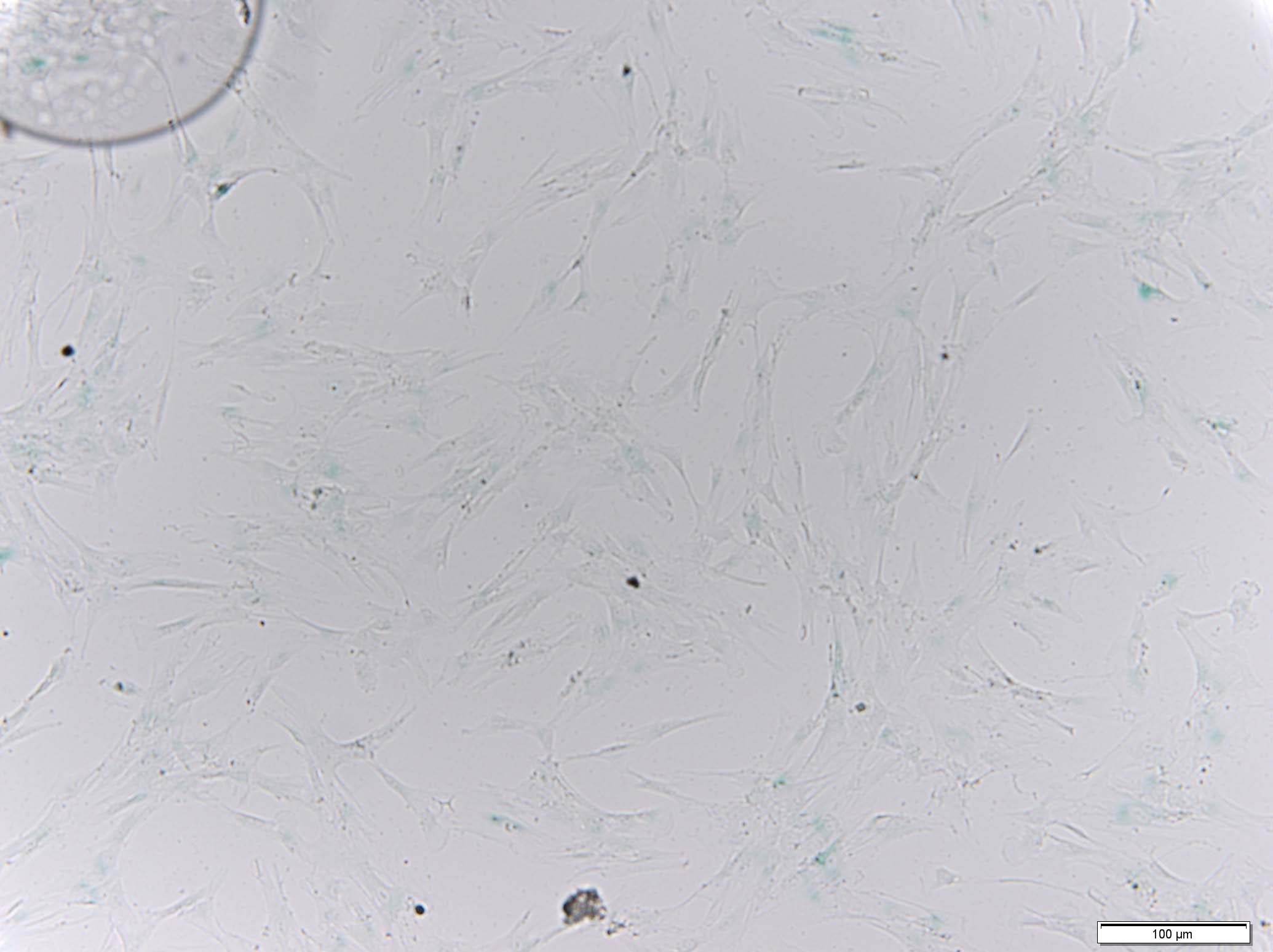

Supplement: Supplemental Information 4 — SA-β-Gal staining of human dental pulp cells with sclerostin overexpression and knockdown. [file peerj-06-5808-s004.zip › SA-B-Gal/sh-SOST/sh-sost/Image_4445.jpg]

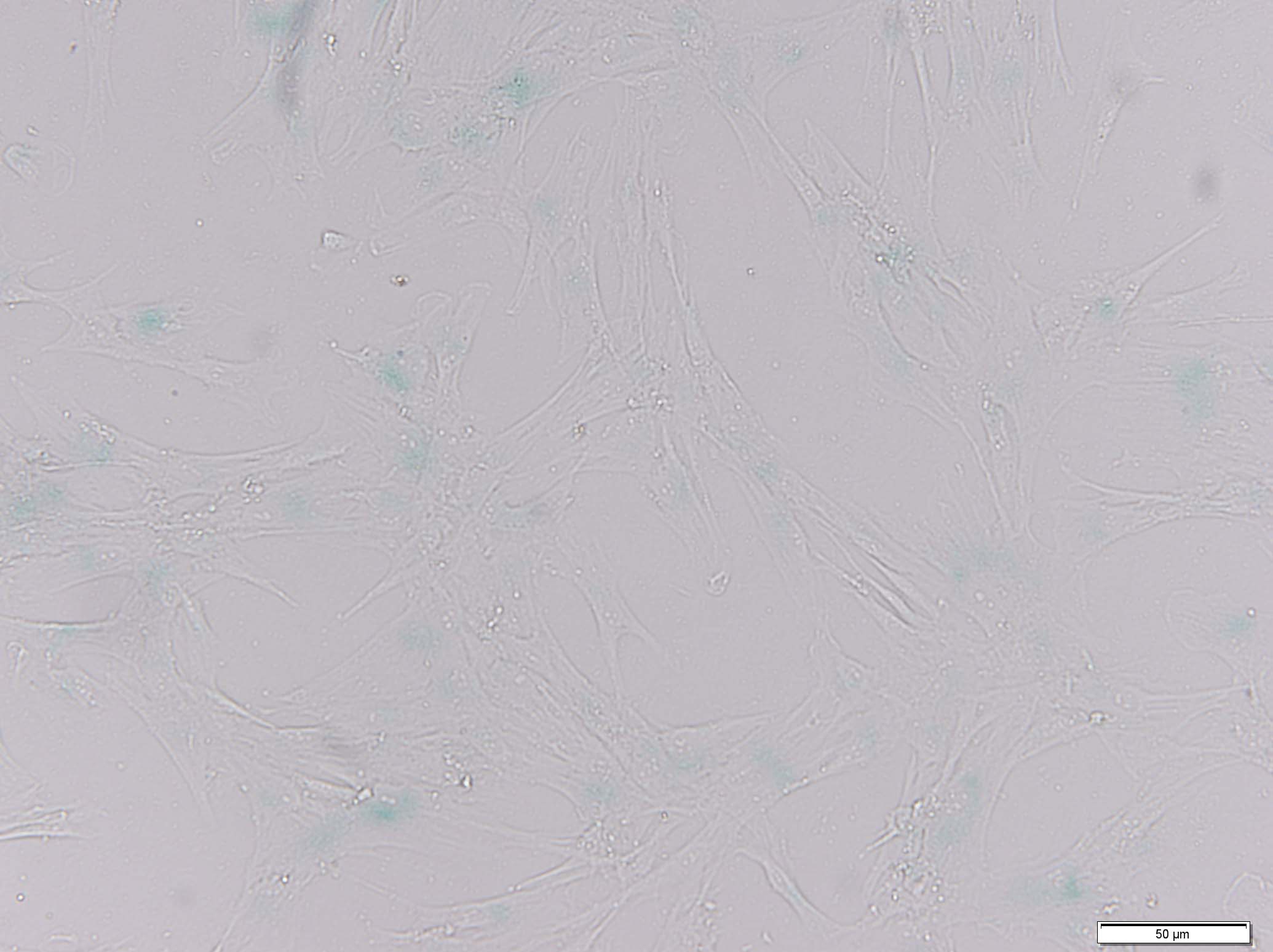

Supplement: Supplemental Information 4 — SA-β-Gal staining of human dental pulp cells with sclerostin overexpression and knockdown. [file peerj-06-5808-s004.zip › SA-B-Gal/sh-SOST/sh-sost/Image_4446.jpg]

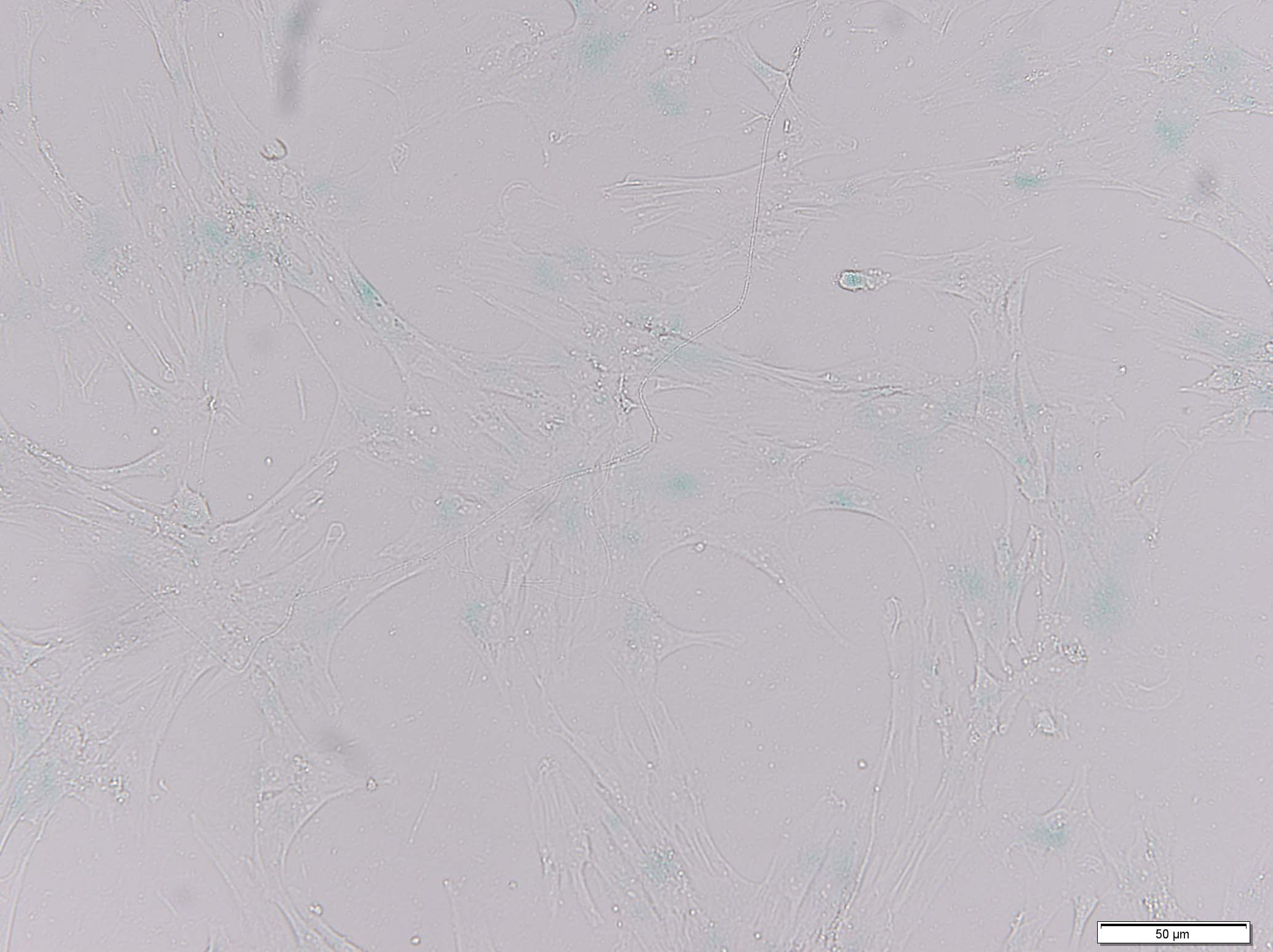

Supplement: Supplemental Information 4 — SA-β-Gal staining of human dental pulp cells with sclerostin overexpression and knockdown. [file peerj-06-5808-s004.zip › SA-B-Gal/sh-SOST/sh-sost/Image_4447.jpg]

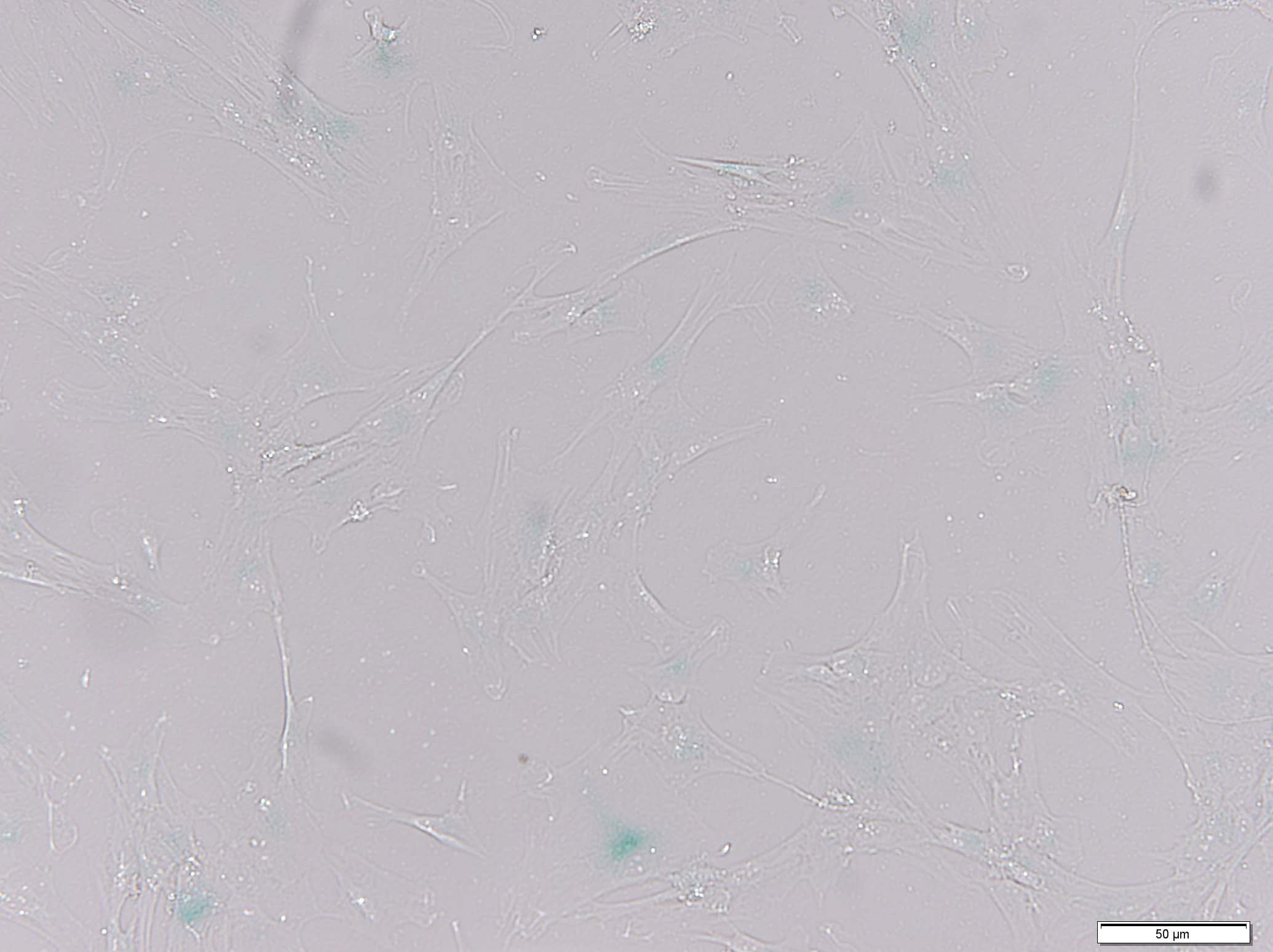

Supplement: Supplemental Information 4 — SA-β-Gal staining of human dental pulp cells with sclerostin overexpression and knockdown. [file peerj-06-5808-s004.zip › SA-B-Gal/sh-SOST/sh-sost/Image_4448.jpg]

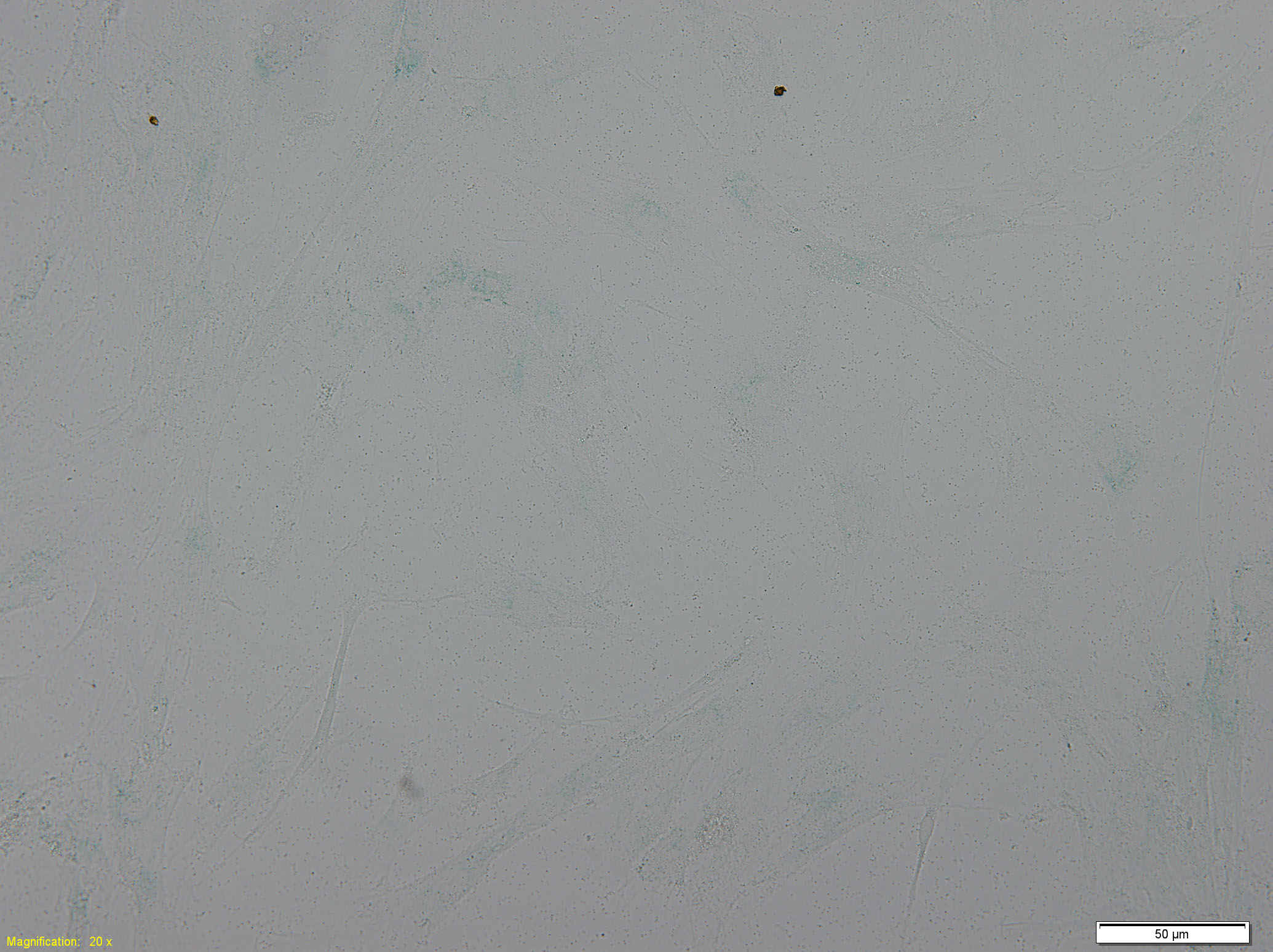

Supplement: Supplemental Information 4 — SA-β-Gal staining of human dental pulp cells with sclerostin overexpression and knockdown. [file peerj-06-5808-s004.zip › SA-B-Gal/sh-SOST/sh-sost/Image_9082.jpg]

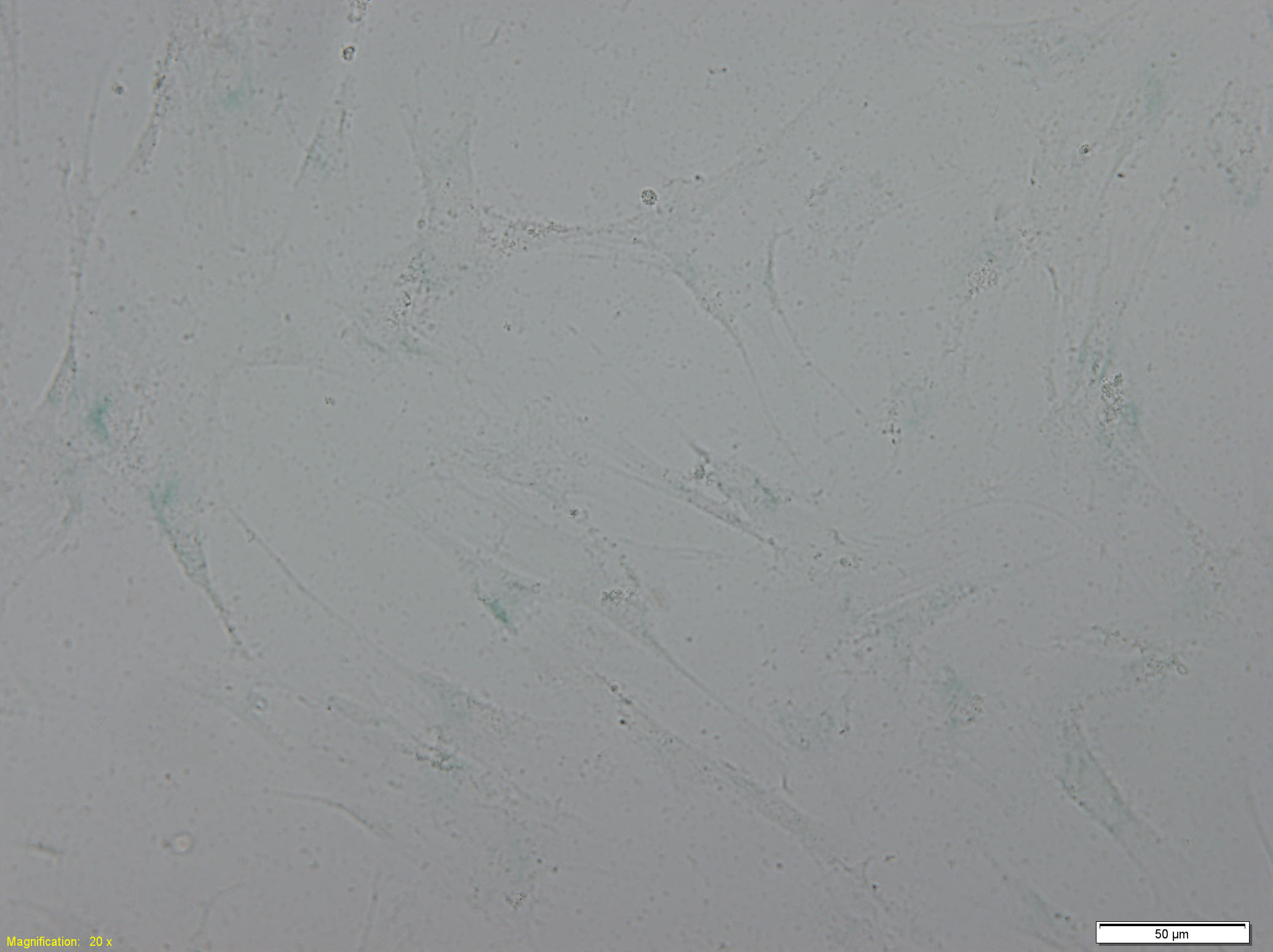

Supplement: Supplemental Information 4 — SA-β-Gal staining of human dental pulp cells with sclerostin overexpression and knockdown. [file peerj-06-5808-s004.zip › SA-B-Gal/sh-SOST/sh-sost/Image_9083.jpg]

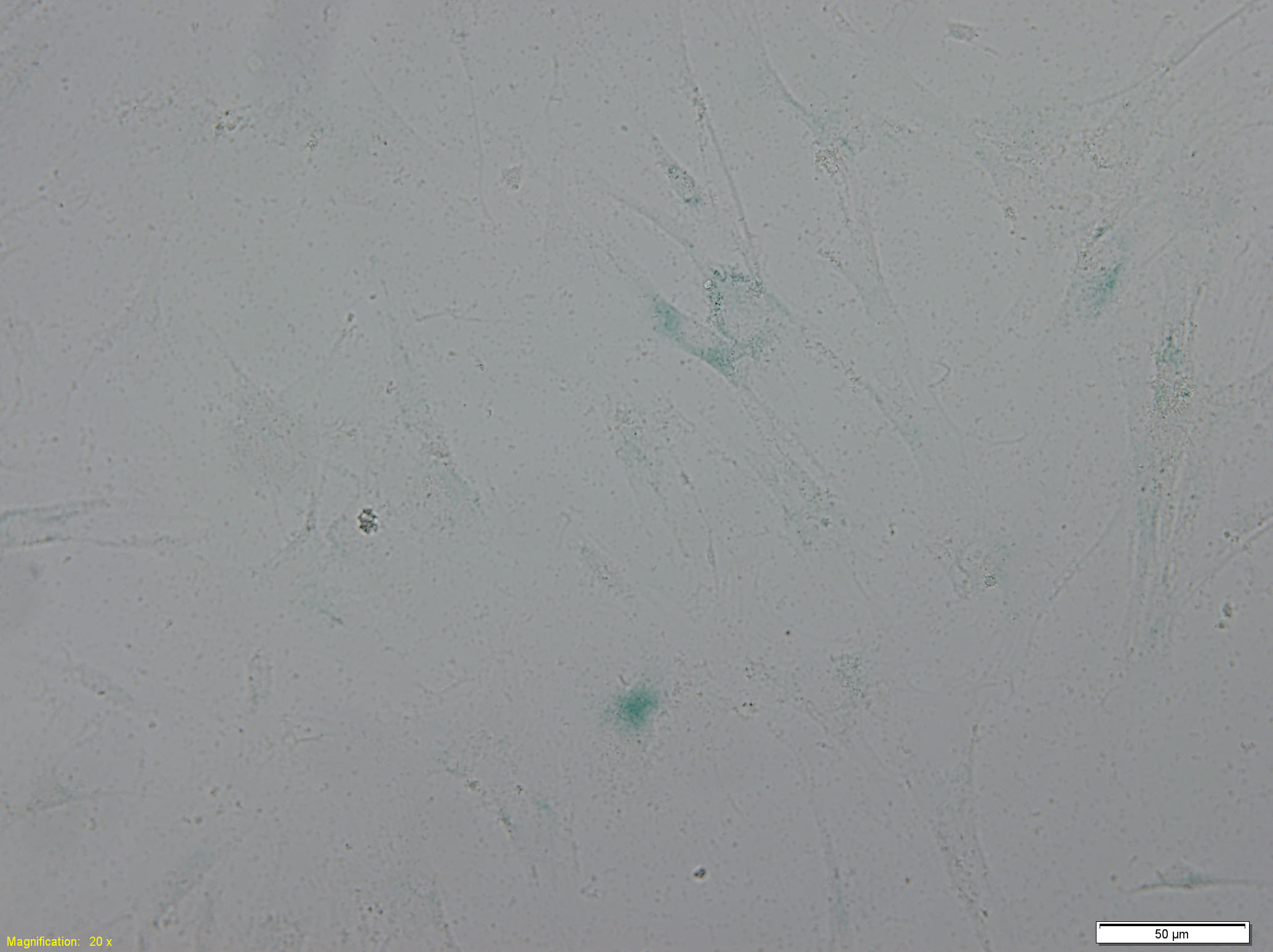

Supplement: Supplemental Information 4 — SA-β-Gal staining of human dental pulp cells with sclerostin overexpression and knockdown. [file peerj-06-5808-s004.zip › SA-B-Gal/sh-SOST/sh-sost/Image_9084.jpg]

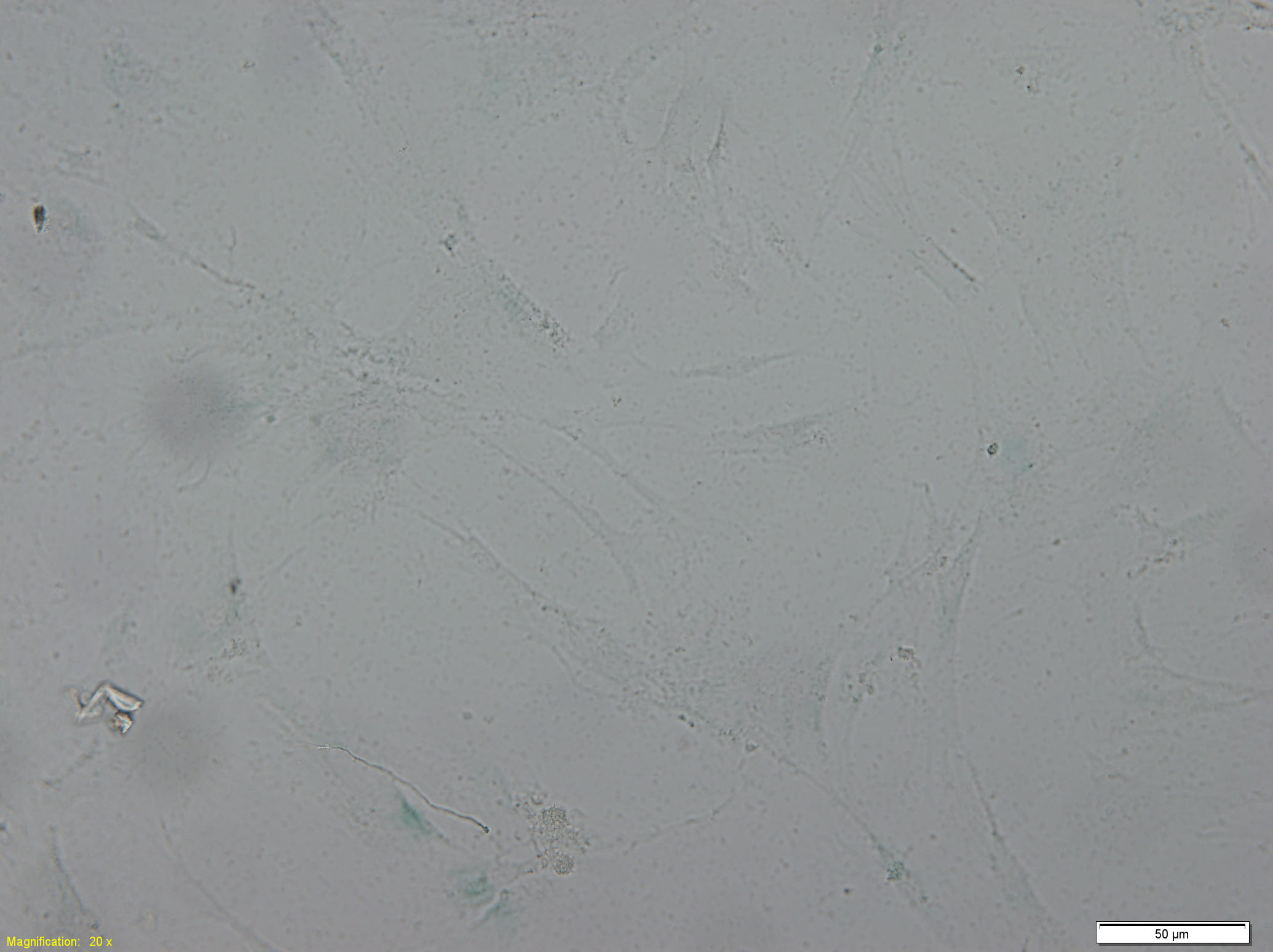

Supplement: Supplemental Information 4 — SA-β-Gal staining of human dental pulp cells with sclerostin overexpression and knockdown. [file peerj-06-5808-s004.zip › SA-B-Gal/sh-SOST/sh-sost/Image_9085.jpg]

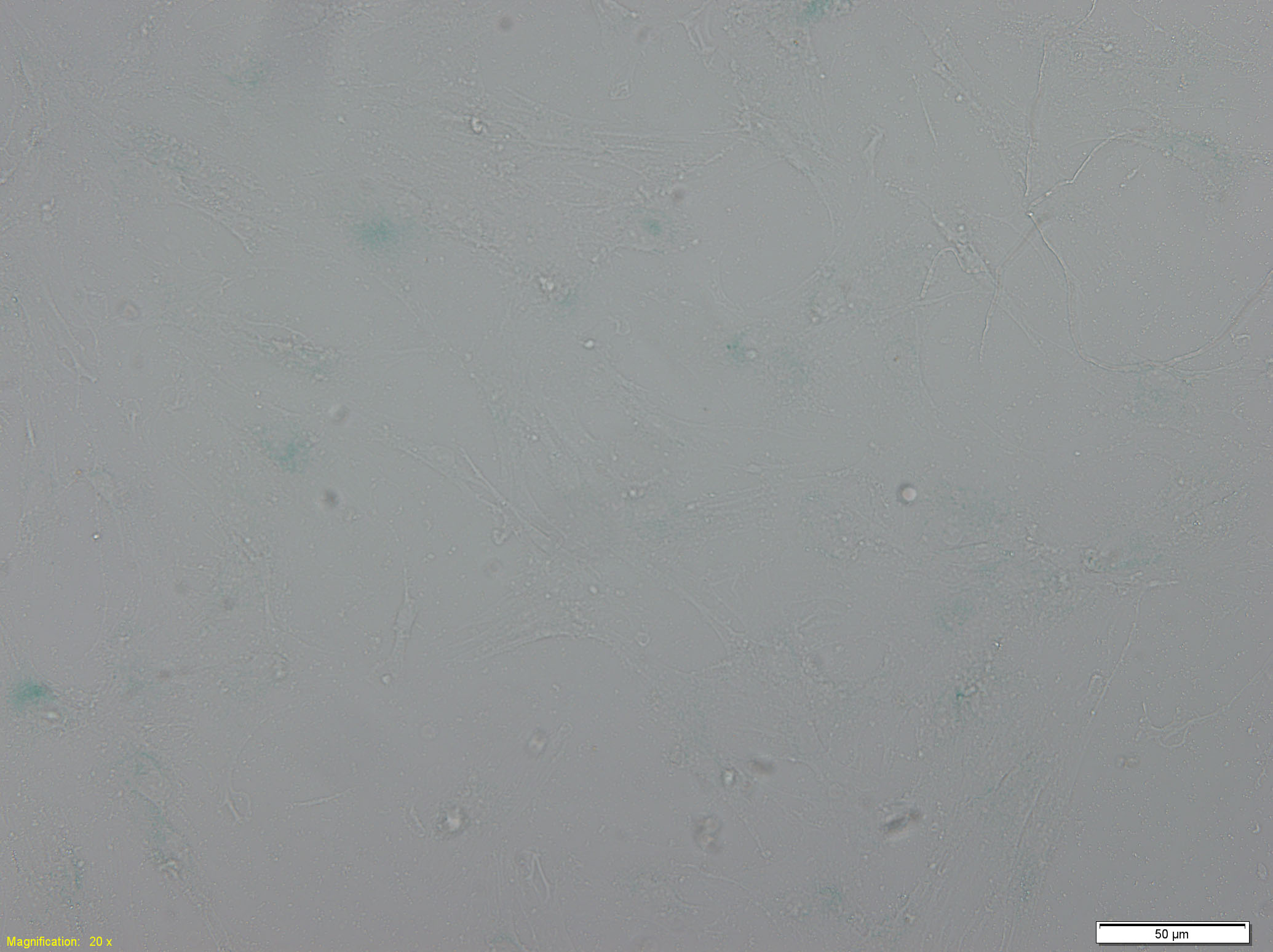

Supplement: Supplemental Information 4 — SA-β-Gal staining of human dental pulp cells with sclerostin overexpression and knockdown. [file peerj-06-5808-s004.zip › SA-B-Gal/sh-SOST/sh-sost/Image_9086.jpg]

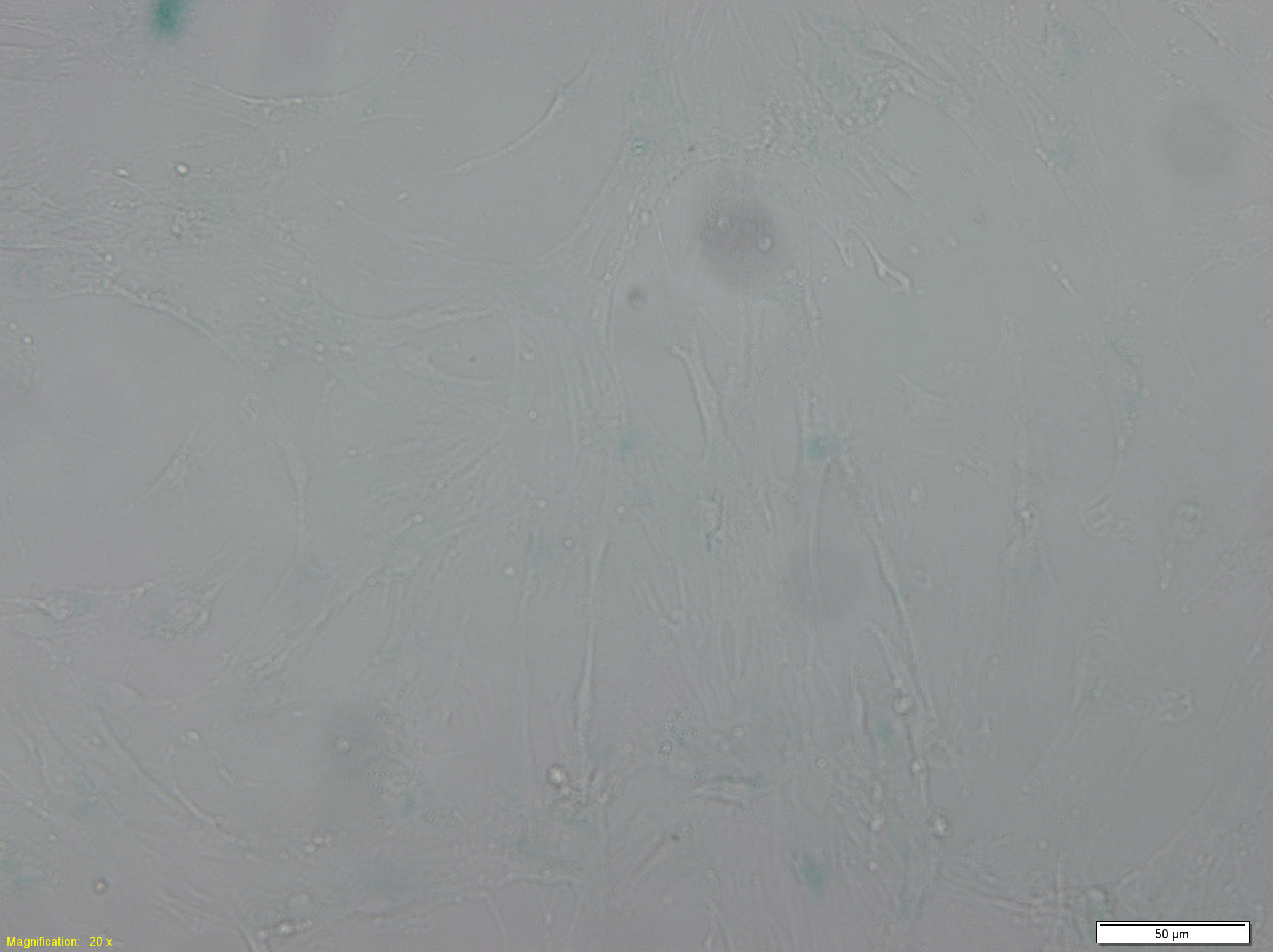

Supplement: Supplemental Information 4 — SA-β-Gal staining of human dental pulp cells with sclerostin overexpression and knockdown. [file peerj-06-5808-s004.zip › SA-B-Gal/sh-SOST/sh-sost/Image_9087.jpg]

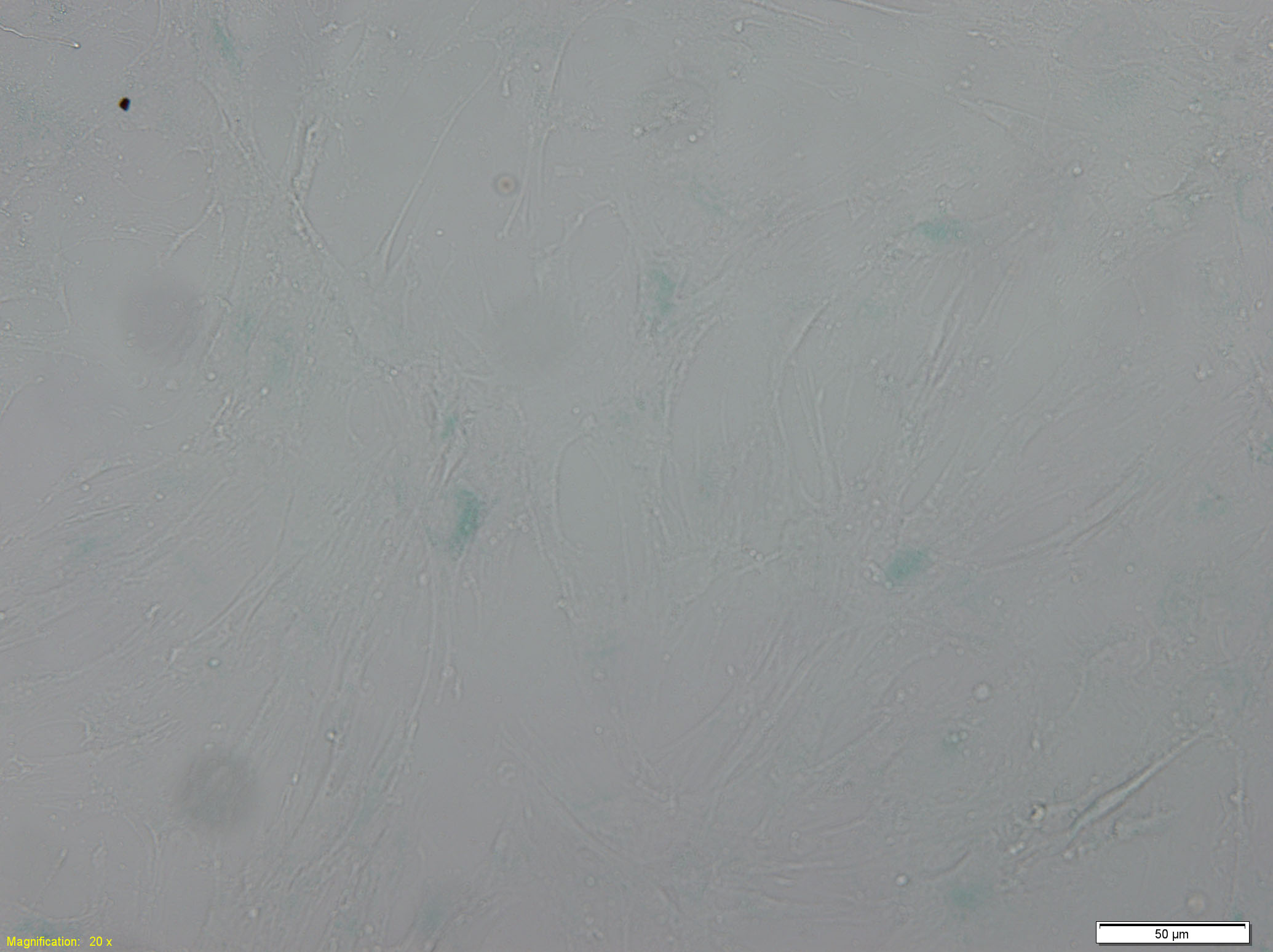

Supplement: Supplemental Information 4 — SA-β-Gal staining of human dental pulp cells with sclerostin overexpression and knockdown. [file peerj-06-5808-s004.zip › SA-B-Gal/sh-SOST/sh-sost/Image_9088.jpg]

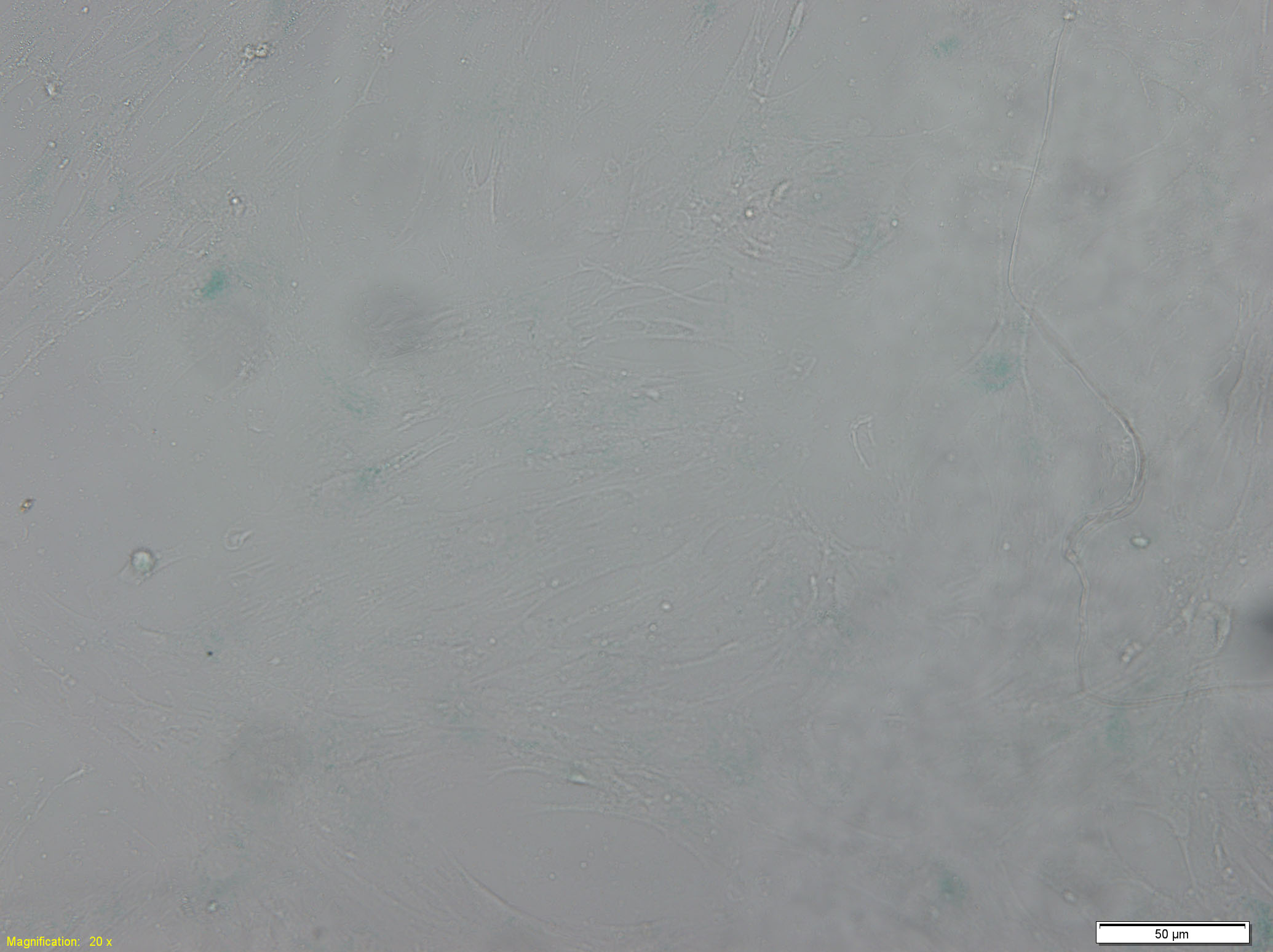

Supplement: Supplemental Information 4 — SA-β-Gal staining of human dental pulp cells with sclerostin overexpression and knockdown. [file peerj-06-5808-s004.zip › SA-B-Gal/sh-SOST/sh-sost/Image_9089.jpg]

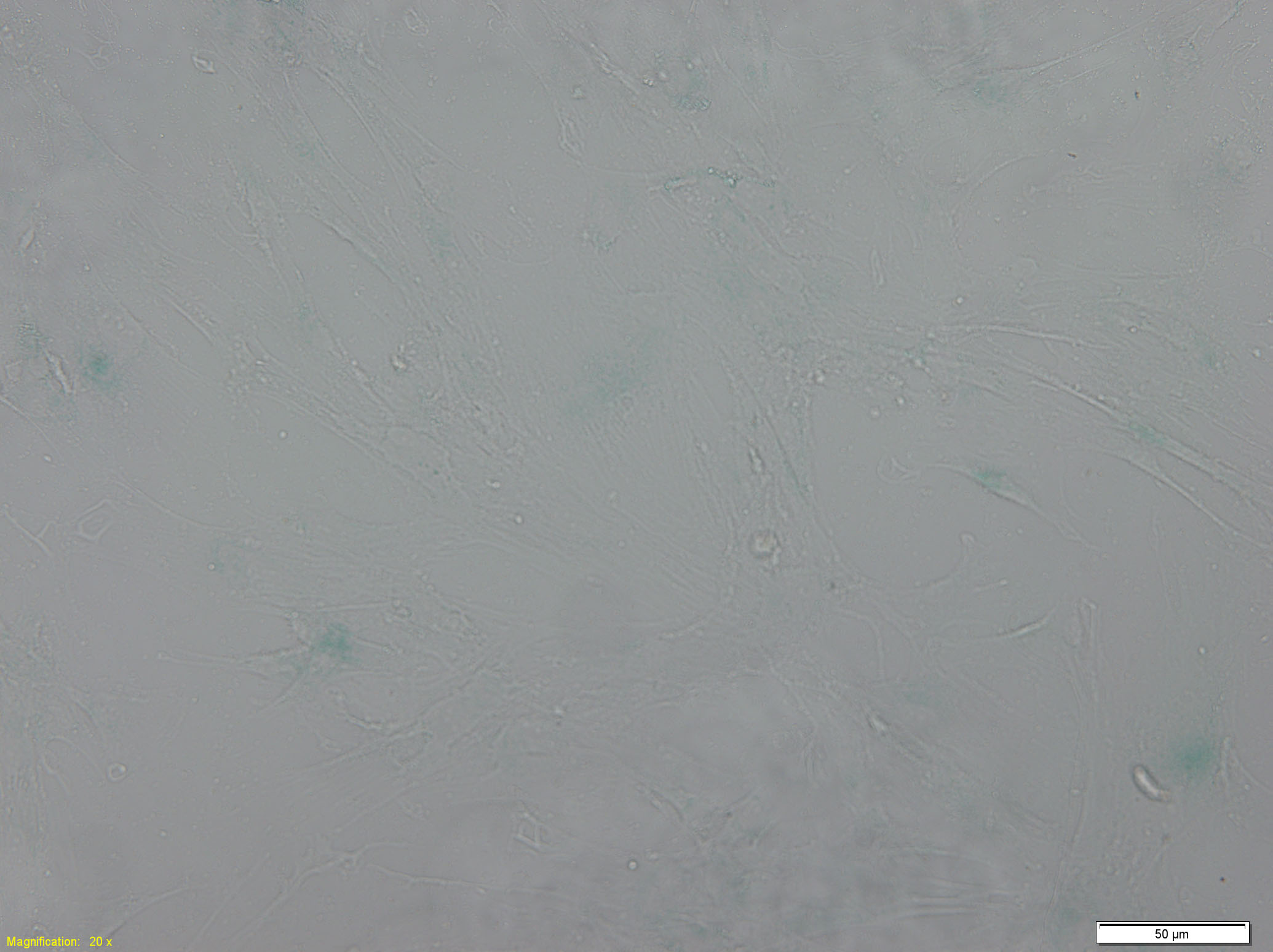

Supplement: Supplemental Information 4 — SA-β-Gal staining of human dental pulp cells with sclerostin overexpression and knockdown. [file peerj-06-5808-s004.zip › SA-B-Gal/sh-SOST/sh-sost/Image_9090.jpg]
